# Mitochondria-derived vesicles with bioenergetic units from brown adipose tissue attenuate cardiac remodeling post-myocardial infarction

**DOI:** 10.1038/s41467-026-73388-3

**Published:** 2026-05-21

**Authors:** Tingting Shi, Feng Chen, Yue Xu, Kai Jiang, Ruhua Deng, Xingbo Yang, Yang Chen, Wenliang Che, John Hwa, Dandan Wang, Yaozu Xiang

**Affiliations:** 1 Department of Cardiology, Shanghai Tenth People’s Hospital, Tongji University School of Medicine, Shanghai, China; 2 State Key Laboratory of Cardiology and Medical Innovation Center, Shanghai East Hospital, Frontier Science Center for Stem Cell Research, Department of Biomedicine, School of Life Sciences and Technology, Tongji University, Shanghai, China; 3 Yale Cardiovascular Research Center, Section of Cardiovascular Medicine, Department of Internal Medicine, Yale University School of Medicine, New Haven, CT USA

**Keywords:** Organelles, Cardiovascular biology, Mechanisms of disease

## Abstract

Post-myocardial infarction remodeling is a major cause of heart failure, with contributions from multiple organs. Brown adipose tissue protects against cardiovascular disease, but the mediators of brown adipose tissue-heart crosstalk and their roles in cardiac remodeling remain elusive. Here, we show that mitochondria-derived vesicles from brown adipose tissue transfer to cardiac macrophages and attenuate pathological remodeling via anti-inflammatory effects. Vesicles containing mitochondrial membranes, rather than mitochondrial matrix, mobilize from brown adipose tissue to the heart in response to stress. VPS35 translocation to mitochondria drives protein packaging into mitochondria-derived vesicles for secretion through extracellular vesicle trafficking machinery. *Becn1* deficiency impairs VPS35 translocation, alters mitochondria-derived vesicle cargo, and abolishes brown adipose tissue-mediated cardioprotection. Proteomics identifies mitochondrial respiratory chain complex V as a hallmark of protective mitochondria-derived vesicles. These vesicles enhance reparative cytokine production and oxidative phosphorylation rewiring in macrophages. Purified mitochondria-derived vesicles markedly improve remodeling in male mice. Our study uncovers an interorgan transfer of bioenergetic units that contributes to tissue repair.

## Abstract (web summary)

Brown adipose tissue protects against cardiovascular disease. Here, the authors show that brown adipose tissue mitigates post-infarction cardiac remodeling through mitochondria-derived vesicles that deliver bioenergetic cargo and promote reparative macrophage polarization.

## Introduction

Ischemic heart disease and its complications remain the leading cause of death worldwide^1,2^. Post-myocardial infarction (MI) adverse remodeling, characterized by fibrosis and progressive loss of systolic function, is a major determinant of heart failure and mortality. Beyond timely revascularization, there is a need for adjunctive strategies that modulate systemic responses to MI and thereby limit chronic fibrotic remodeling.

Emerging evidence indicates that the development of heart failure is governed by a coordinated interorgan network that includes brown adipose tissue (BAT). Clinical imaging studies suggest that metabolically active BAT is associated with a lower prevalence of type 2 diabetes, coronary artery disease, heart failure, and hypertension^3,4^, raising the possibility that enhancing BAT-heart communication could be cardioprotective.

Several BAT-derived endocrine factors have been implicated in this crosstalk. These endocrine factors, also known as BATokines^5–10^, including fibroblast growth factor 21 (FGF21), interleukin-6 (IL-6), neuregulin 4 (NRG4), 12,13-dihydroxy-9Z-octadecenoic acid (12,13-diHOME), bone morphogenetic protein 3b (BMP3b), have been shown to modulate cardiac hypertrophy, metabolism, and response to hemodynamic or ischemic stress. For example, activation of the brown adipocyte A₂A receptor-FGF21 axis attenuates hypertensive cardiac remodeling^5^, and 12,13-diHOME improves cardiac performance in exercising mice by enhancing cardiomyocyte calcium cycling and mitochondria respiration^6^. In addition, BAT-derived exosomes delivering iNOS have been implicated in protection from angiotensin II-induced fibrosis^8^, and BAT-derived small extracellular vesicles (sEVs) and microRNAs are required for exercise-induced cardioprotection^9^. These studies have established BAT as an endocrine and vesicular modulator of cardiac injury.

As a major mitochondria reservoir, brown adipocytes are rich in mitochondria and tend to eject their mitochondria under stress^11–14^. Mitochondria-derived vesicles (MDVs) have been observed to traffic from brown adipocytes to immune cells within BAT, and high-fat diet increases the spill-over of mitochondrial components into the circulation and distant organs, including the heart^14^. This process is associated with BAT thermogenesis, glucose consumption, and lipid accumulation. However, the role of MDVs in BAT-heart crosstalk and heart failure (HF) development is incompletely understood.

We have now identified oxidative phosphorylation (OXPHOS) proteins-enriched MDV transfer from BAT to the injured heart as part of a cross-organ protective network. Using BAT pericardial transplantation and multiple labeling systems, we show that MI enhances the biogenesis and release of MDVs from BAT, and that these vesicles are taken up by cardiac macrophages, where they modulate inflammatory and reparative states. Proteomic analysis reveals that BAT-derived MDVs are enriched for OXPHOS proteins, and functional studies indicate that their cardioprotective effects depend on the BECN1 in brown adipocytes and on intact ATP synthase (complex V) cargo. These findings expand the paradigm of BAT-heart crosstalk by introducing BAT-derived, OXPHOS-rich MDVs as a mitochondria-based mechanism for limiting post-MI cardiac remodeling.

## Results

### BAT transplantation attenuates adverse post-MI remodeling

**Fig. 1:**
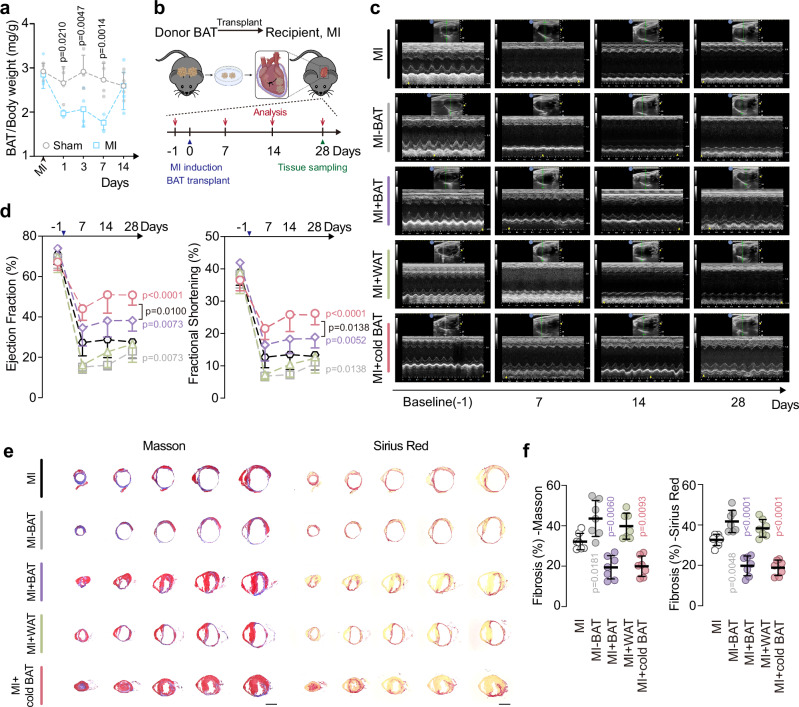
BAT transplantation limits post-MI cardiac remodeling. **a** Mouse BAT weight proportion over the course of MI (mean ± s.d., *n* = 4 mice for each time point, repeated two-way ANOVA with Holm-Sidak’s multiple-comparisons test). **b** Experimental timeline for MI induction, adipose tissue pericardial transplantation, and outcome assessments. Echocardiography was performed at baseline (day -1) and on days 7, 14, and 28. Hearts were harvested on day 28 for Masson’s trichrome and Sirius Red staining. Created in BioRender. Shi, T. (2026) https://BioRender.com/8asl17d.**c, d** Representative B-mode and M-mode echocardiography of BAT-receiving mice at baseline and days 7, 14, and 28 (**c**). Ejection fraction and fractional shortening were quantified (**d**) (mean ± s.d., *n* = 7 mice per group, repeated two-way ANOVA with Holm-Sidak’s multiple-comparisons test). **e, f** Representative Masson and Sirius Red staining images of mouse hearts at day 28. Fibrosis percentage was quantified (mean ± s.d., *n* = 7 mice per group, one-way ANOVA with Bonferroni correction). Scale bar: 2 mm. Source data are provided as a Source Data file.

Myocardial infarction induced by left anterior descending (LAD) ligation caused an acute reduction in BAT mass, which gradually recovered to near-baseline levels by two weeks post-MI (Fig. 1a). This transient BAT atrophy occurs within the same time window as early post-injury remodeling.

To test whether augmenting BAT during the acute phase of MI can influence cardiac outcomes, we used an interscapular BAT (iBAT) transplantation model. Small iBAT grafts (1-2 mm in diameter) were excised from the donor interscapular depot, carefully trimmed to facilitate revascularization, and placed into the pericardial cavity of recipient mice undergoing LAD ligation in the same surgical session (Fig. 1b and Supplementary Fig. 1a). Laser speckle blood-flow imaging confirmed reperfusion of the transplanted BAT (Supplementary Fig. 1b), and histological analysis showed that the grafts retained characteristic BAT morphology, indicating successful engraftment (Supplementary Fig. 1c, d).

Pericardial BAT transplantation mitigated post-MI systolic decline. Across serial echocardiography at days 7, 14, and 28, BAT-treated mice maintained higher ejection fraction and fractional shortening than MI controls, indicating sustained preservation of contractile performance (Fig. 1c, d). Concordant structural benefits were observed on Masson’s trichrome and Picrosirius Red staining, which showed a smaller fibrotic area and better myocardial preservation in the BAT group (Fig. 1e, f). Consistently, BAT-deficient mice (MI-BAT, iBAT excision 1 week before MI) exhibited significantly lower ejection fraction (EF) and fractional shortening (FS) and greater fibrosis than MI controls, supporting an essential role of endogenous BAT in limiting post-MI remodeling (Fig. 1d, f).

As further validation, we compared different adipose grafts. White adipose tissue (WAT) and BAT arise from distinct adipocyte lineages and exert divergent metabolic and cardiovascular effects^3^. WAT transplantation did not modify MI-induced fibrosis or systolic function, whereas BAT transplantation reduced scar burden and preserved contractile performance (Fig. 1c–f), indicating BAT transplantation-related cardioprotection. To test whether BAT activation augments this benefit, donor mice were cold-exposed at 4 °C for 14 days before grafting. MI+cold BAT mice showed higher EF and FS than MI+BAT mice, while fibrosis burden remained comparable between the two groups (Fig. 1d, f). Collectively, these findings support a BAT-related, thermogenesis-enhanced cardioprotective effect on early post-MI remodeling.

### BAT-MDVs transferred to cardiac macrophages

**Fig. 2:**
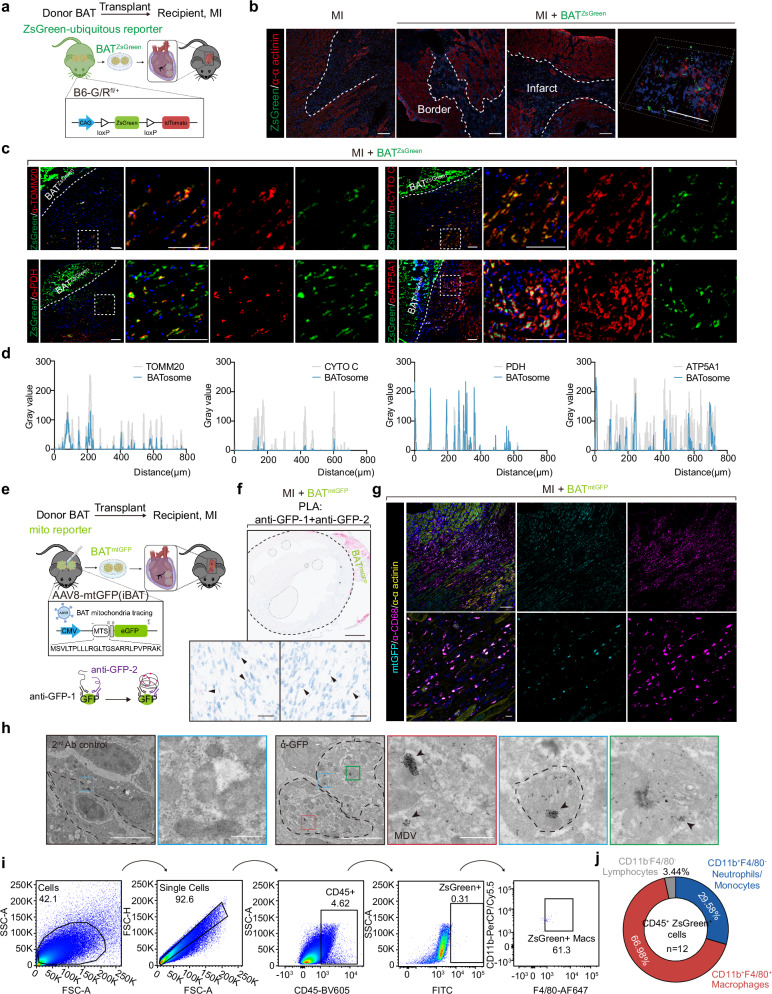
BAT-derived MDVs transfer to cardiac macrophages in infarcted myocardium. **a** Schematics of pericardial BAT transplantation from ZsGreen-ubiquitous fluorescent reporter donor mice (B6-G/R^fl/+^) into wild-type MI recipient mice. Created in BioRender. Shi, T. (2026) https://BioRender.com/755v3rv. **b** Representative confocal images of left ventricles 4 days after MI in mice with or without BAT^ZsGreen^ transplantation, stained for sarcomeric α-actinin (red) and nuclei (DAPI, blue). 3D reconstruction of the infarct zone of BAT^ZsGreen^-receiving mice. Scale bar: 100 μm. The experiment was independently repeated 3 times with similar results. **c, d** Representative confocal images of left ventricles in BAT^ZsGreen^-receiving mice at sham or MI 4 d, stained for TOMM20 or Cytochrome C or PDH or ATP5A1 (red), and nuclei (DAPI, blue). Co-localization analysis of ZsGreen⁺ BATosomes with mitochondrial markers in the infarct myocardium. Scale bar: 100 μm. The experiment was independently repeated 3 times with similar results. **e** Schematic illustration of the BAT transplant from BAT mitochondria-labeled mice to wild-type (WT) MI mice. AAV8-MTS-eGFP was injected in situ into interscapular BAT to generate mitochondria-labeled donor BAT (BAT^mtGFP^). Created in BioRender. Shi, T. (2026) https://BioRender.com/pr99kl5. **f** Representative bright-field images of proximity ligation assay using two anti-GFP antibodies to detect GFP protein in BAT^mtGFP^-receiving mice at MI 4 d, with positive signals visualized by alkaline phosphatase chromogenic reaction (pink). Scale bar: 1 mm; 25 μm (magnification). The experiment was independently repeated 3 times with similar results. **g** Representative confocal images of infarct myocardium 4 days after MI in BAT^mtGFP^-receiving mice. Sections were stained for sarcomeric α-actinin (yellow), CD68 (magenta), and nuclei (DAPI, blue). Upper scale bar: 100 μm; lower scale bar: 10 μm. The experiment was independently repeated 3 times with similar results. **h** Immuno-electron microscopy of the infarct area of BAT^mtGFP^-receiving mice, stained for GFP (black electron-dense gold particles). Scale bar: 5 μm; 500 nm (magnification). The experiment was independently repeated 3 times with similar results. **i** Flow-cytometric gating strategy for identifying BATosome-recipient immune cells in infarcted hearts from BAT^ZsGreen^-receiving mice at MI 4 d. **j** Quantification of immune cell subsets among ZsGreen⁺CD45⁺ cells in infarcted hearts from BAT^ZsGreen^-receiving mice at MI 4 d. Data are presented as mean percentages of each subset among total ZsGreen⁺CD45⁺ cells (*n* = 12 mice). Source data are provided as a Source Data file.

Brown adipocytes are mitochondria-rich cells that can release mitochondria-derived vesicles (MDVs) in response to metabolic or inflammatory stress^11–14^. Together with reports of inter-organ MDV trafficking from brown adipose tissue, these observations led us to hypothesize that brown adipose tissue-derived MDVs are exported from BAT and delivered to the ischemic myocardium after MI. To trace BAT-derived material in vivo, we transplanted ZsGreen-ubiquitous reporter BAT (H11-CAG-LoxP-ZsGreen-Stop-LoxP-tdTomato; B6-G/R^fl/+^) into the pericardial cavity of wild-type mice subjected to MI induction. Four days after MI, ZsGreen⁺ signals were detected in the border and infarct regions of BAT^ZsGreen^ recipients, whereas no such signal was detected in MI-only controls (Fig. 2a, b, and Supplementary Fig. 2a). These signals appeared as discrete intracellular puncta on three-dimensional visualization.

To further characterize these signals, we examined their spatial association with mitochondrial protein markers. In BAT^ZsGreen^-recipient hearts, ZsGreen⁺ puncta exhibited marked co-localization with TOMM20 (outer mitochondrial membrane), cytochrome c (intermembrane space), and ATP5A1 (inner mitochondrial membrane), with comparatively limited overlap with the matrix enzyme PDH (Fig. 2c, d and Supplementary Fig. 2b). These findings support protein-level association of ZsGreen⁺ puncta with multiple mitochondrial compartments.

We next sought to corroborate the mitochondrial origin of BATosomes and their recipient cells using a mitochondrial reporter system. A BAT mitochondria-tracing donor (BAT^mtGFP^) was generated by in situ injection of an AAV8-MTS-eGFP vector into interscapular BAT (Fig. 2e). A GFP-based proximity ligation assay using two anti-GFP antibodies produced chromogenic signals specifically in MI hearts receiving BAT^mtGFP^ when both antibodies were present, whereas single-antibody incubations, GFP-negative BAT, and sham controls were blank (Fig. 2f and Supplementary Fig. 3a), confirming the specificity of the mtGFP signals in BAT^mtGFP^-recipient hearts. At the ultrastructural level, immuno-electron microscopy of BAT^mtGFP^-recipient heart infarcted myocardium revealed electron-dense, gold-labeled GFP⁺ vesicles within the cytoplasm of cells, some in close apposition to or fusing with host mitochondria (Fig. 2h).

We next investigated which cell populations within the infarct zone serve as in vivo recipient cells for BAT-derived MDVs. In the subacute phase after MI, the non-cardiomyocyte compartment of the infarcted myocardium is dominated by innate immune cells (macrophages and neutrophils) and activated fibroblasts^15^. BATosomes accumulated preferentially in regions enriched for CD68⁺ macrophages, whereas co-localization with MPO⁺ neutrophils and ZsGreen signal was largely absent (Supplementary Fig. 2c, d). To further validate this, the BAT^mtGFP^ donor was used. In the BAT^mtGFP^-recipient heart, mtGFP⁺ puncta were abundant within the infarct region and showed marked co-localization with CD68⁺ macrophages, while α-SMA⁺ fibroblasts remained largely mtGFP⁻ (Fig. 2g and Supplementary Fig. 3b). Similar patterns were seen when endogenous BAT was labelled with AAV8-mtGFP, with mtGFP⁺ vesicles again concentrating in CD68⁺ macrophages of the infarcted myocardium (Supplementary Fig. 3c). These complementary reporters therefore converged on macrophages as the dominant cellular destination for BAT-derived MDVs.

Finally, flow cytometric analysis quantitatively confirmed this preferential distribution. In infarcted hearts from BAT^ZsGreen^-recipient mice, the majority of ZsGreen⁺CD45⁺ leukocytes were CD11b⁺F4/80⁺ macrophages, whereas ZsGreen⁺ neutrophils/monocytes and other immune subsets accounted for only a minor fraction of the labelled cells (Fig. 2i, j).

Together, these imaging and cytometric data identify cardiac macrophages as the main recipient cell type for BAT-derived MDVs within the infarct zone.

Although BAT local macrophages can phagocytose mitochondria ejected from brown adipocytes^11–14^, BAT resident immune cells were not observed in BAT-receiving mice (Supplementary Fig. 4a–c). It suggests that BAT resident macrophages were not involved in BAT-MDVs transfer in MI mice with pericardial BAT transplantation.

### BAT-MDVs contribute to MI injury repair

**Fig. 3:**
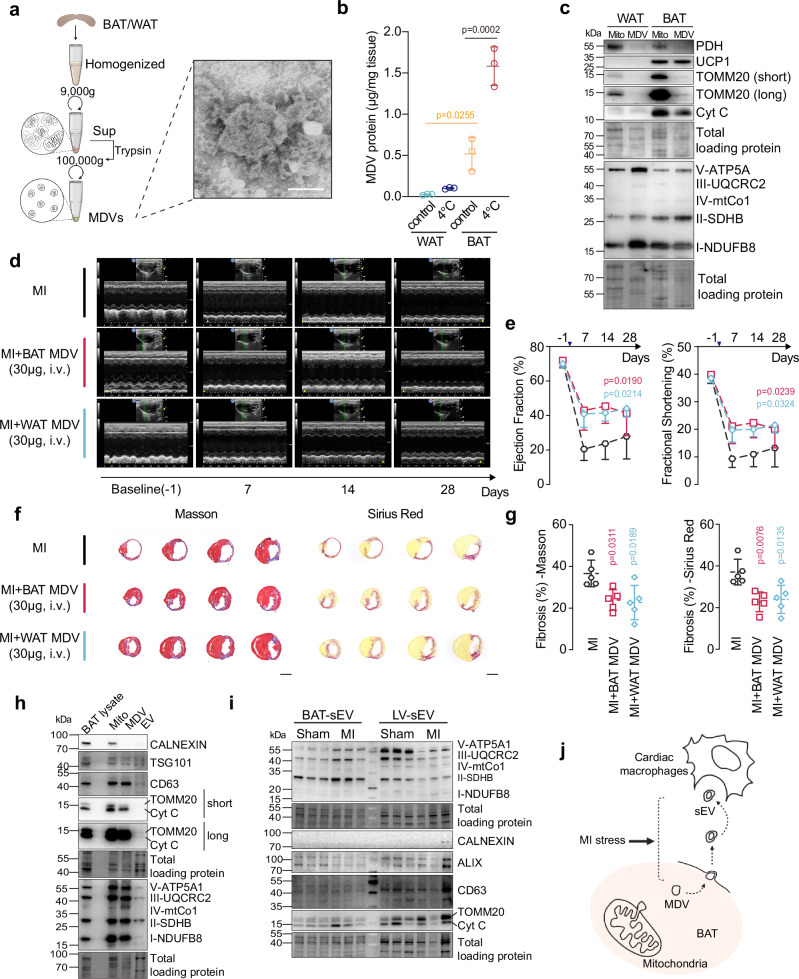
BAT-derived MDVs mediate cardioprotection via EV release post-MI. **a** Schematic of MDVs isolation from mouse adipose tissue. Created in BioRender. Shi, T. (2026) https://BioRender.com/xnpoj8f. Purified MDVs were visualized by negative-stain TEM. Scale bar, 100 nm. **b** Protein quantification of MDVs isolated from WAT or BAT, normalized by tissue weight (mean ± s.d., *n* = 3 mice per group, one-way ANOVA with Bonferroni correction). Mice have been housed at 22 °C (control) or 4 °C for 14 days before tissue harvest. **c** Western blots of purified mitochondria and MDVs from mouse adipose tissue. The experiment was independently repeated on 3 mice with similar results. Representative B-mode and M-mode echocardiography of adipose tissue-derived MDV-treated mice at baseline and days 7, 14, and 28 (**d**). Ejection fraction and fractional shortening were quantified (**e**) (mean ± s.d., *n* = 5 mice per group, repeated two-way ANOVA with Holm-Sidak’s multiple-comparisons test). **f, g** Representative Masson and Sirius Red staining images of MDV-treated mouse hearts at day 28. Fibrosis percentage was quantified (mean±s.d., *n* = 5 mice per group, one-way ANOVA with Bonferroni correction). Scale bar: 2 mm. **h** Western blots of mouse BAT whole tissue lysate, purified mitochondria, MDVs, and EV. The experiment was independently repeated on 3 mice with similar results. **i** Western blots of sEV enriched in BAT and heart left ventricle at steady state or under MI stress (*n* = 3 mice). **j** Schematic demonstrating the process of MDV packaging by EV machinery and the release of MDVs into the extracellular space. Created in BioRender. Shi, T. (2026) https://BioRender.com/op965jz. Source data are provided as a Source Data file.

MDVs were isolated from mouse iBAT and epididymal WAT (eWAT) using established protocols^13,16^ (Fig. 3a). Negative-staining transmission electron microscopy (TEM) confirmed membrane-bound vesicles of approximately 100-200 nm in diameter (Fig. 3a). Quantitative analysis showed that BAT produced substantially more MDVs than WAT, and cold exposure (4 °C, 14 days) significantly increased MDV yield from both depots, with BAT remaining the predominant source (Fig. 3b). Immunoblotting demonstrated that MDVs were enriched in mitochondrial membrane proteins, including TOMM20 and UCP1 on outer membrane of mitochondria (OMM), as well as multiple electron transport chain (OXPHOS) components on inner membrane of mitochondria (IMM) (Fig. 3c).

To assess whether MDVs contribute to BAT transplantation-related cardioprotection, MDVs were isolated from BAT or WAT and administered intravenously to mice subjected to LAD ligation. Vesicle doses were normalized by total protein content (30 µg MDV protein per mouse, quantified by BCA assay). Compared with vehicle-treated MI mice, both BAT-MDV and WAT-MDV-treated animals showed better preservation of cardiac functions, attenuation of adverse left ventricular remodeling, and smaller fibrosis area (Fig. 3d–g). No significant differences were detected between BAT- and WAT-MDV groups, indicating that adipose-derived MDVs, irrespective of depot origin, are capable of mitigating post-infarction structural remodeling.

Notably, whole-tissue WAT transplantation did not confer detectable cardioprotection in contrast to BAT transplantation (Fig. 1c–f), whereas isolated WAT-MDVs were cardioprotective when administered in equal amounts. Several factors may account for this divergence. First, WAT contains substantially fewer MDVs than BAT (approximately 5-10-fold lower MDV yield, Fig. 3b), making endogenous WAT-derived MDV flux to the heart relatively inefficient, whereas direct MDV injection circumvents this limitation by equalizing vesicle dose. Second, WAT releases a complex secretome that includes non-coding RNAs, fatty acids, and proinflammatory adipokines, which are known to promote systemic inflammation, adverse cardiac remodeling, and heart failure^10,17^. These detrimental signals are likely to outweigh any modest benefit from the small amount of WAT-derived MDVs after WAT transplantation, whereas purified WAT-MDVs administered in isolation can reveal the intrinsic cardioprotective potential of MDVs as a vesicle class.

Given that MDVs can be released into the extracellular space and recovered within EV preparations under stress conditions^18,19^, we next asked whether BAT-derived MDVs are present in BAT- and heart-derived EVs. To this end, we isolated MDVs from BAT homogenates and small EVs from BAT explant-conditioned medium by sequential differential centrifugation and compared their protein composition (Supplementary Fig. 5a, b). As expected, BAT-MDVs were strongly enriched for mitochondrial proteins, including TOMM20, cytochrome c, and representative OXPHOS subunits, and displayed a protein profile distinct from BAT mitochondria. By contrast, BAT-sEVs showed the expression of canonical EV markers (CD63, TSG101) and contained detectable OXPHOS components (Fig. 3h), indicating that a subset of sEVs carries a mitochondrial protein signature reminiscent of intracellular MDVs.

We then examined how MI regulates this mitochondrial signature in tissue-derived sEVs. When equal amounts of sEV protein were analyzed, OXPHOS levels were increased in BAT-sEVs but reduced in left ventricular (LV)-derived sEVs after MI (Fig. 3i). These changes are consistent with a model in which BAT enhances the release of OXPHOS-enriched sEVs into the circulation, whereas the injured heart may act as a sink that preferentially internalizes such vesicles in the post-MI setting (Fig. 3j).

### Heart DAMPs engage BECN1-VPS35 in BAT MDV biogenesis

**Fig. 4:**
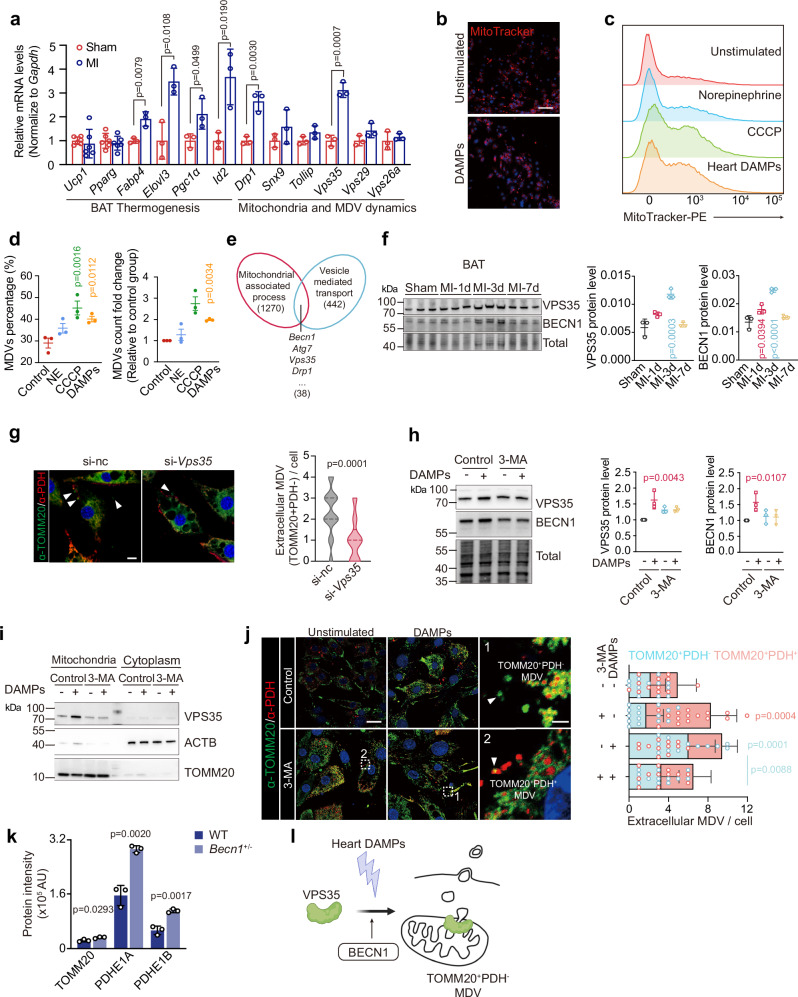
Heart DAMPs engage a BECN1-VPS35-dependent signaling in BAT to drive TOMM20⁺PDH⁻ MDV biogenesis and release. **a** Relative mRNA expression of genes associated with BAT thermogenesis and mitochondria dynamics in BAT from sham-operated and MI mice (mean ± s.d., *n* = 6 mice (*Ucp1* and *Pparg*), 3 mice (other genes) per group, two-tailed unpaired Student’s *t* test). **b** Representative images of differentiated brown adipocytes stained by Mitotracker (500 nM) and treated with heart DAMPs (20 μg/mL). Scale bar: 100 μm. Data are representative of 3 independent experiments with similar results. **c** Flow cytometric histogram of Mitotracker-PE^+^ MDVs release of NE or CCCP or heart DAMPs-treated brown adipocytes. **d** Quantitative results of MitoTracker^+^ vesicles percentage and counts (mean ± s.e.m., *n* = 3 independent experiments, paired one-way ANOVA with Bonferroni correction). **e** Venn diagram showing genes associated with vesicle-mediated transport pathways and mitochondrial-associated processes. The overlapping region (*n* = 38, list in the Supplementary Data 1) highlights genes in both pathways. **f** Time course of VPS35 and BECN1 protein expression in BAT after MI (mean ± s.d., *n* = 3 mice per group, one-way ANOVA with Bonferroni correction). **g** Representative confocal images of brown adipocytes transfected with si-nc or si-*Vps35*. Quantification of extracellular TOMM20⁺PDH⁻ MDVs per cell (Median and quartiles, *n* = 25, two-tailed unpaired Student’s *t* test). Scale bar: 10 μm. **h** Representative western blots of brown adipocytes incubated with 3-MA (10 mM) and stimulated with DAMPs. Quantification of VPS35 and BECN1 protein relative levels (mean ± s.d., *n* = 3 independent experiments, two-tailed paired Student’s *t* test). **i** Representative mitochondria and cytoplasm western blots of brown adipocytes incubated with 3-MA and stimulated with heart DAMPs (*n* = 3 independent experiments). **j** Representative immunofluorescence images of brown adipocytes stimulated with heart-derived DAMPs with or without 3-MA. Extracellular MDV puncta per cell were quantified (mean ± s.e.m., *n* = 15 (3-MA/Unstimulated), 12 (other groups) cells, unpaired one-way ANOVA with Bonferroni correction). Scale bar: 20 μm; 2 μm (insets). **k** Quantification of TOMM20, PDHE1A, and PDHE1B protein levels in MDVs isolated from BAT of WT and *Becn1*^+/–^ mice by proteomics (mean ± s.d., *n* = 3 mice per group, two-tailed unpaired Student’s *t* test). **l** Schematic showing VPS35 recruitment to mitochondria by *Becn1* under MI stress, leading to TOMM20^+^PDH^-^ MDV formation. Created in BioRender. Shi, T. (2026) https://BioRender.com/u1707w5. Source data are provided as a Source Data file.

We first asked whether myocardial injury engages MDV biogenesis in BAT. Myocardial infarction increased BAT expression of thermogenic genes (*Ucp1, Pparg, Fabp4, Elovl3, Pgc1a* and *Id2*) together with genes involved in mitochondrial and vesicular dynamics^11,16,20–24^ (*Drp1, Snx9, Tollip, Vps35, Vps29* and *Vps26a*) (Fig. 4a). Mitophagy-related genes did not significantly change in BAT of MI mice (Supplementary Fig. 6a). Using a flow cytometry-based assay with defined vesicle gating and MitoTracker staining, we established that heart damage-associated molecular patterns (DAMPs), similar to norepinephrine and CCCP, induced mature brown adipocyte (differentiated from C3H10T1/2 cell^25^) mitochondrial remodeling and increased the proportion and number of BAT-derived MDVs in culture supernatants (Fig. 4b–d and Supplementary Fig. 6b–f).

To identify regulators linking mitochondrial quality control to vesicle trafficking in this context, we intersected genes annotated to mitochondrial-associated process and vesicle-mediated transport, which highlighted *Becn1, Atg7, Vps35*, and *Drp1* (Fig. 4e). Among these, BECN1 and VPS35 proteins were progressively increased in BAT during the first week after MI (Fig. 4f). VPS35, as a member of the retromer complex, can be recruited to mitochondria, mediating mitochondria blebbing and MDV formation^15,16,21,24^. In differentiated brown adipocytes, siRNA-mediated knockdown of *Vps35* reduced extracellular TOMM20⁺PDH⁻ MDV puncta per cell (Fig. 4g), indicating that VPS35 is required for efficient MDV release. *Becn1* is notable as it participates not only in autophagosome formation but also in endosomal trafficking via *Vps35*^26,27^. Pharmacological inhibition of class III PI3K with 3-methyladenine (3-MA) decreased BECN1 and VPS35 expression in brown adipocytes (Fig. 4h). Subcellular fractionation showed that DAMPs increased BECN1-dependent modulation of VPS35 mitochondrial recruitment, and this redistribution was diminished by 3-MA (Fig. 4i).

We next examined how this module affects MDV release and cargo selection. Quantification of extracellular TOMM20⁺PDH⁻ and TOMM20⁺PDH⁺ MDVs released from differentiated brown adipocytes treated with heart-derived DAMPs showed that 3-MA selectively reduced the DAMP-induced TOMM20⁺PDH⁻ subset (Fig. 4j). Under basal (unstimulated) conditions, MDV release was low and only modestly affected by 3-MA, whereas heart-derived DAMPs robustly increased TOMM20⁺PDH⁻ MDVs in control cells but not in 3-MA-treated cells. Proteomic analysis of MDV fractions isolated from BAT of wild-type and *Becn1*^*+/–*^ mice showed reduced abundances of the mitochondrial proteins TOMM20, PDHE1A and PDHE1B in MDVs from *Becn1*^*+/–*^ MDVs (Fig. 4k). Together with the schematic summary (Fig. 4l), these data define a BAT signaling module in which myocardial injury-derived DAMPs are associated with induction of a BECN1-VPS35-linked mitochondrial quality-control pathway and enhanced production of TOMM20⁺PDH⁻ MDVs.

### *Becn1*-deficiency ablates BAT cardioprotection

**Fig. 5:**
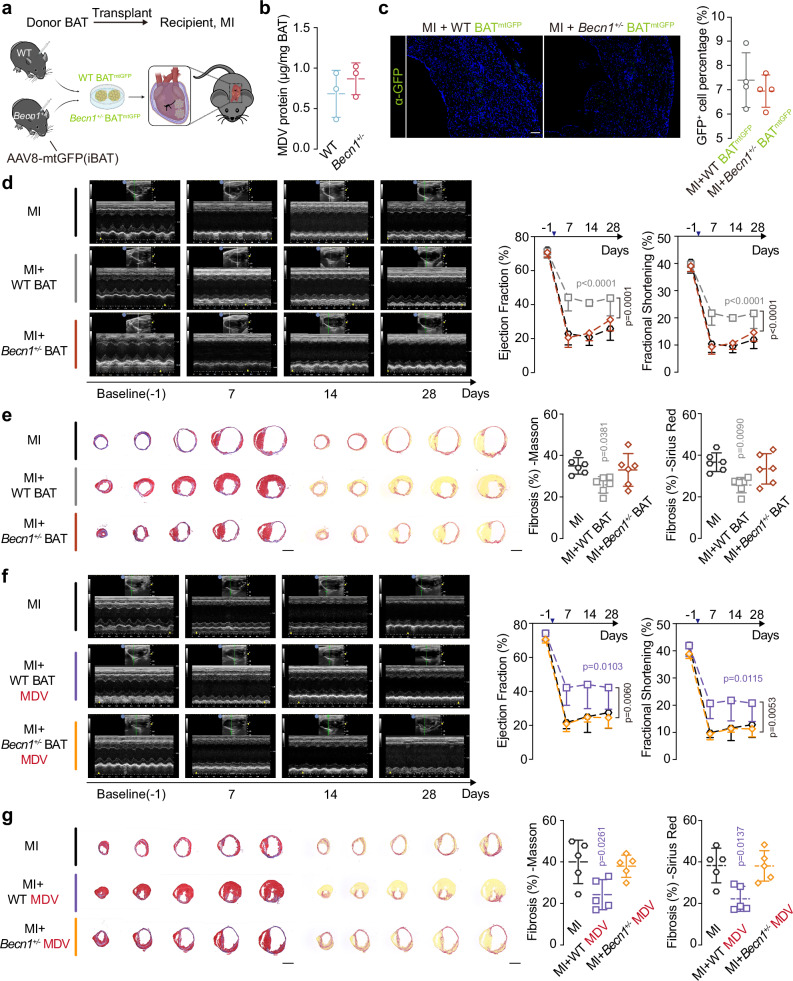
*Becn1*-knockdown ablates MDV-mediated cardioprotection of BAT-derived MDVs. **a** Schematic illustration of the BAT transplant from *Becn1*^*+/–*^ or WT mice (mitochondria labeled) to WT MI mice. Created in BioRender. Shi, T. (2026) https://BioRender.com/wadta8v. **b** Protein quantification of MDVs isolated from WT or *Becn1*^+/–^ BAT, normalized by BAT tissue weight (mean ± s.d., *n* = 3 mice per group, two-tailed unpaired Student’s *t* test). **c** Representative confocal images of BAT^mtGFP^-receiving mice at MI 4 d, stained for GFP (green) and nuclei (DAPI, blue). Scale bar: 100 μm. Mt-EGFP^+^ cell percentage was quantified (mean ± s.d., *n* = 4 mice per group, two-tailed unpaired Student’s *t* test). **d** Representative B-mode and M-mode echocardiography of WT or *Becn1*^+/–^ BAT-receiving mice at baseline and days 7, 14, and 28. Ejection fraction and fractional shortening were quantified (mean ± s.d., *n* = 6 mice per group, repeated two-way ANOVA with Holm-Sidak’s multiple-comparisons test). **e** Representative Masson and Sirius Red staining images of WT or *Becn1*^+/–^ BAT-receiving mouse hearts at day 28. Fibrosis percentage was quantified (mean ± s.d., *n* = 6 mice per group, one-way ANOVA with Bonferroni correction). Scale bar: 2 mm. **f** Representative B-mode and M-mode echocardiography of WT or *Becn1*^+/–^ MDV-treated mice at baseline and days 7, 14, and 28. Ejection fraction and fractional shortening were quantified (mean ± s.d., *n* = 5 mice per group, repeated two-way ANOVA with Holm-Sidak’s multiple-comparisons test). **g** Representative Masson and Sirius Red staining images of WT or *Becn1*^+/–^ MDV-treated mouse hearts at day 28. Fibrosis percentage was quantified (mean ± s.d., *n* = 5 mice per group, one-way ANOVA with Bonferroni correction). Scale bar: 2 mm. Source data are provided as a Source Data file.

To determine the effects of BECN1-defficient BAT and its MDVs, we generated donor BAT expressing a mitochondrial GFP reporter (AAV8-mtGFP) in wild-type or *Becn1*^*+/–*^ mice and transplanted equal amounts of BAT to the pericardial space of MI recipients (Fig. 5a). Donor BAT from wild-type and *Becn1*^*+/–*^ mice released similar amounts of MDVs, and the proportion of GFP⁺ cells in recipient hearts after transplantation was comparable between the two donor genotypes, indicating similar overall vesicle production and delivery (Fig. 5b, c).

Echocardiography revealed that transplantation of wild-type BAT improved left ventricular ejection fraction and fractional shortening during the 4 weeks after MI and reduced infarct fibrosis, whereas BAT from *Becn1*^*+/–*^ donors conferred no improvement despite comparable engraftment (Fig. 5d, e). To directly test the role of BAT-derived MDVs, we isolated MDVs from wild-type or *Becn1*^*+/–*^ BAT and administered them to MI mice. Delivery of wild-type BAT-MDVs recapitulated the functional and structural benefits of BAT transplantation, whereas MDVs from *Becn1*^*+/–*^ BAT showed attenuated effects on both parameters (Fig. 5f, g). These data indicate that BECN1 in donor BAT is dispensable for bulk MDV yield and delivery but is required for the cardioprotective efficacy of BAT-derived MDVs in vivo.

### BAT MDVs deliver bioenergetic cargo to reprogram macrophages

**Fig. 6:**
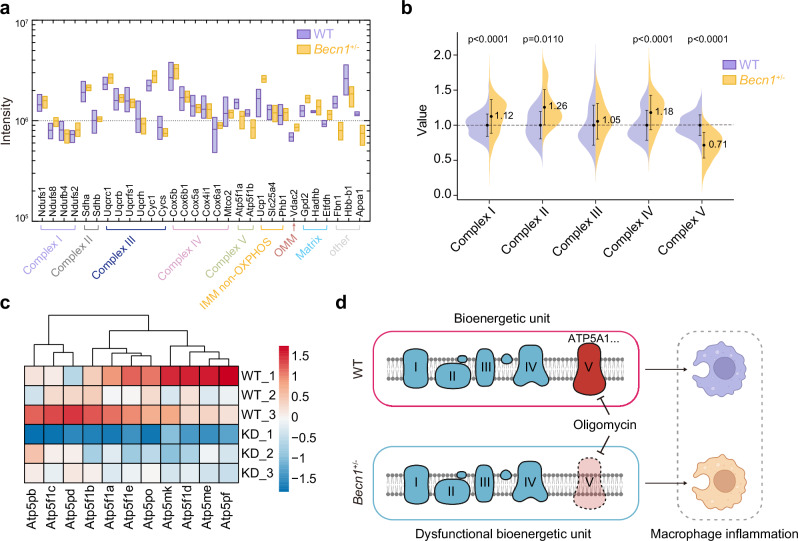
*Becn1* deficiency alters the respiratory-chain cargo of BAT-derived MDVs. **a** Quantitative proteomic analysis of MDVs from WT and *Becn1*^+/–^ BAT. Proteomics analysis of WT and *Becn1*^+/–^ BAT MDV protein cargos. The top 30 abundant proteins were calculated by the average intensity of all samples (median + least to max, *n* = 3 mice per group). **b** Comparative analysis of protein levels of electron-transport-chain Complex I-V key subunits (protein with intensity>20000 was recognized as complex key subunit) in MDVs from WT and *Becn1*^+/–^ BAT (mean ± s.d., *n* = 39 subunits for Complex I, 4 proteins for Complex II, 9 subunits for Complex III, 21 subunits for Complex IV, and 11 subunits for Complex V, two-tailed unpaired Student’s *t* test). **c** Heatmap of Complex V key subunits in MDVs from WT and *Becn1*^+/–^ BAT. **d** Schematic representation of respiratory-chain composition in BAT-derived MDVs from WT versus *Becn1*^+/–^ mice, summarizing differences in the relative contribution of complexes I-V inferred from the proteomic analysis. Created in BioRender. Shi, T. (2026) https://BioRender.com/djhc1u2. Source data are provided as a Source Data file.

To examine how *Becn1* deficiency alters the respiratory-chain cargo of BAT-derived MDVs, quantitative proteomics of MDVs from WT and *Becn1*^*+/–*^ BAT identified multiple subunits of complexes I-V together with inner and outer mitochondrial membrane proteins (Fig. 6a). When these proteins were grouped by respiratory complex, *Becn1*^*+/–*^ MDVs showed a marked reduction for complex V compared with WT MDVs (Fig. 6b and Supplementary Fig. 8a, b). *Becn1*-knockdown impaired mitochondrial respiratory chain expression^28^ (Supplementary Fig. 7) and the packaging of these proteins into MDV (Supplementary Fig. 8c).

To dissect which ATP synthase components were altered, we focused on ATP5 family members in the MDV proteome. Clustering of ATP5 subunit abundances separated WT and *Becn1*^*+/–*^ MDVs into distinct groups and revealed a coordinated reduction of multiple ATP5F1/F₀ subunits in the *Becn1*^*+/–*^ samples (Fig. 6c). Together with the complex-level analysis, these data indicate that a dysfunctional bioenergetic unit in which ATP synthase subunits are selectively reduced from *Becn1*^*+/–*^ BAT-derived MDVs (Fig. 6d).

**Fig. 7:**
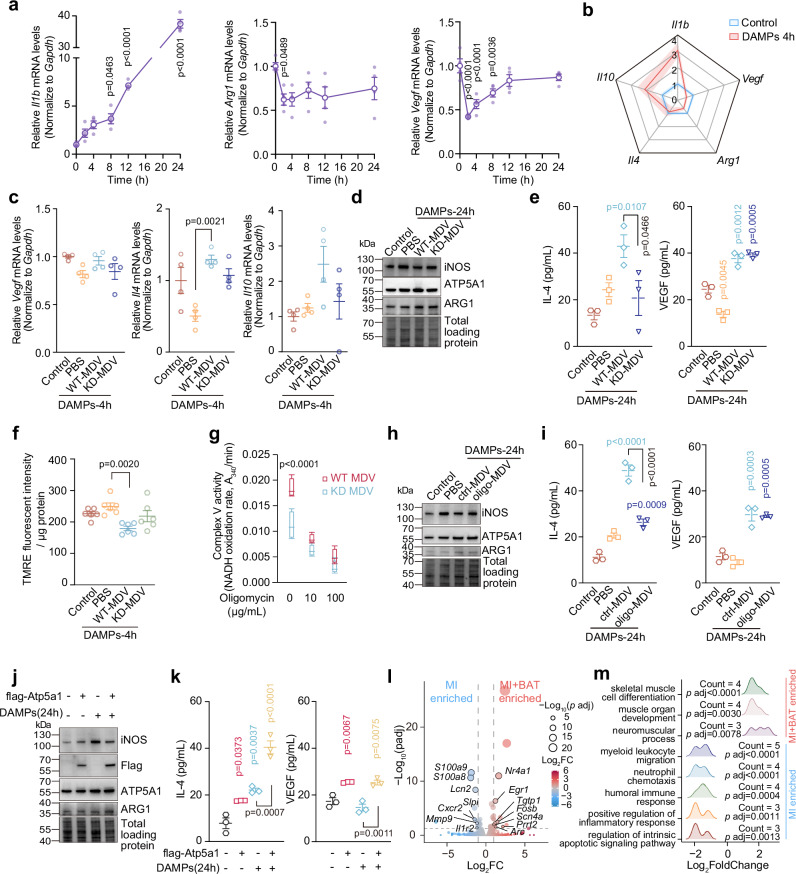
Complex V-dependent effects of BAT MDVs on macrophage polarization. **a** RT-qPCR analysis of inflammatory and reparative cytokine genes in iBMDMs during DAMPs priming (mean ± s.e.m., *n* = 4 independent experiments, one-way ANOVA with Bonferroni correction). **b** Radar plot showing relative expression of inflammation-related genes in iBMDMs. **c** RT-qPCR analysis of reparative cytokine genes in iBMDM co-incubated with WT or *Becn1*^+/–^ BAT MDVs (mean ± s.e.m., *n* = 4 independent experiments, one-way ANOVA with Bonferroni correction). **d** Representative western blots of macrophages treated with DAMPs for 24 h in the presence of PBS, WT-MDVs, or *Becn1*^+/–^-MDVs (*n* = 3 independent experiments). **e** IL-4 and VEGF concentrations in supernatants from macrophages treated as in (**d**) (mean±s.e.m., *n* = 3 independent experiments, one-way ANOVA with Bonferroni correction). **f** Mitochondria membrane potential analysis of macrophages co-incubated with WT or *Becn1*^+/–^-MDVs. (mean ± s.e.m., 6 technical replicates, *n* = 3 independent experiments with similar results, one-way ANOVA with Bonferroni correction). **g** Purified WT-MDVs and *Becn1*^+/–^-MDVs were incubated with oligomycin. NADH oxidation rate (A_340_/min) was measured as a readout of complex V activity (median (center line), the 25th and 75th percentiles (box limits), and the min and max values (whiskers), *n* = 4 mice, two-way ANOVA with Holm-Sidak’s multiple-comparisons test). **h** Representative western blots of macrophages treated with DAMPs for 24 h with control MDVs, or oligomycin (100 μg/mL)-treated MDVs (*n* = 3 independent experiments). **i** IL-4 and VEGF concentrations in supernatants from macrophages treated as in (**h**) (mean ± s.e.m., *n* = 3 independent experiments, one-way ANOVA with Bonferroni correction). **j** Representative western blots of macrophages overexpressing flag-Atp5a1 and treated with DAMPs for 24 h (*n* = 4 independent experiments). **k** IL-4 and VEGF concentrations in supernatants from macrophages treated as in (**j**) (mean ± s.e.m., *n* = 3 independent experiments, one-way ANOVA with Bonferroni correction). **l** Volcano plot displaying differentially expressed genes (DEGs) in LV tissue from MI mice with BAT transplantation vs. MI control mice at MI 3 d (*n* = 3 mice per group, two-sided Wald test, with *p*-values adjusted for multiple comparisons using the Benjamini-Hochberg method, fold change>2, *p* < 0.05). **m** GO pathway enrichment analysis of LV tissue with BAT transplantation vs. MI control at MI 3 d (one-sided Fisher’s Exact Test, with p-values adjusted using the Benjamini-Hochberg method). Source data are provided as a Source Data file.

Next, how BAT-derived MDVs influence the response of cardiac macrophages to heart DAMPs was investigated. In myocardial injury, a timely transition from pro-inflammatory macrophages to reparative macrophages is necessary for cardiac repair. IL-4, IL-10, and ARG1 are biomarkers of reparative macrophage, reported to be beneficial for cardiac remodeling and wound healing^29,30^. Heart DAMPs were generated as previously described^31^. ScRNA-seq data for the temporal dynamics of macrophages in MI have been reported^32^. In vitro stimulation of cardiac macrophages with heart DAMPs induced a rapid increase in *Il1b* transcript activation and *Arg1*, *Vegf* downregulation (Fig. 7a, b), indicating that heart DAMPs polarized immortalized bone marrow-derived macrophage (iBMDM) cells toward a pro-inflammatory phenotype, similar to CCR2^+^ inflammatory macrophages (Supplementary Fig. 9).

Addition of BAT-derived MDVs carrying intact OXPHOS cargo (WT-MDVs) to DAMP-stimulated macrophages selectively increased *Il4* mRNA, whereas MDVs derived from *Becn1*^*+/–*^ BAT (KD-MDVs) with reduced ATP synthase content had no detectable effects (Fig. 7c). At the protein level, WT-MDVs increased ATP5A1 level and ARG1 levels, reduced iNOS, and enhanced secretion of IL-4, whereas KD-MDVs showed diminished capacity to modulate these markers (Fig. 7d, e). Consistent with a role for complex V in mitochondria membrane potential consumption, WT-MDVs decreased mitochondrial membrane potential in DAMP-treated macrophages, whereas KD-MDVs failed to do so (Fig. 7f).

Complex V activity in purified MDVs in the presence of increasing concentrations of oligomycin was tested. WT-MDVs exhibited higher NADH oxidation rates that were sensitive to oligomycin, whereas KD-MDVs displayed reduced complex V activity (Fig. 7g). Thus, we generated oligomycin-treated MDVs (oligo-MDVs) from BAT and compared their effects with vehicle-treated MDVs. In DAMP-stimulated macrophages, oligo-MDVs were less effective than control MDVs at decreasing iNOS and increasing ARG1 expression and elicited lower IL-4 and VEGF secretion (Fig. 7h, i).

To determine whether ATP5A1 alone in recipient cells is sufficient to promote a reparative program, we overexpressed flag-Atp5a1 by lentivirus vector in cardiac macrophages. Atp5a1 overexpression in the presence of DAMPs reduced iNOS, increased ARG1 and ATP5A1 protein, and augmented IL-4 and VEGF release (Fig. 7j, k).

Finally, RNA-seq of the left ventricle from MI and MI+BAT hearts showed that BAT transplantation upregulated genes related to myocardium repair and development, and downregulated genes related to innate immune cell recruitment (Fig. 7l, m).

Together with the in vitro data, these findings indicate that BAT-derived MDVs with intact complex V cargo enhance a pro-reparative phenotype in DAMP-activated macrophages, and that BAT transplantation in vivo is associated with a corresponding shift of macrophages toward tissue-remodeling transcriptional states.

### Macrophage complex V correlates with cardiac function

**Fig. 8:**
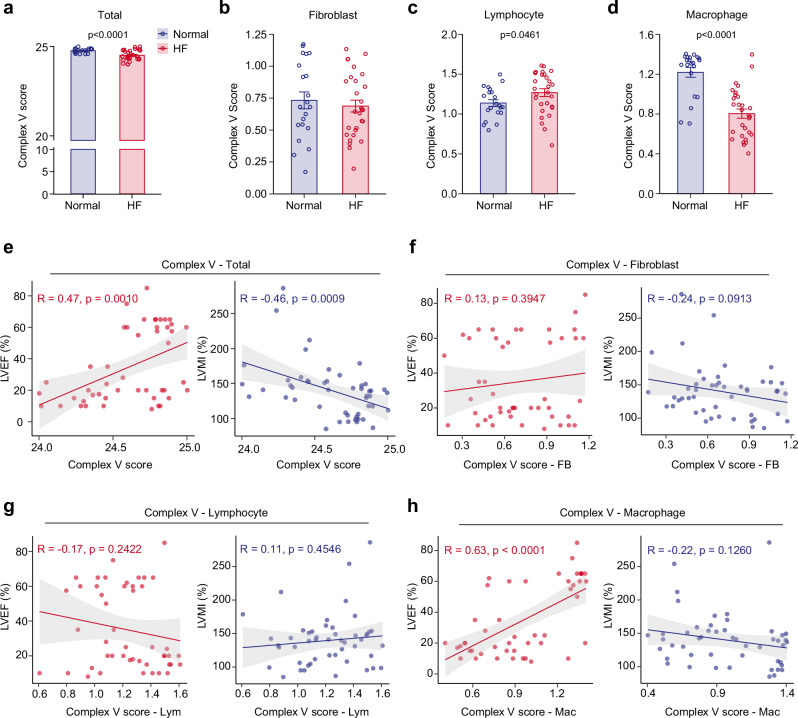
Macrophage complex V level is positively associated with cardiac functions of patients with HF. Complex V expression score in endomyocardial biopsies from normal control and patients with HF across different cell types (**a**), in fibroblasts (**b**), in lymphocytes (**c**), and in macrophages (**d**). The scores were compared (mean ± s.d., *n* = 21 (normal control), *n* = 29 (patients with HF), two-tailed unpaired Student’s *t* test). Correlation between cardiac functions (LVEF, left ventricular ejection fraction; LVMI, left ventricular mass index) and complex V expression score in all cell types (**e**), fibroblasts (**f**), lymphocytes (**g**), and macrophages (**h**) of normal control and patients with HF (Solid lines indicate linear regression curves, and shaded bands represent the 95% confidence intervals, *n* = 47 for LVEF, 49 for LVMI, two-sided Pearson correlation analysis). Source data are provided as a Source Data file.

The myocardial gene expression signatures of patients with HF were identified by Hahn et al^33^. The cell types and gene expression profiles were deconvoluted with BayesPrism as described^34^. Using the public transcriptional data from non-failing and heart-failure patients, we calculated a Complex V gene set score for each sample and major cardiac cell types. Complex V scores were significantly lower in macrophages but not in other cell types (Fig. 8a–d). Higher Complex V scores, particularly in macrophages, were positively associated with LVEF and inversely associated with LV mass index, whereas Complex V scores in fibroblasts or lymphocytes showed no significant relationship with these parameters (Fig. 8e–h).

**Fig. 9:**
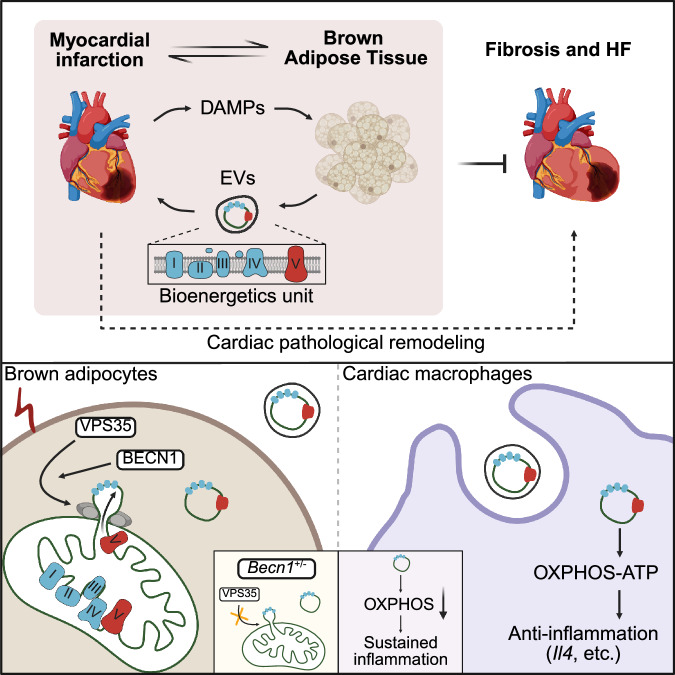
BAT-derived bioenergetic vesicles promote cardiac repair after MI. MI triggers heart-to-brown adipose tissue signaling that induces the production and release of MDVs enriched in mitochondrial respiratory chain proteins. Through *Becn1*-dependent recruitment of VPS35 to mitochondria, mitochondrial proteins are packaged and secreted via extracellular vesicle trafficking pathways, then delivered to cardiac macrophages. The bioenergetic cargo enhances oxidative phosphorylation and anti-inflammatory programming, thereby limiting fibrosis and improving cardiac repair. *Becn1* deficiency reduces complex V enrichment in these vesicles and abolishes cardioprotection. Created in BioRender. Shi, T. (2026) https://BioRender.com/7lxm9ok.

Taken together, BAT facilitates cross-organ repair by delivering complex V proteins to cardiac macrophages (Fig. 9). Cardiac macrophage-specific complex V levels serve as hallmarks of improved prognosis post-MI.

## Discussion

Our study elucidates a mechanism by which BAT mitigates cardiac pathological remodeling by delivering MDVs to cardiac macrophages. The MI-triggered mobilization of MDVs containing mitochondrial respiratory proteins, particularly ATP5 synthase, reprograms macrophage oxidative phosphorylation and promotes reparative cytokine production. The results suggest that BAT-heart bidirectional crosstalk mediated by bioenergetic vesicles contributes to cardiac repair.

Recent work has convincingly established that BAT is a systemic cardiometabolic modulator^3^. BAT is highly metabolically active, and its mitochondria-derived materials can traffic between cells^11,14,35^. Extending this framework, our data points to a more defined interorgan axis in the post-infarct context. BAT releases a mitochondria-derived, OXPHOS-enriched MDV population that appears to be preferentially captured by the cardiac macrophages. Functionally, MDV bioenergetic integrity (especially complex V) is required to couple myocardial uptake to macrophage immunometabolic reprogramming and tissue repair, establishing BAT-derived MDVs as a mechanistically tractable mediator of interorgan mitochondrial signaling in cardiac injury.

The heart-to-adipose signaling is also likely to be relevant. Cardiac injury generates DAMPs and activates the sympathetic system with norepinephrine release that can reshape adipose biogenesis, differentiation, and activation. Consistent with prior reports that MI can induce adipose neogenesis from epicardial progenitors near the infarct site and exacerbate fibrosis^36,37^, and that myocardial I/R injury can induce thermogenic gene programs such as *Ucp1* expression^7^. We find that non-reperfused MI elicits acute BAT atrophy with transcriptional activation of lipid metabolism pathways, while *Ucp1* expression remains largely unchanged. Differences in injury context and timing may explain these divergent thermogenic signatures and suggest that BAT’s response to cardiac injury is multifaceted and stage dependent.

The molecular pathways underlying MDV production have not been fully elucidated. MDVs display significant heterogeneity in their biogenesis mechanism, cargo composition, and fates. Mitochondria outer membrane TOMM20^+^ MDVs start from *Miro1/2* anchoring and *Drp1* scission, then are pulled along cell filaments and degraded by lysosomes^20^. Mitochondria matrix PDH^+^ mtDNA^+^ MDVs are produced by *Snx9* under the condition of mitochondrial metabolic dysfunction, and tend to activate cGAS-STING in the cytoplasm^22,38^. Mitochondria inner membrane MDVs are generated through the budding of the inner membrane via VDACs and are incised by ESCRT machinery^39^. VPS35 has been reported to control vesicle transport between the mitochondria and peroxisomes^24^. Here, we discovered that BAT ejects IMM-dominant MDVs, with their cargo composition being modulated by *Becn1* expression levels. We further propose that BECN1 influences selective cargo sorting, potentially through VPS35, thereby tuning the bioenergetic payload of released MDVs. Defining the upstream signals that couple MI-associated stress sensing to BECN1-dependent MDV biogenesis and cargo selection will be an important next step.

Our data are consistent with the view that the BAT-MDVs analyzed here represent a mitochondria-enriched subpopulation within the BAT-EV pool described by Rosina et al^13^. Whereas Rosina et al. characterized bulk BAT EVs carrying oxidatively damaged mitochondrial components (identified as PDH^+^) involved in local mitochondrial quality control, our MDV-enriched preparations display a proteome dominated by intact OXPHOS, including Complex V, and anti-inflammatory features in cardiac macrophages. The observation that total BAT-EV preparations also promote an anti-inflammatory and pro-reparative macrophage phenotype is compatible with the presence of such OXPHOS-rich MDVs within bulk EVs. Further single-vesicle and in vivo tracking studies will help delineate the composition and functional relationships of these BAT-derived vesicle populations.

At matched doses, isolated BAT- and WAT-derived MDVs showed comparable oxidative phosphorylation cargo and cardioprotective effects, indicating that the intrinsic bioenergetic activity of individual MDVs is not strictly depot-restricted. This, however, is distinct from the effect of whole-tissue transplantation in vivo, which integrates depot-specific differences in mitochondrial abundance, MDV output, and the broader secretory profile. BAT likely confers a stronger benefit in this setting because it contains more mitochondria, releases more MDVs under stress, and provides a more favorable secretory profile for metabolic homeostasis and cardiac repair than WAT.

Mechanistically, our findings position ATP synthase/Complex V as a required, but not exclusive, mediator of MDV-induced macrophage polarization toward a reparative state. The incomplete loss of protection with oligomycin-treated MDVs and the partial restoration of anti-inflammatory features by ATP5A1 overexpression indicate that additional MDV cargo, such as mtDNA, lipids, or mitochondrial ROS, is likely to cooperate with the respiratory chain/bioenergetic unit. Dissecting how these cargoes integrate to control macrophage metabolism and phenotype will be an important objective for future work.

In the human heart-failure cohorts, Complex V was evaluated at the transcript level, and protein-level in cardiac macrophages could not be assessed. Thus, these data represent transcriptomic associations rather than direct evidence of MDV transfer. Future studies will require protein-level validation and the development of new tools (tissue-specific MDV markers, vesicle-tracing strategies) to trace MDV origin and fate in patients.

While this study provides insights into the role of MDVs in intercellular communication and metabolic regulation, several questions remain unanswered. It is not yet clear whether OXPHOS proteins delivered by MDVs act as autonomous bioenergetic units or are incorporated into the host mitochondrial network. Moreover, the upstream pathways that couple stress sensing and mitochondrial quality-control signals to MDV biogenesis and cargo selection are only partially understood.

Also, given that BAT contains multiple non-adipocyte populations, a Ucp1-Cre-driven, brown adipocyte-specific *Becn1* knockout would provide a more definitive test of our model by restricting BECN1 loss to thermogenic adipocytes and minimizing confounding effects from stromal-vascular and immune cells.

Finally, BAT mass and activity decline with age, raising the possibility that impaired BAT-to-heart vesicular signaling could contribute to reduced repair capacity in older individuals. Although therapeutic translation remains speculative, strategies aimed at restoring BAT function, promoting WAT browning, or selectively enhancing beneficial MDV production may ultimately offer complementary avenues to limit adverse remodeling after ischemic injury.

Our findings may help inform future EV engineering by specifying both the functional cargo requirement (OXPHOS, particularly complex V) and the primary recipient cell type (cardiac macrophages), thereby providing a mechanistic design specification that could be a promising translational approach.

## Methods

### Animal studies

This study complies with all relevant ethical regulations. All animal procedures were approved by the Institutional Animal Care and Use Committee of Tongji University (TJ-HB-LAC-2024-16).

All wild-type C57BL/6J mice (4–12 weeks, male) were purchased from SPF (Beijing) Biotechnology Co., Ltd. *Becn1*^*+/−*^ and B6-G/R^fl/+^ mice (both on a C57BL/6J background) were purchased from GemPharmatech Co., Ltd. All mice were housed in a specific-pathogen-free facility at 22 °C and 4060% humidity, with a 12/12 h light/dark cycle and free access to distilled water and sterilized chow diet (Jiangsu Xietong Pharmaceutical Bio-Engineering Co., Ltd, XTI01JX-003). Sex was not considered a biological variable because all experiments were performed in male mice only.

Male mice aged 8–12 weeks were used as BAT donors and MI recipients, with age-matched animals across all treatment groups within each experiment. All animals were randomly assigned to groups using ear labeling and body-weight records. A single-blind method was applied to reduce subjective bias: analysis experiments and data assessments were conducted without awareness of the treatment allocation. Data were excluded only when mice died during surgery. Mouse tissue weight was measured after removal of peripheral connective tissue and drying.

*Becn1*^*+/−*^ mice were crossed with WT mice to generate *Becn1*^*+/−*^ offspring. Genomic DNA was amplified with forward primer (AACCTGAGTTTAACTCCCAGTGG) and reverse primer (TTGATGCTTTCCTCAGTAATCCTG). Genotypes were determined by PCR product size (WT: 1344 bp, KO: ~305 bp). When *Becn1*^*+/−*^ mice were used, their WT littermates were used as controls. B6-G/R^fl/+^ male mice were crossed with B6-G/R^fl/+^ female mice to generate B6-G/R^fl/+^ offspring. Genomic DNA was amplified with tdTomato-knock-in forward primer (TACGGCATGGACGAGCTGTAC) and reverse primer (CCAACCTTTGTTCATGGCAG). Genotypes were determined by PCR product size (WT: 0 bp, KI: 333 bp).

### Adipose tissue transplant preparation

Eight- to twelve-week-old C57BL/6J donor mice (wild-type, *Becn1*^*+/−*^ and B6-G/R^fl/+^ etc., male) were anesthetized with 2.0% isoflurane. Dorsal skin was incised to harvest interscapular BAT. Peripheral white adipose tissue was carefully removed, and interscapular BAT (iBAT) was dissected and washed in cold sterile PBS. BAT was cut into small pieces ~2 mm in diameter using a sterile scalpel. Prepared BAT implants were weighed and kept in cold PBS until use. Epididymal white adipose tissue (WAT) was harvested from the same donor, avoiding inclusion of the epididymis, and processed identically.

Cold-exposed donor mice were housed at 4 °C for 14 days (12/12 h light/dark cycle, free access to water and chow) before adipose tissue harvest.

### LAD ligation and BAT transplantation

MI was induced by permanent ligation of the LAD coronary artery. Ten- to twelve-week-old C57BL/6J male mice were randomly assigned to each group. Mice were anesthetized with 2.0% isoflurane inhalation, and anesthesia was maintained by 2.0% isoflurane via endotracheal intubation with 20-gauge tubes. Oxygen flow (0.4 L/min) was maintained during the procedure using an animal ventilator. All operations were conducted on a 37 °C heating pad to maintain body temperature.

After anesthesia, a small thoracotomy was performed between the second and third ribs to expose the LAD. Without rupturing the pericardium, the LAD was ligated 1-2 mm below the left atrial appendage with 7-0 nylon suture. In BAT transplantation groups, prepared BAT implants were inserted into the pericardial cavity through small pericardial perforations made by the ligation needle. Up to four implants (total weight 10-15 mg) were placed in the recipient pericardial cavity. LAD occlusion was confirmed by ST-segment elevation on the electrocardiogram. After chest closure, mice recovered on a heating pad. For pain management, meloxicam (4 mg/kg) was administered subcutaneously for 2 days. Sham mice underwent identical procedures except for LAD ligation.

Revascularization of transplanted BAT was evaluated by laser speckle imaging. At day 3 after transplantation, recipient mice were anesthetized with 2.0% isoflurane and placed on a heating pad. The pericardial cavity was reopened, and the transplanted BAT was selected as the region of interest (ROI) on the laser speckle blood flow imaging system (RWD, RFLSI ZW). Images and videos were acquired to measure average blood perfusion within the ROI.

### In situ AAV-mediated mitoGFP expression in mouse BAT

Mitochondria-tracing vectors based on the COX VIIIa presequence have been widely used to study mitochondrial transfer in vivo^40,41^. To generate BAT mitochondria-tracing mice, in situ injection of AAV8-mitoGFP (AAV8-CMV-COX VIIIa presequence-eGFP) into BAT was performed. Four- to five-week-old male mice were anesthetized with 2.0% isoflurane. AAV vectors (1 × 10^12^ GC/mL in normal saline) were loaded into a microsyringe (Hamilton, 26 G). After dorsal skin incision, interscapular BAT was exposed and injected with 1 × 10^10^ GC (10 μL) AAV per site at 4-5 sites distributed throughout BAT, avoiding visible vessels. Four weeks after AAV injection, vector expression was confirmed by fluorescence imaging. The AAV vectors are available from the corresponding author upon request.

### Echocardiography

Cardiac function at baseline and after MI was assessed by transthoracic echocardiography. Mice were depilated one day before imaging. On the day of examination, animals were anesthetized with 2.0% isoflurane in oxygen and placed in a supine position on a temperature-controlled imaging platform to maintain body temperature at ~37 °C. Four limb ECG electrodes were attached to monitor heart rate throughout the procedure. Echocardiography was performed using a SiliconWave30 system (KOLO Medical) equipped with a 30 MHz probe (L38-22K3). Pre-warmed ultrasound gel was applied to the chest, and imaging depth, focus, and gain were adjusted to optimize endocardial border definition.

For functional assessment, a parasternal long-axis view of the left ventricle was first obtained in B-mode. The M-mode cursor was then positioned perpendicular to the ventricular septum and posterior wall at the mid-papillary muscle level, and M-mode assessments were recorded. Heart rate during acquisition was maintained between 400 and 600 beats per minute to avoid under- or over-anesthesia.

### Masson trichrome and Sirius Red staining

The fibrotic area of the infarcted mouse heart was assessed by Masson trichrome staining in combination with Sirius Red staining on adjacent sections. Hearts were serially cryosectioned (thickness=10 μm) from the apex toward the ligation level (typically 5-6 evenly spaced section groups per heart). Sections were fixed in 4% paraformaldehyde (PFA), washed with tap water and treated with Bouin’s solution at 60 °C for 1 h, then rinsed under running water until colorless. Slides were stained with Weigert’s iron hematoxylin for 15 min to label nuclei, washed, and then incubated with Biebrich scarlet-acid fuchsin for 15 min. After thorough washing, slides were differentiated in phosphomolybdic-phosphotungstic acid until fibrotic areas became unstained, followed by aniline blue staining for 5–7 min to visualize collagen fibers. Slides were rinsed, differentiated in 1% acetic acid, dehydrated through graded alcohols, cleared in xylene, and mounted with neutral balsam.

For each Masson-stained section, an adjacent section within the same section group was subjected to Sirius Red staining to visualize collagen deposition. PFA-fixed sections were washed, dried, and incubated in picrosirius red solution at room temperature for 30 min. The stained slides were rinsed in water, dehydrated, cleared, and mounted with neutral balsam. Images were captured using a Leica M80 with an IC80 HD camera.

Fibrotic regions appeared blue on Masson-stained sections and Sirius Red-positive on adjacent sections. Fibrosis was quantified independently for each staining method using identical anatomical levels and image-analysis parameters. For each section, the fibrotic area was defined as the positively stained region and presented as a percentage of total myocardial cross-sectional area. For each mouse, fibrosis (%) was calculated as the average across 5-6 serial sections spanning from the apex to the ligation level.

### Immunohistofluorescent (IHF) imaging

MDV transfer was evaluated by tissue fluorescence and immunohistofluorescence imaging. Hearts from mice receiving fluorescent-labelled BAT (BAT^ZsGreen^, BAT^mtGFP^) transplantation were embedded in OCT and cryosectioned (5 μm for fluorescence imaging, 30 μm for 3D reconstruction).

For imaging with endogenous fluorescence, all procedures were performed in a protected environment from light. Sections were fixed in 4% PFA for 10 min and washed thoroughly with PBS. After blocking with 1% BSA, sections were incubated with primary antibodies overnight at 4 °C. Primary antibodies included: anti-TOMM20 (1:50, HUABIO, ET1609-25), anti-PDH (1:1000, Abcam, ab110333), anti-sarcomeric α-actinin (1:500, Abcam, ab137346), anti-CD68 (1:100, Bio-Rad, MCA1957), and anti-MPO (15 μg/mL, R&D Systems, AF3667-SP). On day 2, slides were washed three times and incubated with appropriate secondary antibodies at room temperature for 1 h, followed by additional washes. Samples were mounted with DAPI-containing mounting medium and imaged using a fluorescence or confocal microscope (Keyence BZ-X800 and Nikon A1R). Secondary-only controls underwent identical procedures without primary antibody incubation. Images were acquired using laser power and exposure times not exceeding those used for secondary-only controls.

For conventional immunohistofluorescence on cardiac sections, slides were fixed in 4% PFA for 2 h, then rinsed in PBS, immersed in 10 mM sodium citrate buffer (pH 6.0) and heated to 95-100 °C in a microwave oven for 10 min. After gradual cooling to room temperature in sodium citrate buffer, slides were washed in PBS and permeabilized with 0.05% Triton X-100 in PBS for 10 min and blocked for 1 h at room temperature in blocking solution (1% BSA). Slides were then incubated with primary antibodies (GFP Polyclonal Antibody, Invitrogen, A-11122) diluted in blocking solution overnight at 4 °C, washed three times in PBS and incubated with appropriate secondary antibodies at room temperature for 1 h, followed by additional washes. Slides were incubated with autofluorescence-quenching reagent (TrueBlack Plus Lipofuscin Autofluorescence Quencher, Biotium, #23014-T) for 10 min, washed in PBS three times and mounted with DAPI-containing mounting medium.

### Proximity ligation assay (PLA) in situ detection

Proximity ligation assays were performed on cardiac cryosections using the NaveniBright-MR, AP kit (Navinci Diagnostics) according to the manufacturer’s instructions. BAT^mtGFP^-receiving mouse hearts were cryoprotected (thickness=5 μm). Sections went through regular fixation (4% PFA for 1 h), antigen retrieval, and permeabilization. Then, the slides were incubated with endogenous alkaline phosphatase (AP) inhibitor (Beyotime, P0100C), washed with 1XTBST, and blocked with the kit blocking solution for 1 h at room temperature. For PLA, sections were incubated overnight at 4 °C with two GFP primary antibodies raised in different species (GFP Polyclonal Antibody, Invitrogen, A-11122; GFP Monoclonal Antibody, Invitrogen, MA5-15256). After three washes in buffer, sections were incubated with Navenibody for 60 min at 37 °C, followed by two amplification reactions. Slides were then incubated with AP and developed with the substrate. PLA-positive signals appeared as pink puncta by AP-mediated reaction and were imaged using the bright-field microscope with identical acquisition settings across groups.

### Transmission electron microscopy (TEM)

For cardiac TEM, hearts from mice that had received BAT^mtGFP^ transplantation were harvested under 2.0% isoflurane anesthesia. The transplanted BAT was removed, and the infarct area (pale region) was dissected and immediately immersed in cold 2.5% glutaraldehyde. After fixation, tissue was cut into ~1 mm pieces gently to minimize mechanical damage and stored in 2.5% glutaraldehyde at 4 °C. Samples were washed in 0.1 M phosphate buffer (PB), dehydrated through graded ethanol (30-100%), and infiltrated with 33%, 50%, and 67% resin in ethanol. After embedding in capsules, samples were polymerized under UV for 48 h at -20 °C. Polymerized blocks were ultrathin-sectioned using a Leica UC7 ultramicrotome with a diamond knife (Daitome) and immunogold-labelled with anti-GFP (1:50, HUABIO, ET1607-31).

### Mitochondria and MDVs preparation

Mitochondria and MDVs from mouse adipose tissue were isolated according to the manufacturer’s protocols (Thermo Scientific, 89874) and previous studies^12–14^. BAT or WAT from eight- to twelve-week-old mice was homogenized in 800 μL isolation reagent A (containing 4 mg/mL BSA), followed by 800 μL reagent C. Unbroken cells and nuclei were removed by centrifugation at 700 × *g* for 10 min. Purified mitochondria were pelleted at 3000 × *g* for 15 min, washed with wash buffer, and resuspended in cold PBS. The post-mitochondrial supernatant was centrifuged at 9000 × *g* to pellet and discard other organelles. At this stage, the supernatant contained membrane-enveloped MDVs. The supernatant was incubated with 0.05% trypsin (EDTA-free) on ice for 10 min to digest unprotected proteins, followed by ultracentrifugation at 100,000 × *g* for 1 h (Beckman Coulter Optima XPN-100) to pellet MDVs. The MDV pellet was washed twice with PBS and resuspended in PBS, normal saline, or RIPA buffer according to downstream applications. All centrifugation steps were performed at 4 °C.

For in vivo and in vitro MDV treatment, all reagents were sterilized, and MDVs were resuspended in normal saline or PBS. Aliquots were sonicated for protein quantification using a BCA assay kit (Thermo Scientific, 23225). For all in vivo and in vitro experiments, MDV preparations isolated from adipose tissue of different donor mice were quantified by BCA assay and adjusted to the same total protein concentration, so that MVDs were dose-matched and administered at an equivalent dose per mouse via tail-vein injection or for cell culture. MI surgery was performed immediately after MDV administration.

For negative staining of isolated MDVs, purified MDVs were diluted and applied to grids. Grids were stained with 2% phosphotungstic acid for 5 min, excess stain was removed, and samples were air-dried before TEM imaging.

### Brown adipocyte culture and differentiation

For brown adipocyte induction, 48 h after C3H10T1/2 cells (National collection of authenticated cell cultures, GNM19) reached 100% confluency, brown adipogenic induction media containing 1 μg/mL insulin (MCE, HY-P1156), 1 nM T3 (MCE, HY-A0070A), 1 μM dexamethasone (MCE, HY-14648), 1 μM rosiglitazone (MCE, HY-17386), 125 μM indomethacin (MCE, HY-14397), and 0.5 mM 3-Isobutyl-1-methylxanthine (IBMX) (Sigma-Aldrich, I5879) was used for at least 48 h until cell shape became oval and cytoplasm turned bright. Maintenance media containing 1 μg/mL insulin and 1 nM T3 was then used for 4-6 days, refreshed every 2 days. Mature brown adipocytes exhibited typical multilocular lipid droplets that can be stained by oil red. Oil red staining was performed following the manufacturer’s instructions (Beyotime, C0158). Fixed cells were washed with the wash buffer for 20 s, stained with oil red for 20 min, and again washed with the wash buffer for 30 s. Live cells and oil red-stained cells were imaged with Leica DMi1 inverted microscope.

### siRNA knockdown in brown adipocytes

siRNA transfection was performed using a liposome-based method. C3H10T1/2 cells were first induced to differentiation, and siRNA transfection was carried out at the mid-to-late stage of differentiation. The culture medium was replaced with antibiotic-free DMEM 24 h before transfection. For transfection, siRNAs targeting *Vps35* (gene ID: 65114) and the negative control siRNA (si-NC) were separately diluted in Opti-MEM serum-free medium. Lipofectamine 3000 was diluted in Opti-MEM according to the manufacturer’s instructions and incubated for 5 min, after which it was gently mixed with the diluted siRNA and incubated at room temperature for 15 min to form siRNA-lipid complexes. The complexes were then added dropwise to the cells to achieve a final siRNA concentration of 50 nM. Cells were gently mixed and incubated at 37 °C in 5% CO_2_. After 8 h, the medium was replaced with fresh maintenance medium, and cells were cultured for an additional 72 h. At 72 h after transfection, proteins were collected for western blot validation or cells were fixed for subsequent experiments.

The si-*Vps35* (sense: CGUGUGGACUACGUCGAUAAAdTdT; antisense: UUUAUCGACGUAGUCCACACGdTdT) and si-NC were synthesized by Hippo Biotech.

### Heart DAMPs

Heart DAMPs were cardiomyocytes’ cellular contents released via freeze-thaw damage^31,32^. Eight to ten-week-old male mice were sacrificed for left ventricle acquisition. LV was cut, minced, and dissociated in PBS (5.8 μL/mg tissue, supplemented with protease inhibitor) by rapid freezing in liquid nitrogen and slow thawing on ice. The freeze-thaw cycle was repeated three times. After thawing, the LV lysate was vortexed and centrifuged at 20,000 × *g* to obtain the DAMPs solution (supernatant). DAMPs were stored at -80 °C. The final working concentration was 20 μg/mL.

### Flow cytometric analysis of mitochondria and MDVs

For flow cytometric analysis of MDVs, mitochondria were stained before treatment. After treatment, the released mitochondrial components in the supernatant were tested by flow cytometry. MitoTracker Orange (Invitrogen, M7510) pre-stained mature brown adipocytes were stimulated by 1 μM NE (MCE, HY-13715) or 10 μM CCCP (MCE, HY-100941) or heart 20 μg/mL DAMPs. Four hours later, culture media were collected for MDV enrichment. A series of centrifuges at 300 × *g* (10 min), 1000 × *g* (10 min), 2000 g (10 min), and 10,000 × *g* (30 min) was performed to remove dead cells and cell debris. For vesicle analysis, the flow cytometric detection threshold was set to 500 for FFC and SSC on the FACSAria III cell analyzer (BD). PE-MitoTracker fluorescent intensity of the enriched MDVs sample and 10,000 counting beads (BioLegend, 424902) were acquired and recorded on the flow cytometer. MDV amount was quantified by counting beads.

Cells were incubated with 3-MA or Tat-beclin for 30 min before stimulation by DAMPs and co-existed with DAMPs for 4 h before MDV analysis.

### Macrophage co-culture with MDVs

MDVs were isolated, quantified, and diluted to a final concentration of 30 µg/mL (protein concentration by BCA). The PBS or conditional media containing 30 µg/mL MDVs were added to the iBMDM cells. After 2 h of co-incubation, heart DAMPs were added to the media to activate the inflammatory response in iBMDM cells. The supernatant and iBMDM cells were harvested for downstream analyses, including qPCR (4 h), TMRE mitochondrial membrane potential assay (4 h), western blots (24 h), and ELISA assay (24 h).

### Lentiviral overexpression of ATP5A1 in iBMDMs

A lentiviral vector for overexpression of mouse Atp5f1a (encoding ATP5A1; Gene ID: 11946, NM_007505.2) was generated by cloning the coding sequence into the pGLV-CMV-MCS-EF1-coGFP-Puro vector, with an independent GFP reporter and a puromycin resistance cassette. A 3×FLAG tag was added to the C terminus for protein detection. The insert was amplified using Q5 Hot Start High-Fidelity 2× Master Mix (NEB), gel-purified, and ligated into EcoRI/BamHI-digested vector using T4 ligase. The ligation product was transformed into DH5α competent cells, and positive clones were identified by Sanger sequencing. Plasmids were purified using a plasmid miniprep kit (Qiagen).For lentiviral packaging, 293 T cells at approximately 90% confluence were transfected with the expression plasmid together with psPAX2 and pMD2G at a ratio of 4:3:1 using Lipofectamine 3000 in Opti-MEM, with a total DNA amount of 15 μg. Viral supernatants were collected at 48 h and 72 h after transfection and stored at -80 °C. For infection, iBMDMs at approximately 50% confluence were exposed to lentiviral supernatant for 6 h and then cultured in fresh medium. Cells were harvested 48 h after infection, and overexpression efficiency was assessed by GFP fluorescence and western blot. The lentiviral vectors are available from the corresponding author upon request.

### TMRE mitochondrial membrane potential detection

Mitochondrial membrane potential was determined by TMRE assay (abcam, ab113852) following the manufacturer’s protocols. Particularly, the iBMDM cells were washed twice, stained with 800 nM TMRE for 20 min, and washed three times. The TMRE fluorescent intensity was measured by a microplate reader (Infinite 200 Pro, Tecan) with an excitation wavelength (Ex) of 545 nm and an emission wavelength (Em) of 549 nm.

### Quantitative PCR

Total RNA of mouse tissue samples or cell pellets was extracted using the QIAzol (Qiagen, 79306) lysis method. BAT tissue was homogenized and dissolved completely in 1 mL cold QIAzol. 200 μL chloroform extracted RNAs by vortexing and incubation on ice for 10 min. Supernatant from 12,000 rpm centrifuge for 20 min was carefully collected by an ultra-fine insulin syringe (BD, 6 mm x 31 G) to avoid disturbing the upper lipid layer or the bottom layer. An identical volume of isopropanol was added and mixed. Total RNAs were pelleted at 12,000 rpm centrifuge for 10 min, then washed with 75% alcohol twice. Dried RNAs were dissolved in DEPC-treated water. A cDNA library was established by reverse transcription (SparkJade, AG305) with 1 μg RNA. Quantitative real-time PCR by 7500 qPCR system (Applied Biosystems, 435117) employed cDNA templates (diluted 6 to 10-fold), SYBR Green qPCR mix (Applied Biosystem, A57155), primers, and ddH_2_O. Relative gene expression level was calculated by △△CT method. The sequences of qPCR primers are listed in Supplementary Table 1.

### Western blot

BAT from sham or MI mice were weighed (W) and put into tubes with 6×W volume RIPA buffer (CST, #9806, diluted into 1× with distilled water). Protease inhibitor (Roche, 11836170001) and phosphatase inhibitor (Thermo Scientific, 78428) were added to RIPA buffer before tissue lysis. Tissue in RIPA buffer was homogenized at 1500 rpm for 2 min by tissue homogenizer (Benchmark Scientific, IPD9600) with pre-cooled grinding blocks. Tissue lysate was centrifuged for 15 min at 4 °C. To remove the upper-layer lipid, aspirate a clear protein solution in the middle layer with an ultra-fine insulin syringe (BD, 6 mm x 31 G) without disturbing the lipid layer. This step was repeated until the lipid was completely removed from the protein sample. Culture brown adipocyte whole cell lysate was also prepared by RIPA buffer lysis, centrifuging, and insulin syringe aspiration.

Mitochondria isolation using the mammalian cell mitochondria isolation kit (Thermo Scientific, 89874) was conducted following the manufacturer’s protocols. 2 × 10^7^ brown adipocytes were pelleted at 850 g for 2 min. Cells were added with 800 μL reagent A, then immediately vortexed for 5 seconds. After exactly 2 min incubation on ice, cells were added 10 μL reagent B, then vortexed every minute during 5 min incubation on ice. Finally, the sample was added 800 μL reagent C, gently mixed, and centrifuged at 700 × *g* to remove cell debris and nucleus. Obtaining purified mitochondria from the supernatant required a 3000 × *g* centrifuge. Purified mitochondria were lysed with RIPA buffer and stored at −80 °C. Post-mitochondria supernatant was further centrifuged at 12,000 × *g* to pellet other organelles, and the final supernatant was pure cytoplasm.

For western blot analysis, the protein sample was denatured with the sample buffer at 95 °C for 10 min. Proteins were separated in 10% or 12.5% SDS-PAGE gels and transferred to a PVDF membrane. Total loading protein stain was performed following the manufacture’s protocols (Invitrogen, A44449).

One hour block with 5% BAS was followed by primary antibody incubation overnight at 4 °C. Primary and secondary antibodies are listed in the Supplementary Data 2. Washed with PBST for 5 min × 3 times, the membrane was incubated with secondary antibody for 1 h at RT. Another PBST wash three times was performed before visualization with chemiluminescent substrates. The relative protein level was determined by the grayscale value of the target protein/reference protein. Fold change was calculated by the ratio of relative protein level in the experimental group/control group (Image Lab, v6.0.1). Uncropped and unprocessed scans of the most important blots are provided in the Source Data file.

### ELISA

Concentrations of IL-4 and VEGF in cell culture supernatants were measured by ELISA. After the indicated treatments, culture media were collected, centrifuged at 2000 × *g* for 10 min at 4 °C to remove cells and debris, and the supernatants were stored at -80 °C until use. Mouse IL-4 and VEGF were quantified according to the manufacturer’s instructions (Quantikine ELISA Mouse IL-4 Immunoassay, R&D Systems, M4000B-1; Mouse VEGF ELISA kit, QuantiCyto, EMC103). Briefly, standards and diluted samples were added in duplicate to antibody-coated 96-well plates and incubated at room temperature for the recommended period, followed by incubation with biotinylated detection antibody, streptavidin-HRP, and TMB substrate. The reaction was stopped with stop buffer, and absorbance was read at 450 nm with a reference wavelength of 540 nm using the microplate reader. Cytokine concentrations were calculated from standard curves.

### RNA-seq

The left ventricles were collected from mice that had undergone MI modeling, either with or without BAT transplantation. Total RNA was extracted using the TRIzol (Invitrogen, 15596018) method according to the manufacturer’s protocol. RNA purity and concentration were evaluated by the NanoDrop 2000 spectrophotometer (Thermo Scientific). RNA quality was assessed using the Agilent 2100 Bioanalyzer (Agilent Technologies). Libraries were constructed using the VAHTS Universal V6 RNA-seq Library Prep Kit according to the manufacturer’s instructions. The libraries were sequenced by OE Biotech Co., Ltd. (Shanghai, China) on an Illumina NovaSeq 6000 platform, and 150 bp paired-end reads were generated.

Raw reads files were processed using fastp1. The low-quality reads were removed, and clean reads were mapped to the reference genome NCBI_GRCm39 using HISAT2. FPKM and read counts of each gene were calculated by HTSeq-count. PCA analysis was performed using R (v3.2.0). Differential expression analysis was performed using DESeq2. The threshold *p*-value < 0.05 and fold-change>2 or <0.5 was set to determine significantly differentially expressed genes (DEGs). GO pathway analysis was performed using R (v3.2.0).

### Quantitative proteomics

A total of *n* = 3 biologically independent MDV preparations per group were analyzed, each derived from an independent mouse. BAT-derived MDVs were purified from WT and *Becn1*^*+/–*^ mice and lysed in RIPA buffer. Protein samples were precipitated by adding 1 volume of pre-chilled acetone followed by 4 volumes of pre-chilled acetone and incubation at -20 °C for 2 h. The precipitates were washed 2-3 times with pre-chilled acetone, resuspended in 200 mM tetraethylammonium bromide (TEAB), and ultrasonically dispersed. Trypsin was added at a 1:50 (w/w) trypsin-to-protein ratio for overnight digestion. Samples were then treated with 5 mM dithiothreitol for 30 min at 56 °C and with 11 mM iodoacetamide for 15 min at room temperature in the dark. Peptides were desalted using a Strata X SPE column. No TMT labeling, offline HPLC fractionation, or affinity enrichment was performed.

Tryptic peptides were dissolved in solvent A and loaded onto a home-made reversed-phase analytical column (25 cm length, 100 μm inner diameter). The mobile phases consisted of solvent A (0.1% formic acid and 2% acetonitrile in water) and solvent B (0.1% formic acid in acetonitrile). Peptides were separated on an Easy-nLC1000 UHPLC system at a constant flow rate of 500 nl/min using the following gradient: 6-24% solvent B over 0-14 min, 24-35% solvent B over 14-16 min, 35-90% solvent B over 16-18 min, and 90% solvent B over 18-20 min. Eluted peptides were introduced via a capillary source into a timsTOF Pro mass spectrometer and analyzed in data-independent parallel accumulation serial fragmentation (dia-PASEF) mode. The electrospray voltage was 1.75 kV. The full scan range was set to m/z 300-1,500, 20 PASEF MS/MS scans were acquired per cycle, the MS/MS scan range was m/z 400-850, and the isolation window was 7 m/z. LC-MS/MS analysis was performed by Jingjie PTM BioLab Co., Ltd. (Hangzhou, China).

DIA data were processed using DIA-NN (v1.8). Tandem mass spectra were searched against the Mus musculus protein database Mus_musculus_10090_SP_20231220.fasta (17,191 entries) concatenated with a reverse decoy database. Trypsin/P was specified as the cleavage enzyme with up to one missed cleavage allowed. N-terminal Met excision and carbamidomethylation of Cys were specified as fixed modifications. The false discovery rate was controlled at <1%. Relative protein abundance was calculated as fold change relative to the mean protein intensity of the control group. Mitochondrial respiratory chain complex scores were calculated as the mean relative abundance of all detected subunits, or of selected key subunits with protein intensity >20,000, within each complex.

### Clinical correlation

The endomyocardial biopsies gene expression data and clinical traits of healthy and heart failure with reduced ejection fraction (HFrEF) patients used for correlation analysis were obtained from the Zenodo data repository (doi: 10.5281/zenodo.4114616)^33^. The dataset included 50 left ventricular septal endomyocardial biopsies, comprising 21 donor controls and 29 patients with HFrEF. The 50 cases were included in our re-analysis. Clinical characteristics of control donors and patients with HFrEF are listed in Supplementary Table 2. The prognostic parameters LVEF were available in 47 cases, while LVMI was available in 49 cases. Deconvolution of the bulk RNA-seq data was performed to obtain information on the gene expression levels in each cell type using BayesPrism^34^. The annotated reference datasets human MI snRNA-seq^42^ at cellxgene (https://cellxgene.cziscience.com/collections/8191c283-0816-424b-9b61-c3e1d6258a77) were used as prior information in the deconvolution. All cell types identified through deconvolution included fibroblast, cardiomyocyte, endothelial, myeloid, neuronal, pericyte, mast, lymphoid, cycling cell, vSMC, and Adipocyte.

The expression matrix of Myeloid, Lymphoid, and Cardiomyocyte inferred by BayesPrism was extracted to compute the complex V genes score based on ssGSEA. For each sample, a complex V score value was assigned to each of the three cell types, and the correlation with clinical traits was estimated using Pearson correlation analysis. Human complex V genes include: ENSG00000124172, ATP5F1E, ATP5E; ENSG00000169020, ATP5ME, ATP5K; ENSG00000099624, ATP5F1D, ATP5D; ENSG00000154723, ATP5PF, ATP5J; ENSG00000167863, ATP5H, ATP5PD; ENSG00000228253, ATP8; ENSG00000152234, ATP5F1A, ATP5A1; ENSG00000241837, ATP5PO, ATP5O; ENSG00000110955, ATP5F1B, ATP5B; ENSG00000116459, ATP5PB; ENSG00000167283, ATP5MG, ATP5L; ENSG00000165629, ATP5F1C, ATP5C1; ENSG00000130770, ATPIF1; ENSG00000123472, ATPAF1; ENSG00000159199, ATP5MC1, ATP5G1; ENSG00000135390, ATP5MC2, ATP5G2; ENSG00000154518, ATP5MC3, ATP5G3; ENSG00000198899, ATP6; ENSG00000171953, ATPAF2; ENSG00000156411, ATP68, ATP5MJ; ENSG00000150756, ATPSCKMT; ENSG00000125375, ATP5S; ENSG00000173915, ATP5MK, UMSG5). For each sample, each of the three cell types was attributed a complex V score value to estimate relevance in conjunction with clinical traits using Pearson correlation analysis.

### Statistics and reproducibility

No statistical method was used to predetermine sample size. Data were excluded only when the mice died during the surgery. All animals, cells, and samples were randomly assigned to each treatment group. For animal experiments, the investigators were blinded to allocation during outcome assessment.

Data analysis was performed in GraphPad Prism (v.9.5). Statistical analyses were selected based on data distribution and study design. Normality was assessed using the Shapiro-Wilk test. Two-group comparisons were performed using a two-sided unpaired Student’s *t* test for normally distributed data with equal variance or a two-sided Mann-Whitney U test otherwise. Comparisons among more than two groups were performed using one-way ANOVA with Bonferroni’s multiple-comparisons test for normally distributed data or the Kruskal-Wallis test with Dunn’s multiple-comparisons test otherwise. Repeated measurements across multiple groups were analyzed using two-way repeated-measures ANOVA with Holm-Sidak’s multiple-comparisons test. For paired biological replicate data normalized to the matched control, paired Student’s *t* test or paired one-way ANOVA with Bonferroni correction was used.

*P* values in all figures were presented both in the figure and in the source data file. *P* < 0.05 was recognized as a significant difference. The statistical method used was specified in the figure legends of each panel.

### Reporting summary

Further information on research design is available in the Nature Portfolio Reporting Summary linked to this article.

## Supplementary information

Supplementary Information

Description of Additional Supplementary Files

Supplementary Data 1

Supplementary Data 2

Reporting Summary

Transparent Peer Review file

## Source data

Source data

## Data Availability

The infarct left ventricular RNA-seq data generated in this study, including raw and processed files, have been deposited in the Gene Expression Omnibus (GEO) under accession code GSE291141. The mass spectrometry proteomics data generated in this study have been deposited in the ProteomeXchange Consortium via the iProX partner repository^43,44^ under dataset identifier PXD061744 and under project ID IPX0011275000 in the iProX repository. Previously published data re-analyzed in this study are publicly available. The single-cell RNA-seq data of MI mouse cardiac leukocytes^32^ are available under GSE163129. RNA-seq data of WT or adipocyte-specific *Becn1* KO mice BAT^28^ are available under GSE148275. RNA-seq data of human myocardial samples^33^ are available on Zenodo Data Repository [https://zenodo.org/records/4114617]. Source data are provided with this paper.
