# Supplementary material for: Mitochondria-derived vesicles with bioenergetic units from brown adipose tissue attenuate cardiac remodeling post-myocardial infarction
Source: Nat Commun. 2026 May 21;17:6690. doi: 10.1038/s41467-026-73388-3 (PMC13385750; doi:10.1038/s41467-026-73388-3)

Supplementary Information

Supplementary Figure 1-9

Supplementary Table 1-2

Full blots in Supplementary Figures

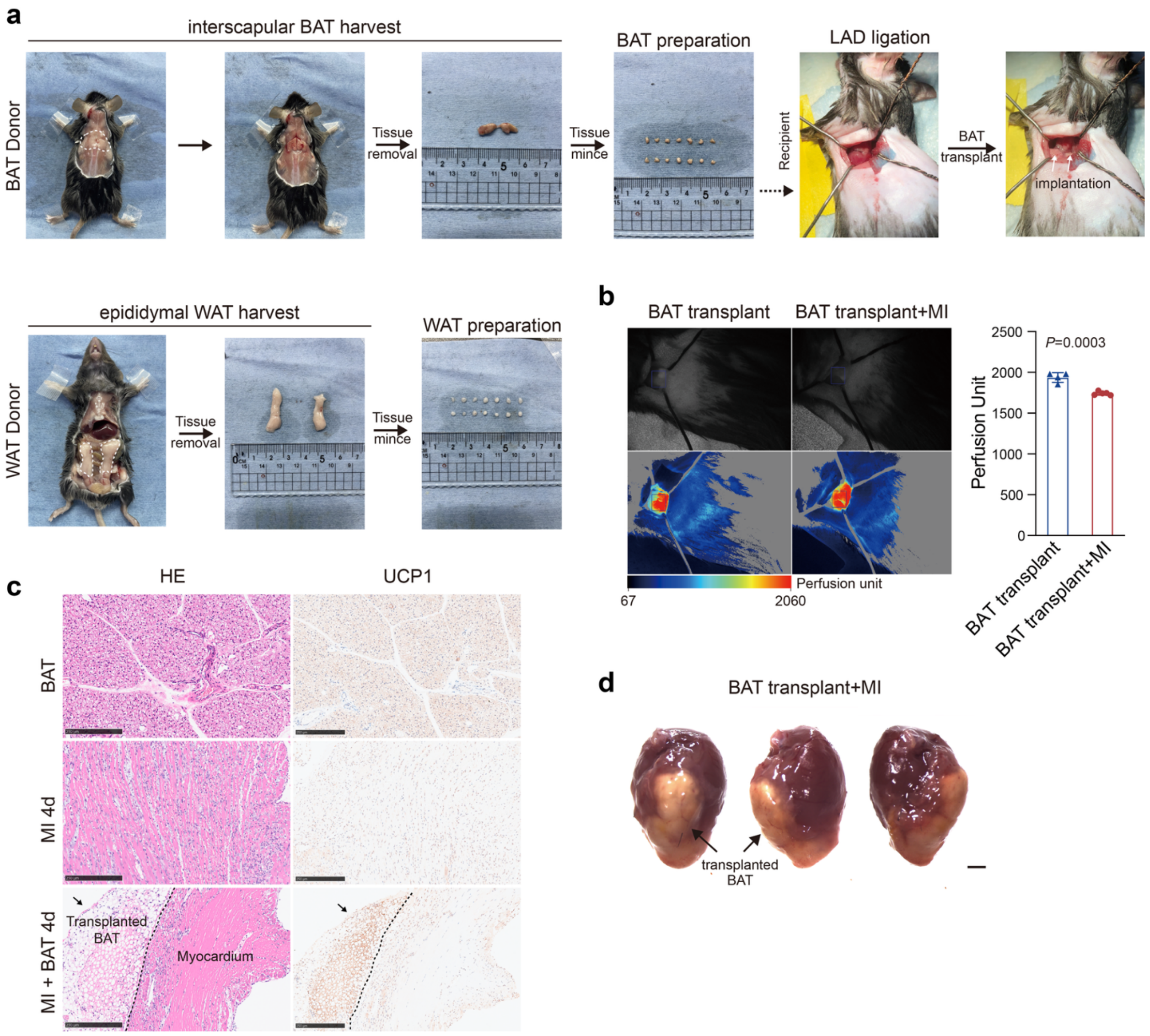

Supplementary Fig. 1 Establishment of pericardial BAT transplant on MI mice.

a, Graphic procedures of BAT or WAT pericardial transplant and MI modeling. BAT was harvested at the interscapular area of the donor mouse and was minced to make small BAT implants with a diameter of approximately 2 mm. Meanwhile, the recipient mouse was subjected to LAD ligation without pericardium rupture. BAT implants were imbedded between the pericardium and myocardium. Epididymal WAT was harvested and prepared as described above.

b, Representative images of laser speckle contrast assay of mice received BAT transplantation with or without LAD ligation at surgery 3d. Perfusion of transplanted BAT

was quantified (mean±s.d., n=4 mice for BAT transplant group, 5 for BAT transplant+MI group, two-tailed unpaired Student's t-test).

c, Representative images of HE and UCP1 immunohistochemistry of normal BAT and transplanted BAT at surgery 3d. Scale bar: 250 μm.

d, Representative images of MI mouse heart with BAT transplant at surgery 7d. Scale bar: 1 mm.

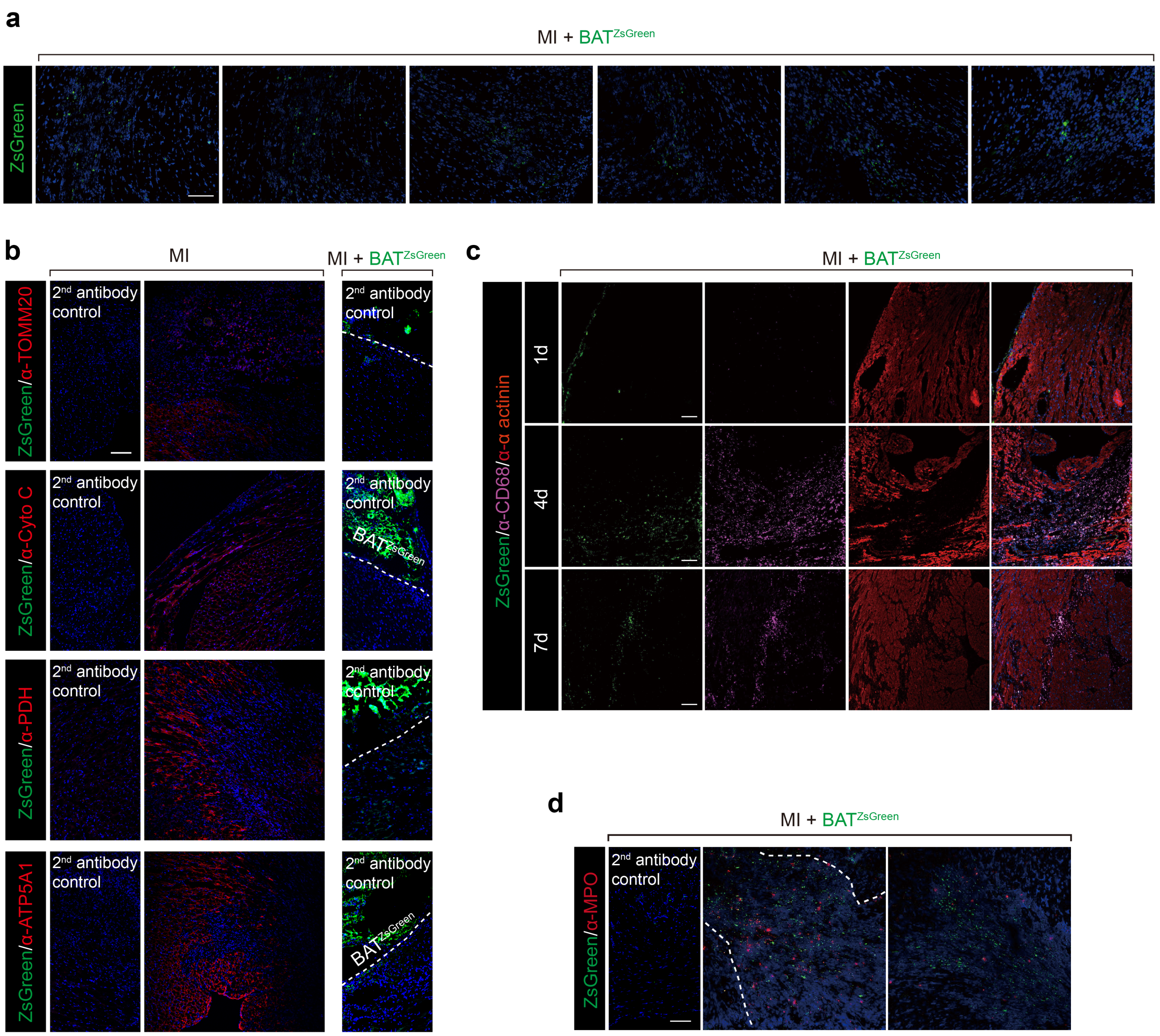

Supplementary Fig. 2 BATosomes are BAT-derived mitochondria-derived vesicles in the infarcted myocardium.

a, Representative confocal images of left ventricles 4 days after MI in BAT^{ZsGreen}-receiving mice. Scale bar: 100 μm.

b, Biological controls (MI hearts without BAT transplantation) and secondary antibody-only staining controls for BAT^{ZsGreen}-receiving mouse heart imaging, **related to Fig. 2c**. Scale bar: 100 μm.

c, Time course of BAT-derived MDVs accumulation and macrophage infiltration in BAT^{ZsGreen}-receiving mice at day 1, 4, and 7 after MI. Sections were stained for sarcomeric

α-actinin (cardiomyocytes, red), CD68 (macrophages, magenta), and nuclei (DAPI, blue). Scale bar: 100 μm.

d, Representative confocal images of the left ventricles in $BAT^{ZsGreen}$-receiving mice at MI 4d, stained for MPO (neutrophil, red) and nuclei (DAPI, blue). Scale bar: 100 μm.

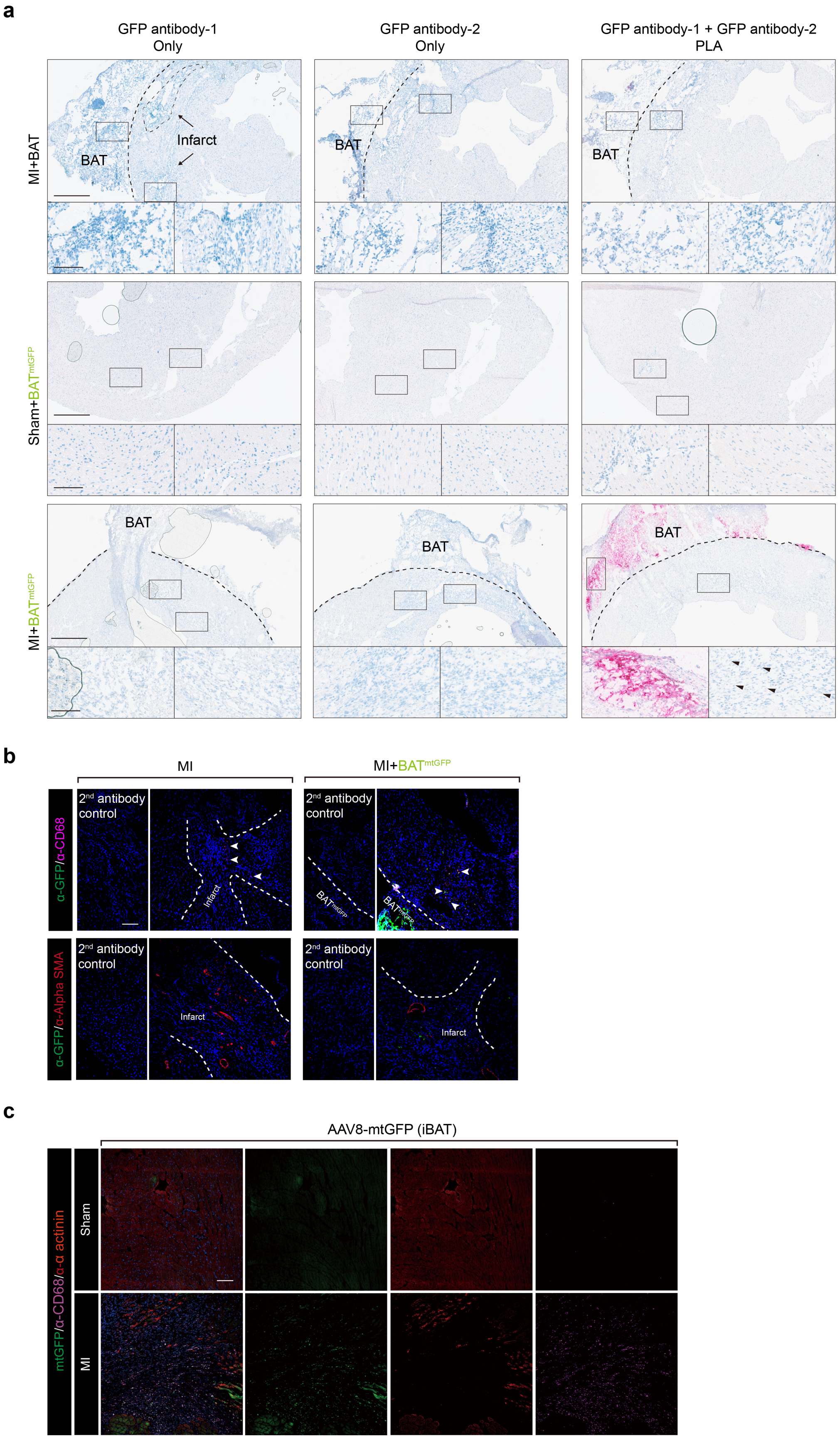
a
GFP antibody-1
Only
GFP antibody-2
Only
GFP antibody-1 + GFP antibody-2
PLA
MI+BAT
Sham+BATmtGFP
MI+BATmtGFP
BAT
Infarct
b
MI
MI+BATmtGFP
α-GFP/α-CD68
α-GFP/α-Alpha SMA
2nd antibody control
Infarct
BATmtGFP
c
AAV8-mtGFP (iBAT)
mtGFP/α-CD68/α-α actinin
Sham
MI

39

Supplementary Fig. 3 MDVs transfer to cardiac macrophages.

a, Proximity ligation assay (PLA) for GFP in hearts from MI mice receiving unlabeled BAT (MI + BAT), sham-operated mice receiving BAT^{mtGFP} (Sham+ BAT^{mtGFP}) and MI mice receiving BAT^{mtGFP} (MI+ BAT^{mtGFP}). Sections were incubated with GFP antibody-1 alone, GFP antibody-2 alone, or with both GFP antibodies, followed by PLA detection. Scale bar: 500 μm; 50 μm (magnification).

b, Immunofluorescence validation of mtGFP signal in infarcted hearts of BAT^{mtGFP}-receiving mice. Sections were stained for GFP (green) with CD68 (macrophages) or α-SMA (fibroblasts) (red). Scale bar: 100 μm.

c, Tracing endogenous BAT-derived MDVs using AAV8-mtGFP injected in situ into interscapular BAT. Hearts from sham and MI mice were analyzed 4 days after surgery and stained for CD68 (magenta), sarcomeric α-actinin (red), and DAPI (blue). Scale bar: 100 μm. The experiment was independently repeated 3 times with similar results.

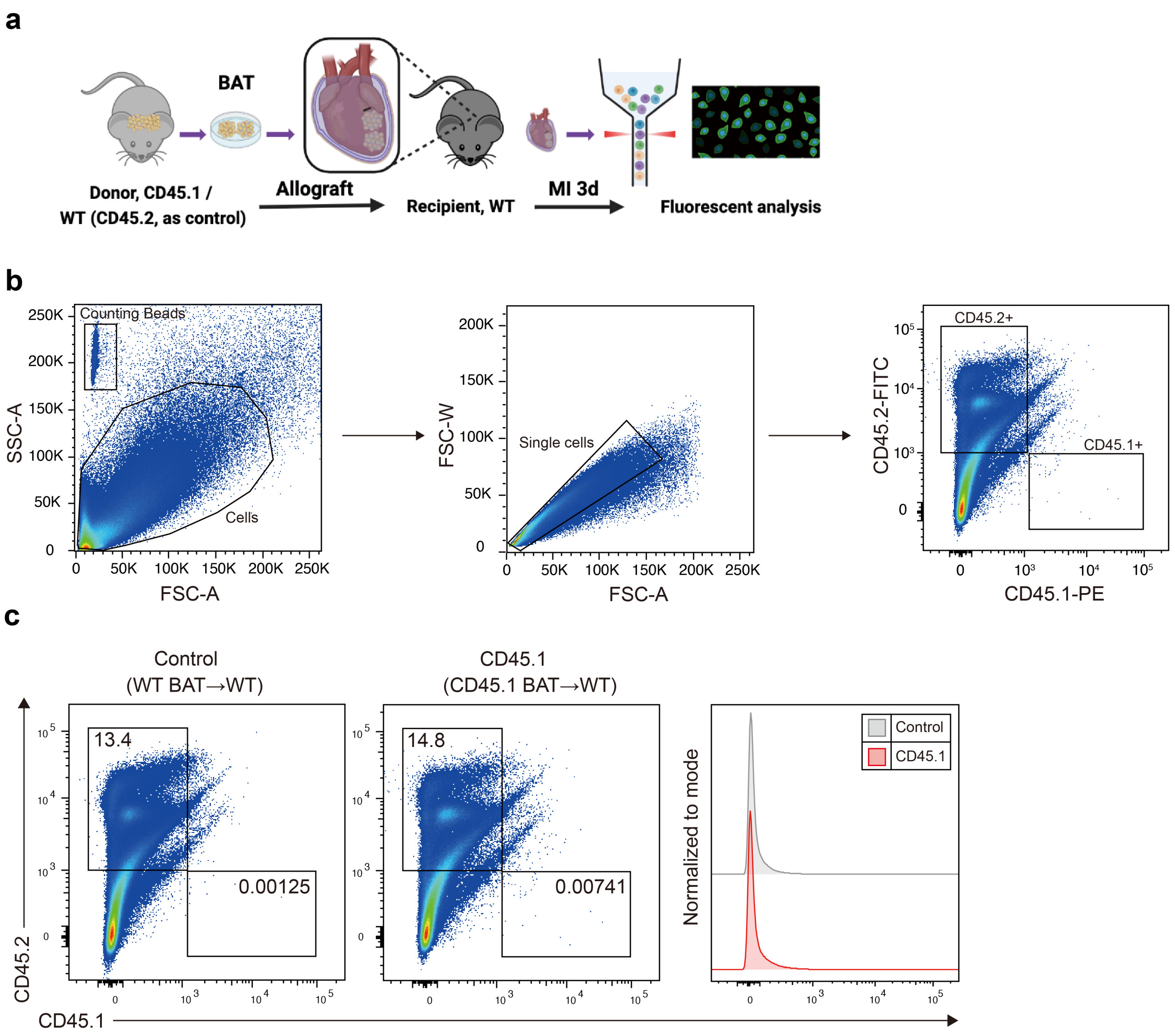

Supplementary Fig. 4 Immune cell interorgan transfer is not involved in BAT cardioprotection.

a, Schematic of BAT transplantation from CD45.1 or CD45.2 (as control) to WT (CD45.2) mice. Recipient mouse hearts are analyzed by flow cytometry at MI 3d. *Created in BioRender. https://BioRender.com/4breevc.*

b, Gating strategy for CD45.1 and CD45.2 immune cells in flow cytometric analysis of wildtype recipient mice heart.

c, Identification of CD45.1 and CD45.2 cells in recipient mice heart on dot plot and histogram (n=3 mice per group).

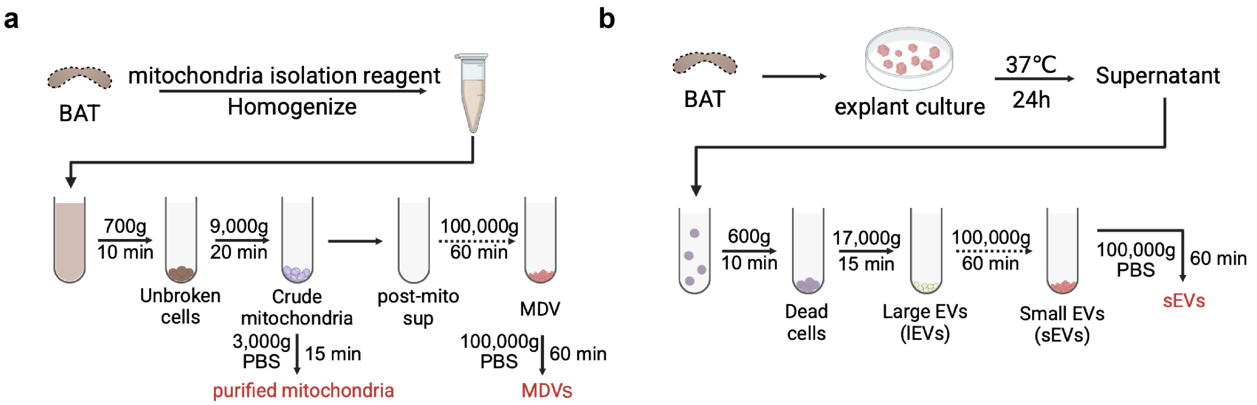

Supplementary Fig. 5 Isolation of MDVs and sEVs from mouse tissue.

a, Schematic illustration of the workflow used to enrich mitochondria-derived vesicles (MDVs) from mouse adipose tissue.

b, Schematic illustration of the protocol to enrich sEVs from mouse tissue.

Created in BioRender. https://BioRender.com/c0dlvaq.

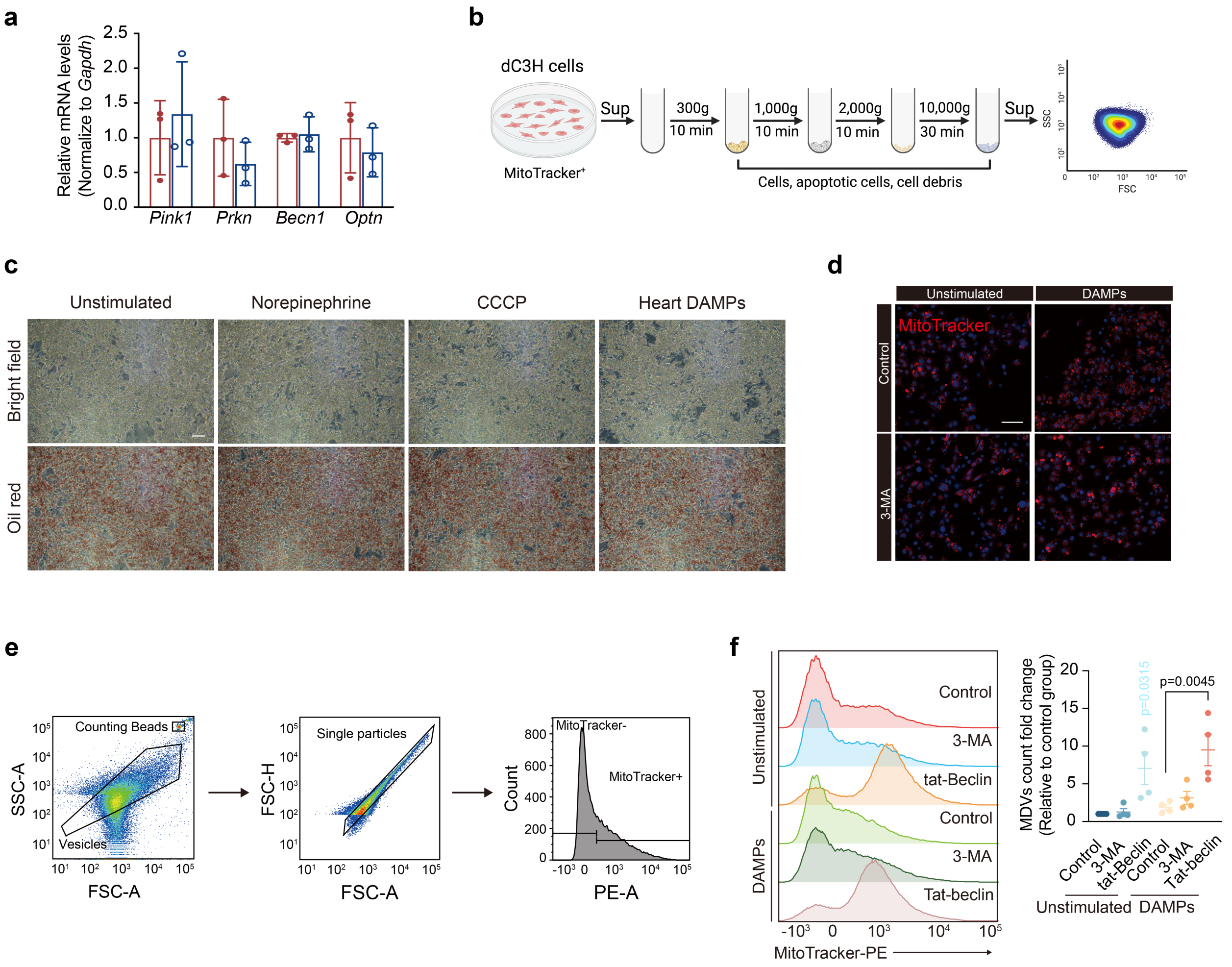

Supplementary Fig. 6 Flow cytometric analysis for MDVs production in brown adipocytes.

a, RT-qPCR analysis of mitophagy genes (*Pink1, Prkn, Becn1, Optn*) in BAT at MI 3d (mean±s.d., n=3, unpaired Student's t test).

b, Schematic of cell culture media treatment for MDVs detection by flow cytometric analysis. *Created in BioRender. https://BioRender.com/0eqo3zy.*

c, Representative bright field images and oil red staining images of NE (1 μM) or CCCP (10 μM) or heart DAMPs (20 μg/mL)-treated brown adipocytes, which were differentiated from the C3H10T1/2 cell line. Scale bar: 100 μm.

d, Representative images of differentiated brown adipocytes stained by Mitotracker (500 nM) and treated with 3-MA (10 mM) and heart DAMPs (20 μg/mL) or PBS (unstimulated). Scale bar: 100 μm.

e, Gating strategy for MDVs on flow cytometry. FSC-A and SSC-A thresholds were adjusted to 500.

f, Flow cytometric histogram of MitoTracker+ MDVs release upon heart DAMPs (20 μg/mL) stimulation for 4 hours. Brown adipocytes were pre-stained by MitoTracker (500 nM) and incubated with complete media(control) or *Becn1* inhibitor 3-MA (10 mM) or BECN1 mimetic Tat-beclin (10 μM) media before DAMPs treatment. MitoTracker$^+$ vesicles count was quantified and normalized by precise counting beads (mean±s.e.m., n=4 independent experiments, paired one-way ANOVA with Bonferroni correction).

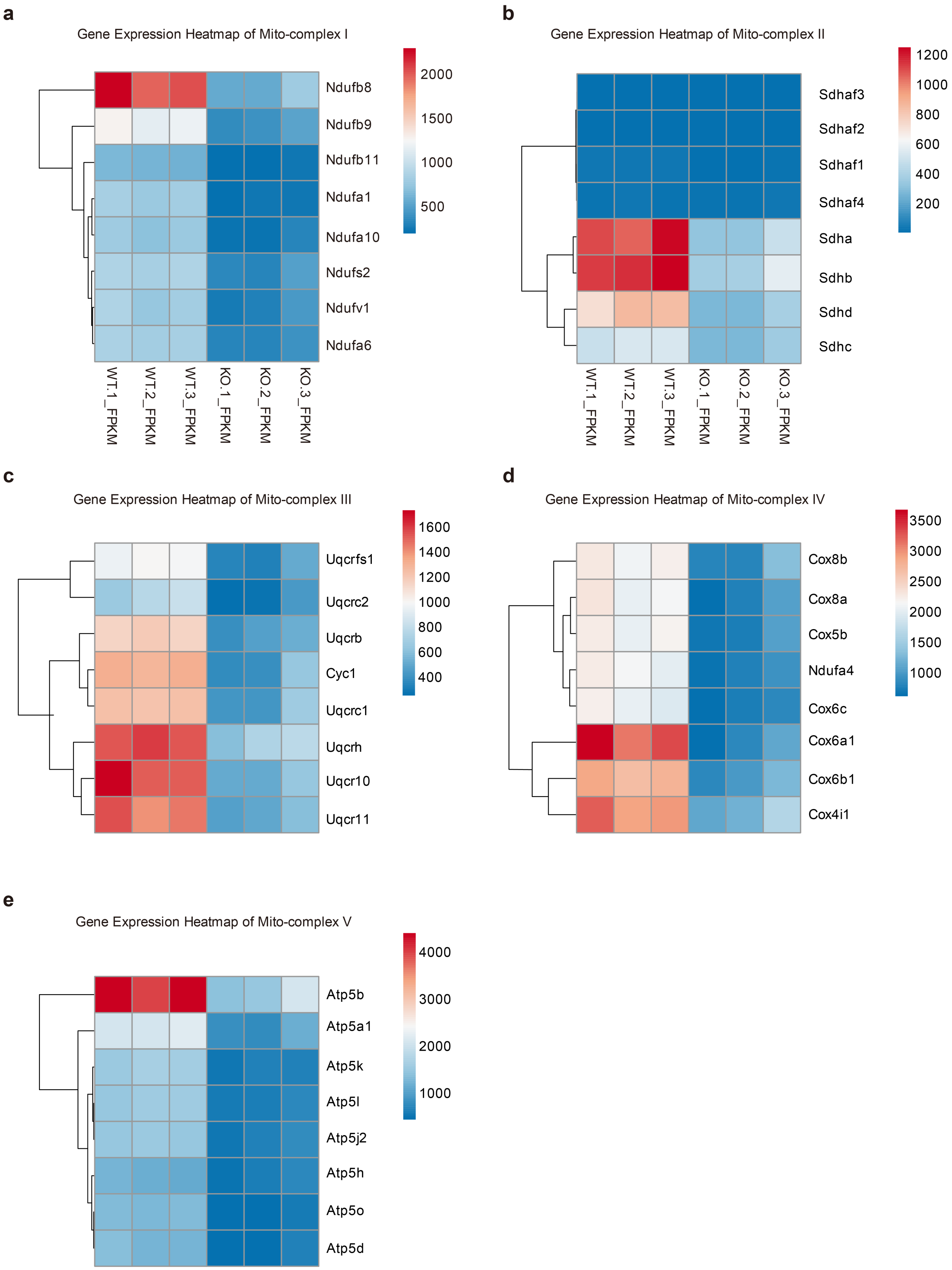

Supplementary Fig. 7 The mitochondrial respiratory chain gene expression in WT

and *Becn1*$^{+/-}$ BAT.

a-e, Heatmap showing the differentially expressed genes (DEGs) of mitochondrial respiratory chain complex I-V top8 highly expressed subunits in WT and *Becn1*$^{+/-}$ BAT. The data were from mouse BAT bulk RNA sequencing (GSE148275).

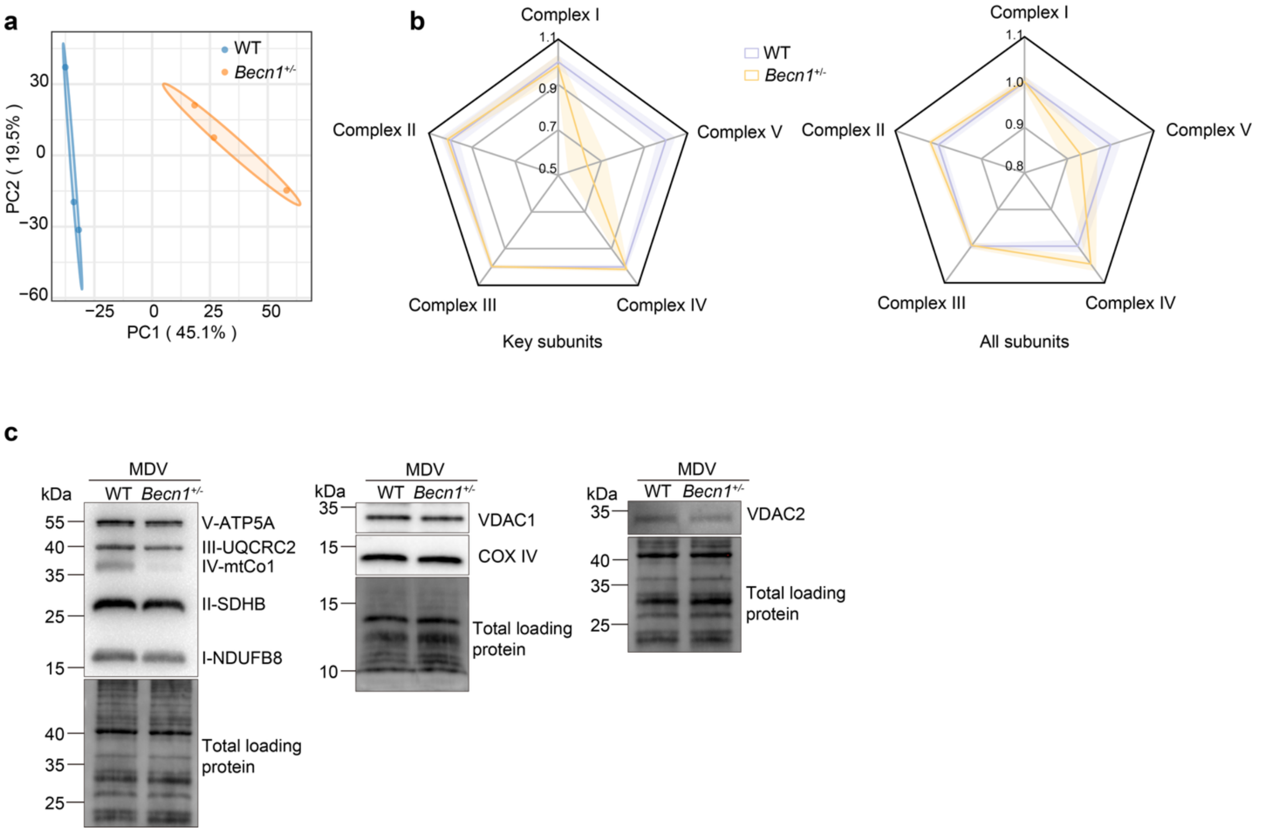

Supplementary Fig. 8 The mitochondrial respiratory chain proteins in MDVs.

a, Principal component analysis (PCA) of quantitative proteomics of WT and *Becn1*$^{+/-}$ MDVs.

b, Relative mitochondrial respiratory chain complex scores based on key or all subunit protein levels (ssGSEA) in WT and *Becn1*$^{+/-}$ MDVs (n=3 mice per group).

c, Western blots of WT and *Becn1*$^{+/-}$ BAT MDVs. The respiratory chain complex I-V representative subunit of WT and *Becn1*$^{+/-}$ MDVs were compared. The experiment was independently repeated 3 times with similar results.

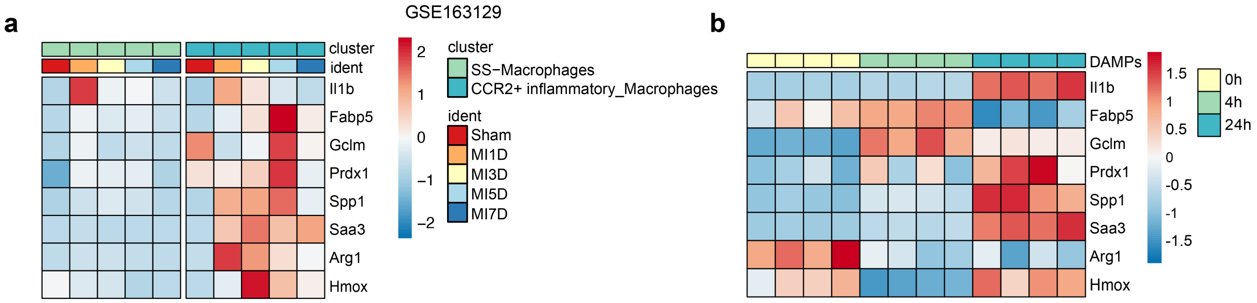

Supplementary Fig. 9 DAMPs-stimulated iBMDM as a cell model for macrophages in MI.

a, Inflammation and lipid metabolism-related gene expression in $CCR2^+$ inflammatory macrophages, the major macrophage subpopulation during MI 3d-5d. The data were from mouse single-cell RNA sequencing (GSE163129). SS-Macrophage, steady-state macrophage.

b, RT-qPCR analysis of above-mentioned genes in DAMPs-stimulated iBMDM cells at 4 hours and 24 hours (n=4 independent experiments).

Supplementary Table 1. qPCR primers sequences.

Gene Name	Species	Forward primer (5'→3')	Reverse Primer (3'→5')
Ucp1	mus	AGGCTTCCAGTACCATTAGGT	CTGAGTGAGGCAAAGCTGATTT
Pparg	mus	TCGCTGATGCACTGCCTATG	GAGAGGTCCACAGAGCTGATT
Fabp4	mus	AAGGTGAAGAGCATCATAACCCT	TCACGCCTTTCATAACACATTCC
Elovl3	mus	TTCTCACGCGGGTTAAAAATGG	GAGCAACAGATAGACGACCAC
Ppargc1a	mus	TATGGAGTGACATAGAGTGTGCT	CCACTTCAATCCACCCAGAAAG
Id2	mus	GCATCCTGTCCTTGCAGGCATCTG	AGTCCAGGCCGGAGAACGACA
Dnm1l (Drp1)	mus	TTACGGTTCCCTAAACTTCACG	GTCACGGGCAACCTTTTACGA
Snx9	mus	ACCAAGGCTCGGGTCATGTA	ACCAACATTCGGGTTTGTAACTG
Tollip	mus	CCTCAGCCCCGCTGTAATG	CAGCATCTTTGTTCCCTCTCTG
Vps35	mus	GCTGTGAAGGTTCAGTCATTCC	GTCAGGTAGACCTCCAAGTAGT
Vps29	mus	CTGCACCAAGGAGAGCTACG	TCAGACCGATCTTGAACTGGC
Vps26a	mus	AAGAGGCTAGAGCATCAAGGA	TTCTCCAGGCAAGGCTAGTTC
Pink1	mus	TTCTTCCGCCAGTCGGTAG	CTGCTTCTCCTCGATCAGCC
Prkn	mus	TCTTCCAGTGTAACCACCGTC	GGCAGGGAGTAGCCAAGTT
Becn	mus	ATGGAGGGGTCTAAGGCGTC	TCCTCTCCTGAGTTAGCCTCT
Optn	mus	ATGTCCCATCAACCTCTGAGC	TCAAATCGCCCTTTCATAGCTTG
Il1b	mus	GCAACTGTTCCTGAACTCAACT	ATCTTTTGGGGTCCGTCAACT
Arg1	mus	GTGAAGAACCCACGGTCTGT	GCACCACACTGACTCTTCCA
Vegf	mus	GCACATAGAGAGAATGAGCTTCC	CTCCGCTCTGAACAAGGCT
Il10	mus	GCTCTTACTGACTGGCATGAG	CGCAGCTCTAGGAGCATGTG
Il4	mus	GGTCTCAACCCCCAGCTAGT	GCCGATGATCTCTCTCAAGTGAT
Fabp5	mus	TGAAAGAGCTAGGAGTAGGACTG	CTCTCGGTTTTGACCGTGATG
Gclm	mus	AGGAGCTTCGGGACTGTATCC	GGGACATGGTGCATTCCAAAA
Prdx1	mus	AATGCAAAAATTGGGTATCCTGC	CGTGGGACACACAAAAGTAAAGT
Spp1	mus	AGCAAGAAACTCTTCCAAGCAA	GTGAGATTCGTCAGATTCATCCG
Saa3	mus	TGCCATCATTCTTTGCATCTTGA	CCGTGAACTTCTGAACAGCCT
Hmox1	mus	AAGCCGAGAATGCTGAGTTCA	GCCGTGTAGATATGGTACAAGGA
Gapdh	mus	AGGTCGGTGTGAACGGATTTG	TGTAGACCATGTAGTTGAGGTCA

Supplementary Table 2. Clinical characteristics of donor controls and patients with HFrEF included in the RNA-seq data re-analysis.

Variable	Controls (n=21)	HFrEF (n=29)	*p* value
Age, y	58.0 (48.5, 63.0)	50.0 (44.5, 61.5)	0.1298
Sex (Female)	8 (38.1)	9 (31.0)	0.6029
Race/ethnicity			0.0613
Black	1 (4.8)	8 (27.6)	
White	19 (90.5)	20 (69.0)	
Hispanic	1 (4.8)	0 (0)	
Other	0 (0)	1 (3.4)	
Medications			
ACEi or ARB	5 (23.8)	19 (65.5)	0.0036
β-blocker	5 (23.8)	27 (93.1)	<0.0001
Loop diuretic	0 (0)	29 (100.0)	<0.0001
History			
Hypertension	10 (47.6)	29 (100.0)	<0.0001
Diabetes	3 (14.3)	9 (31.0)	0.1711
Coronary artery disease	1 (4.8)	5 (17.2)	0.3684
Atrial fibrillation or flutter	3 (14.3)	18 (62.1)	0.0007
BMI, kg/m^2	27.0 (22.9, 31.9)	26.2 (23.6, 30.3)	0.7088
eGFR, $mL/min/1.73m^2$	90.0 (56.5, 106.5)	70.0 (55.0, 84.5)	0.1351

ACEi, angiotensin-converting enzyme inhibitor; ARB, angiotensin II receptor blocker; BMI, body mass index; eGFR, estimated glomerular filtration rate.

Age, presented by median (25th, 75th percentile), p value assessed using two-sided Mann-Whitney U test; Sex, presented by female count (percentage), p value assessed using the two-sided Pearson chi-square test; Race, presented by count (percentage), p value assessed using the two-sided Fisher's exact test; ACEi or ARB, β-blocker, and Loop diuretic, presented by positive count (percentage), p value assessed using the two-sided Pearson chi-square test; Hypertension, presented by positive count (percentage), p value assessed using the two-sided Pearson chi-square test with Yates' continuity correction; Diabetes, presented by positive count (percentage), p value assessed using the two-sided

Pearson chi-square test; Coronary artery disease, presented by positive count (percentage), p value assessed using the two-sided Pearson chi-square test with Yates' continuity correction; Atrial fibrillation or flutter, presented by positive count (percentage), p value assessed using the two-sided Pearson chi-square test; BMI and eGFR, presented by median (25th, 75th percentile), p value assessed using two-sided Mann-Whitney U test.

Full blots in Supplementary Figures

Figure S8C

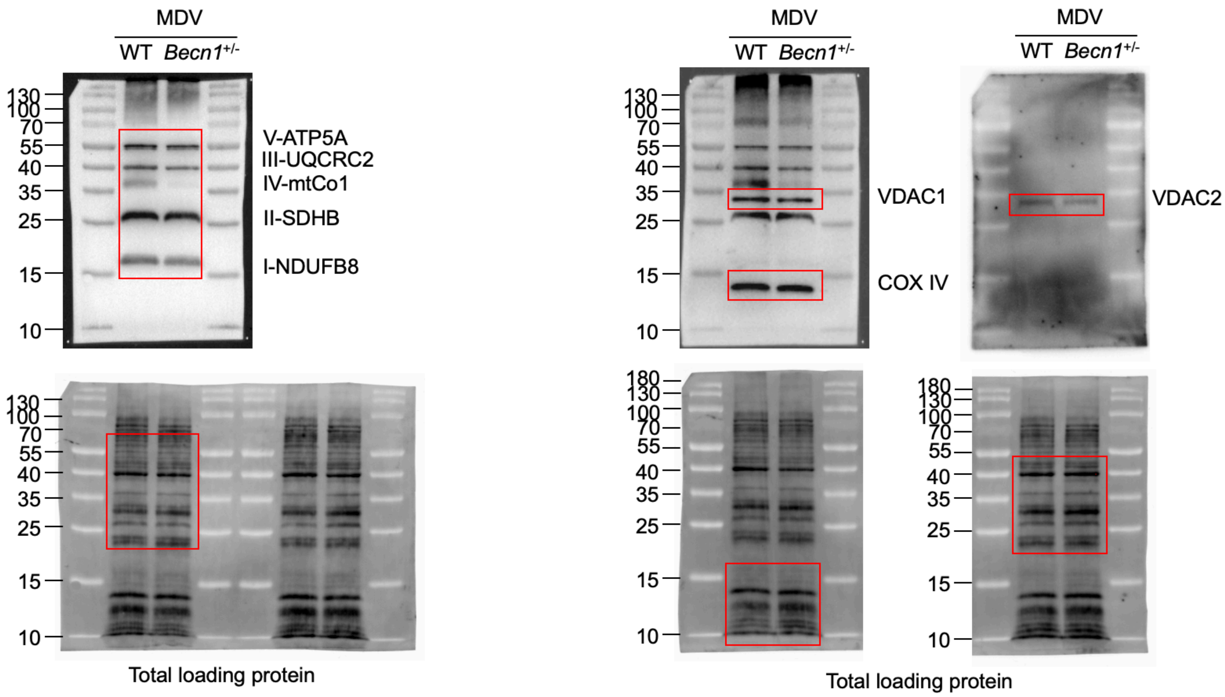

Supplement: Supplementary file 1 — Supplementary Information [file 41467_2026_73388_MOESM1_ESM.pdf]
