# Supplementary material for: Mitochondria-derived vesicles with bioenergetic units from brown adipose tissue attenuate cardiac remodeling post-myocardial infarction
Source: Nat Commun. 2026 May 21;17:6690. doi: 10.1038/s41467-026-73388-3 (PMC13385750; doi:10.1038/s41467-026-73388-3)
Supplement: Supplementary file 2 — Description of Additional Supplementary Files [file 41467_2026_73388_MOESM2_ESM.pdf]

Description of Additional Supplementary Files

File Name: Supplementary Data 1

Description: Mouse genes both involved in vesicle-mediated transport (GO: 0016192) and mitochondrial-associated process (GO:0000423 and GO:0007005).

File Name: Supplementary Data 2

Description: Information for antibodies in this study.
