# Supplementary material for: Mitochondria-derived vesicles with bioenergetic units from brown adipose tissue attenuate cardiac remodeling post-myocardial infarction
Source: Nat Commun. 2026 May 21;17:6690. doi: 10.1038/s41467-026-73388-3 (PMC13385750; doi:10.1038/s41467-026-73388-3)
Supplement: Supplementary file 5 — Reporting Summary [file 41467_2026_73388_MOESM5_ESM.pdf]

nature portfolio

Corresponding author(s): Yaozu Xiang

Last updated by author(s): Apr 15, 2026

Reporting Summary

Nature Portfolio wishes to improve the reproducibility of the work that we publish. This form provides structure for consistency and transparency in reporting. For further information on Nature Portfolio policies, see our Editorial Policies and the Editorial Policy Checklist.

Statistics

For all statistical analyses, confirm that the following items are present in the figure legend, table legend, main text, or Methods section.

n/a	Confirmed	
☐	☒	The exact sample size (*n*) for each experimental group/condition, given as a discrete number and unit of measurement
☐	☒	A statement on whether measurements were taken from distinct samples or whether the same sample was measured repeatedly
☐	☒	The statistical test(s) used AND whether they are one- or two-sided<br>*Only common tests should be described solely by name; describe more complex techniques in the Methods section.*
☒	☐	A description of all covariates tested
☐	☒	A description of any assumptions or corrections, such as tests of normality and adjustment for multiple comparisons
☐	☒	A full description of the statistical parameters including central tendency (e.g. means) or other basic estimates (e.g. regression coefficient) AND variation (e.g. standard deviation) or associated estimates of uncertainty (e.g. confidence intervals)
☐	☒	For null hypothesis testing, the test statistic (e.g. *F*, *t*, *r*) with confidence intervals, effect sizes, degrees of freedom and *P* value noted<br>*Give P values as exact values whenever suitable.*
☒	☐	For Bayesian analysis, information on the choice of priors and Markov chain Monte Carlo settings
☒	☐	For hierarchical and complex designs, identification of the appropriate level for tests and full reporting of outcomes
☐	☒	Estimates of effect sizes (e.g. Cohen's *d*, Pearson's *r*), indicating how they were calculated

Our web collection on statistics for biologists contains articles on many of the points above.

Software and code

Policy information about availability of computer code

Data collection	RT-qPCR data were acquired with Applied Biosystems 7500 Software (v.2.0.6). Western blotting was acquired by BioRad Image Lab (v.5.2.1). The confocal images were acquired with Nikon NIS-Elements (v.5.02.03). Flow cytometry data were collected with BD FACSDiva (v9.0). The absorbance and fluorescent emission data were acquired by Tecan i-control (1.9.17.0).
Data analysis	Data were analyzed by Prism (v.9.5). Images were quantified in ImageJ (v2.9.0). Flowcytometric data were analyzed with FlowJo (v.10.8.1). For RNA-seq generated in this study, raw reads files were processed using fastp1 (v.0.20.1). RNA quality control was performed by RseQC (v.4.0.0). Low-quality reads were identified by fastqc (v0.11.9) and removed. The clean reads were mapped to the reference genome NCBI_GRCm39 using HISAT2 (v.2.1.0). FPKM and read counts of each gene were calculated by HTSeq-count (v.0.11.2). PCA analysis was performed using R (v3.2.0). Differential expression analysis was performed using the DESeq2 (v.1.22.2). GO pathway analysis was performed using R (v3.2.0). The data were processed using DIA-NN search engine (v.1.8). For proteomics data, the acquired tandem mass spectra were searched against the Mus_musculus_10090_SP_20231220.fasta (FDR < 1%). The protein complex score was calculated by R (v.4.4.2) and GSVA (1.50.5). The RNAseq and Proteomics data were visualized by R (v.4.4.2), ggplot2 (v.3.5.0), ggpubr (v.0.5.0), and ggridges (v.0.5.6).<br>For Previously published RNAseq data of human myocardial samples, analysis was conducted by R (v.4.4.2), BayesPrism (2.2.2), Seurat (4.3.0), and SeuratObject (4.1.3).

For manuscripts utilizing custom algorithms or software that are central to the research but not yet described in published literature, software must be made available to editors and reviewers. We strongly encourage code deposition in a community repository (e.g. GitHub). See the Nature Portfolio guidelines for submitting code & software for further information.

Data

Policy information about availability of data

All manuscripts must include a data availability statement. This statement should provide the following information, where applicable:

- Accession codes, unique identifiers, or web links for publicly available datasets
- A description of any restrictions on data availability
- For clinical datasets or third party data, please ensure that the statement adheres to our policy

The infarct left ventricular RNA-seq data generated in this study, including raw and processed files, have been deposited in the Gene Expression Omnibus (GEO) under accession code GSE291141. The mass spectrometry proteomics data generated in this study have been deposited in the ProteomeXchange Consortium via the iProX partner repository under dataset identifier PXD061744.
Previously published data re-analyzed in this study are available. The single-cell RNA-seq data of MI mouse cardiac leukocytes are accessed under GSE163129. RNA-seq data of WT or adipocyte-specific Becn1 KO mice BAT are available under GSE148275. RNA-seq data of human myocardial samples are available on Zenodo Data Repository (doi: 10.5281/zenodo.4114617).

Research involving human participants, their data, or biological material

Policy information about studies with human participants or human data. See also policy information about sex, gender (identity/presentation), and sexual orientation and race, ethnicity and racism.

Reporting on sex and gender	The RNA-seq data from donor control and patients with HFrEF were from previously published work (Hahn et al, Circ, 2021). The re-analysis included 50 patients with available left ventricular myocardium sample RNA-seq data. The male:female ratio is approximately 2:1. Age and sex difference was not observed between control and HFrEF groups.
Reporting on race, ethnicity, or other socially relevant groupings	Participants included individuals of White, Black, Hispanic and other race backgrounds. Other social groupings were not relevant to this study.
Population characteristics	The clinical characteristics of patients, including age, medications, and medical history, were listed and compared in Supplementary Table 3.
Recruitment	N/A
Ethics oversight	N/A

Note that full information on the approval of the study protocol must also be provided in the manuscript.

Field-specific reporting

Please select the one below that is the best fit for your research. If you are not sure, read the appropriate sections before making your selection.

☒ Life sciences ☐ Behavioural & social sciences ☐ Ecological, evolutionary & environmental sciences

For a reference copy of the document with all sections, see nature.com/documents/nr-reporting-summary-flat.pdf

Life sciences study design

All studies must disclose on these points even when the disclosure is negative.

Sample size	Sample size was determined based on previous experience and accepted standard in the field. All the sample sizes were reported in the figure legends.
Data exclusions	Data were excluded only when the mice died during the surgery.
Replication	Every experiments was independently repeated with at lease three biological replicates. All attempts at replication were successful.
Randomization	All animals were randomly assigned to each group with ear labeling and body weight records.
Blinding	A single-blind method was applied to reduce objective bias. The analysis experiments and data assessments were conducted without awareness of the treatment condition of the subjects.

Reporting for specific materials, systems and methods

We require information from authors about some types of materials, experimental systems and methods used in many studies. Here, indicate whether each material, system or method listed is relevant to your study. If you are not sure if a list item applies to your research, read the appropriate section before selecting a response.

Materials & experimental systems

n/a	Involved in the study
☐	☒ Antibodies
☐	☒ Eukaryotic cell lines
☒	☐ Palaeontology and archaeology
☐	☒ Animals and other organisms
☒	☐ Clinical data
☒	☐ Dual use research of concern
☒	☐ Plants

Methods

n/a	Involved in the study
☒	☐ ChIP-seq
☐	☒ Flow cytometry
☒	☐ MRI-based neuroimaging

Antibodies

Antibodies used

Antibodies for immunohistofluorecence (IHF), western blotting (WB), ransmission electron microscopy (TEM), and flow cytometry (FC) are listed below:
Anti-Sarcomeric Alpha Actinin antibody 1:500 (IHF) ab137346 Abcam;
TOMM20 Recombinant Rabbit Monoclonal Antibody 1:200 (IHF),1:2000 (WB) ET1609-25 HUABIO
Cytochrome C Recombinant Rabbit Monoclonal Antibody 1:50 (IHF),1:1000 (WB) ET1610-60 HUABIO
Anti-Pyruvate dehydrogenase E2/E3bp antibody 1:1000 (IHF, WB) ab110333 Abcam
Rat anti Mouse CD68 antibody 1:100 (IHF) MCA1957 Bio-Rad;
Human/Mouse Myeloperoxidase/MPO Antibody 15μg/mL (IHF) AF3667 R&D SYSTEMS;
GFP Polyclonal Antibody 1:500 (PLA; IHC) A-11122 Invitrogen;
GFP Monoclonal Antibody 1:500 (PLA) MA5-15256 Invitrogen;
Alpha smooth muscle actin specific Monoclonal antibody 1:3000 (IHC) 67735-1-Ig Proteintech;
Goat anti-Rabbit IgG (H+L) Cross-Adsorbed Secondary Antibody, Texas Red 1:1000 (IHC) T-2767 Invitrogen;
Donkey anti-Mouse IgG (H+L) Highly Cross-Adsorbed Secondary Antibody, Alexa Fluor™ 647 1:1000 (IHC) A-31571 Invitrogen;
Goat anti-Rat IgG (H+L) Cross-Adsorbed Secondary Antibody, Alexa Fluor™ 647 1:1000 (IHC) A-21247 Invitrogen;
Donkey anti-Goat IgG (H+L) Cross-Adsorbed Secondary Antibody, Alexa Fluor™ 555 1:1000 (IHC) A-21432 Invitrogen;
GFP Recombinant Rabbit Monoclonal Antibody 1:50 (TEM) ET1607-31 HUABIO;
12 nm Colloidal Gold AffiniPure™ Goat Anti-Rabbit IgG (H+L) 1:25 (TEM) 111-205-144 Jackson;
UCP1 Rabbit pAb 1:1000 (WB),1:100 (IHC) A5857 ABclonal;
ATP5A1 Rabbit mAb 1:2000 (WB) A11217 ABclonal;
UQCRC2 Rabbit pAb 1:2000 (WB) A4181 ABclonal;
COX1 Rabbit mAb 1:1000 (WB) A23123 ABclonal;
SDHB Rabbit mAb 1:10000 (WB) A23832 ABclonal;
NDUFB8 Rabbit mAb 1:10000 (WB) A19732 ABclonal;
ALIX Recombinant Rabbit Monoclonal Antibody 1:1000 (WB) ET1705-74 HUABIO;
Calnexin Rabbit pAb 1:1000 (WB) A24433 ABclonal;
TSG101/VPS23 Rabbit mAb 1:1000 (WB) A5789 ABclonal;
CD63 Rabbit mAb (A19023) 1:1000 (WB) A19023 ABclonal;
VPS35 Rabbit mAb 1:1000 (WB) A9278 ABclonal;
Beclin-1 (D40C5) Rabbit mAb 1:500 (WB) 3495 Cell Signaling Technology;
Arginase-1 Polyclonal Antibody 1:1000 (WB) 16001-1-AP Proteintech;
iNOS Polyclonal Antibody 1:2000 (WB) 18985-1-AP Proteintech;
β-Actin Rabbit mAb (High Dilution) 1:100000 (WB) AC026 ABclonal;
GAPDH Mouse mAb (High Dilution) 1:10000 (WB) AC033 ABclonal;
VDAC1 Rabbit mAb 1:5000 (WB) A19707 ABclonal;
VDAC2 Rabbit mAb 1:20000 (WB) A21260 ABclonal;
COX IV Rabbit mAb 1:1000 (WB) A11631 ABclonal;
Goat anti-Rabbit IgG (H+L) Secondary Antibody, HRP 1:10000 (WB) 31460 Invitrogen;
Goat anti-Mouse IgG (H+L) Secondary Antibody, HRP 1:10000 (WB) 31430 Invitrogen;
PE anti-mouse CD45.1 Antibody 1:300 (FC) 110707 BioLegend;
FITC anti-mouse CD45.2 Antibody 1:300 (FC) 109805 BioLegend.

Validation

Primary antibody validation information on manufacturer's website:
Anti-Sarcomeric Alpha Actinin antibody, https://www.abcam.com/en-us/productsALIX Recombinant Rabbit Monoclonal Antibody/primary-antibodies/sarcomeric-alpha-actinin-antibody-ab137346;
TOMM20 Recombinant Rabbit Monoclonal Antibody, https://huabio.cn/products/TOMM20-antibody-ET1609-25;
Cytochrome C Recombinant Rabbit Monoclonal Antibody, https://huabio.cn/products/Cytochrome-C-antibody-ET1610-60;
Anti-Pyruvate dehydrogenase E2/E3bp antibody, https://www.abcam.com/en-us/products/primary-antibodies/pyruvate-dehydrogenase-e2-e3bp-antibody-13g2ae2bh5-ab110333;
Rat anti Mouse CD68 antibody, https://www.bio-rad-antibodies.com/monoclonal/mouse-cd68-antibody-fa-11-mca1957.html;
Human/Mouse Myeloperoxidase/MPO Antibody, https://www.rndsystems.com/cn/products/human-mouse-myeloperoxidase-mpo-antibody_af3667;
GFP Polyclonal Antibody, https://www.thermofisher.com/antibody/product/GFP-Antibody-Polyclonal/A-11122;
GFP Monoclonal Antibody, https://www.thermofisher.com/antibody/product/GFP-Antibody-clone-GF28R-Monoclonal/MA5-15256;

GFP Recombinant Rabbit Monoclonal Antibody, https://huabio.cn/products/GFP-antibody-ET1607-31;
Alpha smooth muscle actin specific Monoclonal antibody, https://www.ptglab.com/products/smooth-muscle-actin-specific-Antibody-67735-1-Ig.htm;
UCP1 Rabbit pAb, https://abclonal.com/catalog-antibodies/UCP1RabbitpAb/A5857;
ATP5A1 Rabbit mAb, https://abclonal.com/catalog-antibodies/ATP5A1RabbitmAb/A11217;
UQCRC2 Rabbit pAb, https://abclonal.com/catalog-antibodies/UQCRC2RabbitpAb/A4181;
COX1 Rabbit mAb, https://abclonal.com/catalog-antibodies/COX1RabbitmAb/A23123;
SDHB Rabbit mAb, https://abclonal.com/catalog-antibodies/SDHBRabbitmAb/A23832;
ALIX Recombinant Rabbit Monoclonal Antibody,https://huabio.cn/products/ALIX-antibody-ET1705-74;
Calnexin Rabbit pAb, https://abclonal.com/catalog-antibodies/CalnexinRabbitpAb/A24433;
TSG101/VPS23 Rabbit mAb, https://abclonal.com.cn/catalog/A5789;
CD63 Rabbit mAb, https://abclonal.com.cn/catalog/A19023;
NDUFB8 Rabbit mAb, https://abclonal.com/catalog-antibodies/NDUFB8RabbitmAb/A19732;
VPS35 Rabbit mAb, https://abclonal.com/catalog-antibodies/VPS35RabbitmAb/A9278#section2;
Beclin-1 (D40C5) Rabbit mAb, https://www.cellsignal.cn/products/primary-antibodies/beclin-1-d40c5-rabbit-mab/3495;
Arginase-1 Polyclonal Antibody, https://www.ptglab.com/products/ARG1-Antibody-16001-1-AP.htm;
iNOS Polyclonal Antibody, https://www.ptglab.com/products/NOS2-Antibody-18985-1-AP.htm;
β-Actin Rabbit mAb (High Dilution); https://abclonal.com/catalog-antibodies/ActinRabbitmAbHighDilution/AC026;
GAPDH Mouse mAb (High Dilution); https://abclonal.com/catalog-antibodies/GAPDHMousemAbHighDilution/AC033;
VDAC1 Rabbit mAb, https://abclonal.com/catalog-antibodies/VDAC1RabbitmAb/A19707;
VDAC2 Rabbit mAb; https://abclonal.com/catalog-antibodies/VDAC2RabbitmAb/A21260;
COX IV Rabbit mAb, https://abclonal.com/catalog-antibodies/COXIVRabbitmAb/A11631.
PE anti-mouse CD45.1 Antibody, https://www.biolegend.com/en-gb/products/pe-anti-mouse-cd45-1-antibody-199;
FITC anti-mouse CD45.2 Antibody, https://www.biolegend.com/en-gb/products/fitc-anti-mouse-cd45-2-antibody-6.

Eukaryotic cell lines

Policy information about cell lines and Sex and Gender in Research

Cell line source(s)	C3H10T1/2 cells from National collection of authenticated cell cultures (GNM19); iBMDM cells from OriCell (M3-1001).
Authentication	C3H10T1/2 and iBMDM cell line were STR confirmed mouse origin with no human or other species contamination by the vendors, and were authenticated by the ability to generate adipocytes upon induction or the ability to phagocytose and respond to LPS stimulation. Mycoplasma infection was tested by PCR every ten passages.
Mycoplasma contamination	Cell lines were tested negative for mycoplasma contamination by the mycoplasma-specific primer PCR.
Commonly misidentified lines (See ICLAC register)	No commonly misidentified cell lines.

Animals and other research organisms

Policy information about studies involving animals; ARRIVE guidelines recommended for reporting animal research, and Sex and Gender in Research

Laboratory animals	All wildtype (WT) C57BL/6J male mice (4-12 weeks, male) were purchased from SPF (Beijing) Biotechnology Co., Ltd. Becn1+/- and B6-G/R fl/+ mice (both from C57BL/6J background) were purchased from GemPharmatech Co., Ltd. All mice were housed in specific-pathogen-free environment at 22 degrees Celsius and 40-60% humidity, with 12/12 h light/dark cycle and free access to distilled water and sterilized chow diet.
Wild animals	No wild animal used.
Reporting on sex	All mice used in the manuscript are male.
Field-collected samples	N/A
Ethics oversight	Animal experiments were approved by the Institutional Animal Care and Use Committee of Tongji University (TJ-HB-LAC-2024-16).

Note that full information on the approval of the study protocol must also be provided in the manuscript.

Plants

Seed stocks	No seed stocks were used in this study.
Novel plant genotypes	*Describe the methods by which all novel plant genotypes were produced. This includes those generated by transgenic approaches, gene editing, chemical/radiation-based mutagenesis and hybridization. For transgenic lines, describe the transformation method, the number of independent lines analyzed and the generation upon which experiments were performed. For gene-edited lines, describe the editor used, the endogenous sequence targeted for editing, the targeting guide RNA sequence (if applicable) and how the editor was applied.*
Authentication	*Describe any authentication procedures for each seed stock used or novel genotype generated. Describe any experiments used to assess the effect of a mutation and, where applicable, how potential secondary effects (e.g. second site T-DNA insertions, mosiacism, off-target gene editing) were examined.*

Flow Cytometry

Plots

Confirm that:

- [x] The axis labels state the marker and fluorochrome used (e.g. CD4-FITC).
- [x] The axis scales are clearly visible. Include numbers along axes only for bottom left plot of group (a 'group' is an analysis of identical markers).
- [x] All plots are contour plots with outliers or pseudocolor plots.
- [x] A numerical value for number of cells or percentage (with statistics) is provided.

Methodology

Sample preparation	For Mouse heart immune cell analysis, samples were minced, digested, and centrifuged at 300g to pellet single cells. Cells were incubated with PE anti-mouse CD45.1 Antibody and FITC anti-mouse CD45.2 Antibody, both 1:300 dilution for 30 min in the dark. For MDV detection, dC3H supernatant was collected to enrich MDVs as described in the manuscript.
Instrument	BD FACSAria III
Software	BD FACSDiva (v.9.0)
Cell population abundance	For mouse heart immune cell analysis, approximately 1x10^5 cells/sample were analyzed. For MDV detection, supernatant from 2x10^6 cells was used for MDV enrichment.
Gating strategy	Cells and vesicles were gated on SSC-A and FSC-A to select primary population, then they were gated on FSC-W/FSC-H and FSC-A to select single particles. Subsequent gating method are reported in Extended data.

- [x] Tick this box to confirm that a figure exemplifying the gating strategy is provided in the Supplementary Information.
