# Supplementary material for: Mitochondria-derived vesicles with bioenergetic units from brown adipose tissue attenuate cardiac remodeling post-myocardial infarction
Source: Nat Commun. 2026 May 21;17:6690. doi: 10.1038/s41467-026-73388-3 (PMC13385750; doi:10.1038/s41467-026-73388-3)
Supplement: Supplementary file 6 — Transparent Peer Review file [file 41467_2026_73388_MOESM6_ESM.pdf]

nature portfolio

Peer Review File

Mitochondria-derived-vesicles with bioenergetic units from brown adipose tissue attenuate cardiac remodeling post-myocardial infarction

Corresponding Author: Professor Yaozu Xiang

Parts of this Peer Review File have been redacted as indicated to remove third-party material.

This file contains all reviewer reports in order by version, followed by all author rebuttals in order by version.

Version 0:

Reviewer comments:

Reviewer #1

(Remarks to the Author)
This study explores the therapeutic potential of brown adipose tissue (BAT)-derived mitochondria-derived vesicles (MDVs) in mitigating cardiac remodeling post-myocardial infarction (MI). While the proposed mechanism is intriguing, the current manuscript has multiple critical gaps that undermine the validity of the conclusions. In particular, the lack of key experimental details, insufficient validation of proposed mechanisms, and questionable data interpretation collectively result in an unreliable and incomplete narrative. Extensive revisions with additional experimentation would be necessary for reconsideration.

Major comments:
1. In multiple panels, such as Figure 1c-e, the statistical analysis uses a standard t-test despite the markedly unequal sample sizes of the groups. To account for potential heterogeneity in variance, a Wilcoxon test would be more appropriate. More generally, it is advisable to increase the number of samples (n) in the group with fewer samples.
2. Echocardiographic evaluation of cardiac function should also include the BATectomy group to comprehensively assess the functional impact of BAT on post-MI cardiac remodeling.
3. Fibrosis data in Figure 1f (and also in other panels throughout the paper) are derived from only two tissue sections per heart, which may not represent the full infarct region. Standard protocols typically involve serial sectioning from the ligation site to the apex, with quantitative analysis across multiple sections (typically 4-5 sections per heart).
4. In Figure 1f, please report p-value between the Control and BATectomy groups to support the claim that BAT is essential for mitigating post-MI fibrosis. Additionally, the sample size for the cold-BAT group appears insufficient to draw robust conclusions. Additionally, the claim of an “additional benefit” from cold-exposed BAT grafting lacks statistical support, as no direct comparison (e.g., p-value) between the BAT and cold-BAT groups is provided.
5. In Figure 2c, a much higher resolution is needed.
6. In Figure 2g, CD68 is a cell surface marker, yet the signals appear throughout the cell except for the nucleus. The staining should be refined to accurately reflect CD68's localization. In Figure 2h, identifying GFP+ vesicles within macrophages based solely on morphology is insufficient. Additional evidence should be provided to reveal their identity.
7. The study attributes the anti-inflammatory effects of MDVs to ATP synthase content and metabolic reprogramming of macrophages. However, the possibility of other cargo (e.g., mitochondrial DNA, lipids, or ROS) contributing to macrophage polarization remains unexplored. Additionally, loss-of-function experiments (e.g., ATP synthase inhibition) may further clarify the mechanism.
8. In Figure 3, the authors propose that BAT-derived MDVs harboring mitochondrial complex V is a key driver of cardiac repair post-MI, with MDVs transferred to infarct regions via EVs. However, several points require further validation: a. While the authors claimed that only BAT MDVs with complex V enhance repair, their data show that WAT MDVs also contain ATP5A (a complex V subunit) yet lack efficacy in reducing fibrosis. This inconsistency raises questions about whether complex V components alone are sufficient for repair or if other MDV-associated factors (e.g., metabolic cargoes, scaffold proteins) synergize with complex V. Additional experiments are needed to isolate the role of complex V. b. Figure 3e shows minimal ATP5A in EVs, conflicting with the claim that MDVs transfer complex V. Clarification is needed on how MDVs selectively package complex V into EVs if ATP5A levels are low. c. The analysis of mitochondrial OXPHOS components in

Figure 3f lacks normalization to total loading protein. Uneven loading within groups (notably the LV-EV MI group) obscures statistical comparisons and biological interpretation.
9. In Figure 4e, the quantitative data appear to reflect variability in the loading control (Actb) rather than meaningful changes in VPS35 protein levels. Notably, the representative blots suggest no clear upregulation of VPS35 in BAT under MI stress, contrary to the authors' claims of time-dependent protein upregulation.
10. In Figure 4j, the labels "1" and "2" on the images lack clarity regarding which parts are the insets. Please provide clarification to specify the insets.
11. In Figure 6b, the fluorescence images are too dark to discern meaningful details.
12. The authors state at line 618 that “the effects of WT and Becn1+/- BAT were notably opposite.” However, in Figure 5d, while the WT BAT transport group shows enhanced repair compared to the control, the Becn1+/- BAT group only exhibits no significant difference from the control. This does not represent “opposite effects” but rather a lack of effect in the Becn1+/- group. Please refine the text to more accurately reflect the observed differences.
13. In multiple panels, the authors report measurements of LV wall thickness, but no differences are shown, and these results are also not discussed in the text. Additionally, since MI surgery typically results in injury primarily around the LAD region, leading to non-uniform damage across the LV wall, it is essential to specify exactly which part of the LV wall was measured. Please clarify the location of the measured regions and address the lack of observed differences in the text.
14. In Figure 6, the authors propose that MDV delivery of bioenergetic units shifts macrophages toward an anti-inflammatory phenotype. However, the evidence provided is predominantly transcriptional. To strengthen this conclusion, please include serial experiments validating macrophage polarization at the protein and cytokine levels.
15. In Figure 7, the data are largely transcriptomic. Validation at the protein level is necessary to strengthen these findings. Additionally, there is insufficient evidence provided regarding the origin of Complex V.
16. The manuscript would benefit from substantial text refinement to enhance clarity and precision. In particular, summary sentences for each part of the results are either missing or improperly formatted.

Reviewer #2

(Remarks to the Author)
Shi and coworkers describe that BAT-derived MDVs, containing mitochondrial membrane components such as OXPHOS proteins (e.g., Complex V), exert a cardioprotective effect. Upon myocardial infarction (MI), cardiac macrophages potentially uptake these MDVs, leading to reduced fibrotic remodeling and an enhanced anti-inflammatory response through augmented reparative cytokine production and OXPHOS rewiring. The findings suggest that physical contact between BAT-MDVs and cardiac macrophages is essential for the cardioprotective effect, with VPS35 and Becn1 proteins facilitating endosomal trafficking.
While the concept is interesting and a lot of data is provided, some analyses lack robustness. One concern regards the analysis of cardiac function and outcome after myocardial infarction. Authors assessed fibrosis (by histology) and left ventricular functon(by echocardiography) at day 4 or 7 after induction of myocardial infarction. Fibroblast activation occurs within the first days after MI, leading to potential changes in Trichom stainings. However, fibroblast maturation, scar formation and conclusion of remodeling takes significantly longer. Specifically in the setting of permanent ligation, the inflammatory phase require longer periods of time as compared to I/R injury. Assessment of scaring and functional outcomes are typically conducted two to fours weeks after MI. In the same line, as remodeling is not completed at day7 after infarction, LV wall thickness is not a good parameter at this timepoint and highly variable (see 1e). Thus interpretation of functional outcome after BAT transplantation is limited.

The following additional points should be addressed:
1. In line 403: How much protein is loaded per well?
2. Page 12, line 328: blue fibrotic area/heart cross-section area…. Should be corrected to "heart cross-section". In addition, details about biological/technical replicates are missing in the legend.
3. In line 403: How much protein is loaded per well?
4. In line 440: TEAB stands for?
5. Line 568 - " Specifically, OXPHOS levels in BAT-EVs increased post-MI, while they decreased in the left ventricle (LV)-EVs (Fig. 3f)" – From figure 3 this point is not clear - please add the quantification of the proteins, picture of whole blot (in supplement), and exposure time as shown in Figure 4, e.
6. Terminology - "under DAMPs stimulation" line 609, is misleading for figure 4 k. Please change or explain separately.
7. In line 612, authors claims that Becn1 regulates VPS35 recruitment to mitochondria, lacks mechanistic detail. Suggestion: To prove this point, Co-IP experiment or PLA experiment could strengthen the conclusion.
8. In line 659, author links MDV uptake to increased OXPHOS and IL-10 production (Fig. 6), the role of Complex V proteins remains indirect and conclusion lacks mechansitic evidence. Suggestion: Use of CRISPR- or SiRNA-mediated (e.g., ATP5A1 knockdown) in macrophages to test whether Complex V components are necessary for the observed findings.
9. In previous study by Rosina et al, 2022 demonstrated that EV-derived from the BAT contain oxidatively damaged mitochondrial components and are primarily destined for degradation to avoid failure of the thermogenic program. PLease discuss. Further, please clarify whether their MDVs represent a distinct subpopulation from those described by Rosina et al., or whether there is functional heterogeneity within BAT-derived vesicles.
10. Appropriate control experiments to BAT pericardial transplantation is lacking. Authors should at least transplant PBS or carrier solutions, or WAT.
11. It is unclear to this reviewer why both male and female mice were used as BAT donors? Sex differences in brown adipose tissue are well documented. This could include adipose tissue mitochondrial function and metabolic flexibility (https://www.nature.com/articles/s41366-021-00843-0).

Specific comments to figures:

1c: It is uncommon to see such massive LV dilation, even in permanent ligation. Further, parts of the valves are visualized on the baseline images, suggesting that echocardiography measurements were not carried out with the same imaging window and angle.
1d: Baseline data is not quantified nor shown.
2: Author did not quantify MDVs. It would be more informative if the author quantifies the MDVs through flow cytometer. Additionally, the images look better with proximity ligation assay than IHF staining. To confirm that the MDVs rescue fibrosis, the author should stain for the Masson staining on the heart section post MI.
2c/2g: Labeling of images could be improved. Control antibody stainings are missing.
2g: The uptake of BAT(Zs-green) in macrophages is not convincing
3d: Fibrosis quantification at day 4 after infarction does not make any sense. This readout is conducted way too early in the course of postinfarct remodeling. Authors showed in figure 1f that WAT increases fibrosis, resulting in larger fibrotic area than in controls. Therefore, authors should compare BAT-MDV also to controls (not only to WAT-MDV).
5b, the immunostaining images should be replaced with higher magnification image that corresponds well with the quantification (in Fig 5c).
6: Label y axis in plot a; KD equivalent to Becn1+/- mice in plot c and j, not clear in image legend? Please check. Additionally, the bulk analysis was derived from which mouse strain? WT-MI, WT- MI+BAT transplant?
Extended Data Fig. 3: The uptake of BAT(Zs-green) in macrophages is not convincing. This could in part be autofluorescence. Control stainings are lacking. Further, this reviewer suggests quantification of BAT uptake by flow cytometry, i.e. quantification of ZS-green+ macrophages.
Extended Data Fig. 8: “transient macrophages” is not a common denominator of macrophage populations. Recent scRNA-sequencing dataset of cardiac myeloid populations in mouse myocardial infarction models have provided clear nomenclature for immune cells, e.g. inflammatory/antigen-presenting/etc..
Extended Data Fig. 10: Data annotation and representation could be improved.

Reviewer #3

(Remarks to the Author)
Xiang and colleagues identify a novel role for brown adipose tissue (BAT) in cardioprotection through the release of mitochondria-derived vesicles (MDVs) following myocardial infarction (MI). These MDVs, enriched in mitochondrial membrane components, are taken up by cardiac macrophages, where they promote an anti-inflammatory and reparative phenotype. The authors propose that MDVs is generated by mitochondrial recruitment of VPS35, a process dependent on Becn1. Loss of Becn1 disrupts MDV biogenesis and abolishes BAT's protective effect. Proteomic profiling reveals enrichment of electron transport chain Complex V proteins in MDVs, which enhances oxidative phosphorylation and cytokine production in recipient macrophages. Therapeutically, administration of BAT-derived MDVs attenuated post-MI fibrotic remodeling. While the manuscript explores an intriguing interorgan communication mechanism, several methodological and interpretative issues need to be addressed to strengthen the work.

Major comments:
(1) The Authors suggest an interorgan BAT-heart crosstalk via new mediator MDVs. BAT has the beneficial effects on cardiovascular diseases, such as coronary artery disease, heart failure, and hypertension (Nat. Med. 2021,27,58–65). The concept about mitochondrial vesicle trafficking and intercellular mitochondrial exchange, has also been previously described (Cell Metab. 2021;33(9):1853-1868, Cell Metab. 2022; 34(10): 1499-1513, Cell Transplant. 2023:32:9636897221148457). These previous reports limit the conceptual novelty of this manuscript. The authors fail to establish how BAT-derived MDVs uniquely advance this field.

(2) From the therapeutic view, does using MDVs offer a distinct and necessary advantage over using engineered EVs, or direct mitochondrial transfer, or BAT? Why did the authors choose MDVs over the others? A direct comparison of these strategies—including delivery routes (e.g., intravenous vs. tissue transplantation)—would significantly enhance the translational relevance the utility of MDVs.

(3) In mouse models of myocardial infarction (MI), researchers typically assess cardiac fibrosis and pathological hypertrophic remodeling at 2 to 6 weeks post-MI. At 1–3 days post MI, cardiomyocyte death, acute inflammation; at 1-week, inflammatory response, beginning of fibrotic deposition, at 2-4 weeks, active fibrotic remodeling and hypertrophy, functional decline, at 6–8 weeks, chronic remodeling, scar stabilization, ventricular dilation to heart failure (Circulation Research 2016;119(1):91-112, PMID: 27340270). In this study, the authors assessed the fibrosis and hypertrophic remodeling at very early timepoints (day 4–7) post MI, which are not accurate for claiming the BAT improvement in remodeling phenotype. The authors should provide late stage assessment of fibrosis and cardiac hypertrophy. In addition, aSMA, Periostin ColI, Col III, and ANP, BNP, myh7, which are all markers for fibrotic and hypertrophic remodeling, should be evaluated.

(4) Previous report indicates that stressed adipocytes release small extracellular vesicles (sEV), which contain respiration-competent and enter circulation. sEV are taken up by cardiomyocytes and subsequently protect cardiomyocytes and ischemic heart injury (Cell Metab. 2021;33(9):1853-1868). In this study, the authors found the MDVs are mainly delivered to macrophage and protect the cardiac remodeling, in which MDVs are not taken up by cardiomyocytes. Although the authors used the BAT mitGFP mice to label BAT mitochondria, did the authors check the border zone or remote zone? The fig2g, Extent fig3d, are not clear enough to exclude the MDVs’ transferring to other cell types, (such as neutrophils, fibroblasts), which may contribute to the cardiac protection as well. The authors may conduct FACs analysis of mtGFP+ populations across cardiac cell types to elucidate the cell types taken up by the MDVs.

(5) Myocardial infarction reduces BAT mass (Fig1a) and stresses the BAT. The authors transplant donor unstressed BAT to recipient mice with MI to find out the roles of MDVs. Did the authors also do MI surgery in BATmtGFP mice and check if stressed BAT delivery its own MDVs to the heart in the same mice? It seems that BAT undergoing stress has better protective roles, like cold-BAT reduces more fibrosis than BAT transplantation in Fig1f. Whether MI stressed BAT may function as well when transplanting to MI mice?
(6) Brown adipocytes tend to eject their mitochondria under stress for the interorgan mechanisms. But how MI injured heart initiates the signaling and impacts BAT metabolism and mitochondria as well as the biogenesis mechanism of MDVs are unknown in the manuscript.

(7) There is missing cardiac functon(echocardiography) data in Fig3d, which is important for claiming the MDVs protection, although the authors present fibrosis and remodeling data.
(8) How long the MDVs stay or last in the heart or macrophage?
In Fig2h, round-shaped GFP+ vesicles appeared in the macrophage cytoplasm, and some were fused with recipient macrophage mitochondria, what's the percentage of fused or cytoplasmic MDVs? Whether they have the same function? How fused with recipient macrophage mitochondria function?
(9) BECN1 increased in heart DAMPs-treated brown adipocytes (Fig. 4g), The use of systemic Becn1-deficient mice raises concerns about cell-type specificity. Effects observed may not reflect BAT-specific mechanisms. A BAT-specific Becn1 knockout model would more convincingly support the proposed mechanism. In addition, Extended Data 7ab, the heart functon(EF% and FS%) actually exhibited no changes, indicating Becn1 may not attribute for the protection in this time point. The authors may assess the cardiac function at late stage.

Minor concerns:
(1) Functional measurements are absent in Fig. 5e and Extended Fig. 7i. These should be included to support the therapeutic claims of MDV administration.
(2) In Fig. 6c, only IL-4 shows changes. It would be informative to assess other M2-associated markers, such as IL-10 and Arg1, to confirm polarization phenotype.
(3) Fig. 2g lacks sham controls for comparison
（4） Immunofluorescence images throughout the manuscript are of relatively low resolution.
（5） In Fig. 4e, ACTB levels are dramatically reduced post-MI, raising concern about loading controls. This should be clarified or alternative loading controls used.

Reviewer #4

(Remarks to the Author)
This manuscript by Shi and co-workers describe their study with the aim to demonstrate the impact of BAT on the heart post-MI. The authors show that MI stimulates the release of BAT-derived mitochondria-derived vesicles that influence the inflammatory status of cardiac macrophages. The MDV cargo, dominantly OXPHOS proteins, mediates its cardioprotective effects. The BAT-heart connection, taken into considerations the BATtokines: FGF21, IL6, NRG4, 12,13-diHOME and BMP3b are a timely topic to understand and appreciate interorgan communication. Especially if BAT-derived exosomes delivering iNOS to sites of ischemia and as BAT is rich in mitochondria which they release under stress.

However, there are some issues that need to be addressed. One of them is that it would be appreciated if a native English speaker would be able to read the manuscript and if the material and methods are not organized in a more logical manner.

Other questions are:

Line 102: The authors write that data were excluded only when the mice died during surgery or before the time points of analysis. The first argument is acceptable, but during the course of the experiment is very relevant and excluding may introduce a bias.

Line 463-464: The authors write: suggested a link between cardiac injury and BAT reduction. This can be just a coincidence.

Figure 1D&E: The number of animals in the MI group vs BAT transplanted animals differs significantly which may skew the data. What is the rationale behind this discrepancy? Furthermore, an EF of less than 10% is extremely low, which does not translate to the thickness of the wall. Can the authors explain this? Furthermore, the LV wall thickness is much thicker in F compared to E. Finally, it is not clear what Base to Apex means in figure 1F.

It is not clear from the data presented if the macrophage co-localization of BAT-MDVs is an active process or just because macrophage are programmed to clear damage areas.

The IF images presented in figure 2 are difficult to appreciate as the resolution is not very good. In 2B&D, the BATzgreen is difficult to see, and the same problem is for 2C.

Line 551-553: If the numbers of MDVs were lower in WAT compared to BAT, is it the number that makes the difference of the content? Especially if (line 558) equal amount of tissue is used and not number of MDVs

Figure 3D: as previously mentioned, it is not clear what the Base -> apex refers to. Furthermore, in the comparison the

control is missing to appreciate the impact of MDVs on fibrosis. The amount of fibrosis 4 days after MI is quite intensive. Is this true fibrosis or scar tissue? The process normally takes some more time. Again, the control without injection is missing. Figure 3G is not informative. How did the authors distinguish LV-EVs from BAT-EVs post MI?

The rationale to check the genes in figure 4a is not clear. What does Id2 (line 579) tell us?

Line 602-604: If autophagy is inhibited, is MDV release hampered? Is Becn1 involved in the MDV release or, more likely, in a biological response linked to autophagy? How does this impact cardioprotection, as a direct relation is not clear.

Figure 4E, the increase in VPS35 is only due to a difference in beta actin. This is therefore not the correct housekeeping gene to correlate expression to and should be corrected. Figure 4I has the same bias.

The date presented in figure 5B is not visible. Again figure D and E are difficult to appreciate, is there no cardioprotection because the MDVs now are not taken up by the cells they normally would modify? As the mechanism of action is not clear, figure 6 is difficult to appreciate.

Figure 7 feels a bit out of scope of the paper. If in the graphical abstract OXPHOS is key, more mechanistic validation is needed to support that finding.

Version 1:

Reviewer comments:

Reviewer #1

(Remarks to the Author)
The authors have made substantial revisions to the manuscript, which have greatly strengthened the paper. However, a few points still require attention.

1. The magnification and clarity of Figure 2c, d and R3 are insufficient to convincingly demonstrate the localization of ZsGreen. The images barely allow for the identification of individual cells, let alone the resolution of subcellular structures. They are certainly not adequate for distinguishing mitochondrial membrane structures. Please do provide images with at least enough resolution to distinguish individual mitochondria and improve the image brightness.

2. The colors of α-actinin and CD68 in Figure 2g are too similar to distinguish.

3. Figures 3d, e, and f indicate that equal amounts of MDVs derived from WAT and BAT exhibit identical promoting effects on the repair of cardiac structure and function following myocardial infarction. This should be further discussed in the text.

4. The authors used both Masson's trichrome and Sirius Red staining methods to demonstrate cardiac fibrosis levels. However, only one statistical graph is provided (Fig. 1e, f; Fig. 3f, g; Fig. 5e, g). Please provide the statistical results for both staining methods.

Reviewer #2

(Remarks to the Author)
The authors have provided additional data, increasing time points and n-numbers. Overall this makes the data more robust.

Reviewer #3

(Remarks to the Author)
The authors have made significant efforts to improve the manuscript. The revised version has been substantially enhanced, and I have no further comments. Congratulations.

Open Access This Peer Review File is licensed under a Creative Commons Attribution 4.0 International License, which permits use, sharing, adaptation, distribution and reproduction in any medium or format, as long as you give appropriate credit to the original author(s) and the source, provide a link to the Creative Commons license, and indicate if changes were made.
In cases where reviewers are anonymous, credit should be given to 'Anonymous Referee' and the source.
The images or other third party material in this Peer Review File are included in the article's Creative Commons license, unless indicated otherwise in a credit line to the material. If material is not included in the article's Creative Commons license and your intended use is not permitted by statutory regulation or exceeds the permitted use, you will need to obtain permission directly from the copyright holder.
To view a copy of this license, visit https://creativecommons.org/licenses/by/4.0/

Peer Review File

Response to reviewers for "**Mitochondria-derived-vesicles with bioenergetic units from brown adipose tissue attenuate cardiac remodeling post-myocardial infarction**" by "Tingting Shi, …, Yaozu Xiang"

REVIEWER #1 2

REVIEWER #2 41

REVIEWER #3 82

REVIEWER #4 116

Reviewer #1 Comments

This study explores the therapeutic potential of brown adipose tissue (BAT)-derived mitochondria-derived vesicles (MDVs) in mitigating cardiac remodeling post-myocardial infarction (MI). While the proposed mechanism is intriguing, the current manuscript has multiple critical gaps that undermine the validity of the conclusions. In particular, the lack of key experimental details, insufficient validation of proposed mechanisms, and questionable data interpretation collectively result in an unreliable and incomplete narrative. Extensive revisions with additional experimentation would be necessary for reconsideration.

Response:

We extend our sincere gratitude for your meticulous review and insightful comments. In revising the work, we have undertaken a substantial experimental and textual revision aimed at addressing the key concerns you raised, (i) refined the methods in mouse cardiac phenotype experiments and molecular experiment, (ii) added more evidence to further validate that MDV transfer from BAT to the infarcted heart, and (iii) explored mechanistically of how MDVs modulate macrophage function.

(i) Animal phenotyping and cardiac outcome experiments

We repeated and expanded all key in vivo experiments using a refined mid-LAD MI model, balanced and increased sample sizes, and extended follow-up to 28 days to get the whole profile the cardiac remodeling. Serial echocardiography (baseline, days 7, 14, 28) and multi-level Masson and Sirius Red staining now provide a more robust assessment of post-MI remodeling.

(ii) Validation of MDV transfer from BAT to the heart

We strengthened evidence for MDV transfer using complementary imaging, refined protocols to reduce non-specific signals, and flow cytometry to further validate the phenomenon. High-resolution confocal of $BAT^{ZsGreen}$ and BAT^{mtGFP} recipients now show BATosome vesicles within the infarct zone that co-localize with mitochondrial membrane markers (TOMM20, cytochrome c, ATP5A1), consistent with MDV identity. Flow cytometry of hearts from $BAT^{ZsGreen}$ recipients demonstrates that the majority of

ZsGreen$^+$ immune cells are CD11b$^+$F4/80$^+$ macrophages, confirming macrophages as the main MDV recipients in vivo.

(iii) Mechanistic study of MDV effects on macrophage function

To elucidate the functions of complex V and its major unit ATP5A1 in macrophage polarization, we added in vitro experiments in DAMP-activated macrophages treated with MDVs. WT BAT-MDVs reprogram the canonical iNOS/ARG1 “molecular switch” toward an iNOS^{low}ARG1^{high} state, accompanied by higher IL-4 secretion. Loss-of-function experiments show that oligomycin-treated MDVs with inhibited Complex V activity, and MDVs from *Becn1*$^{+/-}$ BAT, largely lose these effects, whereas macrophage ATP5A1 overexpression partially recapitulates the pro-reparative phenotype. Together, these data provide multi-level support for the concept that BAT-derived MDVs deliver bioenergetic cargo that promotes a pro-reparative macrophage program.

Major comments:

1. In multiple panels, such as Figure 1c-e, the statistical analysis uses a standard t-test despite the markedly unequal sample sizes of the groups. To account for potential heterogeneity in variance, a Wilcoxon test would be more appropriate. More generally, it is advisable to increase the number of samples (n) in the group with fewer samples.

Response:

We appreciate this important point. We addressed the concern in two ways.

1) We conducted the Wilcoxon rank-sum test (Mann-Whitney U test) for comparison between two independent groups with unequal sample sizes. We fully agree that the normality test and the homogeneity of variances test lack power when n is small. The results of the Wilcoxon test support the conclusion that BAT transplantation significantly improved cardiac contractility and reduced post-MI fibrosis (Figure R1).

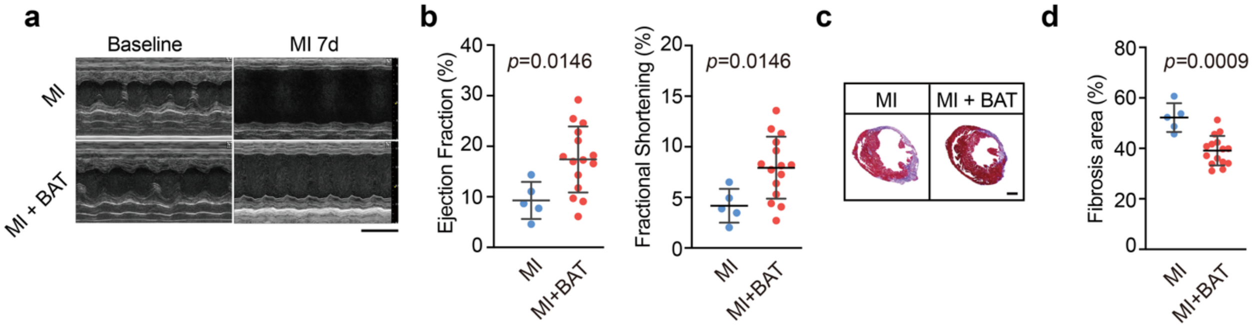

Fig. R1 a, b, Representative M-mode echocardiography and quantitative results of BAT-receiving mice at baseline and MI 7d (mean±s.d., n=5(MI), 15(MI+BAT), Mann-Whitney U test). Horizontal scale bar: 250ms. **c, d**, Representative Masson staining images and quantitative fibrosis percentage of BAT-receiving mice at MI 7d (mean±s.d., n=5(MI), 15(MI+BAT), Mann-Whitney U test). Scale bar: 1mm.

2) We re-conducted the BAT transplantation phenotype experiment with increased sample size. We increased the sample size to n=7 per group across all five groups: MI, MI-BAT (BATectomy performed 1 week before MI), MI+BAT (MI with BAT transplantation), MI+WAT (MI with WAT transplantation), and MI+cold BAT (MI with cold-exposed BAT transplantation). Also, we extended the outcome

assessments to 28 days post-MI, considering the early assessment at day 4-7 after MI induction may not capture the complete remodeling process. Serial echocardiography at baseline, day 7, 14, and 28 post-MI induction was performed to assess cardiac functions. Hearts were harvested at day 28 for multiple spaced sections, then fibrotic stains (Masson staining and Sirius Red staining together to define the fibrotic area).
Additionally, we applied the refined LAD ligation technique that induced moderate MI to eliminate MI-related mortality and thereby minimize survival bias.

In the revised manuscript, statistical methods were as follows. Data normality and variance were assessed using the Shapiro-Wilk test and F-test. Gaussian data are analyzed by Student's t-test (two-group comparisons) or ANOVA test followed by the Bonferroni method (multi-group comparisons). Non-Gaussian data are analyzed by Wilcoxon rank-sum (two-group comparisons) or Kruskal-Wallis with Dunn's post-hoc (multi-group comparisons). The exact tests used are specified in the figure legends, and adjusted P values are reported.

We evaluated the impact of BAT manipulations on post-MI remodeling. BATectomy worsened cardiac function and increased fibrosis relative to MI controls. In contrast, BAT transplantation improved both functional and histological outcomes, whereas WAT transplantation produced no detectable change (Fig. R2). Notably, BAT from cold-exposed donors conferred additional benefit, yielding higher ejection fraction (EF%) and fraction shortening (FS%) than BAT from room-temperature housed donors (EF%, p=0.01; FS%, p=0.0138; Fig. R2c). Together, these data indicate that BAT, particularly cold-activated BAT, provides cardioprotection that WAT can not reproduce.

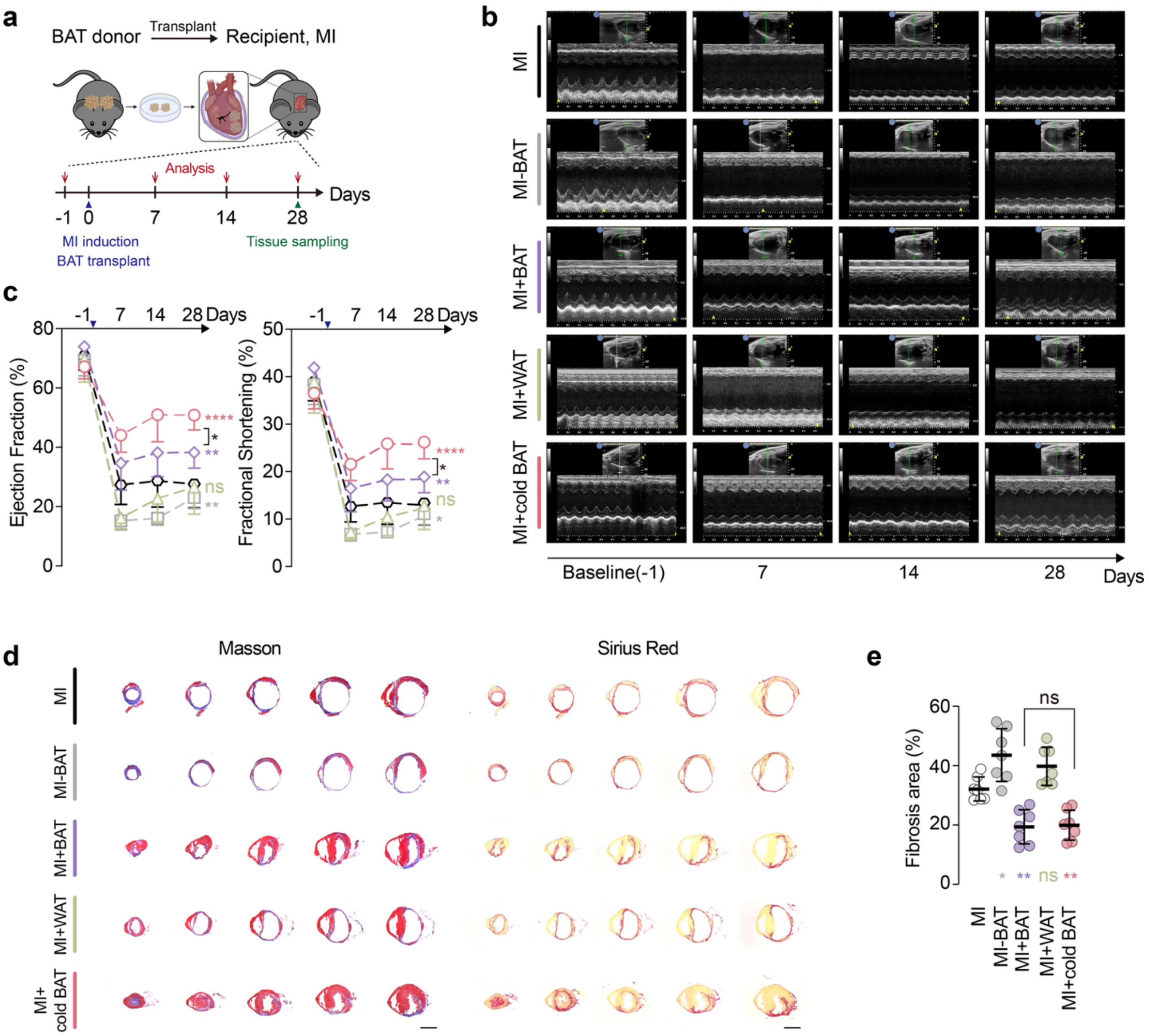

Fig. R2 a, Experimental timeline for MI induction, adipose tissue pericardial transplantation, and outcome assessments. Echocardiography was performed at baseline (day −1) and on days 7, 14, and 28. Hearts were harvested on day 28 for Masson's trichrome and Sirius Red staining. **b, c,** Representative B-mode and M-mode echocardiography of BAT-receiving mice at baseline and days 7, 14, and 28 (**b**). Ejection fraction and fractional shortening were quantified (**d**) (mean±s.d., n=7, repeated two-way ANOVA and Holm-Sidak post hoc, *$p < 0.05$, **$p < 0.01$, ****$p < 0.0001$). **d, e,** Representative Masson and Sirius Red staining images of mouse hearts at day 28. Fibrosis percentage was quantified (mean±s.d., n=7, One-way ANOVA test followed by

Bonferroni method, *p < 0.05, **p < 0.01). Scale bar: 2mm. **The above new data are included in Fig. 1 in the revised manuscript.**

Manuscript edits: “Results”

Pericardial BAT transplantation mitigated post-MI systolic decline. Across serial echocardiography at days 7, 14, and 28, BAT-treated mice maintained higher ejection fraction and fractional shortening than MI controls, indicating sustained preservation of contractile performance (Fig. 1c, d). Concordant structural benefits were observed on Masson’s trichrome and Picrosirius Red staining, which showed a smaller fibrotic area and better myocardial preservation in the BAT group (Fig. 1e, f). Consistently, BAT-deficient mice (MI-BAT, iBAT excision 1 week before MI) exhibited significantly lower EF%/FS% and greater fibrosis than MI controls, supporting an essential role of endogenous BAT in limiting post-MI remodeling (Fig. 1d, f).

As further validation, we compared different adipose grafts. White adipose tissue (WAT) and BAT arise from distinct adipocyte lineages and exert divergent metabolic and cardiovascular effects[1]. WAT transplantation did not modify MI-induced fibrosis or systolic function, whereas BAT transplantation reduced scar burden and preserved contractile performance (Fig. 1c-f), indicating BAT-specific cardioprotection. To test whether BAT activation augments this benefit, donor mice were cold-exposed at 4 °C for 14 days before grafting. MI+cold BAT mice showed higher EF% and FS% than MI+BAT mice, while fibrosis burden remained comparable between the two groups (Fig. 1d,f, f). Collectively, these findings support a BAT-specific, thermogenesis-enhanced cardioprotective effect on early post-MI remodeling.

2. Echocardiographic evaluation of cardiac function should also include the BATectomy group to comprehensively assess the functional impact of BAT on post-MI cardiac remodeling.

Response:

Thank you for this constructive suggestion. We agree that including all cohorts in the functional evaluation is important for a comprehensive assessment of BAT's role in post-MI remodeling. In the revision, we therefore added serial echocardiography for the BATectomy, MI+WAT, and MI+cold BAT groups alongside MI and MI+BAT cohorts (Fig. R2a-c).

3. Fibrosis data in Figure 1f (and also in other panels throughout the paper) are derived from only two tissue sections per heart, which may not represent the full infarct region. Standard protocols typically involve serial sectioning from the ligation site to the apex, with quantitative analysis across multiple sections (typically 4-5 sections per heart).

Response:

We appreciate your important comment regarding the sampling strategy for fibrosis quantification. We apologize for the lack of clarity in our original description. In fact, fibrosis in Figure 1f and all related panels was derived from 5-6 sections per heart. Each heart was serially sectioned from the apex toward the ligation site, and fibrosis was quantified on multiple evenly spaced sections (5-6 sections per heart). For each animal, the mean fibrosis percentage across these serial sections was used for analysis, in line with standard protocols. The images presented in the figures are representative sections, whereas the numerical data reflect the averaged values from the entire series. To avoid further misunderstanding, we have revised the Methods to explicitly describe this serial-sectioning approach. Also, in the revised manuscript, we displayed section-by-section images and applied both Masson's trichrome staining and Picrosirius Red staining on adjacent sections. Masson's trichrome staining was used to quantify scar area and fibrosis percentage, while adjacent sections underwent Picrosirius Red staining to assess collagen deposition and fiber architecture, thereby providing a comprehensive evaluation of myocardial fibrosis extent. The per-heart mean fibrosis percentage was calculated as the mean fibrotic area across 5-6 sections for each heart (Fig. R2d, e).

Manuscript edits: "Methods"

Masson trichrome and Sirius Red staining

Fibrotic area appeared blue on Masson-stained sections and Sirius Red–positive on adjacent sections. For each level, fibrosis area was defined as the region corresponding to Masson-blue and Sirius Red–positive signals, and fibrosis percentage was calculated as fibrotic area divided by total cross-sectional area. For each mouse, fibrosis% was taken as the average across 5–6 serial section pairs from apex to ligation level.

4. In Figure 1f, please report p-value between the Control and BATectomy groups to support the claim that BAT is essential for mitigating post-MI fibrosis. Additionally, the sample size for the cold-BAT group appears insufficient to draw robust conclusions. Additionally, the claim of an "additional benefit" from cold-exposed BAT grafting lacks statistical support, as no direct comparison (e.g., p-value) between the BAT and cold-BAT groups is provided.

Response:

Thank you for raising this important point on the statistical analysis.

In the revised manuscript, where we re-conducted the experiments with increased and balanced sample size for all groups, we now explicitly report the P value for the comparison between Control (MI) and BATectomy (MI-BAT) groups (Fig. R2c, e) to substantiate the conclusion that endogenous BAT contributes to limiting post-MI fibrosis. The decrease in EF% and FS% was significant in the MI-BAT group compared to MI controls ($p=0.0073$, 0.0138, two-way ANOVA and Holm-Sidak post hoc). The fibrosis also showed a significant progression when BAT was deficient ($p=0.0181$, One-way ANOVA test followed by Bonferroni method).

We now also provide a direct statistical comparison between the BAT and cold-exposed BAT transplantation groups (Fig. R2c, e). MI+cold BAT mice exhibited higher EF% and FS% than MI+BAT mice ($p=0.010$, 0.0138, two-way ANOVA and Holm-Sidak post hoc), whereas fibrosis burden did not differ significantly between the two groups.

These data support a key role of endogenous BAT in restraining post-MI fibrosis and systolic impairment. Cold-activated BAT provides an extra functional benefit but no further antifibrotic effect.

5. In Figure 2c, a much higher resolution is needed.

Response:

Thank you for pointing out the image quality problems. In the revised manuscript, we have replaced Figure 2c with high-resolution confocal images exported at the original acquisition resolution and added zoomed-in insets highlighting the regions of interest.

To further characterize the nature of BATosomes, we performed additional co-localization immunohistofluorescence analysis of mitochondrial markers with BATosome signal. BAT grafts were obtained from B6-G/R mice, which express ZsGreen ubiquitously, and transplanted pericardially into MI recipients. Recipient hearts were harvested on day 4 after MI and imaged. ZsGreen-positive BATosomes in the myocardium showed extensive co-localization with the outer mitochondrial membrane marker TOMM20, cytochrome c (intermembrane/inner membrane), and the Complex V subunit ATP5A1, but only rare overlap with the mitochondrial matrix enzyme PDH (Fig. R3). These data support the interpretation that BATosomes are mitochondria-derived-vesicles enriched in mitochondrial membranes and intermembrane/inner membrane components, with minimal matrix content.

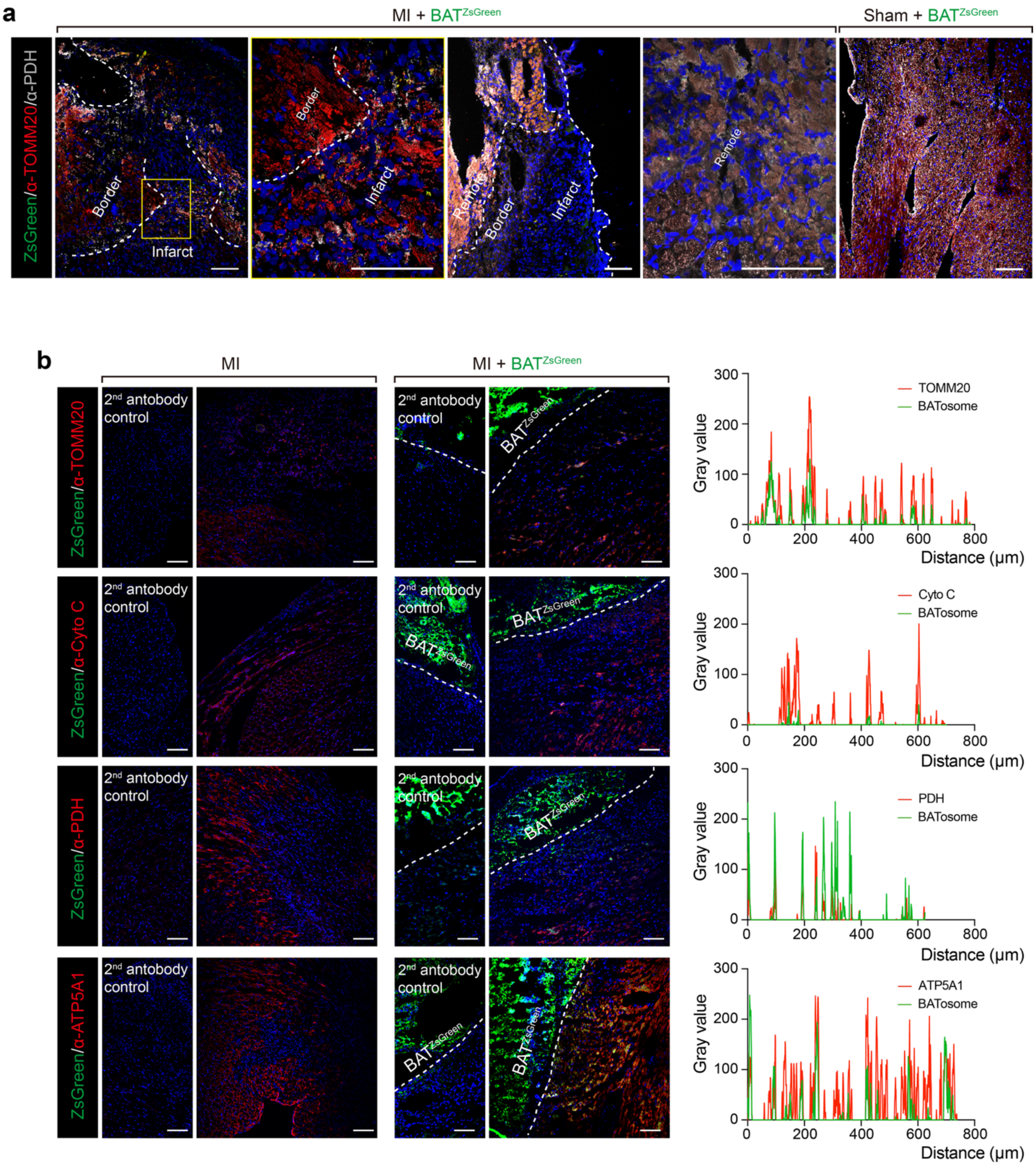

Fig. R3 a, Representative IHC images of the myocardium of fluorescent BAT-receiving mice at MI 4d, with ZsGreen labels (green), stained with TOMM20 (red) and PDH (silver). Scale bar: 100μm. **b,** Representative IHC images of the myocardium of fluorescent BAT-receiving mice at MI 4d, with ZsGreen labels (green), stained with TOMM20 or Cyto C or PDH or ATP5A1 (red). Colocalization analysis of ZsGreen signals

(green curve) and TOMM20 or Cyto C or PDH or ATP5A1 (red curve) shows the distribution of signal across the image. **The above new data are included in Fig. 2 and Extended Data Fig. 2 in the revised manuscript.**

6. In Figure 2g, CD68 is a cell surface marker, yet the signals appear throughout the cell except for the nucleus. The staining should be refined to accurately reflect CD68's localization. In Figure 2h, identifying GFP+ vesicles within macrophages based solely on morphology is insufficient. Additional evidence should be provided to reveal their identity.

Response:

We are grateful for raising these important points regarding macrophage identification.

CD68 is widely used as a macrophage marker and is predominantly localized to lysosomal and endosomal membranes rather than strictly confined to the plasma membrane (PMID: 21572087). CD68 is a type I transmembrane glycoprotein of the LAMP family that is predominantly localized to lysosomal and endosomal compartments in macrophages (PMID: 27869795). Accordingly, a perinuclear/vesicular cytoplasmic pattern is commonly observed and is consistent with published reports.

[FIGURE REDACTED]

Reference: Cell, 2020. PMID: 32937105.

[FIGURE REDACTED]

Reference: Sci Adv, 2020. PMID: 33310846.

[FIGURE REDACTED]

Reference: J Exp Med 2009. PMID: 19139167.

In the revised manuscript, we optimized the CD68 immunofluorescence staining by reducing the permeabilization time and antibody concentration to achieve clearer and more specific labeling. Consistent with previous results, CD68 macrophages exhibit a predominantly cytoplasmic or perinuclear pattern rather than a continuous cell-surface distribution both in cultured macrophages and in heart tissue (Fig. R4a, b). Secondary-only controls have been included to demonstrate minimal nonspecific background.

We transplanted BAT from ZsGreen-ubiquitous reporter mice or from donor mice in which BAT mitochondria were selectively labeled with AAV-mtGFP into the pericardial space of MI recipient mice. Confocal imaging demonstrated that $ZsGreen^{+}$/$mtGFP^{+}$ BATosomes are distributed throughout the infarct area, which is predominantly composed of neutrophils, macrophages, and fibroblasts. Co-localization analysis revealed that BATosome signals largely overlap with $CD68^{+}$ macrophages, with only occasional co-localization with MPO^{+} neutrophils, and virtually no overlap with α-SMA^{+} fibroblasts (Extended Data Fig. 2c, d; Fig. R4b). These data support that cardiac macrophages are the major in situ recipients of BATosomes within the infarcted myocardium.

In addition to addressing the recipient cell identity, we performed flow cytometric analysis to assess the cell-type specificity of BATosome uptake in vivo. BAT was harvested from ZsGreen-ubiquitous donor mice and transplanted pericardially into non-fluorescent MI recipients. Hearts were collected at day 4 after MI, enzymatically dissociated, and immune cells were analyzed for ZsGreen positivity. The majority of $ZsGreen^{+}$ immune cells were macrophages (approximately 60-70%), whereas the remaining $ZsGreen^{+}$ cells fell within monocyte- or dendritic cell-like populations. These data support cardiac macrophages as the predominant recipients of BATosomes in vivo, while indicating that other myeloid subsets may also take up BAT-derived vesicles (Fig. R5).

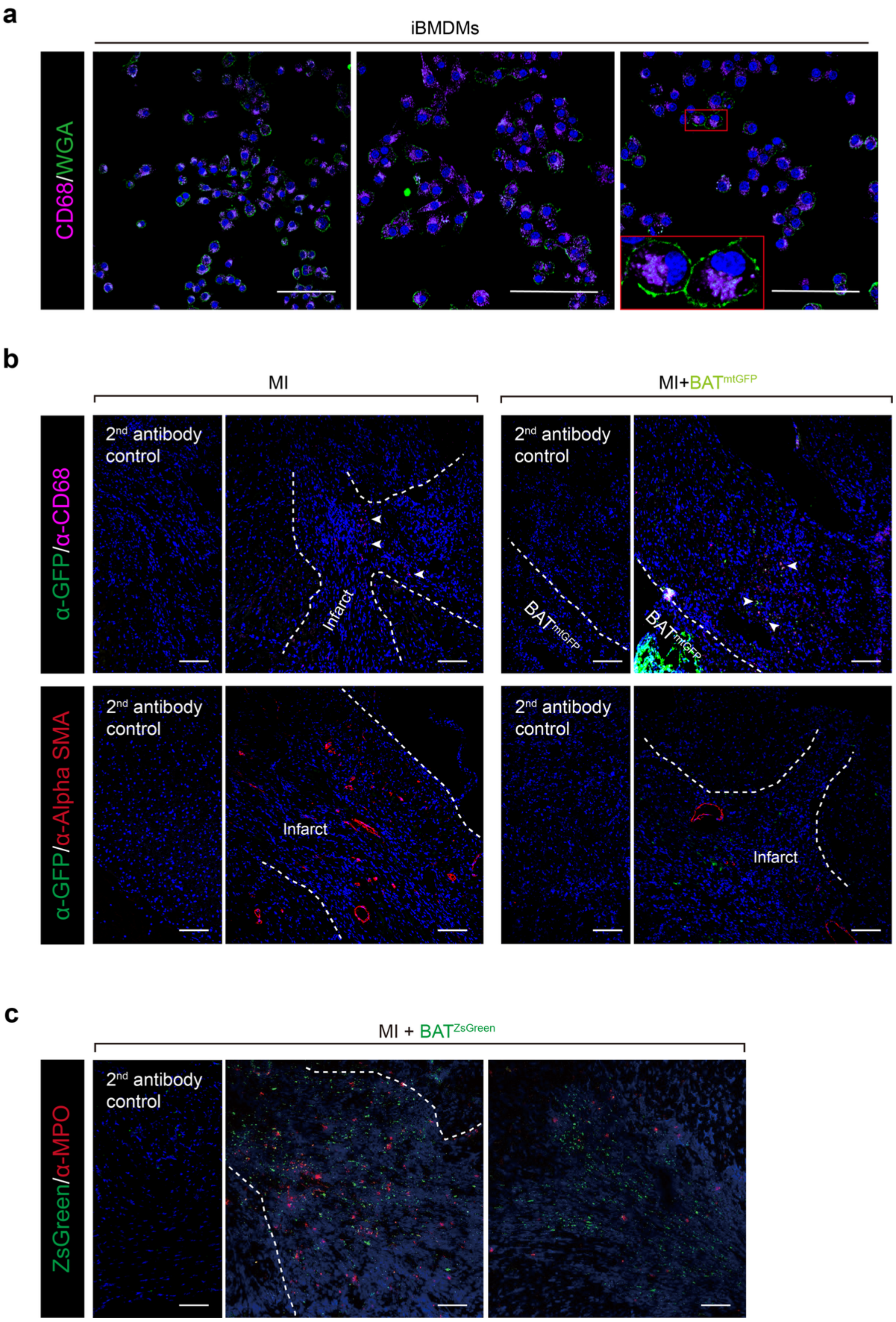

Fig. R4 a, Representative ICC images of iBMDM cells, stained for CD68 (magenta), WGA (green), and DAPI (blue). Scale bar: 10μm. **b,** Immunofluorescence validation of

mtGFP signal in infarcted hearts of BAT^{mtGFP}-receiving mice. Sections were stained for GFP (green) with CD68 (macrophages) or α-SMA (fibroblasts) (red). Scale bar: 100μm. **c,** Representative confocal images of the left ventricles in BAT^{ZsGreen}-receiving mice at MI 4d, stained for MPO (neutrophil, red) and nuclei (DAPI, blue). Scale bar: 100μm. **The above new data are included in Extended Data Fig. 2 and Extended Data Fig. 3 in the revised manuscript.**

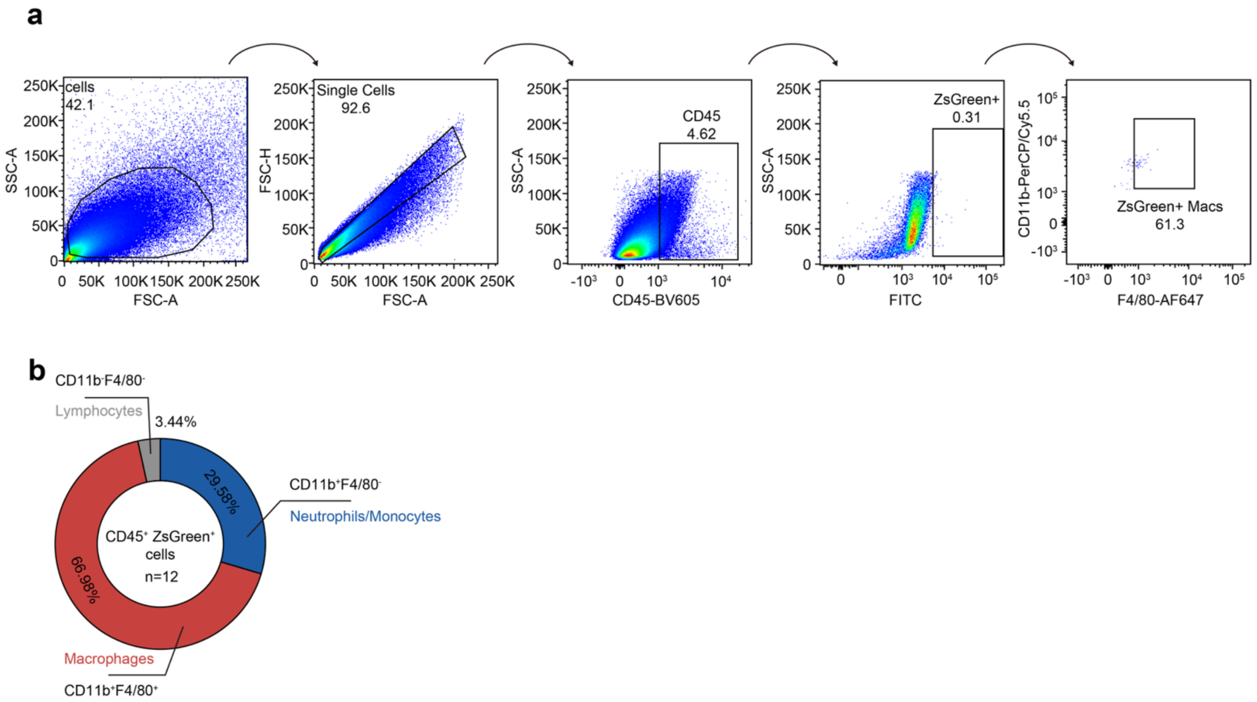

Fig. R5 a, Flow-cytometric gating strategy for identifying BATosome-recipient immune cells in infarcted hearts from BAT^{ZsGreen}-receiving mice at MI 4d. After debris exclusion and singlets selection, leukocytes were gated as CD45^{+} cells, followed by ZsGreen^{+} events and CD11b^{+}F4/80^{+} macrophages within the CD45^{+}ZsGreen^{+} population. **b**, Quantification of immune cell subsets among ZsGreen^{+}CD45^{+} cells in infarcted hearts from BAT^{ZsGreen}-receiving mice at MI 4d. Data are presented as mean percentages of

each subset among total $ZsGreen^+CD45^+$ cells (n as indicated). **The above new data are included in Fig. 2 in the revised manuscript.**

7. The study attributes the anti-inflammatory effects of MDVs to ATP synthase content and metabolic reprogramming of macrophages. However, the possibility of other cargo (e.g., mitochondrial DNA, lipids, or ROS) contributing to macrophage polarization remains unexplored. Additionally, loss-of-function experiments (e.g., ATP synthase inhibition) may further clarify the mechanism.

Response:

Thank you for this insightful mechanistic comment. To directly address whether ATP synthase/Complex V activity is required for the anti-inflammatory effects of MDVs, we performed additional loss-of-function experiments using pharmacologic inhibition.

First, we treated purified BAT-MDVs with oligomycin before adding them to macrophages culture media. Oligomycin (100μg/mL) markedly reduced Complex V activity in MDVs, as assessed by NADH oxidation (Fig. R6a). Oligomycin(100μg/mL)-treated MDVs (oligo-MDVs) no longer suppressed iNOS or induced ARG1 expression compared with control MDVs (Fig. R6b). Consistently, oligo-MDVs failed to increase IL-4 and VEGF secretion, whereas untreated MDVs significantly enhanced these cytokines (Fig. R6c). These data indicate that ATP synthase activity in MDVs contribute to macrophage reprogramming.

Second, we modulated ATP5A1 within macrophages. Overexpression of Flag-Atp5a1 in macrophages decreased iNOS and increased ARG1 protein, accompanied by higher IL-4 and VEGF levels (Fig. R6 d, e), further supporting a functional role for Complex V in promoting a pro-reparative phenotype.

We fully agree that other MDV cargo (mtDNA, lipids, ROS) may also contribute, as oligomycin does not completely abolish the protective effects of MDVs and ATP5A1 overexpression only partially restores the anti-inflammatory phenotype. In the revised Discussion we now explicitly acknowledge these possibilities and state that ATP synthase/Complex V should be regarded as a required, but not necessarily exclusive, mediator of MDV-induced macrophage polarization.

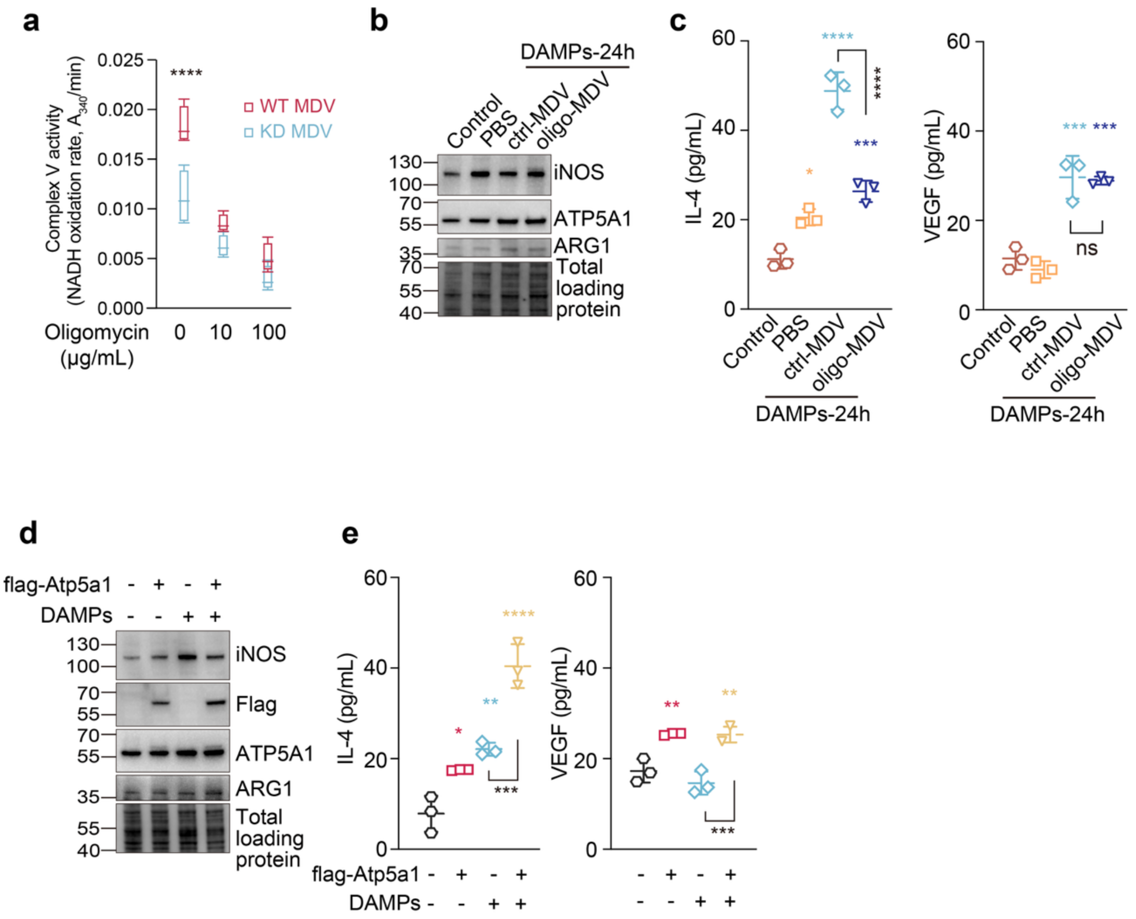

Fig. R6 a, Purified WT-MDVs and KD-MDVs were incubated with the indicated concentrations of oligomycin. NADH oxidation rate (A_{340}/min) was measured as a readout of complex V activity. (mean±s.d., n=4, two-way ANOVA and Holm-Sidak post hoc, ****p < 0.0001). **b,** Representative western blots of macrophages treated with DAMPs for 24 h in the presence of PBS-treated MDVs, or oligomycin(100μg/mL)-treated MDVs (n=3 independent experiments). **c,** IL-4 and VEGF concentrations in supernatants from macrophages treated as in (**a**), measured by ELISA. (n=3 independent experiments). **d,** Representative western blots of macrophages infected with flag-Atp5a1 lentivirus and treated with DAMPs for 24 h (n=4 independent experiments). **e,** IL-4 and VEGF concentrations in supernatants from macrophages treated as in (**d**), measured by ELISA. (n=3 independent experiments). **The above new data are included in Fig. 7 in the revised manuscript.**

Manuscript edits: “Discussion”

Mechanistically, our findings position ATP synthase/Complex V as a required, but not exclusive, mediator of MDV-induced macrophage polarization toward a reparative state. The incomplete loss of protection with oligomycin-treated MDVs and the partial restoration of anti-inflammatory features by ATP5A1 overexpression indicate that additional MDV cargo, such as mtDNA, lipids or mitochondrial ROS, are likely to cooperate with the respiratory chain/bioenergetic unit. Dissecting how these cargoes integrate to control macrophage metabolism and phenotype will be an important objective for future work.

8. In Figure 3, the authors propose that BAT-derived MDVs harboring mitochondrial complex V is a key driver of cardiac repair post-MI, with MDVs transferred to infarct regions via EVs. However, several points require further validation: a. While the authors claimed that only BAT MDVs with complex V enhance repair, their data show that WAT MDVs also contain ATP5A (a complex V subunit) yet lack efficacy in reducing fibrosis. This inconsistency raises questions about whether complex V components alone are sufficient for repair or if other MDV-associated factors (e.g., metabolic cargoes, scaffold proteins) synergize with complex V. Additional experiments are needed to isolate the role of complex V. b. Figure 3e shows minimal ATP5A in EVs, conflicting with the claim that MDVs transfer complex V. Clarification is needed on how MDVs selectively package complex V into EVs if ATP5A levels are low. c. The analysis of mitochondrial OXPHOS components in Figure 3f lacks normalization to total loading protein. Uneven loading within groups (notably the LV-EV MI group) obscures statistical comparisons and biological interpretation.

Response:

We really appreciate your detailed and thoughtful critique, which prompted us to perform additional experiments and to clarify our interpretation of Figure 3.

(a) BAT- vs WAT-derived MDVs and the role of Complex V

In the original manuscript, MDVs were isolated from equal tissue mass (800 mg BAT or WAT). Because WAT produces substantially fewer MDVs than BAT (Fig. 3b), mice in the WAT-MDV group effectively received a much lower MDV dose, which likely explained their weaker protection despite detectable ATP5A1. In the revised study, we therefore normalized MDV administration by MDV protein (take a small proportion of isolated MDVs and quantify the protein concentration by BCA method), injecting 100μL MDV (at the concentration of 300μg protein/mL) per mouse (i.v.) from either WAT or BAT. Under these dose-matched conditions, WAT- and BAT-derived MDVs produced very similar improvements in ejection fraction, fractional shortening, and fibrosis across 7-28 days post-MI (Fig. R7 a,b).

These data indicate that WAT-MDVs are also cardioprotective when administered at an equivalent MDV dose, and that the earlier “BAT-specific” effect was confounded by MDV yield rather than the simple presence of ATP5A1.

To more directly address the role of Complex V, we additionally performed loss- and gain-of-function experiments. Pharmacologic inhibition of Complex V with oligomycin markedly abolished the ability of MDVs to suppress iNOS, induce ARG1, and increase IL-4 and VEGF in DAMP-stimulated macrophages (Fig. R6 a-c). Conversely, ATP5A1 overexpression in macrophages decreased iNOS, increased ARG1, and enhanced IL-4/VEGF secretion (Fig. R6 d,e).

In the revised manuscript, we therefore explicitly state that Complex V is necessary but not exclusive, and we avoid implying that it is BAT-specific cardioprotection.

(b) ATP5A1 levels in EVs and MDVs

We agree that the ATP5A1 signal in bulk EV preparations in the original Fig. 3e appeared low by Western blot. The ATP5A1 level in EVs is influenced by experimental parameters such as the EV isolation method, sample purity, and antibody usage et al. In the revision, we optimized our EV isolation based on the BAT sEV protocol described by Rosina et al (Cell Metab. 2022, PMID: 35305295), in which the proteomics of BAT-sEV also confirms the presence of complex V units (such as ATP5A1) in BAT-sEVs. The recovered sEV were washed, resuspended with PBS, and then ultracentrifuged again to increse purity. OXPHOS antibody concentrations in the micture was optimized to visualize all proteins.

Using this refined approach, we now show that ATP5A1 is detectable in sEVs derived from BAT and LV, although its abundance remains lower than in purified MDVs, as expected if only a minor subset of EVs are MDV-like vesicles enriched for OXPHOS proteins (Fig. R7 c,d).

Importantly, after MI we observe the pattern: ATP5A1 levels increase in BAT sEVs and decrease in LV sEVs. A possible model that explains this phenomenon is that BAT

secretes ATP5A1-containing sEVs into the interstitial space and circulation, whereas the injured LV acts as a sink, preferentially internalizing these ATP5A1-containing vesicles.

(c) Normalization of OXPHOS components and loading concerns in Fig. 3f

In response, we have analyzed Western blots in Fig. R7d using total-protein normalization as the primary loading control and quantified ATP5A1 protein level in BAT and LV sEVs (Fig. R7d). MI upregulated ATP5A1 levels in BAT sEVs and downregulated ATP5A1 in LV sEVs.

Manuscript edits: “Results”

BAT-MDVs contribute to MI injury repair

Notably, whole-tissue WAT transplantation did not confer detectable cardioprotection in contrast to BAT transplantation (Fig. 1c-f), whereas isolated WAT-MDVs were cardioprotective when administered in equal amounts. Several factors may account for this divergence. First, WAT contains substantially fewer MDVs than BAT (approximately 5–10-fold lower MDV yield, Fig. 3b), making endogenous WAT-derived MDV flux to the heart relatively inefficient, whereas direct MDV injection circumvents this limitation by equalizing vesicle dose. Second, WAT releases a complex secretome that includes non-coding RNAs, fatty acids, and proinflammatory adipokines, which are known to promote systemic inflammation, adverse cardiac remodeling, and heart failure[10,24]. These detrimental signals are likely to outweigh any modest benefit from the small amount of WAT-derived MDVs after WAT transplantation, whereas purified WAT-MDVs administered in isolation can reveal the intrinsic cardioprotective potential of MDVs as a vesicle class.

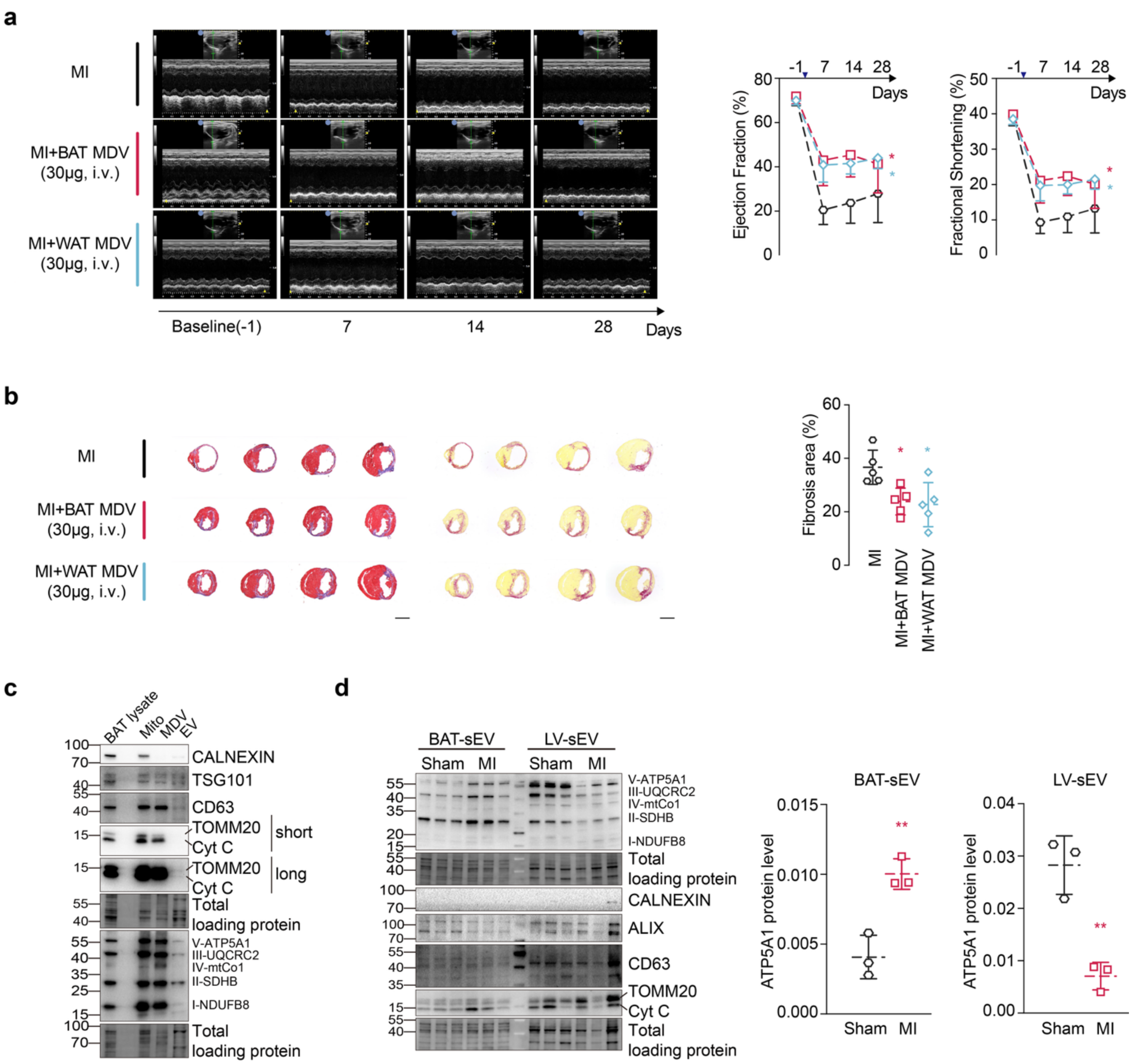

Fig. R7 a, Representative B-mode and M-mode echocardiography of adipose tissue-derived MDV-treated mice at baseline and days 7, 14, and 28. Ejection fraction and fractional shortening were quantified (mean±s.d., n=5, repeated two-way ANOVA and Holm-Sidak post hoc, *p < 0.05). **b,** Representative Masson and Sirius Red staining images of MDV-treated mouse hearts at day 28. Fibrosis percentage was quantified (mean±s.d., n=5, One-way ANOVA test followed by Bonferroni method, *p < 0.05). Scale bar: 2mm. **c,** Western blots of mouse BAT whole tissue lysate, purified mitochondria, MDV, and EV. **d,** Western blots of sEV enriched in BAT and heart left ventricle at steady state or under MI stress. The ATP5A1 protein levels were quantified (mean±s.d., n=3, unpaired

student’s t-test, **p < 0.01). **The above new data are included in Fig. 3 in the revised manuscript.**

9. In Figure 4e, the quantitative data appear to reflect variability in the loading control (Actb) rather than meaningful changes in VPS35 protein levels. Notably, the representative blots suggest no clear upregulation of VPS35 in BAT under MI stress, contrary to the authors' claims of time-dependent protein upregulation.

Response:

We are grateful for this careful and important observation.

We agree that Actb may not be an appropriate loading control in the context of MI-induced stress, and that variability in Actb could confound the interpretation of VPS35 changes in our original Fig. 4e. To address this, we re-collected BAT samples at MI day 1, 3, 7 and conducted the BAT Western blots using total protein staining as the loading reference instead of Actb. Total loading protein was used as reference in all western blots in the revised manuscript.

VPS35 and BECN1 were quantified and normalized to the total protein signal in each lane (Fig. R8a). This analysis shows a statistically significant, time-associated increase in VPS35 and BECN1 levels at MI d3 and the return towards baseline by MI d7.

These data indicate an early, transient activation of the BECN1-VPS35 axis in BAT under MI stress.

a

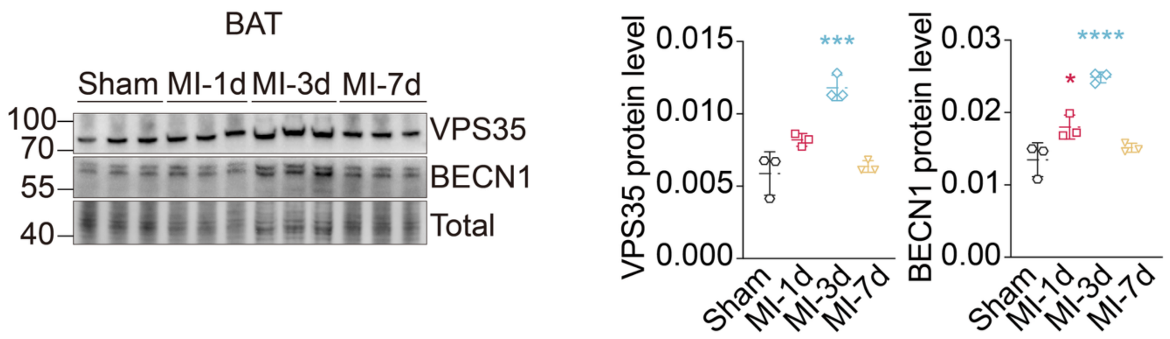

Fig. R8 a, Time course of VPS35 and BECN1protein expression in BAT after MI. Representative immunoblots and quantification of BECN1 and VPS35 in BAT lysates from sham, MI-1 d, MI-3 d, and MI-7 d mice (mean±s.d., n=3, One-way ANOVA test followed by Bonferroni method). **The above new data are included in Fig. 4 in the revised manuscript.**

10. In Figure 4j, the labels "1" and "2" on the images lack clarity regarding which parts are the insets. Please provide clarification to specify the insets.

Response:

Thank you for pointing out this issue. In the revised version, we have re-drawn Figure 4j to make the relationship between the main image and the insets unambiguous. We now mark the regions of interest in the main image with clearly visible rectangular boxes labeled “1” and “2,” and we use matching labels on the corresponding higher-magnification panels.

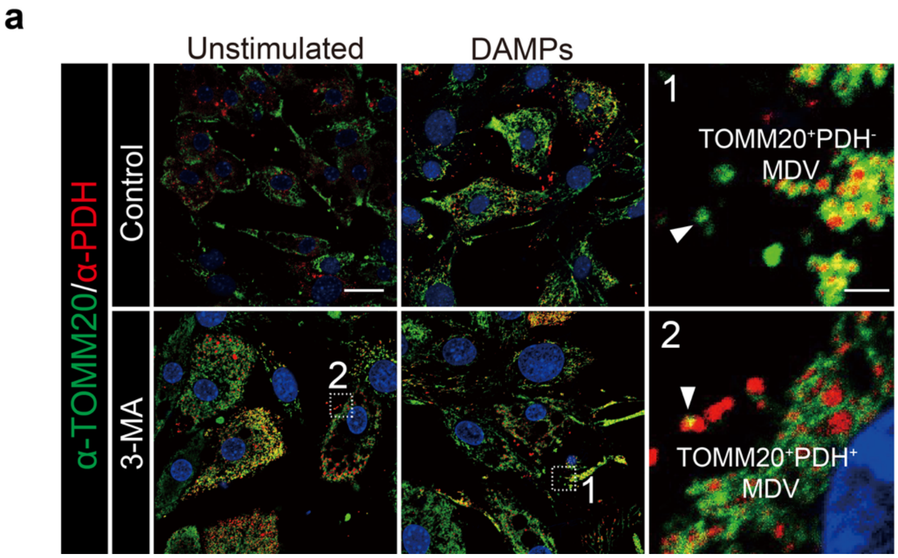

Fig. R9 a, Representative immunocytochemistry images of 3-MA and DAMPs-treated brown adipocytes stained with DAPI (blue), TOMM20 (green), and PDH (red). Scale bar: 20μm; 2μm (insets). **The above new data are included in Fig. 4 in the revised manuscript.**

11. In Figure 6b, the fluorescence images are too dark to discern meaningful details.

Response:

Thank you for drawing our attention to the display quality of these images.

In the revised manuscript, we have re-rendered the confocal images from the original image and adjusted the brightness and contrast to improve the visibility of GFP^{+} cells.

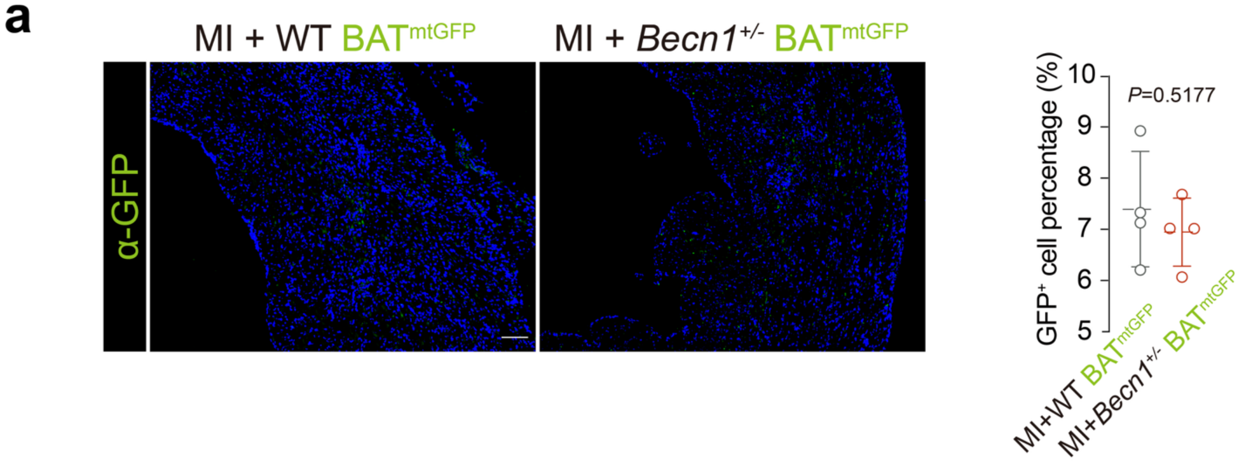

Fig. R10 a, Representative confocal images of BAT^{mtGFP}-receiving mice at MI 4d, stained for GFP (green) and nuclei (DAPI, blue). Scale bar: 100μm. Mt-EGFP^{+} cell percentage was quantified (mean±s.d., n=4, unpaired Student's t-test). **The above new data are included in Fig. 5 in the revised manuscript.**

Manuscript edits: "Results"

***Becn1*-deficiency ablates BAT cardioprotection**

To determine the effects of BECN1-defficient BAT and its MDV, we generated donor BAT expressing a mitochondrial GFP reporter (AAV8-mtGFP) in wild-type or *Becn1* $^{+/-}$ mice and transplanted equal amounts of BAT to the pericardial space of MI recipients (Fig. 5a). Donor BAT from wild-type and *Becn1* $^{+/-}$ mice released similar amounts of MDV, and the proportion of GFP^{+} cells in recipient hearts after transplantation was comparable between the two donor genotypes, indicating similar overall vesicle production and delivery (Fig. 5b, c).

12. The authors state at line 618 that "the effects of WT and Becn1+/- BAT were notably opposite." However, in Figure 5d, while the WT BAT transport group shows enhanced repair compared to the control, the Becn1+/- BAT group only exhibits no significant difference from the control. This does not represent "opposite effects" but rather a lack of effect in the Becn1+/- group. Please refine the text to more accurately reflect the observed differences.

Response:

Thank you for pointing out. We agree that the conclusion "the effects of WT and *Becn1*$^{+/-}$ BAT were notably opposite" was misleading.

We re-performed all animal phenotyping experiments, including the WT vs *Becn1*$^{+/-}$ BAT transplantation study, using an improved study design: (i) mid-LAD ligation to produce a more standardized infarct, (ii) increased sample size, and (iii) extended follow-up time. In these revised experiments, WT BAT transplantation again resulted in significant improvement in cardiac function (EF, FS) and reduced fibrosis compared with MI controls, whereas *Becn1*$^{+/-}$ BAT transplantation failed to provide detectable benefit, with EF, FS, and fibrotic area remaining similar to MI controls (Fig. R11). These data support the conclusion that *Becn1*-knockdown ablates the cardioprotective effect of BAT transplantation.

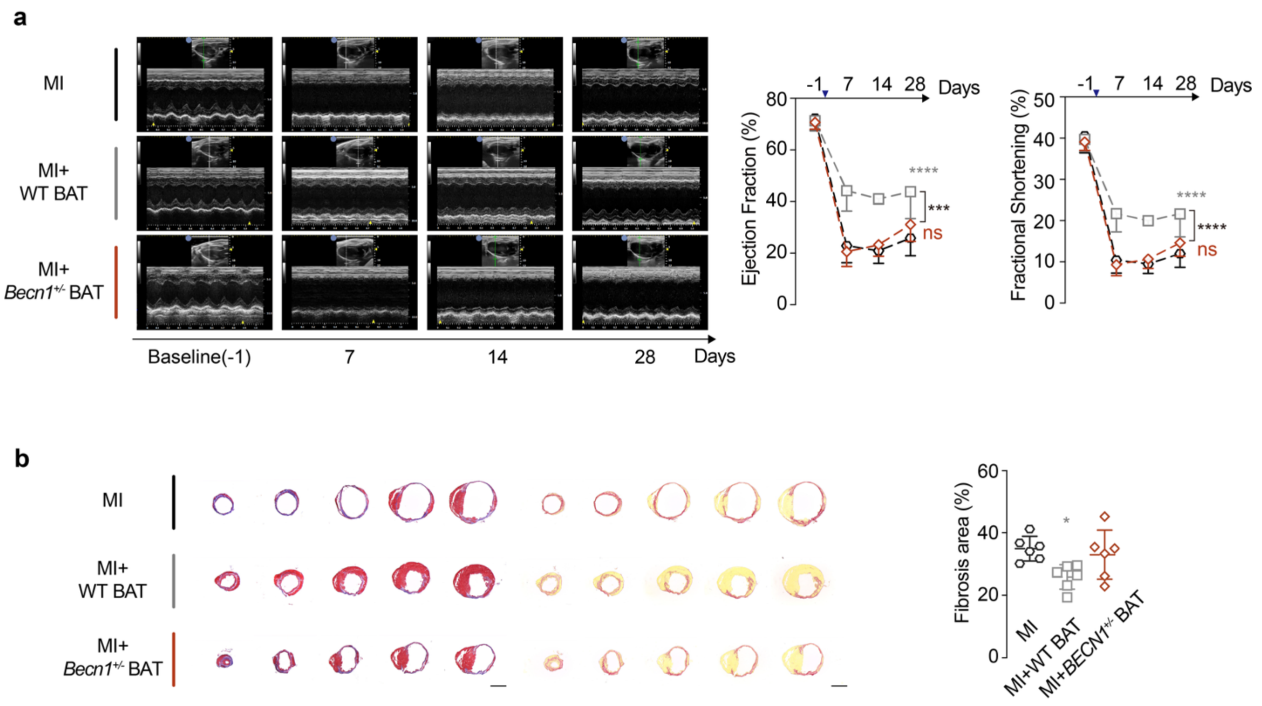

Fig. R11 a, Representative B-mode and M-mode echocardiography of WT or *Becn1*$^{+/-}$ BAT-receiving mice at baseline and days 7, 14, and 28. Ejection fraction and fractional shortening were quantified (mean±s.d., n=6, repeated two-way ANOVA and Holm-Sidak post hoc, ***p < 0.001, ****p < 0.0001). **b,** Representative Masson and Sirius Red staining images of WT or *Becn1*$^{+/-}$ BAT-receiving mice hearts at day 28. Fibrosis percentage was quantified (mean±s.d., n=6, One-way ANOVA test followed by Bonferroni method, *p < 0.05). Scale bar: 2mm. **The above new data are included in Fig. 5 in the revised manuscript.**

Manuscript edits: “Results”

***Becn1*-deficiency ablates BAT cardioprotection**

Echocardiography revealed that transplantation of wild-type BAT improved left ventricular ejection fraction and fractional shortening during the 4 weeks after MI and reduced infarct fibrosis, whereas BAT from *Becn1*$^{+/-}$ donors conferred no improvement despite

comparable engraftment (Fig. 5d, e). To directly test the role of BAT-derived MDVs, we isolated MDVs from wild-type or *Becn1*$^{+/-}$ BAT and administered them to MI mice. Delivery of wild-type BAT-MDVs recapitulated the functional and structural benefits of BAT transplantation, whereas MDVs from *Becn1*$^{+/-}$ BAT showed attenuated effects on both parameters (Fig. 5f, g). These data indicate that BECN1 in donor BAT is dispensable for bulk MDV yield and delivery but is required for the cardioprotective efficacy of BAT-derived MDVs in vivo.

13. In multiple panels, the authors report measurements of LV wall thickness, but no differences are shown, and these results are also not discussed in the text. Additionally, since MI surgery typically results in injury primarily around the LAD region, leading to non-uniform damage across the LV wall, it is essential to specify exactly which part of the LV wall was measured. Please clarify the location of the measured regions and address the lack of observed differences in the text.

Response:

We appreciate your important comment. In the original submission, the LV wall thickness was measured as follows: (i) identify the endocardial and epicardial borders in the anterior LV free wall within the infarct territory, (ii) 3 perpendicular measurements were made from the endocardial surface to the epicardial surface, and (iii) the mean of these measurements was used as the LV wall thickness for each heart.

We fully agree that (i) LV wall thickness is highly sensitive to regional heterogeneity after LAD ligation and (ii) without a clear anatomical definition of the measurement site, such data are difficult to interpret. As a result, LV wall thickness could be a highly viriable and regionally biased parameter in the murine LAD-ligation model, and therefore is not a robust primary readout of cardiac remodeling.

Therefore we removed LV wall thickness readouts from the main figures, retaining EF, FS and histological fibrosis as the primary functional and structural endpoints. In addition, we strengthened the assessment of structural remodeling by applying Masson staining and Sirius Red staining together to determine the exact fibrosis area at day 28 after MI induction. Hearts were harvested and serially sectioned from apex to the ligation site. Usually 5-6 section levels were made, and for each level we perform Masson trichrome and Sirius Red staining on adjacent sections. The fibrotic area was defined blue-stained collagen deposition on Masson staining and Sirius Red-positive fibrillar collagen area on corresponding Sirius Red-stained sections. The mean fibrosis percentage across all levels is used as the primary pathological endpoint per heart.

14. In Figure 6, the authors propose that MDV delivery of bioenergetic units shifts macrophages toward an anti-inflammatory phenotype. However, the evidence provided is predominantly transcriptional. To strengthen this conclusion, please include serial experiments validating macrophage polarization at the protein and cytokine levels.

Response:

Thank you for raising this important point. We agree that macrophage polarization cannot rely on transcriptional signatures alone and should be supported by protein and cytokine readouts.

To address this, we first focused on inducible nitric oxide synthase (iNOS) and arginase-1 (ARG1), which compete for the common substrate L-arginine and act as core metabolic “switch” that determines the inflammation status of macrophages. $iNOS^{high}ARG1^{low}$ defines classically activated macrophage, whereas $iNOS^{low}ARG1^{high}$ macrophages are associated with tissue-repair and anti-inflammatory programmes. Thus, protein-level measurement of iNOS and ARG1 is widely used as a core criterion for defining macrophage polarization.

In the revised manuscript we therefore performed Western blots and cytokine assays in DAMP-activated macrophages treated with MDVs (Fig. R12 a-c). WT-MDVs decreased iNOS and increased ARG1 protein compared with PBS and Becn1-KD MDVs, and this shift was accompanied by higher IL-4 secretion and increased Il4 mRNA expression. In contrast, KD-MDVs largely failed to induce these changes. Complementary experiments with Complex V inhibition (oligomycin-treated MDVs) and ATP5A1 overexpression (Fig. R6) show that disrupting MDV complex V activity prevents the enzyme shift and blunts IL-4 induction, further linking MDV Complex V activity to the protein-level polarization state.

These results provide the protein- and cytokine-level validation and, together with the RNA-seq data in Fig. 7, support a conclusion that MDV delivery of bioenergetic cargo is consistent with a shift toward a pro-reparative, anti-inflammatory macrophage phenotype.

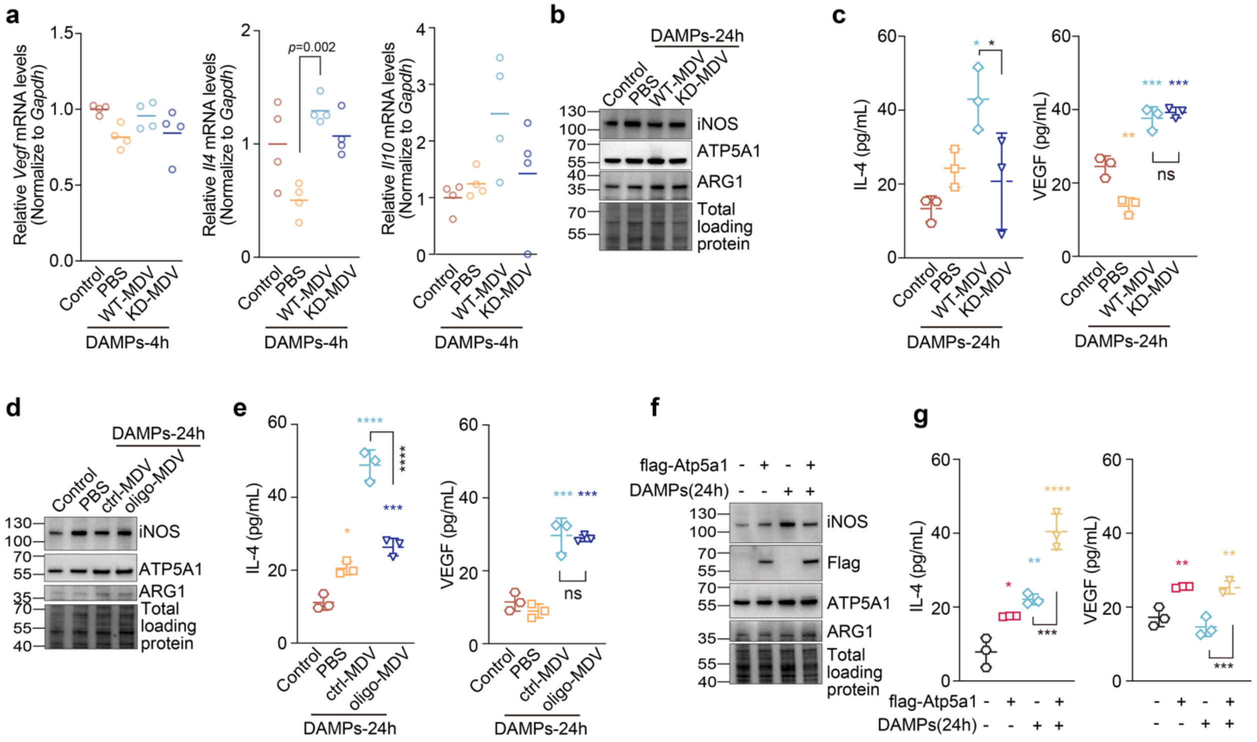

Fig. R12 a, RT-qPCR analysis of pro-reparative cytokine genes (*Vegf*, *Il4*, *Il10*) in iBMDM co-incubated with WT or *Becn1*$^{+/-}$ BAT MDV (mean, n=4 independent experiments, one-way ANOVA test followed by Bonferroni method). **b,** Representative western blots of macrophages treated with DAMPs for 24 h in the presence of PBS, WT-MDVs, or KD-MDVs (n=3 independent experiments). **c,** IL-4 and VEGF concentrations in supernatants from macrophages treated as in (**b**), measured by ELISA. (n=3 independent experiments). **d,** Representative western blots of macrophages treated with DAMPs for 24 h in the presence of PBS-treated MDVs, or oligomycin(100μg/mL)-treated MDVs (n=3 independent experiments). **e,** IL-4 and VEGF concentrations in supernatants from macrophages treated as in (**d**), measured by ELISA. (n=3 independent experiments). **f**, Representative western blots of macrophages infected with flag-Atp5a1 lentivirus and treated with DAMPs for 24 h (n=4 independent experiments). **g,** IL-4 and VEGF concentrations in supernatants from macrophages treated as in (**f**), measured by ELISA. (n=3 independent experiments). **The above new data are included in Fig. 7 in the revised manuscript.**

15. In Figure 7, the data are largely transcriptomic. Validation at the protein level is necessary to strengthen these findings. Additionally, there is insufficient evidence provided regarding the origin of Complex V.

Response:

Thank you for bringing up this important point.

The aim of this analysis is to explore whether a Complex V-related signature in human cardiac cell populations, particularly macrophages, is associated with ventricular remodeling. Using a public human heart RNA-seq dataset that provides cell-type-resolved expression and cardiac functional data, we developed a Complex V gene-set score and found that higher Complex V scores, both at the tissue level and specifically in macrophages, correlate with higher LVEF and lower LV mass index, whereas Complex V scores in fibroblasts or lymphocytes show no significant relationship with these parameters (Fig. 8). These observations support our conclusion that macrophage Complex V is positively associated with better cardiac function and less adverse remodeling in human HF, in line with our animal and cell-level data. We have retained Fig. 8 as a translational correlative analysis that connects our mechanistic mouse data to human heart-failure cohorts.

We fully acknowledge these two important limitations. First, protein-level validation in humans is needed. The human dataset is purely transcriptomic and publicly available; no matched proteomic data are accessible, and additional human sampling is not feasible within this study. We have therefore revised the text to explicitly state that the human findings represent transcriptional associations, and highlight in the Discussion that future, prospective cohorts with integrated proteomics will be required to confirm the conclusion.

Second, origin of complex V in patients remained unclear. With current clinical data, we can not determine whether Complex V detected in human cardiac macrophages derives from endogenous mitochondrial biogenesis or from uptake of MDV-derived OXPHOS cargo. We have therefore revised the manuscript to state explicitly that, in the human

analysis, Complex V is used as a indicator for macrophage OXPHOS capacity, not as direct evidence of MDV transfer. We also acknowledge that tracing the origin of MDVs in patients will require new vesicle-tracing and multi-omic approaches.

Manuscript edits: “Results”

Macrophage complex V levels correlate with cardiac functions clinically

Using the public transcriptional data from non-failing and heart-failure patients, we calculated a Complex V gene set score for each sample and major cardiac cell types. Complex V scores were significantly lower in macrophages but not in other cell types (Fig. 8 a-d). Higher Complex V scores, particularly in macrophages, were positively associated with LVEF and inversely associated with LV mass index, whereas Complex V scores in fibroblasts or lymphocytes showed no significant relationship with these parameters (Fig. 8 e-h).

“Discussion”

In the human heart-failure cohorts, Complex V was evaluated at the transcript level, and protein-level in cardiac macrophages could not be assessed. Thus, these data represent transcriptomic associations rather than direct evidence of MDV transfer. Future studies will require protein-level validation and the development of new tools (tissue-specific MDV markers, vesicle-tracing strategies) to trace MDV origin and fate in patients.

16. The manuscript would benefit from substantial text refinement to enhance clarity and precision. In particular, summary sentences for each part of the results are either missing or improperly formatted.

Response:

We are deeply grateful for this very helpful comment. In the revised manuscript, we have therefore undertaken a line-by-line revision of the Results section to improve clarity, precision, and logic flow. Specifically, we revised the text throughout the Results to describe the data in more precise and scientifically rigorous terms and avoided long or redundant sentences, standardized the description of experimental groups and figure readouts, and rewrote the summary and transition sentences in each Results subsection so that the logical flow are clearly presented.

Reviewer #2 Comments

Shi and coworkers describe that BAT-derived MDVs, containing mitochondrial membrane components such as OXPHOS proteins (e.g., Complex V), exert a cardioprotective effect. Upon myocardial infarction (MI), cardiac macrophages potentially uptake these MDVs, leading to reduced fibrotic remodeling and an enhanced anti-inflammatory response through augmented reparative cytokine production and OXPHOS rewiring. The findings suggest that physical contact between BAT-MDVs and cardiac macrophages is essential for the cardioprotective effect, with VPS35 and Becn1 proteins facilitating endosomal trafficking.

While the concept is interesting and a lot of data is provided, some analyses lack robustness. One concern regards the analysis of cardiac function and outcome after myocardial infarction. Authors assessed fibrosis (by histology) and left ventricular functon(by echocardiography) at day 4 or 7 after induction of myocardial infarction. Fibroblast activation occurs within the first days after MI, leading to potential changes in Trichom stainings. However, fibroblast maturation, scar formation and conclusion of remodeling takes significantly longer. Specifically in the setting of permanent ligation, the inflammatory phase require longer periods of time as compared to I/R injury. Assessment of scaring and functional outcomes are typically conducted two to fours weeks after MI. In the same line, as remodeling is not completed at day7 after infarction, LV wall thickness is not a good parameter at this timepoint and highly variable (see 1e). Thus interpretation of functional outcome after BAT transplantation is limited.

Response：

We appreciate your positive comments on the conceptual advances of our work, and we are deeply grateful for your important comments on the methodological limitation.

We fully agree that the early (day 4-7) detection window incompletely captures the biology of scar maturation and global functional remodeling after permanent LAD ligation. We re-conducted all the mouse phenotyping with a primary endpoint at day 28 after MI induction and improved the study design.

i) In the revised manuscript, left ventricular function was assessed by serial echocardiography at baseline (a day before MI induction), day 7, day 14, and day 28 after MI induction. On day 28 after MI induction, hearts were harvested. Ventricular remodeling and fibrosis were assessed by Masson’s trichrome staining and Picrosirius Red staining.
ii) We also increased group sizes across groups to enhance statistical power.
iii) We have standardized the surgical procedure, adopting a moderate LAD ligation technique (consistent placement ~1-2 mm below the LAA with controlled tension) instead of the proximal LAD ligation technique used in the original manuscript, to reduce mortality and minimize survival bias.

The results of these revised data for four major phenotype experiments are listed below.

1. Pericardial BAT transplantation attenuated adverse post-MI remodeling, as BAT-grafted mice showed higher EF/FS and smaller fibrotic areas than MI controls, whereas BAT-deficient mice had worse function and more fibrosis. WAT transplantation provided no benefit, while cold-activated BAT further improved systolic function, indicating a BAT-related, thermogenesis-enhanced cardioprotective effect.
2. Intravenous delivery of adipose-derived MDVs (30 μg protein per mouse) after MI improved LV systolic function, limited adverse remodeling, and reduced fibrosis compared with vehicle controls. BAT- and WAT-derived MDVs exerted comparable benefits, indicating that MDVs from either adipose depot possess intrinsic cardioprotective activity.

3. Transplantation of wild-type BAT improved EF/FS over 4 weeks after MI and reduced infarct fibrosis, whereas BAT from *Becn1*$^{+/-}$ donors, despite similar engraftment, failed to confer functional or structural benefit, indicating that BECN1 in donor BAT is required for BAT-mediated cardioprotection.
4. Intravenous delivery of wild-type BAT-MDVs improved LV function and reduced fibrosis, whereas MDVs from *Becn1*$^{+/-}$ BAT showed markedly attenuated effects, demonstrating that BECN1 is dispensable for bulk MDV yield but essential for the cardioprotective efficacy of BAT-derived MDVs in vivo.

The inconsistency between the effects of WAT-transplantation and WAT-MDV treatment can be explained by the much lower yield of MDV from WAT, and the secretion of cardio deleterious lipids and adipokines from WAT, thereby unmasking the intrinsic cardioprotective capacity of adipose-derived MDVs.

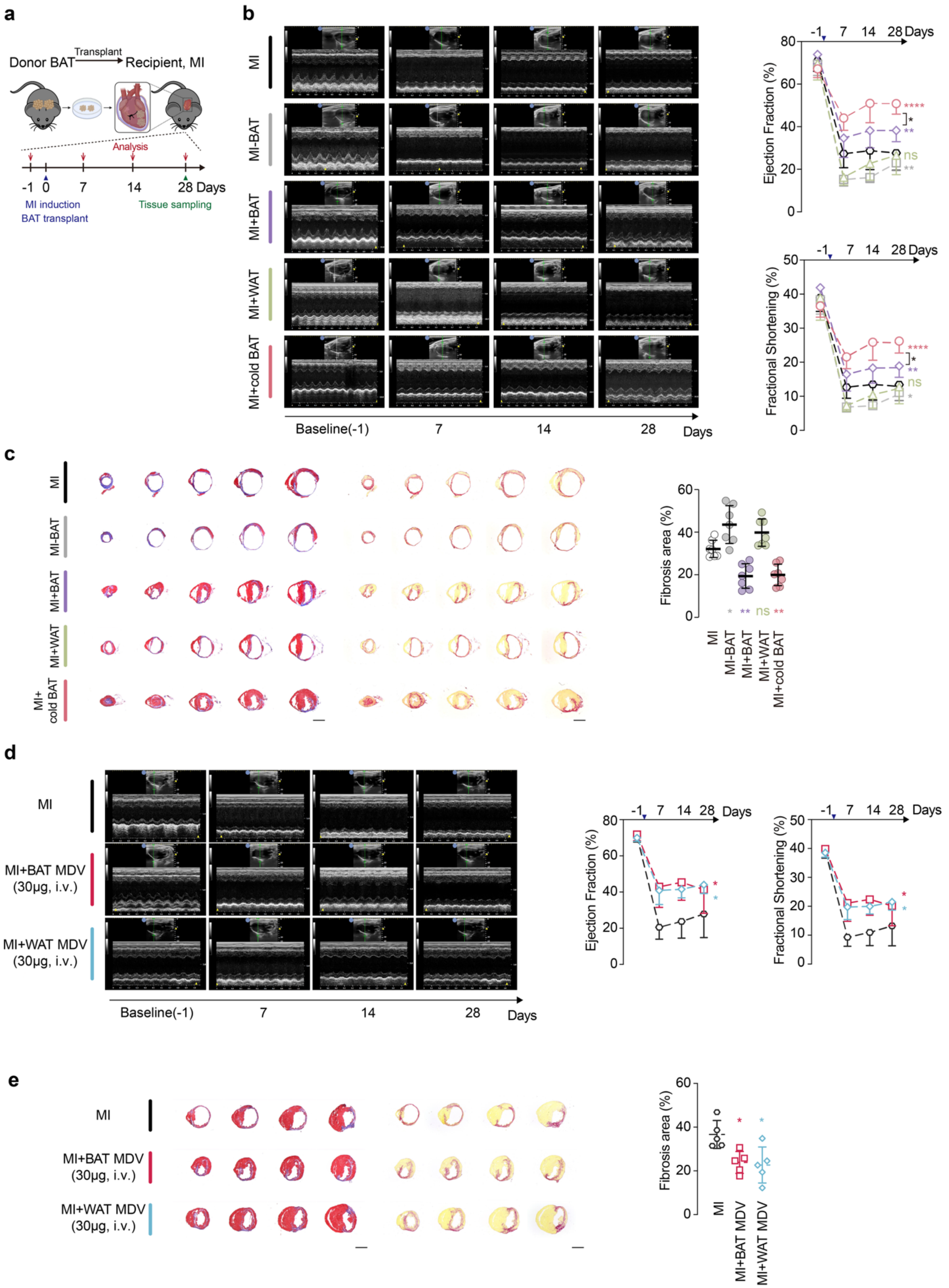

a
Donor BAT
Transplant
Recipient, MI
Analysis
-1 0 7 14 28 Days
MI induction
BAT transplant
Tissue sampling
b
MI
MI-BAT
MI+BAT
MI+WAT
MI+cold BAT
Baseline(-1) 7 14 28 Days
Ejection Fraction (%)
Fractional Shortening (%)
c
MI
MI-BAT
MI+BAT
MI+WAT
MI+cold BAT
Fibrosis area (%)
d
MI
MI+BAT MDV (30μg, i.v.)
MI+WAT MDV (30μg, i.v.)
Baseline(-1) 7 14 28 Days
Ejection Fraction (%)
Fractional Shortening (%)
e
MI
MI+BAT MDV (30μg, i.v.)
MI+WAT MDV (30μg, i.v.)
Fibrosis area (%)

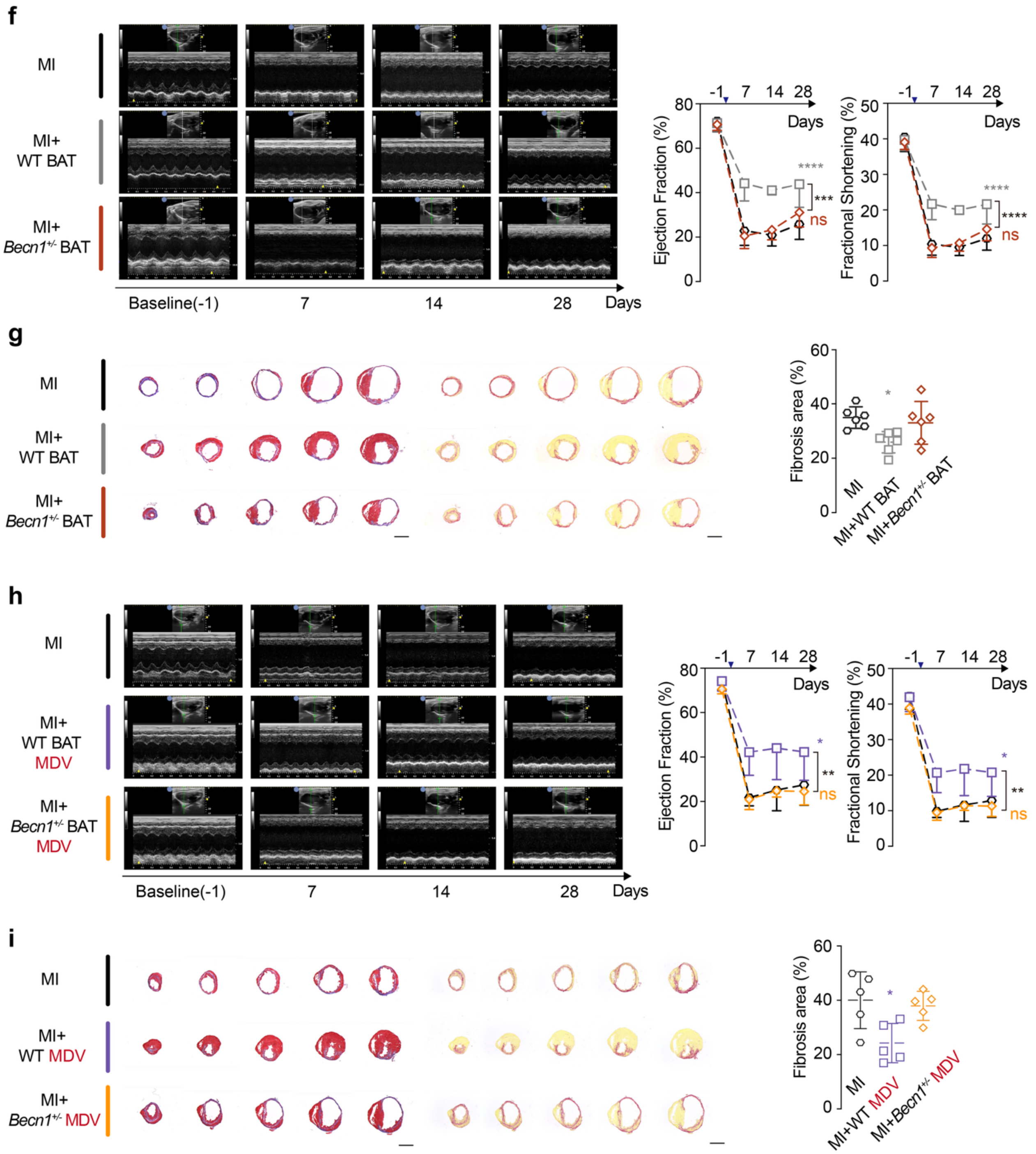

Fig. R13 a, Experimental timeline for MI induction, adipose tissue pericardial transplantation, and outcome assessments. Echocardiography was performed at baseline (day −1) and on days 7, 14, and 28. Hearts were harvested on day 28 for Masson's trichrome and Sirius Red staining. **b,** Representative B-mode and M-mode echocardiography of BAT-receiving mice at baseline and days 7, 14, and 28. Ejection

fraction and fractional shortening were quantified (mean±s.d., n=7, repeated two-way ANOVA and Holm-Sidak post hoc, *p < 0.05, **p < 0.01, ****p < 0.0001). **c,** Representative Masson and Sirius Red staining images of mouse hearts at day 28. Fibrosis percentage was quantified (mean±s.d., n=7, One-way ANOVA test followed by Bonferroni method, *p < 0.05, **p < 0.01, ***p < 0.001, ****p < 0.0001). Scale bar: 2mm. **d,** Representative B-mode and M-mode echocardiography of adipose tissue-derived MDV-treated mice at baseline and days 7, 14, and 28. Ejection fraction and fractional shortening were quantified (mean±s.d., n=5, repeated two-way ANOVA and Holm-Sidak post hoc, *p < 0.05). **e,** Representative Masson and Sirius Red staining images of MDV-treated mouse hearts at day 28. Fibrosis percentage was quantified (mean±s.d., n=5, One-way ANOVA test followed by Bonferroni method, *p < 0.05). Scale bar: 2mm. **f,** Representative B-mode and M-mode echocardiography of WT or *Becn1*$^{+/-}$ BAT-receiving mice at baseline and days 7, 14, and 28. Ejection fraction and fractional shortening were quantified (mean±s.d., n=6, repeated two-way ANOVA and Holm-Sidak post hoc, ***p < 0.001, ****p < 0.0001). **g,** Representative Masson and Sirius Red staining images of WT or *Becn1*$^{+/-}$ BAT-receiving mice hearts at day 28. Fibrosis percentage was quantified (mean±s.d., n=6, One-way ANOVA test followed by Bonferroni method, *p < 0.05). Scale bar: 2mm. **h,** Representative B-mode and M-mode echocardiography of WT or *Becn1*$^{+/-}$ MDV-treated mice at baseline and days 7, 14, and 28. Ejection fraction and fractional shortening were quantified (mean±s.d., n=5, repeated two-way ANOVA and Holm-Sidak post hoc, *p < 0.05, **p < 0.01). **i,** Representative Masson and Sirius Red staining images of WT or *Becn1*$^{+/-}$ MDV-treated mice hearts at day 28. Fibrosis percentage was quantified (mean±s.d., n=5, One-way ANOVA test followed by Bonferroni method, *p < 0.05). Scale bar: 2mm. **The above new data are included in Fig. 1, 3, and 5 in the revised manuscript.**

Manuscript edits: “Results”

BAT transplantation attenuates adverse post-MI cardiac remodeling

Pericardial BAT transplantation mitigated post-MI systolic decline. Across serial echocardiography at days 7, 14, and 28, BAT-treated mice maintained higher ejection

fraction and fractional shortening than MI controls, indicating sustained preservation of contractile performance (Fig. 1c, d). Concordant structural benefits were observed on Masson's trichrome and Picrosirius Red staining, which showed a smaller fibrotic area and better myocardial preservation in the BAT group (Fig. 1e, f). Consistently, BAT-deficient mice (MI-BAT, iBAT excision 1 week before MI) exhibited significantly lower EF%/FS% and greater fibrosis than MI controls, supporting an essential role of endogenous BAT in limiting post-MI remodeling (Fig. 1d, f).

As further validation, we compared different adipose grafts. White adipose tissue (WAT) and BAT arise from distinct adipocyte lineages and exert divergent metabolic and cardiovascular effects[3]. WAT transplantation did not modify MI-induced fibrosis or systolic function, whereas BAT transplantation reduced scar burden and preserved contractile performance (Fig. 1c-f), indicating BAT transplantation-related cardioprotection. To test whether BAT activation augments this benefit, donor mice were cold-exposed at 4 °C for 14 days before grafting. MI+cold BAT mice showed higher EF% and FS% than MI+BAT mice, while fibrosis burden remained comparable between the two groups (Fig. 1d,f, f). Collectively, these findings support a BAT-related, thermogenesis-enhanced cardioprotective effect on early post-MI remodeling.

BAT-MDVs contribute to MI injury repair

To assess whether MDVs contribute to BAT transplantation-related cardioprotection, MDVs were isolated from BAT or WAT and administered intravenously to mice subjected to LAD ligation. Vesicle doses were normalized by total protein content (30 μg MDV protein per mouse, quantified by BCA assay). Compared with vehicle-treated MI mice, both BAT-MDV and WAT-MDV-treated animals showed better preservation of cardiac functions, attenuation of adverse left ventricular remodeling, and smaller fibrosis area (Fig. 3d-g). No significant differences were detected between BAT- and WAT-MDV groups, indicating that adipose-derived MDVs, irrespective of depot origin, are capable of mitigating post-infarction structural remodeling.

Notably, whole-tissue WAT transplantation did not confer detectable cardioprotection in contrast to BAT transplantation (Fig. 1c-f), whereas isolated WAT-MDVs were

cardioprotective when administered in equal amounts. Several factors may account for this divergence. First, WAT contains substantially fewer MDVs than BAT (approximately 5-10-fold lower MDV yield, Fig. 3b), making endogenous WAT-derived MDV flux to the heart relatively inefficient, whereas direct MDV injection circumvents this limitation by equalizing vesicle dose. Second, WAT releases a complex secretome that includes non-coding RNAs, fatty acids, and proinflammatory adipokines, which are known to promote systemic inflammation, adverse cardiac remodeling, and heart failure. These detrimental signals are likely to outweigh any modest benefit from the small amount of WAT-derived MDVs after WAT transplantation, whereas purified WAT-MDVs administered in isolation can reveal the intrinsic cardioprotective potential of MDVs as a vesicle class.

Becn1-deficiency ablates BAT cardioprotection

Echocardiography revealed that transplantation of wild-type BAT improved left ventricular ejection fraction and fractional shortening during the 4 weeks after MI and reduced infarct fibrosis, whereas BAT from *Becn1*$^{+/-}$ donors conferred no improvement despite comparable engraftment (Fig. 5d, e). To directly test the role of BAT-derived MDVs, we isolated MDVs from wild-type or *Becn1*$^{+/-}$ BAT and administered them to MI mice. Delivery of wild-type BAT-MDVs recapitulated the functional and structural benefits of BAT transplantation, whereas MDVs from *Becn1*$^{+/-}$ BAT showed attenuated effects on both parameters (Fig. 5f, g). These data indicate that BECN1 in donor BAT is dispensable for bulk MDV yield and delivery but is required for the cardioprotective efficacy of BAT-derived MDVs in vivo.

The following additional points should be addressed:

1. In line 403: How much protein is loaded per well?

Response:

Before Western blotting, a small aliquot of each protein sample was taken for BCA-based protein quantification. Unless otherwise stated, cell or tissue lysates were loaded at 30 μg total protein per lane, and mitochondria or vesicle preparations at 15 μg per lane.

2. Page 12, line 328: blue fibrotic area/heart cross-section area…. Should be corrected to "heart cross-section". In addition, details about biological/technical replicates are missing in the legend.

Response:

Thank you for pointing out this issue. We have corrected the text “The fibrosis percentage was calculated by fibrotic area percentage on heart cross-section. Fibrosis% for each mouse was determined by its average fibrosis% from heart apex to ligated cross-section (5-6 sections per heart).” In addition, we have revised the figure legend to specify the biological or technical replicates.

3. In line 403: How much protein is loaded per well?

Response:

30 μg total protein per lane (cell or tissue lysates) or 15 μg total protein per lane (vesicle or mitochondria).

4. In line 440: TEAB stands for?

Response:

We apologize for the unclarity. TEAB was short for “tetraethylammonium bromide”. We have revised the manuscript “Method” section- Quantitative proteomics “Then the precipitates were washed with the pre-cooled acetone three times, redissolved in 200 mM tetraethylammonium bromide (TEAB), and subjected to ultrasonic dispersion.”)

5. Line 568 - " Specifically, OXPHOS levels in BAT-EVs increased post-MI, while they decreased in the left ventricle (LV)-EVs (Fig. 3f)" - From figure 3 this point is not clear - please add the quantification of the proteins, picture of whole blot (in supplement), and exposure time as shown in Figure 4, e.

Response:

We appreciate your careful observation and agree that the ATP5A1 signal in bulk EV preparations in the original Fig. 3e appeared relatively weak. Because ATP5A1 detection in EVs is highly sensitive to experimental conditions (including the isolation procedure, vesicle purity, and antibody concentration), we have re-optimized our EV workflow in the revised manuscript. Specifically, we adapted the BAT sEV isolation protocol described by Rosina et al. (Cell Metab. 2022, PMID: 35305295). sEV pellets were washed, resuspended in PBS, and subjected to an additional ultracentrifugation step to improve purity, and the OXPHOS antibody cocktail was freshly prepared to permit reliable visualization of all subunits.

Using this refined protocol, ATP5A1 is now clearly detectable in sEVs derived from both BAT and LV, although its abundance remains lower than in purified MDVs, as expected if only a minority of EVs represent MDV, OXPHOS-enriched vesicles (Fig. R14). Importantly, with the optimized conditions we observe an increase in ATP5A1 levels in BAT sEVs and a reciprocal decrease in LV sEVs after MI. These data are consistent with a model in which BAT secretes ATP5A1-containing sEVs into the interstitial space and circulation, whereas the injured LV functions as a sink that preferentially internalizes these ATP5A1-positive vesicles.

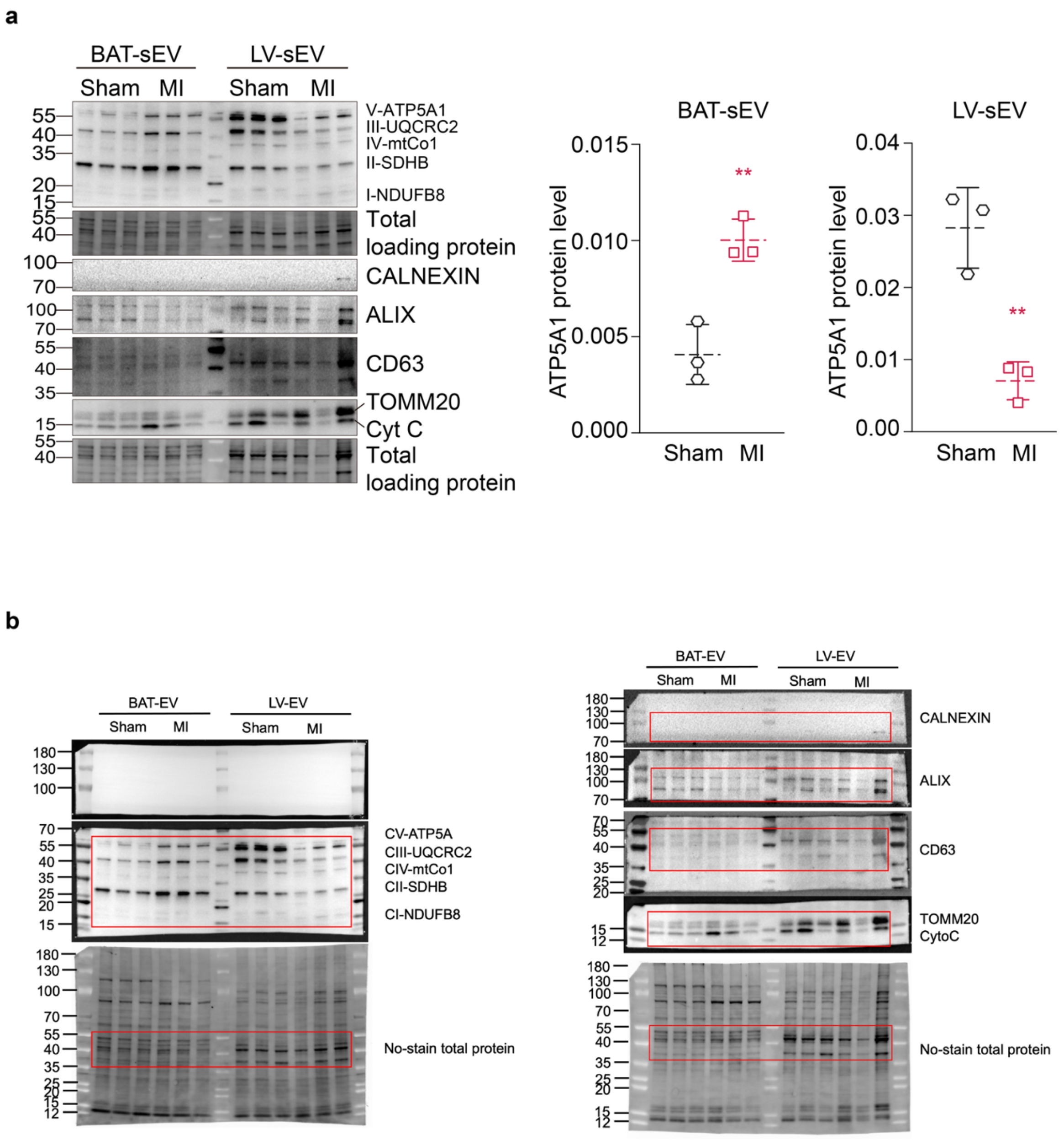

Fig. R14 a, Western blots of sEV enriched in BAT and heart left ventricle at steady state or under MI stress. The ATP5A1 protein levels were quantified (mean±s.d., n=3, unpaired student's t-test, $**p < 0.01$). **b,** The uncut gel in (**a**). **The above new data are included in Fig. 3 in the revised manuscript.**

6. Terminology - "under DAMPs stimulation" line 609, is misleading for figure 4 k. Please change or explain separately.

Response：

Thank you for pointing out that the phrase “under DAMPs stimulation” was imprecise in the context of Fig. 4k. This panel quantifies MDVs released from differentiated brown adipocytes stimulated in vitro with heart-derived DAMPs in the presence or absence of 3-MA, in comparison to unstimulated control cells.

To avoid confusion, we have revised the text in the Results and figure legend. The sentence now reads: “Quantification of extracellular $TOMM20^+PDH^-$ and $TOMM20^+PDH^+$ MDVs in differentiated brown adipocytes treated with heart-derived DAMPs revealed that 3-MA preferentially suppressed the DAMP-induced $TOMM20^+PDH^-$ subset (Fig. 4j). Under basal (unstimulated) conditions, MDV release was low and only modestly affected by 3-MA, whereas heart-derived DAMPs robustly increased $TOMM20^+PDH^-$ MDVs in control cells but not in 3-MA-treated cells. ”

In addition, the figure legend now explicitly states that the experiment was performed in cultured BAT adipocytes with or without heart-derived DAMPs and 3-MA. We believe this clarified terminology accurately reflects the experimental design shown in Fig. 4j and resolves the potential misunderstanding.

Manuscript edits: “Results”

Heart DAMPs engage the BECN1-VPS35 module in BAT that drives MDV biogenesis and cargo selection

Quantification of extracellular $TOMM20^+PDH^-$ and $TOMM20^+PDH^+$ MDVs released from differentiated brown adipocytes treated with heart-derived DAMPs showed that 3-MA selectively reduced the DAMP-induced $TOMM20^+PDH^-$ subset (Fig. 4j). Under basal (unstimulated) conditions, MDV release was low and only modestly affected by 3-MA, whereas heart-derived DAMPs robustly increased $TOMM20^+PDH^-$ MDVs in control cells but not in 3-MA-treated cells.

Figure legends:

Fig.4 j, Representative immunofluorescence images of differentiated brown adipocytes left unstimulated or stimulated with heart-derived DAMPs in the presence or absence of 3-MA, stained with DAPI (blue), TOMM20 (green), and PDH (red). Extracellular TOMM20^{+}PDH^{-} and TOMM20^{+}PDH^{+} MDV puncta per cell were quantified (mean±s.e.m., n =12 (except for 3-MA/Unstimulated group), 15 (3-MA/Unstimulated) cells, unpaired one-way ANOVA test followed by Bonferroni method). Scale bar: 20μm; 2μm (insets).

7. In line 612, authors claims that Becn1 regulates VPS35 recruitment to mitochondria, lacks mechanistic detail. Suggestion: To prove this point, Co-IP experiment or PLA experiment could strengthen the conclusion.

Response:

We are grateful for raising this important mechanistic point. To test whether BECN1 directly interacts with VPS35, we performed a GFP-Trap pulldown assay in HeLa cells co-expressing VPS35-GFP and BECN1-mCherry (Fig. R15). VPS35-GFP was efficiently precipitated by GFP-Trap, but BECN1-mCherry was not detected in the GFP-Trap fraction, indicating that under these conditions BECN1 and VPS35 do not form a stable complex.

Taken together with our subcellular fractionation data showing that heart-derived DAMPs increase VPS35 levels in mitochondrial fractions in a BECN1- and 3-MA-sensitive manner, we conclude that VPS35 mitochondrial recruitment is BECN1-dependent, but most likely regulated indirectly rather than through a stable BECN1-VPS35 interaction. To avoid overstating the mechanistic detail, we have revised the text in Results from “Becn1 regulates VPS35 recruitment to mitochondria” to “BECN1-dependent modulation of VPS35 mitochondrial recruitment”.

a

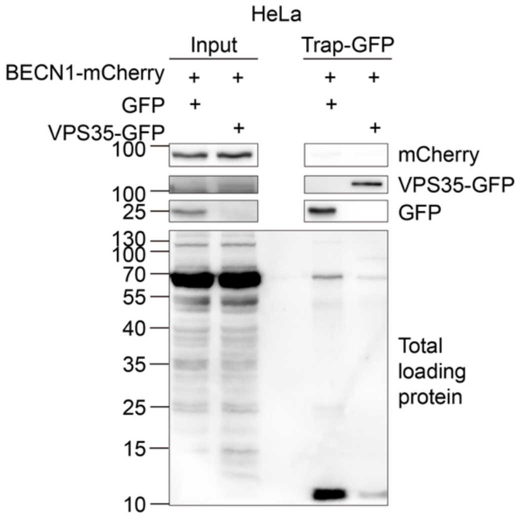

Fig. R15 a, HeLa cells transfected with BECN1-mCherry together with GFP or VPS35-GFP as indicated. Input lysates and GFP-Trap fractions (Trap-GFP) were analyzed by immunoblot for mCherry and GFP.

8. In line 659, author links MDV uptake to increased OXPHOS and IL-10 production (Fig. 6), the role of Complex V proteins remains indirect and conclusion lacks mechanistic evidence. Suggestion: Use of CRISPR- or SiRNA-mediated (e.g., ATP5A1 knockdown) in macrophages to test whether Complex V components are necessary for the observed findings.

Response：

We appreciate this very helpful comment. We agree that, in the original submission, the link between MDV uptake, increased OXPHOS and higher anti-inflammatory cytokine production in macrophages relied primarily on correlative evidence and that the specific contribution of Complex V proteins required more direct testing.

In the revised manuscript, we therefore performed additional mechanistic experiments focusing on Complex V-dependent macrophage polarization and cytokine responses. We first examined the classical polarization markers inducible nitric oxide synthase (iNOS) and arginase-1 (ARG1), which compete for the common substrate L-arginine and act as core metabolic “switch” that determines the inflammation status of macrophages. $iNOS^{high}ARG1^{low}$ defines classically activated macrophage, whereas $iNOS^{low}ARG1^{high}$ macrophages are associated with tissue-repair and anti-inflammatory programmes.

As shown by Western blots and ELISA assays (Fig. R16 a-c), DAMP-activated macrophages treated with WT-MDVs displayed decreased iNOS and increased ARG1 protein, accompanied by higher Il4 mRNA, IL-4 secretion, whereas Becn1-KD MDVs largely failed to induce these changes.

To directly examine the role of complex V, we combined pharmacological inhibition and genetic gain-of-function approaches (Fig. R16 d-g). Pre-treating MDVs with oligomycin to inhibit Complex V before their addition to macrophages prevented the MDV-induced shift in iNOS and ARG1 and markedly blunted IL-4 secretion, despite comparable MDV

exposure. Conversely, ATP5A1 overexpression in macrophages enhanced ARG1 and IL-4 responses to DAMPs, partially mimicking the effect of WT-MDVs. These findings indicate that MDV-driven reprogramming of macrophages is critically dependent on Complex V activity.

The combination of inhibotor and gain-of-function experiments provides direct mechanistic support that Complex V components are required for the full ability of BAT-derived MDVs to enhance OXPHOS and skew cytokine production toward a pro-reparative, anti-inflammatory phenotype.

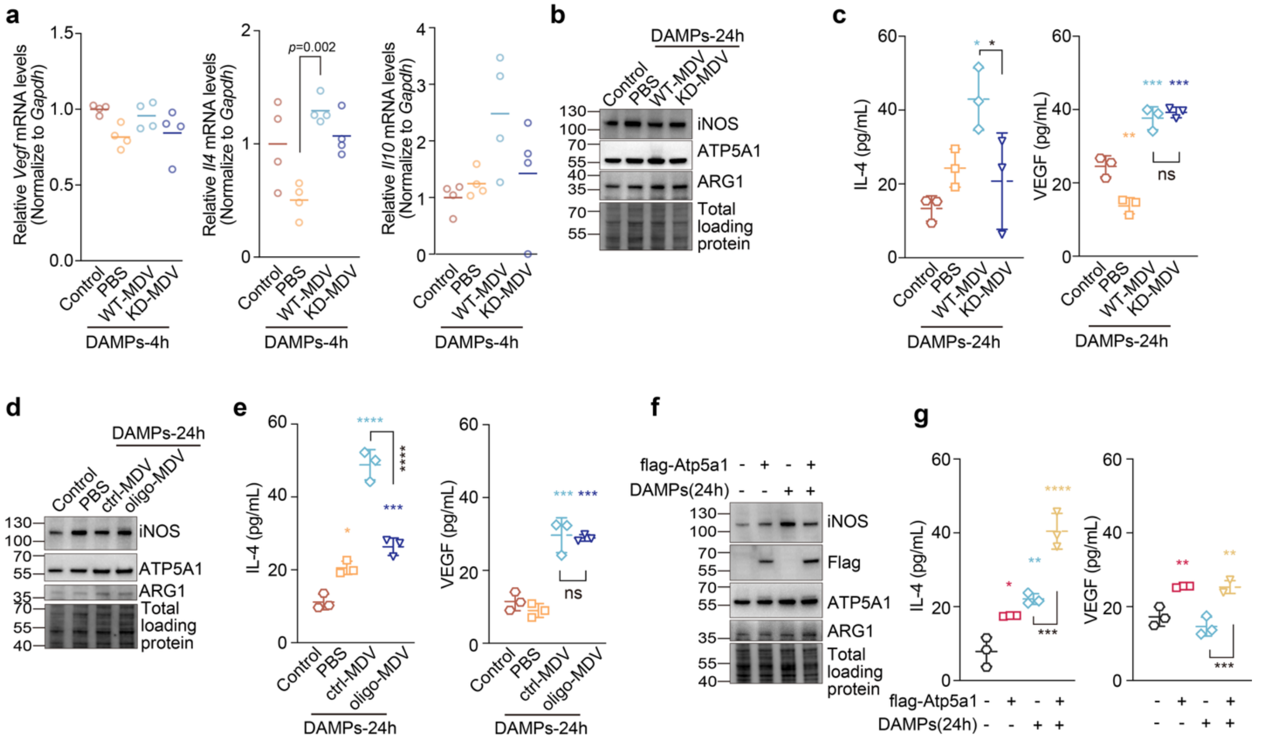

Fig. R16 a, RT-qPCR analysis of pro-reparative cytokine genes (*Vegf*, *Il4*, *Il10*) in iBMDM co-incubated with WT or *Becn1*$^{+/-}$ BAT MDV (mean, n=4 independent experiments, one-way ANOVA test followed by Bonferroni method). **b,** Representative western blots of macrophages treated with DAMPs for 24 h in the presence of PBS, WT-MDVs, or KD-MDVs (n=3 independent experiments). **c,** IL-4 and VEGF concentrations in supernatants from macrophages treated as in (**b**), measured by ELISA. (n=3 independent experiments). **d,** Representative western blots of macrophages treated with DAMPs for 24 h in the presence of PBS-treated MDVs, or oligomycin(100μg/mL)-treated MDVs (n=3

independent experiments). **e,** IL-4 and VEGF concentrations in supernatants from macrophages treated as in (**d**), measured by ELISA. (n=3 independent experiments). **f**, Representative western blots of macrophages infected with flag-Atp5a1 lentivirus and treated with DAMPs for 24 h (n=4 independent experiments). **g,** IL-4 and VEGF concentrations in supernatants from macrophages treated as in (**f**), measured by ELISA. (n=3 independent experiments). **The above new data are included in Fig. 7 in the revised manuscript.**

9. In previous study by Rosina et al, 2022 demonstrated that EV-derived from the BAT contain oxidatively damaged mitochondrial components and are primarily destined for degradation to avoid failure of the thermogenic program. Please discuss. Further, please clarify whether their MDVs represent a distinct subpopulation from those described by Rosina et al., or whether there is functional heterogeneity within BAT-derived vesicles.

Response:

We sincerely the appreciate your comment that draws our attention to the study by Rosina et al. (Cell Metab. 2022, PMID: 35305295), which is indeed highly relevant to our work. Rosina and colleagues showed that, under cold stress, BAT releases mitochondria-containing EVs/MDVs that are taken up mainly by two cell types within BAT, brown adipocytes and tissue-resident macrophages. Acceptance of these sEVs by brown adipocytes leads to impaired oxidative metabolism and downregulation of thermogenic gene expression, thereby acting as a negative feedback on the thermogenic process (Cell Metab. 2022, Fig. 3).

In parallel, BAT-resident macrophages uptake these BAT EVs and acquire an M2-like signature with increased expression of CD206 and CD200R following cold stress. Macrophage subsets analysis suggested the transition from inflammatory monocytes to BAT-resident macrophages under cold-exposure (Cell Metab. 2022, Fig. 4).

Together, their data support a model in which BAT sEVs are part of a quality-control circuit that both protects brown adipocytes from mitochondrial damage and shifts resident macrophages toward a tissue-remodelling, anti-inflammatory phenotype.

In our study, we focus on BAT-derived MDVs that traffic to the injured heart, where the predominant recipient population is macrophages rather than parenchymal cardiomyocytes. Similar to the M2-like shift described for BAT macrophages in Rosina et al., we find that cardiac macrophages taking up BAT-MDVs in the post-MI setting exhibit enhanced anti-inflammation features, including increased IL-4 production and release, higher ARG1 level. Thus, although the anatomical context differs (BAT vs. heart), both studies converge on the concept that BAT-derived mitochondrial vesicles

are potent immunometabolic modulators that bias macrophages toward an M2-like, tissue-repair phenotype.

Based on the available evidence, we favour a model in which the BAT MDVs analyzed in our study represent a mitochondria-enriched subpopulation within the heterogeneous EV pool described by Rosina et al. Rosina et al. performed proteomic profiling on bulk BAT sEVs recovered from the interstitial space, thereby capturing the composite cargo of all vesicles released from BAT. Accordingly, their most abundant proteins include both mitochondrial and non-mitochondrial origin, consistent with a mixed vesicle population.

[FIGURE REDACTED]

Ref: Rosina et al, Cell Metab. 2022, PMID: 35305295.

In contrast, we subjected BAT samples to cell disruption folloswed by differential centrifugation to purify mitochondria-derived vesicles, and proteomic analysis of this enriched MDV fraction (Fig. 6a) shows that its most abundant proteins are dominated by

mitochondrial and OXPHOS components, with detectable but low levels of TOMM20, PDH et al. These methodological and compositional differences support the view that our BAT-MDVs correspond to a specific, bioenergetical-rich subset of BAT-derived vesicles

We now discuss explicitly in the revised Discussion and propose that BAT generates multiple mitochondrial vesicle subpopulations: (i) oxidative mitcohondiral matrix proteins (PDH) (as described by Rosina et al.) and (ii) OXPHOS-enriched MDVs, both prone to be uptaken by macrophages and regulate immunometabolic functions. Future single-vesicle and in vivo tracking studies will be required to fully map the relationships among these vesicle subsets.

This model is also consistent with the macrophage data. Bulk BAT-sEV preparations contain a fraction of the OXPHOS-rich MDVs described here, macrophages exposed to total BAT EVs likewise adopt an anti-inflammatory, pro-reparative phenotype.

Manuscript edits: “Discussion”

Our data are consistent with the view that the BAT-MDVs analyzed here represent a mitochondria-enriched subpopulation within the BAT-EV pool described by Rosina et al. Whereas Rosina et al. characterized bulk BAT EVs carrying oxidatively damaged mitochondrial components (identified by PDH^{+}) involved in local mitochondrial quality control, our MDV-enriched preparations display a proteome dominated by intact OXPHOS, including Complex V, and anti-inflammatory features in cardiac macrophages. The observation that total BAT-EV preparations also promote an anti-inflammatory and pro-reparative macrophage phenotype is compatible with the presence of such OXPHOS-rich MDVs within bulk EVs. Further single-vesicle and in vivo tracking studies will help delineate the composition and functional relationships of these BAT-derived vesicle populations.

10. Appropriate control experiments to BAT pericardial transplantation is lacking. Authors should at least transplant PBS or carrier solutions, or WAT.

Response：

Thank you for highlighting the need for appropriate controls for the pericardial BAT transplantation experiments.

In the revised manuscript, mice were randomized to: MI, MI with BATectomy (MI–BAT), MI with pericardial BAT graft (MI+BAT), MI with pericardial WAT graft (MI+WAT), or MI with cold-activated BAT graft (MI+cold BAT). All groups were followed by serial echocardiography (baseline, 7, 14 and 28 days) and 28-day histology with quantified fibrosis. BAT and cold BAT transplantation significantly improved LV ejection fraction and fractional shortening over 4 weeks and reduced fibrotic area at day 28, whereas WAT transplantation did not improve systolic function or scar burden compared with MI controls. Conversely, BAT-deficient mice (MI-BAT) exhibited worse LV function and larger fibrotic areas than MI controls (Fig. R17).

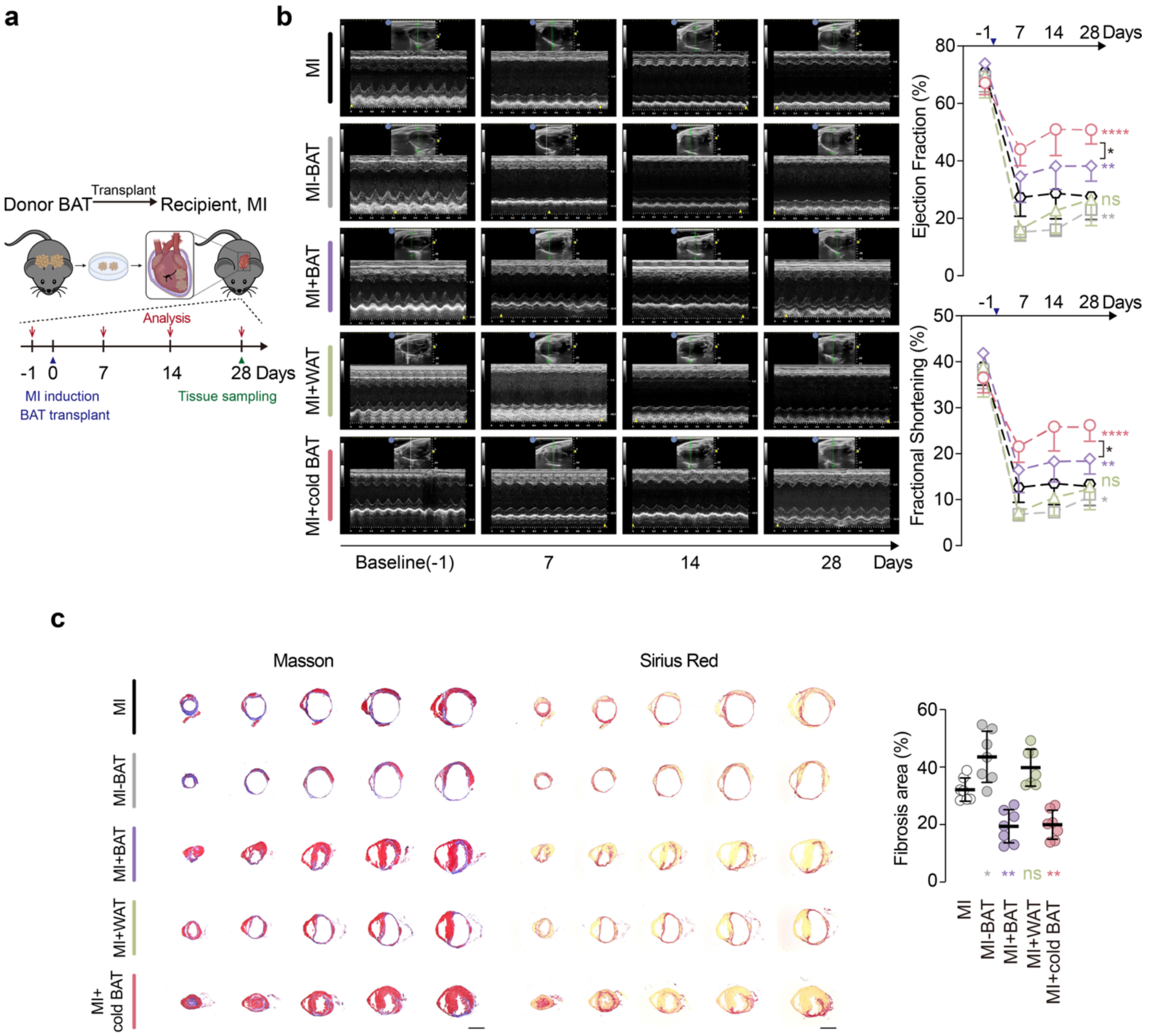

Fig. R17 **a**, Experimental timeline for MI induction, adipose tissue pericardial transplantation, and outcome assessments. Echocardiography was performed at baseline (day −1) and on days 7, 14, and 28. Hearts were harvested on day 28 for Masson's trichrome and Sirius Red staining. **b,** Representative B-mode and M-mode echocardiography of BAT-receiving mice at baseline and days 7, 14, and 28 (**c**). Ejection fraction and fractional shortening were quantified (**d**) (mean±s.d., n=7, repeated two-way ANOVA and Holm-Sidak post hoc, *p < 0.05, **p < 0.01, ****p < 0.0001). **c,** Representative Masson and Sirius Red staining images of mouse hearts at day 28. Fibrosis percentage was quantified (mean±s.d., n=7, One-way ANOVA test followed by Bonferroni method, *p

< 0.05, **p < 0.01, ***p < 0.001, ****p < 0.0001). Scale bar: 2mm. **The above new data are included in Fig. 1 in the revised manuscript.**

11. It is unclear to this reviewer why both male and female mice were used as BAT donors? Sex differences in brown adipose tissue are well documented. This could include adipose tissue mitochondrial function and metabolic flexibility (https://www.nature.com/articles/s41366-021-00843-0).

Response:

We are grateful for raising this point and fully agree that sex differences in BAT biology, including mitochondrial function and metabolic flexibility, are well established.

In the original submission, female mice were used only in the first BAT transplantation experiment (Fig. 1c-f) to potentially test whether the cardioprotective effect of BAT was affected by sex. In that experiment, MI recipient mice receiving male vs. female BAT grafts showed no significant differences in LV function or infarct fibrosis, and the data were therefore pooled. Based on this result, all subsequent in vivo experiments in the original manuscript use male BAT donors and male MI recipient mice.

In the revised manuscript, all BAT donor and MI recipient mice are now male, and we have updated the Methods section. This adjustment ensures that potential sex-related variability in BAT biology does not confound the interpretation of our transplantation and MDV experiments in the current study.

Specific comments to figures:

1c: It is uncommon to see such massive LV dilation, even in permanent ligation. Further, parts of the valves are visualized on the baseline images, suggesting that echocardiography measurements were not carried out with the same imaging window and angle.

1d: Baseline data is not quantified nor shown.

Response：

Thank you for bringing up these important points.

First, we agree that the degree of LV dilation in the original Fig. 1c appeared more severe than usually reported for permanent LAD ligation. In the initial experiments we used a high-level LAD ligation, which produced large infarcts and marked LV remodeling. In the revised manuscript, all in vivo functional and structural experiments have been repeated using a mid-LAD ligation, resulting in infarct size and LV dilation that are within the normal range. The representative images in Fig. 1 and the corresponding functional data have been updated accordingly.

Second, we would like to clarify the echocardiographic acquisition protocol. All quantitative measurements (EF, FS, LV dimensions) were obtained from a standard parasternal short-axis view at the mid-papillary level using M-mode, with fixed imaging depth and gain settings. The structures visible in the baseline images of the original figure are papillary muscles rather than valve leaflets, which are sometimes difficult to avoid in this view and are also seen in many published mouse echocardiography images.

[FIGURE REDACTED]

Ref: Nat Commun. 2020. PMID: 33328477

[FIGURE REDACTED]

Ref: Circulation. 2022. PMID: 35430895

In the revised manuscript, we have clarified the detailed echocardiogram protocols in the Methods. Also, we have added representative echo images and quantified data for each in vivo cardiac function experiments in the revised manuscript (Fig. R17).

Third, baseline LV function was assessed in all animals 1 day before LAD ligation. In the original submission, these baseline values were acquired but not shown. In the revised version, we now display and quantify baseline EF and FS for all groups (Fig. R17).

2: Author did not quantify MDVs. It would be more informative if the author quantifies the MDVs through flow cytometer. Additionally, the images look better with proximity ligation assay than IHF staining. To confirm that the MDVs rescue fibrosis, the author should stain for the Masson staining on the heart section post MI.

2c/2g: Labeling of images could be improved. Control antibody stainings are missing.

2g: The uptake of BAT(Zs-green) in macrophages is not convincing

Response:

(1) MDV quantification

We appreciate your suggestion to better quantify MDVs. In our study, flow cytometry was used in a limited manner to assess MDVs in cell-culture supernatants, where the vesicle suspension is relatively clean. We did not extend this approach to enzymatically digested heart tissue, because infarcted myocardium digestion generates abundant subcellular debris and fragmented membranes that overlap in size and scatter with MDVs, making reliable quantification on conventional flow cytometer technically difficult. Nano-flow platforms would be better for this application but are not available to us currently.

To ensure consistent and quantitative dosing of MDVs, we adopted a total-protein-based approach, which is commonly used in the extracellular vesicle field. MDV preparations from different adipose depots or genotypes were quantified by BCA assay, and in both in vivo and in vitro experiments we applied fixed MDV protein doses regardless of source.

(2) Image quality improvement

Thank you for these constructive comments on the imaging data and the evidence for macrophage uptake. In the revised manuscript, we have substantially strengthened both the experimental pipeline and the figure presentation.

First, in response to the suggestion that a proximity ligation assay (PLA) would provide higher specificity than conventional immunofluorescence, we have performed PLA using

two independent GFP antibodies to detect mtGFP-derived signal. This assay yields discrete PLA puncta within infarct area in BAT^{mtGFP}-recipient hearts, whereas non-transplanted and non-fluorescent BAT controls show no PLA signal (Fig. R18).

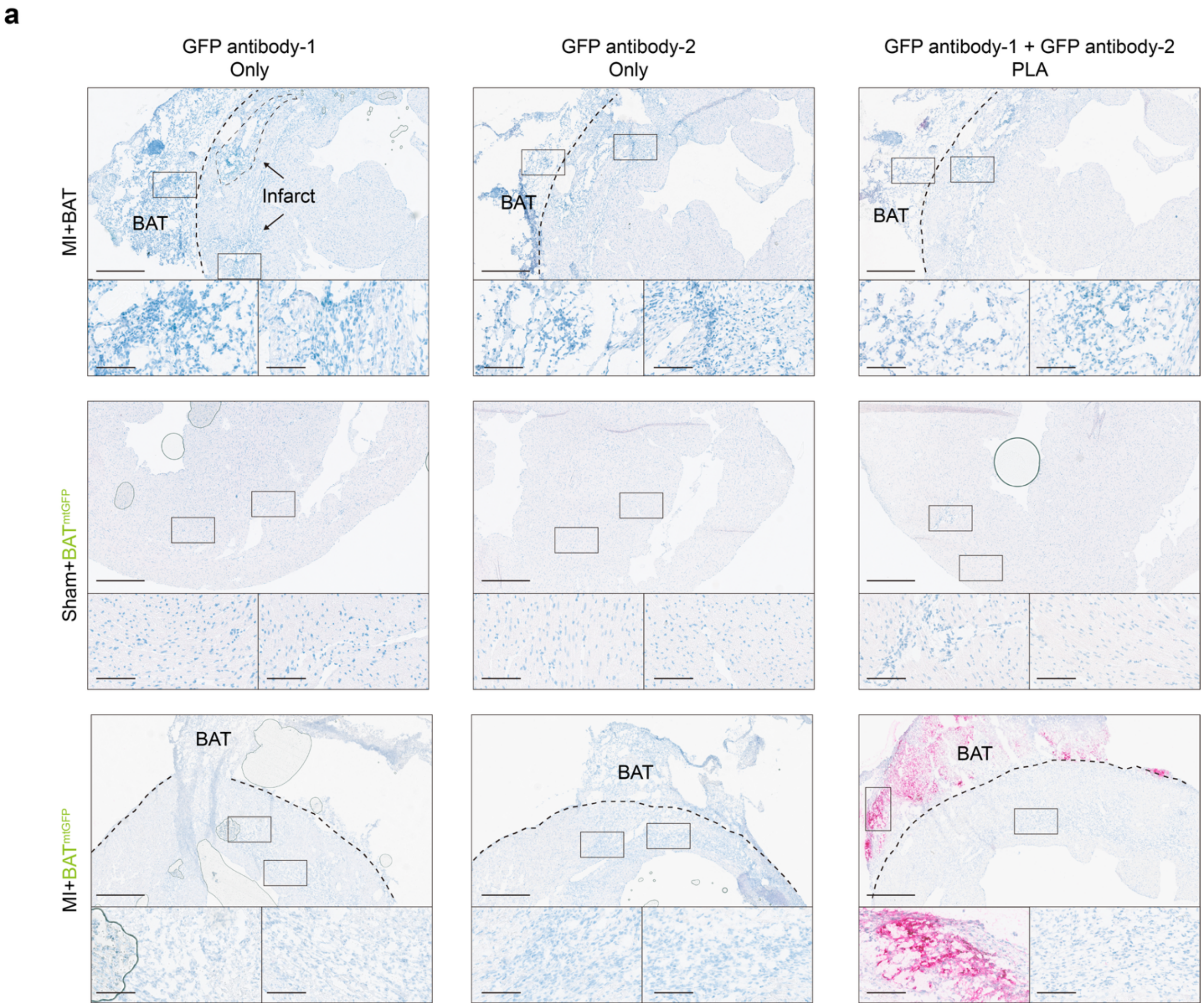

Fig. R18 a, Proximity ligation assay (PLA) for GFP in hearts from MI mice receiving unlabeled BAT (MI + BAT), sham-operated mice receiving BAT^{mtGFP} (Sham+ BAT^{mtGFP}) and MI mice receiving BAT^{mtGFP} (MI+ BAT^{mtGFP}). Sections were incubated with GFP antibody-1 alone, GFP antibody-2 alone, or with both GFP antibodies, followed by PLA detection. Scale bar: 500μm; 50μm (magnification). **The above new data are included in Extended Data Fig. 3 in the revised manuscript.**

Second, to directly address the concern that uptake of BATosome by macrophages was not convincing, we performed flow cytometric analysis of dissociated hearts from $BAT^{ZsGreen}$-recipient and control mice. After gating on cardiac immune cells, we quantified $ZsGreen^{+}$ cells and determined their lineage composition. The analysis shows that the majority of $ZsGreen^{+}$ immune cells are macrophages, with smaller fraction are neutrophils (Fig. R19).

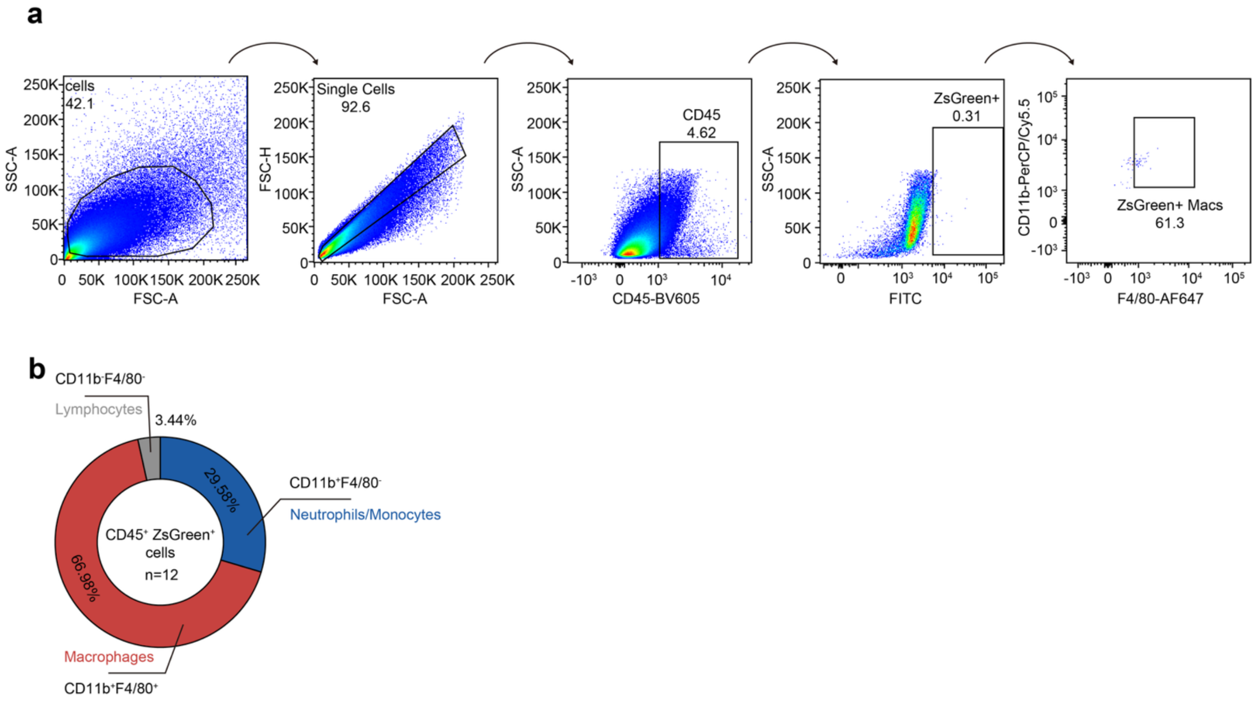

Fig. R19 a, Flow-cytometric gating strategy for identifying BATosome-recipient immune cells in infarcted hearts from $BAT^{ZsGreen}$-receiving mice at MI 4d. After debris exclusion and singlets selection, leukocytes were gated as $CD45^{+}$ cells, followed by $ZsGreen^{+}$ events and $CD11b^{+}F4/80^{+}$ macrophages within the $CD45^{+}ZsGreen^{+}$ population. **b**, Quantification of immune cell subsets among $ZsGreen^{+}CD45^{+}$ cells in infarcted hearts from $BAT^{ZsGreen}$-receiving mice at MI 4d. Data are presented as mean percentages of each subset among total $ZsGreen^{+}CD45^{+}$ cells (n as indicated). **The above new data are included in Fig. 2 in the revised manuscript.**

Third, we have optimized the immunofluorescence protocol and re-acquired key images for both $BAT^{ZsGreen}$ and BAT^{mtGFP} transplantation experiments. For the mitochondrial reporter, we now detect mtGFP with a GFP-specific antibody rather than relying on autogeneous GFP fluorescence, which improves detecting sensitivity. For all stainings, we apply an autofluorescence-quenching reagent (TrueBlack Plus Lipofuscin Autofluorescence Quencher, Biotium) to minimize background signal from infarcted myocardium. Second antibody controls were presented in the figure (Fig. R20).

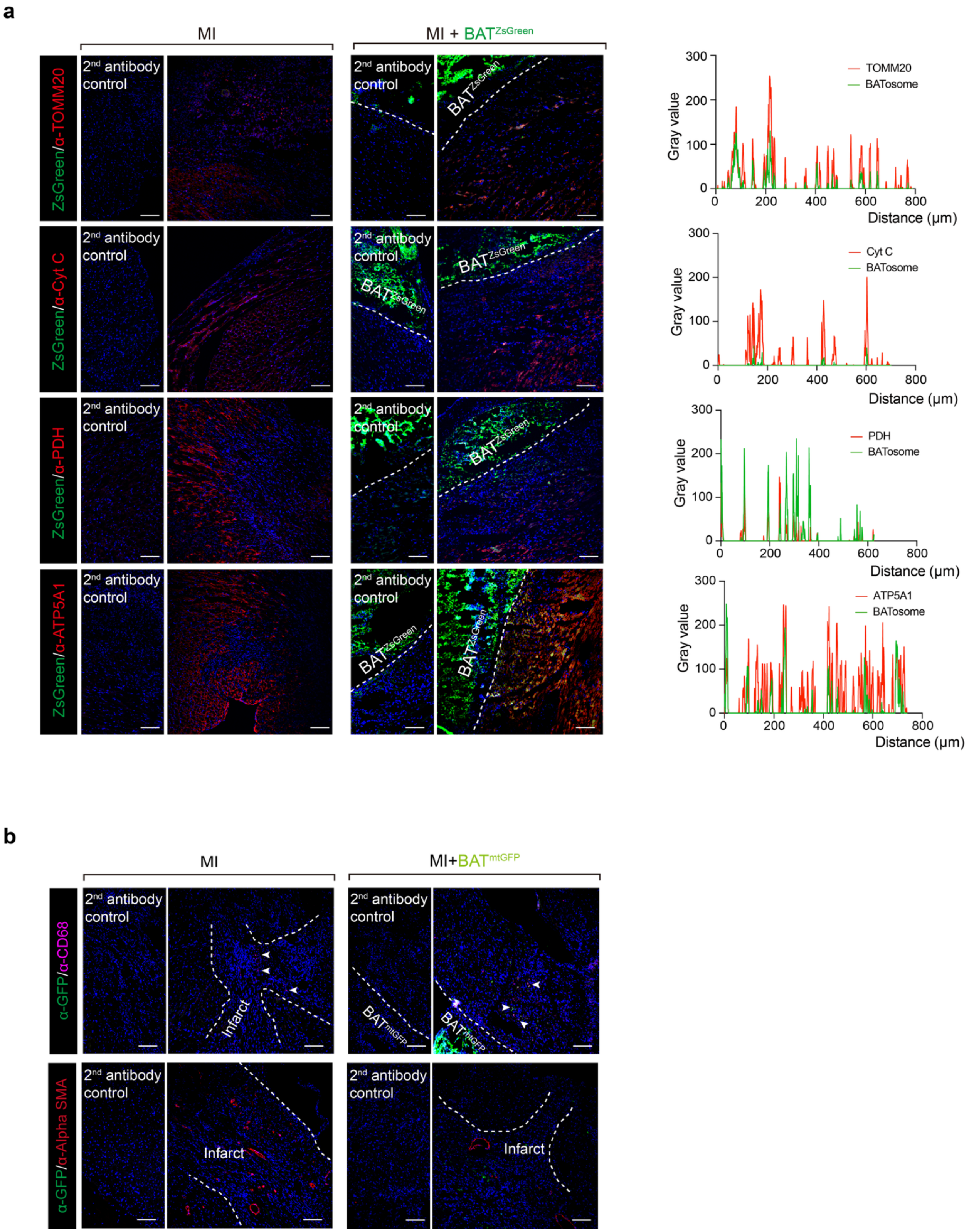

Fig. R20 a, Representative confocal images of left ventricles in BAT^{ZsGreen}-receiving mice at MI 4d, stained for TOMM20 or Cytochrome C or PDH or ATP5A1(red), and

nuclei (DAPI, blue). Co-localization analysis of ZsGreen^{+} BATosomes with mitochondrial markers in the infarct myocardium. Scale bar: 100μm. **b,** Immunofluorescence validation of mtGFP signal in infarcted hearts of BAT^{mtGFP}-receiving mice. Sections were stained for GFP (green) with CD68 (macrophages) or α-SMA (fibroblasts) (red). Scale bar: 100μm. **The above new data are included in Extended Data Fig. 2 and 3 in the revised manuscript.**

Fourth, we would like to clarify that the original immunofluorescence experiments included appropriate biological controls, including non-transplanted hearts and hearts transplanted with non-fluorescent BAT, which served as negative controls. Each slide also contained a secondary-antibody-only section. The secondary antibody control section was imaged first and used to define the maximal laser power and PMT settings at which no visible signal was detected. These settings were then applied to all experimental sections on the same slide to limit overexposure and nonspecific background. Also, in the revised figures, we have refined panel labels, color keys and scale bars to clarify signal identity and localization. Brightness and contrast were adjusted to improve visual readability (Fig. 2, Extended Data Fig. 2, Extended Data Fig. 3).

(3) MDV rescue in vivo experiments

To address this concern directly, we repeated the in vivo MDV treatment experiments using a dose-normalized design in which MDVs from BAT and WAT were administered at the same total MDV protein dose. MDVs were isolated from BAT or WAT, quantified by BCA assay, and injected intravenously at 30 μg MDV protein per mouse after MI. Serial echocardiography was performed at baseline and at 7, 14, and 28 days post-MI to assess LV function (EF and FS).

In this dose-normalized setting, both BAT- and WAT-derived MDVs preserved cardiac functions**.** Compared with MI controls, the BAT-MDV and WAT-MDV groups showed significantly higher EF and FS at all post-MI time points examined, with no appreciable difference between the two MDV sources (Fig. R21a). Thus, when MDV dosage is

matched by total protein, WAT-MDVs are also cardioprotective and the previously weaker effect of WAT-MDVs in our original submission is most likely attributable to lower MDV dose rather than an intrinsic efficacy.

To complement the functional readouts, we also performed systematic fibrosis quantification at day 28. In line with the echo data, both BAT-MDV and WAT-MDV treatment significantly reduced LV fibrosis compared with MI controls, with comparable protection (Fig. R21b). These experiments support the conclusion that, under protein-normalized dosing, MDVs from both BAT and WAT confer similar benefits on post-MI cardiac function and structural remodeling.

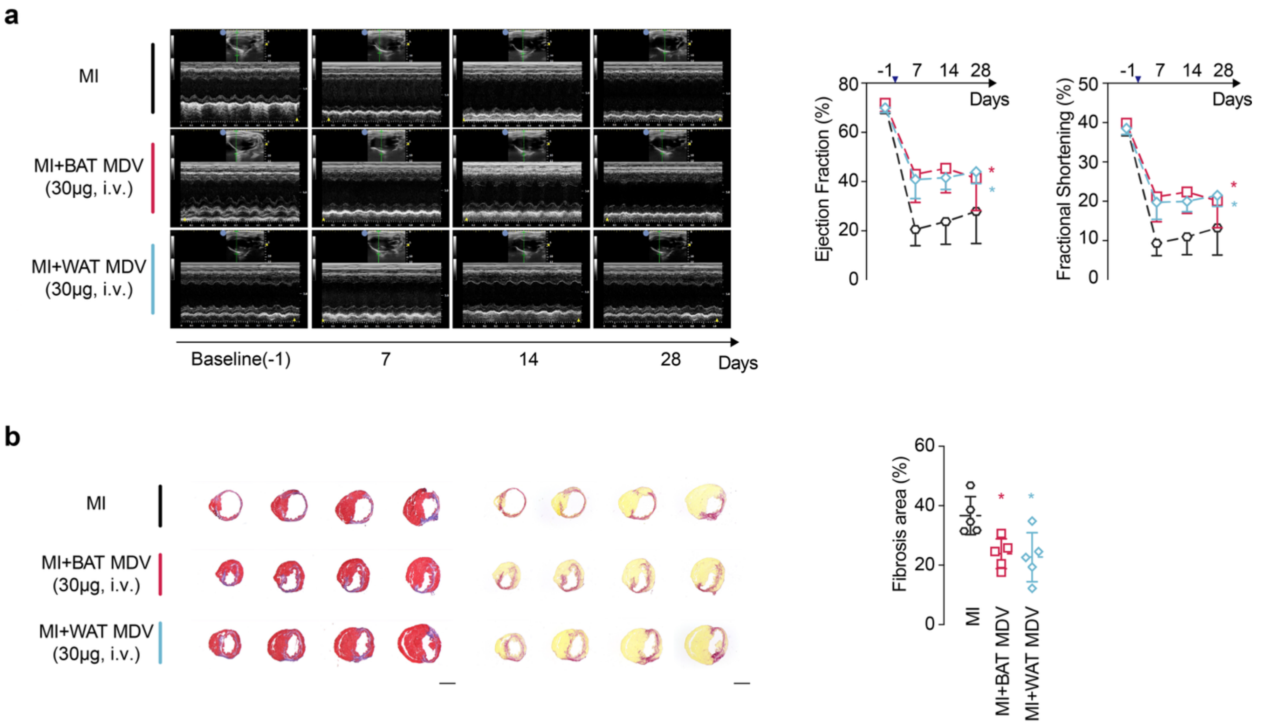

Fig. R21 a, Representative B-mode and M-mode echocardiography of adipose tissue-derived MDV-treated mice at baseline and days 7, 14, and 28. Ejection fraction and fractional shortening were quantified (mean±s.d., n=5, repeated two-way ANOVA and Holm-Sidak post hoc, *$p < 0.05$). **b,** Representative Masson and Sirius Red staining images of MDV-treated mouse hearts at day 28. Fibrosis percentage was quantified (mean±s.d., n=5, One-way ANOVA test followed by Bonferroni method, *$p < 0.05$).

Scale bar: 2mm. **The above new data are included in Fig. 3 in the revised manuscript.**

3d: Fibrosis quantification at day 4 after infarction does not make any sense. This readout is conducted way too early in the course of postinfarct remodeling. Authors showed in figure 1f that WAT increases fibrosis, resulting in larger fibrotic area than in controls. Therefore, authors should compare BAT-MDV also to controls (not only to WAT-MDV).

Response：

We appreciate this insightful comment and agree that day-4 histology is too early to make meaningful quantification. In the revised manuscript we have therefore removed day 4 fibrosis quantification and shifted our primary structural analysis to a late remodeling time point (day 28).

Specifically, we repeated the MDV treatment experiment with an improved design in which hearts were collected at day 28 post-MI, and BAT- and WAT-derived MDVs were administered at a dose-normalized level (30 µg MDV protein per mouse, i.v.).

In this setting, both BAT-MDV and WAT-MDV groups exhibited significantly lower fibrosis than MI controls. Both BAT-MDV and WAT-MDV treatment significantly reduced LV fibrosis compared with MI controls (Fig. R21a).

5b, the immunostaining images should be replaced with higher magnification image that corresponds well with the quantification (in Fig 5c).

Response：

We appreciate this helpful suggestion. In the revised manuscript, we have replaced the original low magnification immunostaining images in Fig. 5b with new higher magnification and better resolution confocal images acquired from the same regions that were used for quantitative analysis in Fig. 5c. The new panels provide clearer visualization of the structure.

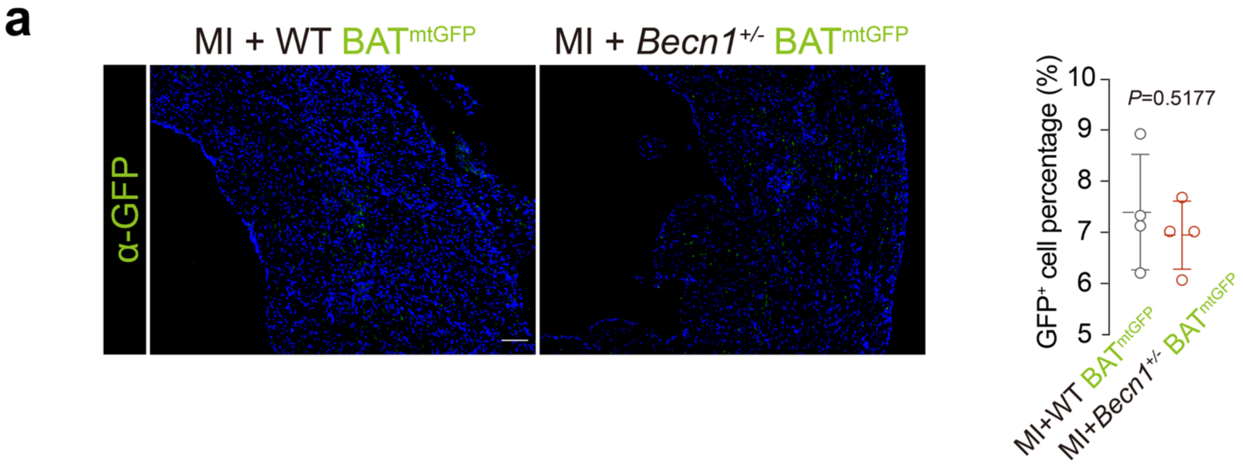

Fig. R22 a, Representative confocal images of BAT^{mtGFP}-receiving mice at MI 4d, stained for GFP (green) and nuclei (DAPI, blue). Scale bar: 100μm. Mt-$EGFP^{+}$ cell percentage was quantified (mean±s.d., n=4, unpaired Student's t-test). **The above new data are included in Fig. 5 in the revised manuscript.**

6: Label y axis in plot a; KD equivalent to Becn1+/- mice in plot c and j, not clear in image legend? Please check. Additionally, the bulk analysis was derived from which mouse strain? WT-MI, WT- MI+BAT transplant?

Response:

Thank you for these helpful detailed comments.

First, we have added an explicit y-axis and x-axis labels (now Fig,7a), indicating the y variable (Il1b, Arg1, and Vegf mRNA relative levels) and x (DAMPs treated time).

Second, we have clarified that "KD" referred to "*Becn1*$^{+/-}$" in panels (c) and (j) in Figure legends and in the Results.

Third, we have specified the mouse strain and experimental groups for the bulk RNAseq analysis shown in panel (l). The bulk dataset was derived from wild-type mice subjected to MI with or without BAT transplantation (WT-MI and WT-MI+BAT), as now stated in the figure legend and in the Methods section of bulk RNAseq analysis.

Extended Data Fig. 3: The uptake of BAT(Zs-green) in macrophages is not convincing. This could in part be autofluorescence. Control stainings are lacking. Further, this reviewer suggests quantification of BAT uptake by flow cytometry, i.e. quantification of ZS-green+ macrophages.

Response：

We are grateful for this helpful comment. In the revised manuscript, we have addressed both the autofluorescence concern and the need for quantitative analysis.

First, we repeated the immunofluorescence staining with biological controls (non-transplanted hearts and hearts transplanted with non-fluorescent BAT) and technical controls (secondary-antibody-only sections on each slide). We also used an autofluorescence-quenching reagent to reduce background signal in infarcted myocardium and acquired new, higher-magnification confocal images (Fig. R20).

Second, we have performed flow cytometric quantification of $ZsGreen^{+}$ macrophages in hearts from $BAT^{ZsGreen}$-recipient and control mice. After gating on live $CD45^{+}$ $ZsGreen^{+}$ cells, we quantified the fraction of $ZsGreen^{+}$ macrophages. The analysis show that $ZsGreen^{+}$ cells are mainly macrophages (60%+), providing quantitative support for BATosome uptake by cardiac macrophages (Fig. R19).

Extended Data Fig. 8: “transient macrophages” is not a common denominator of macrophage populations. Recent scRNA-sequencing dataset of cardiac myeloid populations in mouse myocardial infarction models have provided clear nomenclature for immune cells, e.g. inflammatory/antigen-presenting/etc..

Response:

Thank you for drawing attention to the terminology.

We agree that “transient macrophages” is less recognized than canonical macrophage subset markers, which might be confusing to readers.

Macrophage subsets are now annotated using widely accepted subset markers (Nat Rev Cardiol. 2025. PMID: 397435). On this basis, the iBMDMs stimulated with cardiac DAMPs exhibits a transcriptional profile similar to that of $CCR2^+$ inflammatory macrophage subset (Fig. R23).

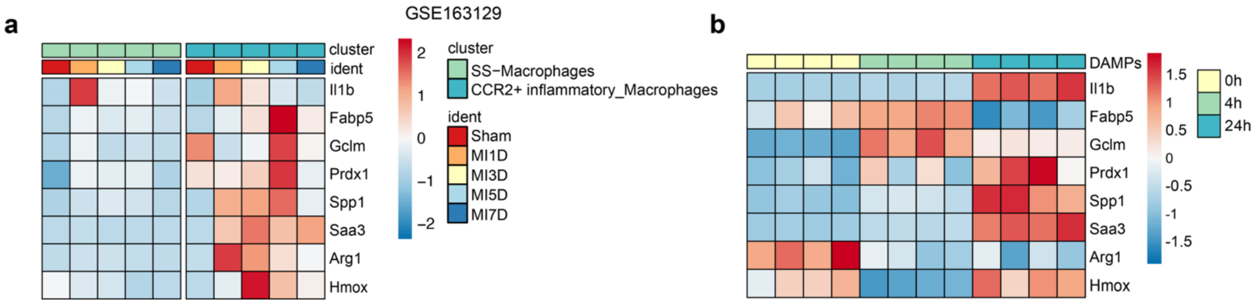

Fig. R23 DAMPs-stimulated iBMDM as a cell model for macrophages in MI.
a, Genes with high expression specifically in $CCR2^+$ inflammatory macrophages, the major macrophage subpopulation during MI 3d-5d. The data were from mouse single-cell RNA sequencing (GSE163129). SS-Macrophage, steady-state macrophage.
b, RT-qPCR analysis of transient macrophage signature genes in DAMPs-stimulated iBMDM cells at 4 hours and 24 hours (n=4 independent experiments). **The above new data are included in Extended Data Fig. 9 in the revised manuscript.**

Extended Data Fig. 10: Data annotation and representation could be improved.

Response：

Thank you for this helpful suggestion regarding data representation. The underlying dataset includes all subunits of the five OXPHOS complexes, which made the original figure dense and difficult to read. In the revised version, we have simplified the visualization by displaying only the eight most highly expressed subunits for each complex (Fig. R24).

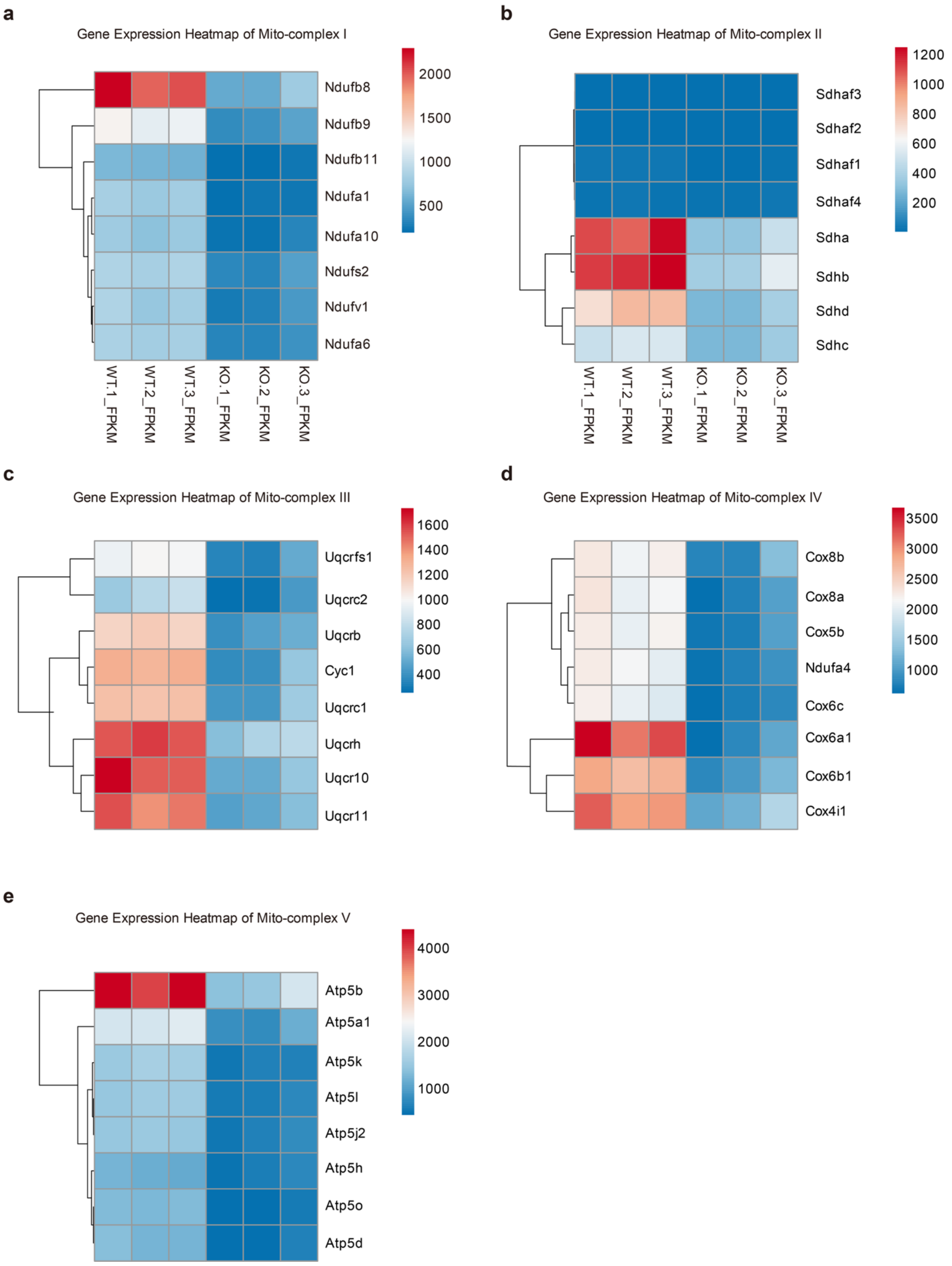
a
Gene Expression Heatmap of Mito-complex I
Ndufb8
Ndufb9
Ndufb11
Ndufa1
Ndufa10
Ndufs2
Ndufv1
Ndufa6
2000
1500
1000
500
WT.1_FPKM
WT.2_FPKM
WT.3_FPKM
KO.1_FPKM
KO.2_FPKM
KO.3_FPKM
b
Gene Expression Heatmap of Mito-complex II
Sdhaf3
Sdhaf2
Sdhaf1
Sdhaf4
Sdha
Sdhb
Sdhd
Sdhc
1200
1000
800
600
400
200
WT.1_FPKM
WT.2_FPKM
WT.3_FPKM
KO.1_FPKM
KO.2_FPKM
KO.3_FPKM
c
Gene Expression Heatmap of Mito-complex III
Uqcrfs1
Uqcrc2
Uqcrb
Cyc1
Uqcrc1
Uqcrh
Uqcr10
Uqcr11
1600
1400
1200
1000
800
600
400
d
Gene Expression Heatmap of Mito-complex IV
Cox8b
Cox8a
Cox5b
Ndufa4
Cox6c
Cox6a1
Cox6b1
Cox4i1
3500
3000
2500
2000
1500
1000
e
Gene Expression Heatmap of Mito-complex V
Atp5b
Atp5a1
Atp5k
Atp5l
Atp5j2
Atp5h
Atp5o
Atp5d
4000
3000
2000
1000

Fig. R24 a-e, Heatmap showing the differentially expressed genes (DEGs) of mitochondrial respiratory chain complex I-V top8 highly expressed subunits in WT and *Becn1*$^{+/-}$ BAT. The data were from mouse BAT bulk RNA sequencing (GSE148275). **The above new data are included in Extended Data Fig. 7 in the revised manuscript.**

Reviewer #3 (Remarks to the Author):

Xiang and colleagues identify a novel role for brown adipose tissue (BAT) in cardioprotection through the release of mitochondria-derived vesicles (MDVs) following myocardial infarction (MI). These MDVs, enriched in mitochondrial membrane components, are taken up by cardiac macrophages, where they promote an anti-inflammatory and reparative phenotype. The authors propose that MDVs is generated by mitochondrial recruitment of VPS35, a process dependent on Becn1. Loss of Becn1 disrupts MDV biogenesis and abolishes BAT's protective effect. Proteomic profiling reveals enrichment of electron transport chain Complex V proteins in MDVs, which enhances oxidative phosphorylation and cytokine production in recipient macrophages. Therapeutically, administration of BAT-derived MDVs attenuated post-MI fibrotic remodeling. While the manuscript explores an intriguing interorgan communication mechanism, several methodological and interpretative issues need to be addressed to strengthen the work.

Response：

We thank you sincerely for your thoughtful and comprehensive evaluation of our work and for clearly articulating both the conceptual strengths and the methodological and interpretative limitations of the original submission. We are encouraged that you view the work as an intriguing inter-organ communication mechanism in which BAT-derived MDVs, enriched in mitochondrial membrane and Complex V components, are taken up by cardiac macrophages to induce an anti-inflammatory and reparative phenotype and to attenuate post-MI remodeling.

In response to your methodological concerns, we have undertaken a substantial experimental revision. We repeated and extended the in vivo studies using a mid-LAD MI model with increased and balanced sample sizes and prolonged follow-up to 28 days. Also, we improved imaging quality, including optimized immunofluorescence, PLA for mtGFP, and flow-cytometric quantification of ZsGreen^{+} cell types.

To strengthen the mechanistic link between MDV cargo and macrophage function, we incorporated new loss- and gain-of-function experiments targeting Complex V and the BECN1-VPS35 axis in BAT, and we added protein- and cytokine-level validation of macrophage polarization (iNOS/ARG1, IL-4, VEGF).

Throughout the Results and Discussion, we have refined the text, added clear summary for each block to make sure that conclusions are proportionate to the supporting data. We hope that these extensive revisions address your main methodological and interpretative concerns and substantially strengthen the manuscript.

Major comments:

(1) The Authors suggest an interorgan BAT-heart crosstalk via new mediator MDVs. BAT has the beneficial effects on cardiovascular diseases, such as coronary artery disease, heart failure, and hypertension (Nat. Med. 2021,27,58-65). The concept about mitochondrial vesicle trafficking and intercellular mitochondrial exchange, has also been previously described (Cell Metab. 2021;33(9):1853-1868, Cell Metab. 2022; 34(10): 1499-1513, Cell Transplant. 2023:32:9636897221148457). These previous reports limit the conceptual novelty of this manuscript. The authors fail to establish how BAT-derived MDVs uniquely advance this field.

Response：

We are very grateful for this comment and fully acknowledge the importance of work you mentioned.

Becher et al have shown in humans that the presence of BAT is associated with a lower prevalence of cardiometabolic and cardiovascular diseases, including coronary artery disease, heart failure and hypertension, thereby establishing BAT as an organ clinically relevant to cardiovascular risk (Nat Med. 2021. PMID: 33398160).

Crewe and colleagues demonstrated that mitochondria-containing small extracellular vesicles released from stressed adipocytes are taken up by cardiomyocytes and trigger adaptive oxidative responses (Cell Metab. 2021 PMID: 34418352).

Borcherding et al. systematically mapped the transfer of mitochondria from adipocytes to other cell types, which is inhibited by high-fat diet, diverting adipocyte mitochondria into the blood and to distal organs such as the heart. (Cell Metab. 2022. PMID: 36070756)

Mori et al. reported that transplantation of adipose-derived mesenchymal stem cells (AD-MSCs) in rat models of ischemic heart disease leads to improved cardiac function. The blockade of mitochondrial transfer attenuates these benefits, identifying mitochondrial transfer as a mechanism of MSC-based cardioprotection. (Cell Transplant. 2023. PMID: 36624995).

Based on these reported mitochondria transfer, our work extended and refined this emerging framework in several specific aspects.

Aspect	Previous work	Our work
Physiological context	Chronic metabolic stress.	Acute MI
Vesicle identity and biogenesis	Mitochondria-containing EVs; Crude EV preparations;<br><br>Incomplete understanding of biogenesis mechanism.	Structurally, biochemically and functionally characterized MDVs.<br><br>BECN1-VPS35-dependent mechanism in response to danger signals.
Recipient cell type and mechanism	Cardiomyocytes; Adipose-resident immune cells (especially macrophages).	Cardiac macrophages; Macrophage polarization and tissue repair mechanism.
Cargo and function	Bulk cargo; Overall functions.	Complex V and subunit ATP5A1; Gain- and loss-of-function validation.
Disease relevance	CVD risk/prevalence; Obesity; Cold adaption.	MI prognosis

Our work extends the emerging concept of adipose-heart mitochondrial communication by characterizing BAT MDVs structurally, biochemically and functionally. Also, the BECN1-VPS35-dependent MDV biogenesis and release process was identified. Therefore, we show that BAT-MDVs are selectively enriched for Complex V-ATP synthase subunits, retain ATP synthase activity, and that pharmacologic Complex V inhibition blunt MDV-induced benefits on macrophage function and cardiac remodeling, indicating that Complex V-rich bioenergetic units are a necessary component of the protective MDV signal. Third, we explained that MDV uptake reprograms boost

macrophage polarization from a pro-inflammatory toward a reparative, pro-angiogenic state, establishing the immunometabolic mechanism for post-infarct repair.

We appreciate your concern that these conceptual links were not sufficiently highlighted in the original version. In the revised Discussion, we now: (i) explicitly summarize the key contributions of the cited studies, (ii) clearly delineate how our work builds on and extends these prior findings and (iii) describe our contribution cautiously and precisely.

Manuscript edits: “Discussion”

Recent work has convincingly established that BAT as a systemic cardiometabolic modulator (Nat Med. 2021.). BAT is highly metabolically active, and its mitochondria-derived materials can traffic between cells (Cell Metab. 2021; Cell Metab. 2022; Cell Transplant. 2023). Extending this framework, our data point to a more defined interorgan axis in post-infarct context. BAT releases a mitochondria-derived, OXPHOS-enriched MDV population that appears to be preferentially captured by the cardiac macrophages. Functionally, MDV bioenergetic integrity (especially complex V) is required to couple myocardial uptake to macrophage immunometabolic reprogramming and tissue repair, establishing BAT-derived MDVs as a mechanistically tractable mediator of interorgan mitochondrial signaling in cardiac injury.

(2) From the therapeutic view, does using MDVs offer a distinct and necessary advantage over using engineered EVs, or direct mitochondrial transfer, or BAT? Why did the authors choose MDVs over the others? A direct comparison of these strategies-including delivery routes (e.g., intravenous vs. tissue transplantation)-would significantly enhance the translational relevance the utility of MDVs.

Response：

Thank you for the insightful translational perspective! We agree that multiple bioenergetic delivery strategies, including engineered EVs, mitochondrial transfer approaches, and BAT-based interventions, represent important and potentially complementary translational directions. In the current study, our primary intent was to identify and mechanistically define an endogenous BAT-to-heart mediator after MI and to establish cargo-dependent causality.

While we were not able to complete head-to-head comparisons against engineered EVs or isolated mitochondrial administration within this revision, we have strengthened the translational relevance in two ways. First, we now provide the in vivo comparison between BAT transplantation and BAT-MDV administration in the same MI setting using matched functional, histological readouts. BAT-MDV administration produced improvements in cardiac function and remodelling that were comparable to those observed with BAT transplantation (Fig. R25). These data suggest that a cell-free, cargo-defined MDV preparation can achieve the tissue-level benefit. Such MDV product may offer practical translational attributes, standardizable dosing and product characterization, and avoidance of tissue implantation.

Second, Regarding delivery, we have clarified the MDV administration route, dosing normalization, and timing in the Methods. We selected intravenous delivery as a proof-of-principle route to test whether a cell-free, circulation-accessible mediator can reach the injured heart and elicit biological efficacy without tissue implantation. Additional route-level and modality-level comparisons will be important future work, and we now outline a concrete experimental framework for these comparisons in the Discussion.

Engineered EVs offer clear advantages in programmable targeting, controlled cargo loading, flexibility, and less immune activation. Our work specifies both the functional cargo requirement (OXPHOS, particularly complex V) and the primary recipient cell type (cardiac macrophages), thereby providing a mechanistic design specification that could offer rational for future EV engineering.

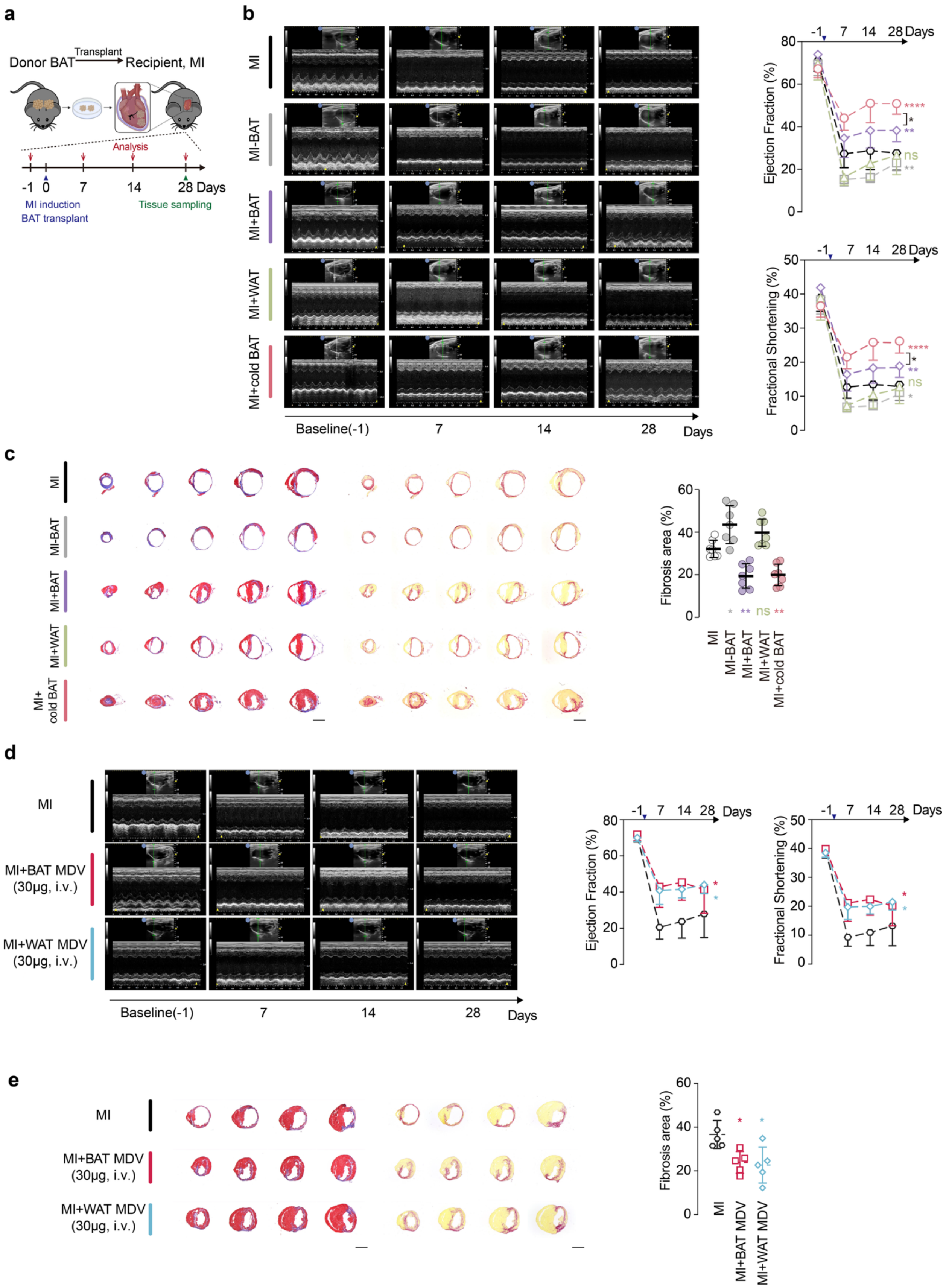
a
Donor BAT
Transplant
Recipient, MI
Analysis
-1 0 7 14 28 Days
MI induction
BAT transplant
Tissue sampling
b
MI
MI-BAT
MI+BAT
MI+WAT
MI+cold BAT
Baseline(-1) 7 14 28 Days
Ejection Fraction (%)
Fractional Shortening (%)
ns
c
MI+
cold BAT
Fibrosis area (%)
MI
MI-BAT
MI+BAT
MI+WAT
MI+cold BAT
d
MI
MI+BAT MDV
(30μg, i.v.)
MI+WAT MDV
(30μg, i.v.)
Baseline(-1) 7 14 28 Days
Ejection Fraction (%)
Fractional Shortening (%)
e
MI
MI+BAT MDV
(30μg, i.v.)
MI+WAT MDV
(30μg, i.v.)
Fibrosis area (%)
MI
MI+BAT MDV
MI+WAT MDV

Fig. R25 a, Experimental timeline for MI induction, adipose tissue pericardial transplantation, and outcome assessments. Echocardiography was performed at baseline (day −1) and on days 7, 14, and 28. Hearts were harvested on day 28 for Masson's trichrome and Sirius Red staining. **b,** Representative B-mode and M-mode echocardiography of BAT-receiving mice at baseline and days 7, 14, and 28. Ejection fraction and fractional shortening were quantified (mean±s.d., n=7, repeated two-way ANOVA and Holm-Sidak post hoc, *$p < 0.05$, **$p < 0.01$, ****$p < 0.0001$). **c,** Representative Masson and Sirius Red staining images of mouse hearts at day 28. Fibrosis percentage was quantified (mean±s.d., n=7, One-way ANOVA test followed by Bonferroni method, *$p < 0.05$, **$p < 0.01$, ***$p < 0.001$, ****$p < 0.0001$). Scale bar: 2mm. **d,** Representative B-mode and M-mode echocardiography of adipose tissue-derived MDV-treated mice at baseline and days 7, 14, and 28. Ejection fraction and fractional shortening were quantified (mean±s.d., n=5, repeated two-way ANOVA and Holm-Sidak post hoc, *$p < 0.05$). **e,** Representative Masson and Sirius Red staining images of MDV-treated mouse hearts at day 28. Fibrosis percentage was quantified (mean±s.d., n=5, One-way ANOVA test followed by Bonferroni method, *$p < 0.05$). Scale bar: 2mm.

(3) In mouse models of myocardial infarction (MI), researchers typically assess cardiac fibrosis and pathological hypertrophic remodeling at 2 to 6 weeks post-MI. At 1-3 days post MI, cardiomyocyte death, acute inflammation; at 1-week, inflammatory response, beginning of fibrotic deposition, at 2-4 weeks, active fibrotic remodeling and hypertrophy, functional decline, at 6-8 weeks, chronic remodeling, scar stabilization, ventricular dilation to heart failure (Circulation Research 2016;119(1):91-112, PMID: 27340270). In this study, the authors assessed the fibrosis and hypertrophic remodeling at very early timepoints (day 4-7) post MI, which are not accurate for claiming the BAT improvement in remodeling phenotype. The authors should provide late-stage assessment of fibrosis and cardiac hypertrophy. In addition, aSMA, Periostin ColI, Col III, and ANP, BNP, myh7, which are all markers for fibrotic and hypertrophic remodeling, should be evaluated.

Response：

We deeply appreciate your comment regarding the temporal dynamics of post-MI remodeling.

We fully agree that the early (day 4-7) detection window incompletely captures the biology of scar maturation and global functional remodeling after permanent LAD ligation. We re-conducted all the mouse phenotyping with a primary endpoint at day 28 after MI induction and improved the study design.

i) In the revised manuscript, left ventricular function was assessed by serial echocardiography at baseline (a day before MI induction), day 7, day 14, and day 28 after MI induction. On day 28 after MI induction, hearts were harvested. Ventricular remodeling and fibrosis were assessed by Masson’s trichrome staining and Picrosirius Red staining.
ii) We also increased group sizes across groups to enhance statistical power.
iii) We have standardized the surgical procedure, adopting a moderate LAD ligation technique (consistent placement ~1-2 mm below the LAA with controlled tension) instead of the proximal LAD ligation technique used in the original manuscript, to reduce mortality and minimize survival bias.

The results of these revised data for four major phenotype experiments are listed below (Fig. R26).

1. Pericardial BAT transplantation attenuated adverse post-MI remodeling, as BAT-grafted mice showed higher EF/FS and smaller fibrotic areas than MI controls, whereas BAT-deficient mice had worse function and more fibrosis. WAT transplantation provided no benefit, while cold-activated BAT further improved systolic function, indicating a BAT-related, thermogenesis-enhanced cardioprotective effect.
2. Intravenous delivery of adipose-derived MDVs (30 μg protein per mouse) after MI improved LV systolic function, limited adverse remodeling, and reduced fibrosis compared with vehicle controls. BAT- and WAT-derived MDVs exerted comparable benefits, indicating that MDVs from either adipose depot possess intrinsic cardioprotective activity.
3. Transplantation of wild-type BAT improved EF/FS over 4 weeks after MI and reduced infarct fibrosis, whereas BAT from *Becn1*$^{+/-}$ donors, despite similar engraftment, failed to confer functional or structural benefit, indicating that BECN1 in donor BAT is required for BAT-mediated cardioprotection.
4. Intravenous delivery of wild-type BAT-MDVs improved LV function and reduced fibrosis, whereas MDVs from *Becn1*$^{+/-}$ BAT showed markedly attenuated effects, demonstrating that BECN1 is dispensable for bulk MDV yield but essential for the cardioprotective efficacy of BAT-derived MDVs in vivo.

The inconsistency between the effects of WAT-transplantation and WAT-MDV treatment can be explained by the much lower yield of MDV from WAT, and the secretion of cardio deleterious lipids and adipokines from WAT, thereby unmasking the intrinsic cardioprotective capacity of adipose-derived MDVs.

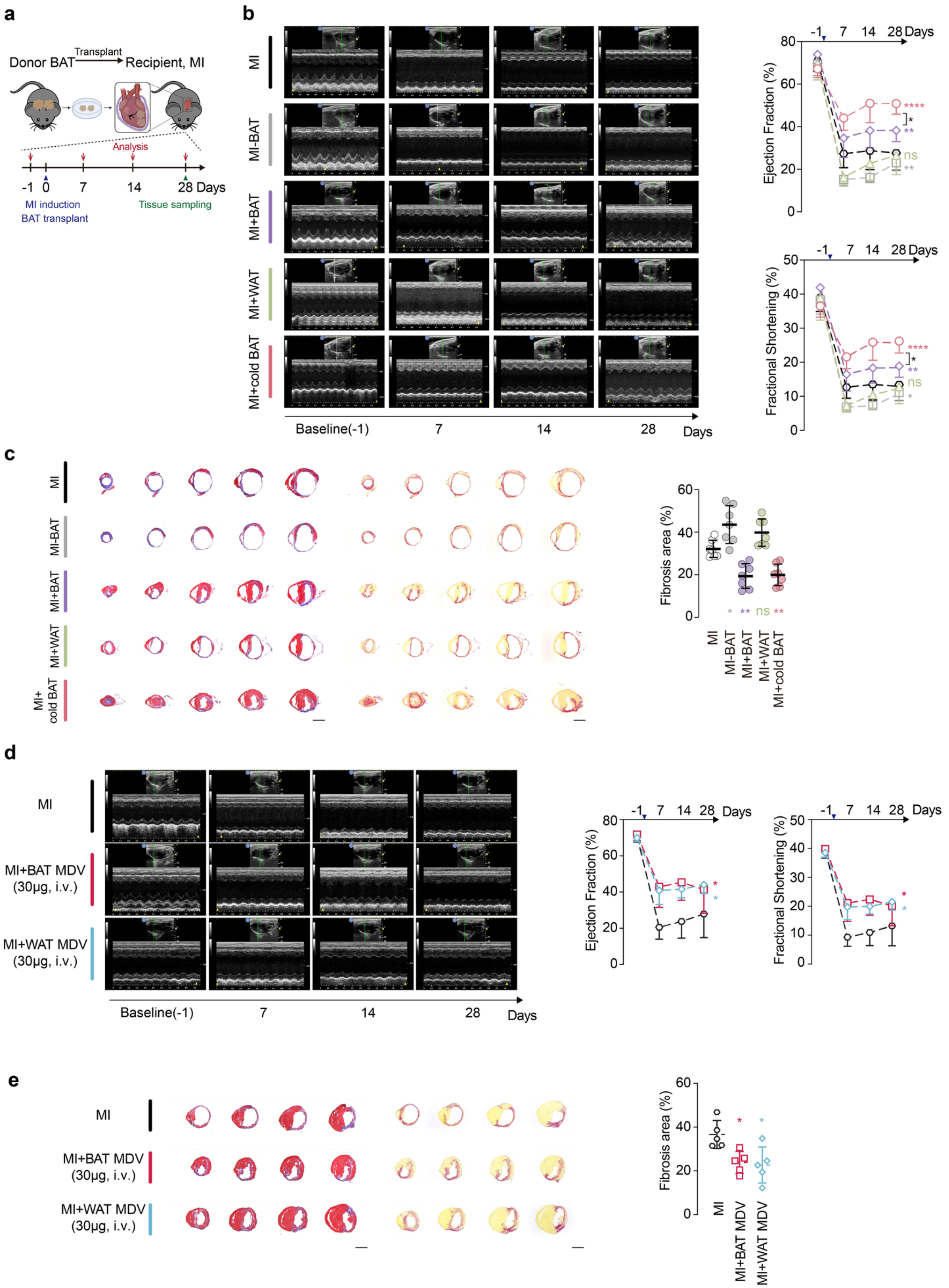
a
Donor BAT
Transplant
Recipient, MI
Analysis
-1 0 7 14 28 Days
MI induction
BAT transplant
Tissue sampling
b
MI
MI-BAT
MI+BAT
MI+WAT
MI+cold BAT
Baseline(-1)
7
14
28
Days
Ejection Fraction (%)
Fractional Shortening (%)
****
*
**
ns
c
MI
MI-BAT
MI+BAT
MI+WAT
MI+ cold BAT
Fibrosis area (%)
MI+cold BAT
d
MI
MI+BAT MDV (30μg, i.v.)
MI+WAT MDV (30μg, i.v.)
Baseline(-1)
7
14
28
Days
Ejection Fraction (%)
Fractional Shortening (%)
e
MI
MI+BAT MDV (30μg, i.v.)
MI+WAT MDV (30μg, i.v.)
Fibrosis area (%)
MI+BAT MDV
MI+WAT MDV

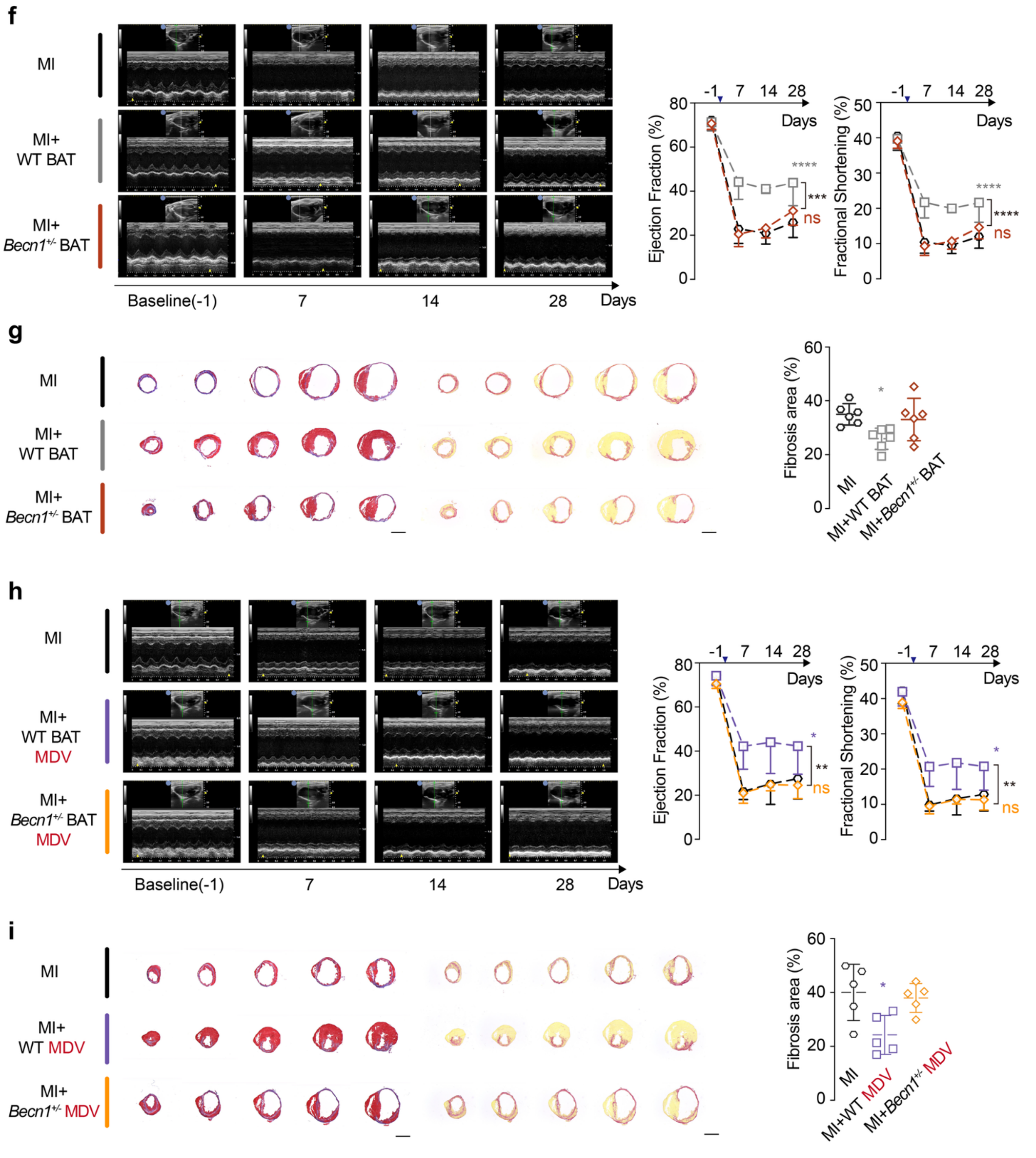

Fig. R26 a, Experimental timeline for MI induction, adipose tissue pericardial transplantation, and outcome assessments. Echocardiography was performed at baseline (day −1) and on days 7, 14, and 28. Hearts were harvested on day 28 for Masson's trichrome and Sirius Red staining. **b,** Representative B-mode and M-mode echocardiography of BAT-receiving mice at baseline and days 7, 14, and 28. Ejection

fraction and fractional shortening were quantified (mean±s.d., n=7, repeated two-way ANOVA and Holm-Sidak post hoc, *p < 0.05, **p < 0.01, ****p < 0.0001). **c,** Representative Masson and Sirius Red staining images of mouse hearts at day 28. Fibrosis percentage was quantified (mean±s.d., n=7, One-way ANOVA test followed by Bonferroni method, *p < 0.05, **p < 0.01, ***p < 0.001, ****p < 0.0001). Scale bar: 2mm. **d,** Representative B-mode and M-mode echocardiography of adipose tissue-derived MDV-treated mice at baseline and days 7, 14, and 28. Ejection fraction and fractional shortening were quantified (mean±s.d., n=5, repeated two-way ANOVA and Holm-Sidak post hoc, *p < 0.05). **e,** Representative Masson and Sirius Red staining images of MDV-treated mouse hearts at day 28. Fibrosis percentage was quantified (mean±s.d., n=5, One-way ANOVA test followed by Bonferroni method, *p < 0.05). Scale bar: 2mm. **f,** Representative B-mode and M-mode echocardiography of WT or *Becn1*$^{+/-}$ BAT-receiving mice at baseline and days 7, 14, and 28. Ejection fraction and fractional shortening were quantified (mean±s.d., n=6, repeated two-way ANOVA and Holm-Sidak post hoc, ***p < 0.001, ****p < 0.0001). **g,** Representative Masson and Sirius Red staining images of WT or *Becn1*$^{+/-}$ BAT-receiving mice hearts at day 28. Fibrosis percentage was quantified (mean±s.d., n=6, One-way ANOVA test followed by Bonferroni method, *p < 0.05). Scale bar: 2mm. **h,** Representative B-mode and M-mode echocardiography of WT or *Becn1*$^{+/-}$ MDV-treated mice at baseline and days 7, 14, and 28. Ejection fraction and fractional shortening were quantified (mean±s.d., n=5, repeated two-way ANOVA and Holm-Sidak post hoc, *p < 0.05, **p < 0.01). **i,** Representative Masson and Sirius Red staining images of WT or *Becn1*$^{+/-}$ MDV-treated mice hearts at day 28. Fibrosis percentage was quantified (mean±s.d., n=5, One-way ANOVA test followed by Bonferroni method, *p < 0.05). Scale bar: 2mm. **The above new data are included in Fig. 1, 3, and 5 in the revised manuscript.**

(4) Previous report indicates that stressed adipocytes release small extracellular vesicles (sEV), which contain respiration-competent and enter circulation. sEV are taken up by cardiomyocytes and subsequently protect cardiomyocytes and ischemic heart injury (Cell Metab. 2021;33(9):1853-1868). In this study, the authors found the MDVs are mainly delivered to macrophage and protect the cardiac remodeling, in which are not taken up by cardiomyocytes. Although the authors used the BAT mtGFP mice to label BAT mitochondria, did the authors check the border zone or remote zone? The fig2g, Extent fig3d, are not clear enough to exclude the MDVs' transferring to other cell types, (such as neutrophils, fibroblasts), which may contribute to the cardiac protection as well. The authors may conduct FACs analysis of mtGFP+ populations across cardiac cell types to elucidate the cell types taken up by the MDVs.

Response:

We are very grateful for your thoughtful comment. To address the cell types by which BAT-MDVs are uptaken, we have performed additional experiments to (i) asses BATosome signals across infarct, border, and remote zones; (ii) improved lineage co-staining for potential recipient cell types; (iii) flow cytometric profiling of vesicle-receiving immune populations.

According to the results, we now describe BAT-derived MDVs as being preferentially detectable in cardiac macrophages, with limited signals in other myeloid cells (e.g. neutrophils) and lymph cells (Fig. R27). Only a rare cardiomyocyte-associated signal was detected under our conditions and within the examined time window.

A likely reason for the inconsistency with the Crewe et. al. (Cell Metab. 2021 PMID: 34418352) is that the vesicle populations and the biological context under investigation are not directly comparable. The previous work analyzed an adipocyte-derived small EV pool released into the extracellular space, whereas in our study we isolate BAT-derived MDVs with a defined sub-mitochondrial cargo profile. These MDVs may differ from bulk sEVs in biogenesis abd membrane identity, which can influence vesicle-cell interactions and receptor binding. It is therefore plausible that adipocyte sEVs and BAT-derived MDVs exhibit distinct recipient-cell profile.

In addition, our experiments focus on the sub-acute MI setting, where the infarct and border zones are populated by inflammatory myeloid cells with high phagocytic activity. In such a milieu, circulating or locally delivered vesicles may be preferentially captured by macrophages before they can accumulate and be taken into cardiomyocytes.

The results of additional experiments are listed below:

(i) Assessment across infarct, border, and remote zones.

We analyzed reporter signal ($BAT^{ZsGreen}$) in infarct, border, and remote regions of the post-MI heart. These data show that $ZsGreen^{+}$ signal is detectable in the infarct and border zones and is largely absent in remote myocardium.

(ii) Evaluation of additional candidate recipient cell types.

We examined reporter signal ($BAT^{ZsGreen}$ or BAT^{mtGFP}) in candidate recipient cells (neutrophil, MPO; myofibroblast, α-SMA). In this analysis, ZsGreen or mtGFP signals in MPO^{+} and $αSMA^{+}$ cells are detectable but less prominent than in $CD68^{+}$ macrophages, indicating that while additional myeloid or stromal subsets may receive MDVs, they are not dominant recipient population.

(iii) Flow-cytometric profiling of vesicle-receiving immune populations.

We performed flow cytometry on infarcted hearts from $BAT^{ZsGreen}$ recipients to quantify reporter-positive cells across immune lineages. Among $CD45^{+}$ $ZsGreen^{+}$ cells, $CD11b^{+}F4/80^{+}$ macrophages account for 66.98%, $CD11b^{+}F4/80^{-}$ neutrophil/monocyte populations for 29.58%, and lymphocytes for 3.44% (n = 12 hearts) (Fig. R27). Within the immune cells, macrophages represent the predominant vesicle-receiving population, while other myeloid cells can acquire BATosomes as well.

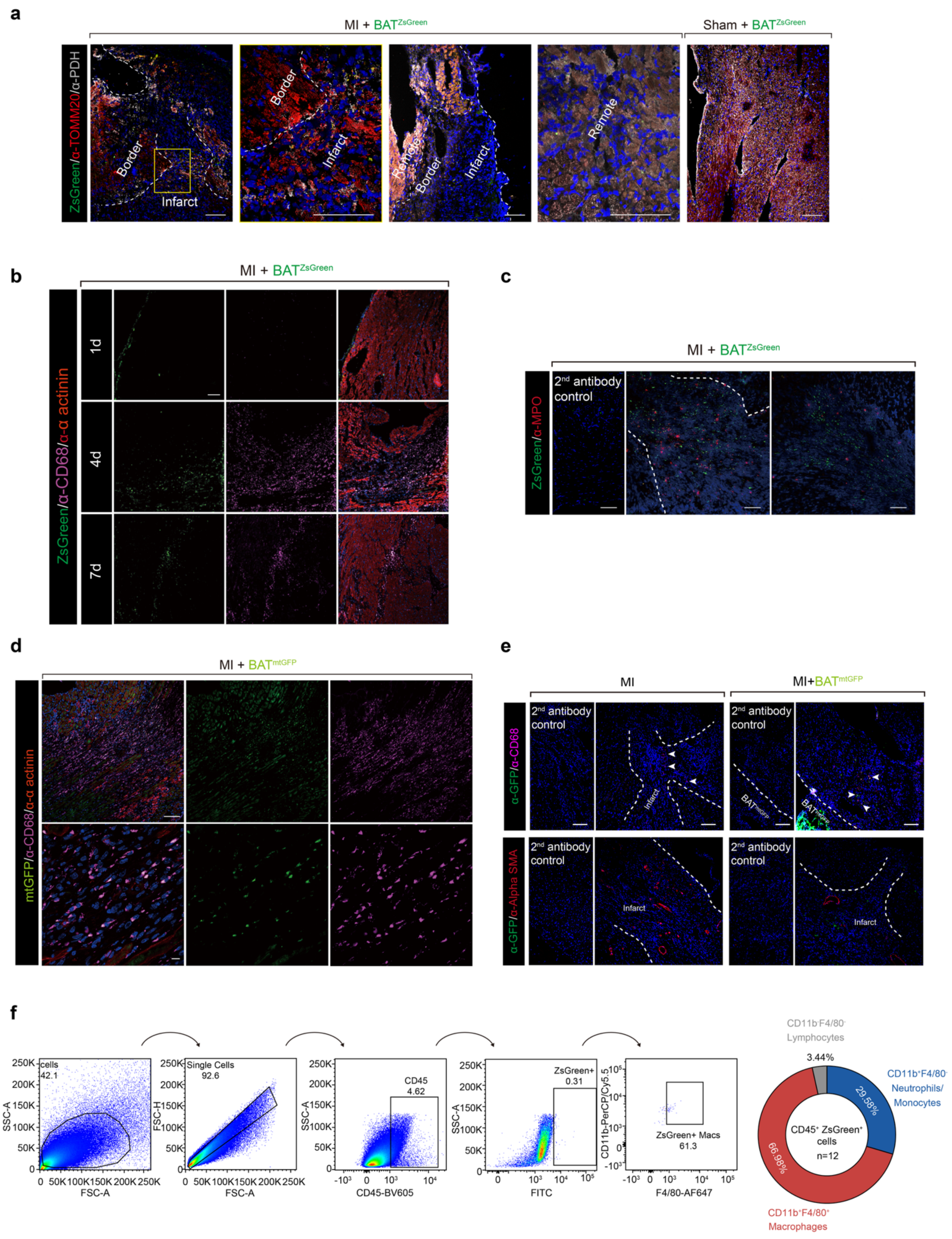
a
MI + BAT^ZsGreen
Sham + BAT^ZsGreen
ZsGreen/α-TOMM20/α-PDH
Border
Infarct
Remote
b
MI + BAT^ZsGreen
ZsGreen/α-CD68/α-α actinin
1d
4d
7d
c
MI + BAT^ZsGreen
ZsGreen/α-MPO
2nd antibody control
d
MI + BAT^mtGFP
mtGFP/α-CD68/α-α actinin
e
MI
MI+BAT^mtGFP
α-GFP/α-CD68
α-GFP/α-Alpha SMA
2nd antibody control
Infarct
f
cells 42.1
Single Cells 92.6
CD45 4.62
ZsGreen+ 0.31
ZsGreen+ Macs 61.3
SSC-A
FSC-A
FSC-H
CD45-BV605
FITC
CD11b-PerCP/Cy5.5
F4/80-AF647
CD11b-F4/80- Lymphocytes
3.44%
CD11b+F4/80- Neutrophils/ Monocytes
29.58%
CD45+ ZsGreen+ cells n=12
66.98%
CD11b+F4/80+ Macrophages

Fig. R27 a, Representative confocal images of left ventricles 4 days after MI in mice with or without $BAT^{ZsGreen}$ transplantation, stained for sarcomeric α-actinin (red) and nuclei (DAPI, blue). 3D reconstruction of the infarct zone of $BAT^{ZsGreen}$-receiving mice at MI 4d. Scale bar: 100μm. **b,** Time course of BAT-derived MDVs accumulation and macrophage infiltration in $BAT^{ZsGreen}$-receiving mice at day 1, 4, and 7 after MI. Sections were stained for sarcomeric α-actinin (cardiomyocytes, red), CD68 (macrophages, magenta), and nuclei (DAPI, blue). Scale bar: 100μm. **c,** Representative confocal images of the left ventricles in $BAT^{ZsGreen}$-receiving mice at MI 4d, stained for MPO (neutrophil, red) and nuclei (DAPI, blue). Scale bar: 100μm. **d**, Representative confocal images of infarct myocardium 4 days after MI in BAT^{mtGFP}-receiving mice. Sections were stained for sarcomeric α-actinin (red), CD68 (magenta), and nuclei (DAPI, blue). Upper scale bar: 100μm; lower scale bar: 10μm. **e,** Immunofluorescence validation of mtGFP signal in infarcted hearts of BAT^{mtGFP}-receiving mice. Sections were stained for GFP (green) with CD68 (macrophages) or α-SMA (fibroblasts) (red). Scale bar: 100μm. **f,** Flow-cytometric gating strategy for identifying BATosome-recipient immune cells in infarcted hearts from $BAT^{ZsGreen}$-receiving mice at MI 4d. After debris exclusion and singlets selection, leukocytes were gated as $CD45^+$ cells, followed by $ZsGreen^+$ events and $CD11b^+F4/80^+$ macrophages within the $CD45^+ZsGreen^+$ population. Quantification of immune cell subsets among $ZsGreen^+CD45^+$ cells in infarcted hearts from $BAT^{ZsGreen}$-receiving mice at MI 4d. Data are presented as mean percentages of each subset among total $ZsGreen^+CD45^+$ cells (n as indicated). **The above data are included in Fig. 2, Extended Data Fig. 2 and 3 in the revised manuscript.**

(5) Myocardial infarction reduces BAT mass (Fig1a) and stresses the BAT. The authors transplant donor unstressed BAT to recipient mice with MI to find out the roles of MDVs. Did the authors also do MI surgery in BATmtGFP mice and check if stressed BAT delivery its own MDVs to the heart in the same mice? It seems that BAT undergoing stress has better protective roles, like cold-BAT reduces more fibrosis than BAT transplantation in Fig1f. Whether MI stressed BAT may function as well when transplanting to MI mice?

Response:

We appreciate your insightful question regarding the role of stressed BAT and the possibility that MI drives BAT-to-heart MDV transfer within the same animal. To address this point, we established a BAT-specific mitochondrial labelling model using AAV8-mtGFP injected in situ into the interscapular BAT. Four weeks after AAV administration, when mtGFP expression in BAT mitochondria was stable, we subjected these mice to MI or sham surgery and harvested hearts at day 4 post-MI. In this setting without BAT transplantation, we observed mtGFP^{+} puncta in the infarct zones of MI hearts, whereas absent in sham animals. Co-staining with CD68 indicated that mtGFP^{+} structures co-localize CD68^{+} macrophages, with a rare co-localization with cardiomyocytes (Figure R28).

a

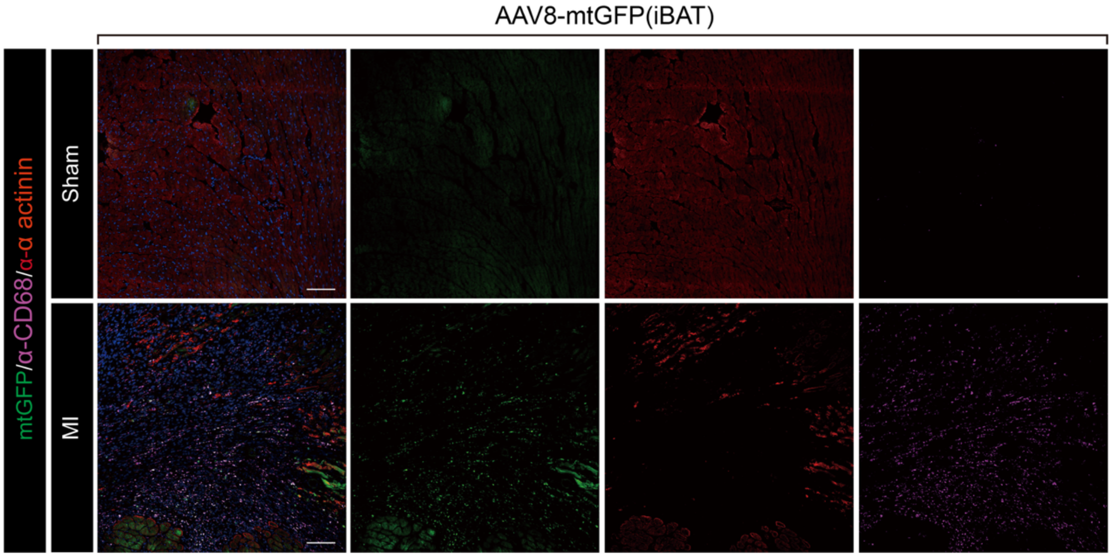

Fig. R28 a, Tracing endogenous BAT-derived MDVs using AAV8-mtGFP injected in situ into interscapular BAT. Hearts from sham and MI mice were analyzed 4 days after surgery and stained for CD68 (magenta), sarcomeric α-actinin (red), and DAPI (blue). Scale bar: 100μm. **The above new data are included in Extended Data Fig. 3 in the revised manuscript.**

Regarding the question whether MI-stressed BAT can function better than normal BAT, we sincerely appreciate this biologically insightful question and completely agree that the MI-stressed BAT is an important issue to consider.

The physiologically activated BAT (cold-exposed donor BAT) transplantation shows greater protection than transplantation of room-temperature BAT (Fig. 1b-f), supporting the hypothesis that enhanced mitochondrial function are extraordinarily beneficial.

Our transcriptional and histological analysis indicate that MI induces the acute atrophy (Fig. 1a), lipid mpbilization and metabolism (Fig. 4a), and global inflammation in BAT (RNA-seq data not shown in this manuscript). These data suggest that MI-stressed BAT is not simply a more activated version of BAT, but rather a qualitatively different state from cold-activated BAT.

Donor MI is associated with global inflammation, neurohumoral activation and metabolic stress, all of which could alter BAT function in ways that are difficult to isolate from the effects of MDV transfer itself. Also, mitochondrial and MDV cargo quality may be impaired compared with naïve or activated BAT.

Based on these considerations, the results of MI-BAT transplantation would be difficult to interpret independently of donor-related systemic inflammatory and metabolic confounding factors.

(6) Brown adipocytes tend to eject their mitochondria under stress for the interorgan mechanisms. But how MI injured heart initiates the signaling and impacts BAT metabolism and mitochondria as well as the biogenesis mechanism of MDVs are unknown in the manuscript.

Response：

Thank you for highlighting this important mechanistic aspect. We acknowledge that the upstream signaling, how the signals from the injured heart are received by brown adipose tissue and activate mitochondrial quality-control mechanisms within brown adipocytes, remains incompletely understood.

We have collected some data informative of the upstream signaling. At the organ level, MI induced BAT atrophy, with lipolysis and lipid mobilization, together with a shift toward stress-responsive transcriptional program, whereas *Ucp1* expression remains unchanged. These observations suggest that MI drives BAT into a metabolically stressed state that is distinct from classical thermogenic activation. At the cellular level, BECN1 inhibition reduced VPS35 levels especially in mitochondria fraction, and impaired the release of $TOMM20^{+}PDH^{-}$ MDVs.

On the basis of our data, we propose a working model in which VPS35 functions as an intracellular integrator of extracellular cues, linking heart-derived stress signals to MDV biogenesis in brown adipocytes. We hypothesize that VPS35, as a core component of the retromer complex, senses upstream stress signals and engages retromer-mediated retrograde trafficking, leading to its recruitment to mitochondrial membranes where it organizes cargo-selective budding domains and thereby promotes the formation of OXPHOS-enriched mitochondria-derived vesicles.

Molecularly, we doubt whether BECN1-dependent VPS35 mitochondria recruitment is mediated by the direct binding of BECN1-VPS35. Thus, we performed a GFP-Trap pulldown assay in HeLa cells co-expressing VPS35-GFP and BECN1-mCherry (Fig. R29). VPS35-GFP was efficiently precipitated by GFP-Trap, but BECN1-mCherry was not detected in the GFP-Trap fraction, indicating that under these conditions BECN1 and VPS35 do not form a stable complex.

a

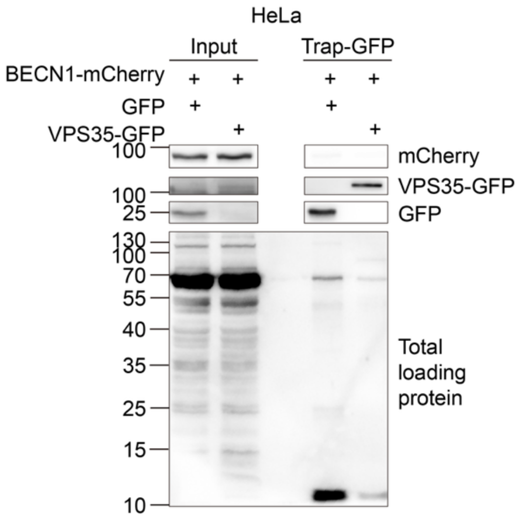

Fig. R29 a, HeLa cells transfected with BECN1-mCherry together with GFP or VPS35-GFP as indicated. Input lysates and GFP-Trap fractions (Trap-GFP) were analyzed by immunoblot for mCherry and GFP.

The precise receptors and signalling pathways that position VPS35 at the mitochondria, and how retromer-mediated trafficking is coordinated with other components of the mitochondrial quality-control machinery, remain to be elucidated in future work.

(7) There is missing cardiac function(echocardiography) data in Fig3d, which is important for claiming the MDVs protection, although the authors present fibrosis and remodeling data.

Response：

Thank you for pointing out!

In the revised manuscript, we have now performed echocardiography on BAT- and WAT-MDV-treated mice at baseline and day 7, 14, and 28 post-MI (Fig. R30).

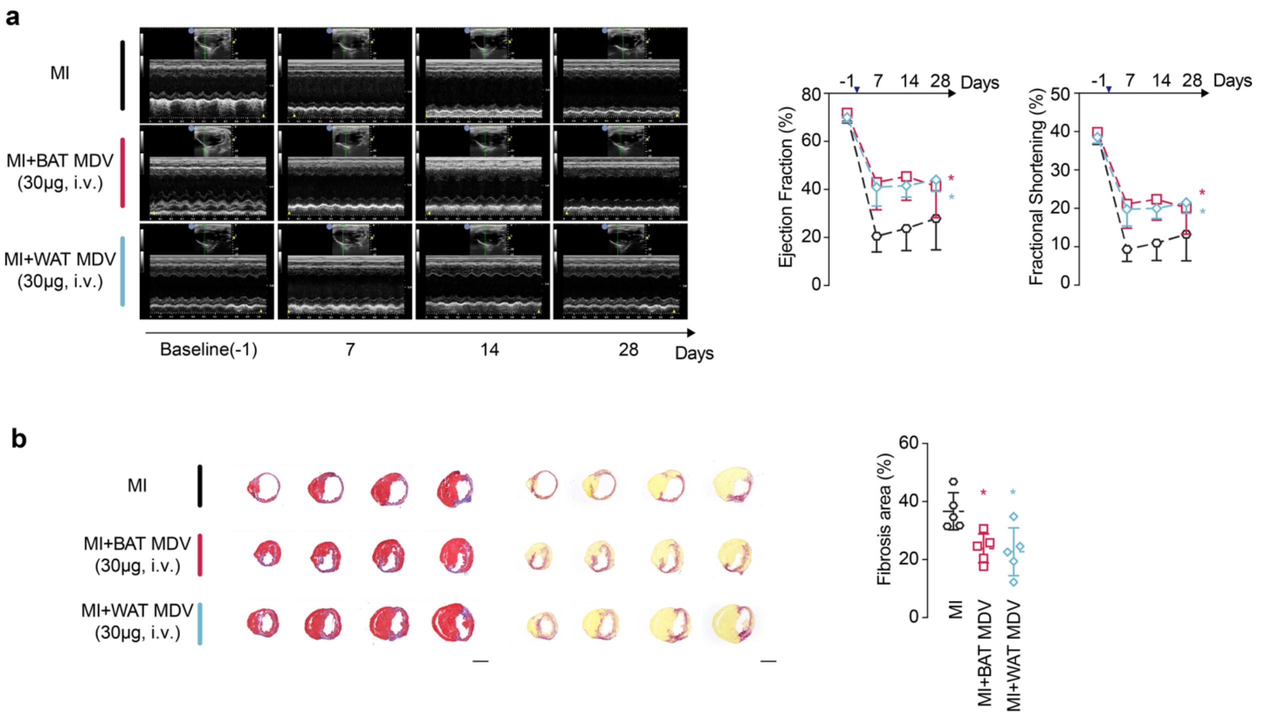

Fig. R30 a, Representative B-mode and M-mode echocardiography of adipose tissue-derived MDV-treated mice at baseline and days 7, 14, and 28. Ejection fraction and fractional shortening were quantified (mean±s.d., n=5, repeated two-way ANOVA and Holm-Sidak post hoc, *p < 0.05). **b,** Representative Masson and Sirius Red staining images of MDV-treated mouse hearts at day 28. Fibrosis percentage was quantified (mean±s.d., n=5, One-way ANOVA test followed by Bonferroni method, *p < 0.05). Scale bar: 2mm. **The above new data are included in Fig. 3 in the revised manuscript.**

(8) How long the MDVs stay or last in the heart or macrophage? In Fig2h, round-shaped GFP+ vesicles appeared in the macrophage cytoplasm, and some were fused with recipient macrophage mitochondria, what's the percentage of fused or cytoplasmic MDVs? Whether they have the same function? How fused with recipient macrophage mitochondria function?

Response:

We are grateful for these thoughtful questions regarding the fate and persistence of BAT-derived MDVs in the heart and in recipient macrophages.

These are mechanistically insightful questions and touches the fundamental aspects of MDV biology, the intracellular fate and functional specialization of vesicles that engage vs. remain separate from the host mitochondrial network.

Based on our data and the international consensus statement on mitochondria transfer and transplantation (Nat Metab. 2025. PMID: 39820558), we could summarize as follows.

(i) The MDV retention time in heart and macrophages.

Using $BAT^{ZsGreen}$ and BAT^{mtGFP} donors, we detect BAT-derived reporter-positive puncta in $CD68^{+}$ cardiac macrophages as early as day 1 after MI and BAT transplantation, with robust signal at day 4 and clear persistence at least to day 7 in the infarct zone. The exact retention time has not been defined, as the analysis was not extended beyond 7 days. In the context of mitochondria transplant, the extracellular mitochondria could be detected after 28 days (J Thorac Cardiovasc Surg. 2017. PMID: 27938904).

(ii) Subcellular localization and fate of uptaken MDVs.

Transferred mitochondria (or MDVs) can follow multiple, non-exclusive fates after cell entry, including rapid degradation, incorporation into the endogenous mitochondrial network, transient metabolic support without long-term persistence, or repackaging and re-export to other cells (Nat Metab. 2025. PMID: 39820558). The consensus further cautions that these fates are likely to vary with donor cell type, acceptor cell type and

disease context, and that precise definition of fate is not required to establish that transfer occurs, although it is important for mechanistic interpretation.

(iii) The function of host mitochondria-fused MDVs

MDVs that enter acceptor cells can follow multiple fates, including rapid degradtation, stable engagement with the host mitochondrial network, or transient signalling roles. In our setting, BAT-derived MDV structures in heart appear in at least two morphological states: discrete GFP^+ puncta in the cytoplasm and GFP^+ structures closely apposed to host mitochondria. Our functional assays capture the net effect of MDV uptake and do not allow us to attribute specific functions to these subpopulations. It is therefore not yet possible to determine whether mitochondria-associated MDVs and cytoplasmic MDVs make equivalent or distinct contributions to macrophage rewiring.

(9) BECN1 increased in heart DAMPs-treated brown adipocytes (Fig. 4g), The use of systemic Becn1-deficient mice raises concerns about cell-type specificity. Effects observed may not reflect BAT-specific mechanisms. A BAT-specific Becn1 knockout model would more convincingly support the proposed mechanism. In addition, Extended Data 7ab, the heart function (EF% and FS%) actually exhibited no changes, indicating Becn1 may not attribute for the protection in this time point. The authors may assess the cardiac function at late stage.

Response：

We appreciate your thoughtful comments regarding the interpretation of our BECN1 data. We agree that both cell-type specificity and appropriate functional endpoints are critical for supporting the proposed mechanism.

Late-stage cardiac function in Becn1 experiments.

In the original version of Extended Data Fig. 7a,b, EF% and FS% at the early time point did not differ between groups, which limits the strength of any conclusion regarding BECN1-dependent cardioprotection at that stage. In response, we have extended echocardiographic follow-up in the Becn1 transplantation paradigm to day 28 post-MI. As shown in the new data (Fig. R31), mice receiving WT BAT display better preserved EF and FS from day 7 through day 28 compared with MI alone, whereas mice receiving *Becn1* $^{+/-}$ BAT exhibit a blunted and non-significant improvement in systolic function at day 28. Masson's staining demonstrates reduced fibrosis area in the MI+WT BAT group compared with MI+*Becn1* $^{+/-}$ BAT and MI alone.

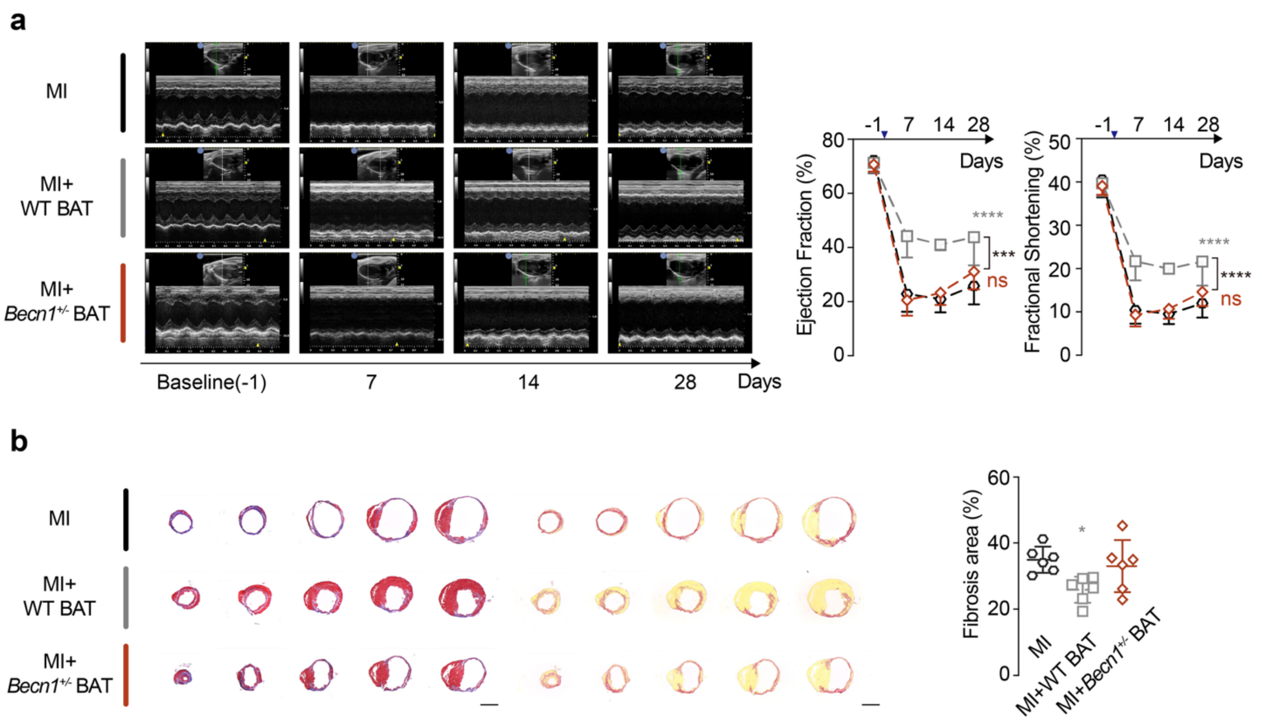

Fig. R31 a, Representative B-mode and M-mode echocardiography of WT or *Becn1*$^{+/-}$ BAT-receiving mice at baseline and days 7, 14, and 28. Ejection fraction and fractional shortening were quantified (mean±s.d., n=6, repeated two-way ANOVA and Holm-Sidak post hoc, ***$p < 0.001$, ****$p < 0.0001$). **b,** Representative Masson and Sirius Red staining images of WT or *Becn1*$^{+/-}$ BAT-receiving mice hearts at day 28. Fibrosis percentage was quantified (mean±s.d., n=6, One-way ANOVA test followed by Bonferroni method, *$p < 0.05$). Scale bar: 2mm. **The above new data are included in Fig. 3 in the revised manuscript.**

These late-stage data indicate that BAT transplantation-mediated preservation of cardiac function is attenuated when *Becn1* is reduced in the donor BAT, consistent with the notion that BECN1-dependent MDV quality contributes to the long-term cardioprotective effect.

Cell-type specificity of Becn1 manipulation.

In our in vivo experiments, *Becn1*$^{+/-}$ heterozygote is confined to the donor BAT compartment. Specifically, interscapular BAT from *Becn1*$^{+/-}$ or wild-type mice is transplanted into wild-type MI recipients. Thus, differences between the MI+WT BAT and MI+*Becn1*$^{+/-}$ BAT groups reflect the Becn1 status of the transplanted BAT.

In parallel, our in vitro data provide cell-intrinsic support for a BAT-localized role of BECN1. *Becn1* inhibition in brown adipocytes reduces the production of mitochondrial-membrane MDVs and attenuates their ability to augment macrophage respiration and induce a reparative transcriptional programme (Fig. 4 and Fig. 7). Taken together, these findings support a role for BECN1 within brown adipocytes in modulating MDV biogenesis and cargo composition.

We concur that an inducible, BAT-specific Becn1 deletion would provide a more stringent genetic test of this mechanism, and we now explicitly highlight such models in the Discussion as an important direction for future work.

However, BAT also contains a minor stromal vascular fraction, including adipocyte progenitors, mesenchymal stromal cells and endothelial cells, and is infiltrated by diverse immune populations. Although resident immune cells within the transplanted BAT graft did not migrate to the myocardium (Extended Data Fig. 4), we cannot fully exclude indirect contributions from BAT-resident immune cells or other stromal-vascular components. An inducible, UCP1^{+} cell-restricted Becn1 deletion model would offer a more rigorous test of this mechanism. However, it is not feasible to generate such brown adipocyte-specific Becn1 knockout mice within the current revision of this manuscript. we now explicitly acknowledge the importance of Becn1 conditional knockout in the Discussion.

Manuscript edits: “Discussion”

Also, given that BAT contains multiple non-adipocyte populations, a Ucp1-Cre-driven, brown adipocyte-specific Becn1 knockout would provide a more definitive test of our

model by restricting BECN1 loss to thermogenic adipocytes and minimizing confounding effects from stromal-vascular and immune cells.

Minor concerns:

(1) Functional measurements are absent in Fig. 5e and Extended Fig. 7i. These should be included to support the therapeutic claims of MDV administration.

Response:

Thank you for bringing up this point. We have re-conducted the experiments with intact phenotype data (Fig. R32).

a

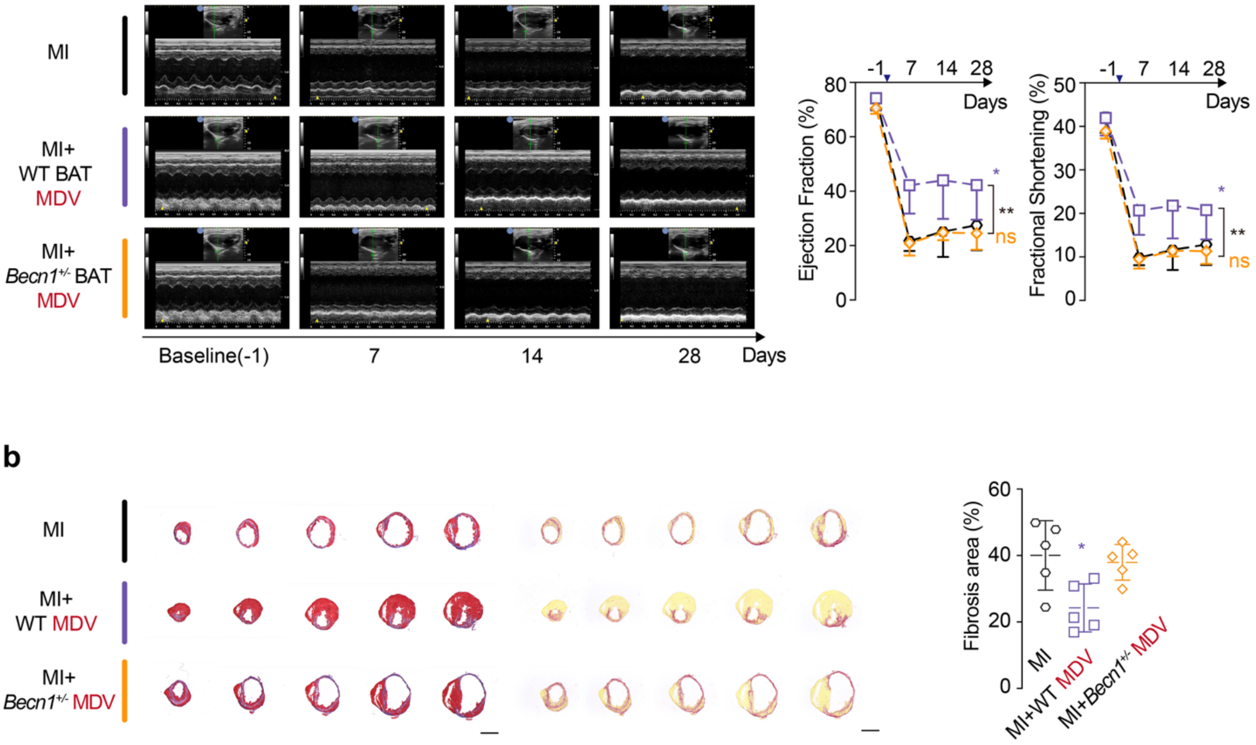

Fig. R32 a, Representative B-mode and M-mode echocardiography of WT or *Becn1*$^{+/-}$ MDV-treated mice at baseline and days 7, 14, and 28. Ejection fraction and fractional shortening were quantified (mean±s.d., n=5, repeated two-way ANOVA and Holm-Sidak post hoc, *p < 0.05, **p < 0.01). **b,** Representative Masson and Sirius Red staining images of WT or *Becn1*$^{+/-}$ MDV-treated mice hearts at day 28. Fibrosis percentage was quantified

(mean±s.d., n=5, One-way ANOVA test followed by Bonferroni method, $^*p < 0.05$). Scale bar: 2mm. **The above new data are included in Fig. 5 in the revised manuscript.**

(2) In Fig. 6c, only IL-4 shows changes. It would be informative to assess other M2-associated markers, such as IL-10 and Arg1, to confirm polarization phenotype.

Response：

We agree that macrophage polarization cannot rely on transcriptional signatures alone and should be supported by other readouts.

To address this, we first focused on inducible nitric oxide synthase (iNOS) and arginase-1 (ARG1), which compete for the common substrate L-arginine and act as core metabolic “switch” that determines the inflammation status of macrophages. $iNOS^{high}ARG1^{low}$ defines classically activated macrophage, whereas $iNOS^{low}ARG1^{high}$ macrophages are associated with tissue-repair and anti-inflammatory programmes. Thus, protein-level measurement of iNOS and ARG1 is widely used as a core criterion for defining macrophage polarization.

In the revised manuscript we therefore performed Western blots and cytokine assays in DAMP-activated macrophages treated with MDVs (Fig. R33 a-c). WT-MDVs decreased iNOS and increased ARG1 protein compared with PBS and Becn1-KD MDVs, and this shift was accompanied by higher IL-4 secretion and increased Il4 mRNA expression. In contrast, KD-MDVs largely failed to induce these changes. Complementary experiments with Complex V inhibition (oligomycin-treated MDVs) and ATP5A1 overexpression (Fig. R33 d-g) show that disrupting MDV complex V activity prevents the enzyme shift and blunts IL-4 induction, further linking MDV Complex V activity to the protein-level polarization state.

These results provide the protein- and cytokine-level validation and, together with the RNA-seq data in Fig. 7, support a conclusion that MDV delivery of bioenergetic cargo is

consistent with a shift toward a pro-reparative, anti-inflammatory macrophage phenotype.

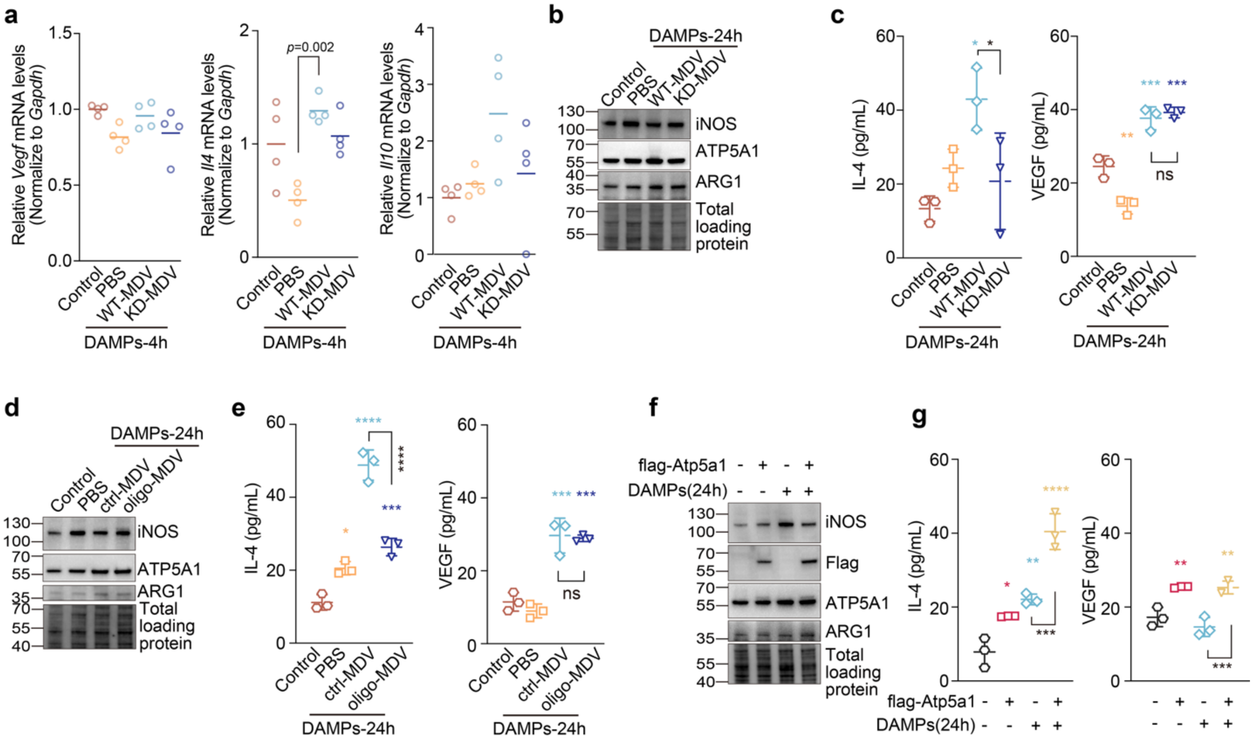

Fig. R33 a, RT-qPCR analysis of pro-reparative cytokine genes (*Vegf*, *Il4*, *Il10*) in iBMDM co-incubated with WT or *Becn1*$^{+/-}$ BAT MDV (mean, n=4 independent experiments, one-way ANOVA test followed by Bonferroni method). **b,** Representative western blots of macrophages treated with DAMPs for 24 h in the presence of PBS, WT-MDVs, or KD-MDVs (n=3 independent experiments). **c,** IL-4 and VEGF concentrations in supernatants from macrophages treated as in (**b**), measured by ELISA. (n=3 independent experiments). **d,** Representative western blots of macrophages treated with DAMPs for 24 h in the presence of PBS-treated MDVs, or oligomycin(100μg/mL)-treated MDVs (n=3 independent experiments). **e,** IL-4 and VEGF concentrations in supernatants from macrophages treated as in (**d**), measured by ELISA. (n=3 independent experiments). **f**, Representative western blots of macrophages infected with flag-Atp5a1 lentivirus and treated with DAMPs for 24 h (n=4 independent experiments). **g,** IL-4 and VEGF concentrations in supernatants from macrophages treated as in (**f**), measured by ELISA. (n=3 independent experiments). **The above new data are included in Fig. 7 in the revised manuscript.**

(3) Fig. 2g lacks sham controls for comparison

Response:

Thank you for the comment. Images of control groups were acquired with the experiment group. However, the sham control images were of low resolution and were not presented in the original submission.

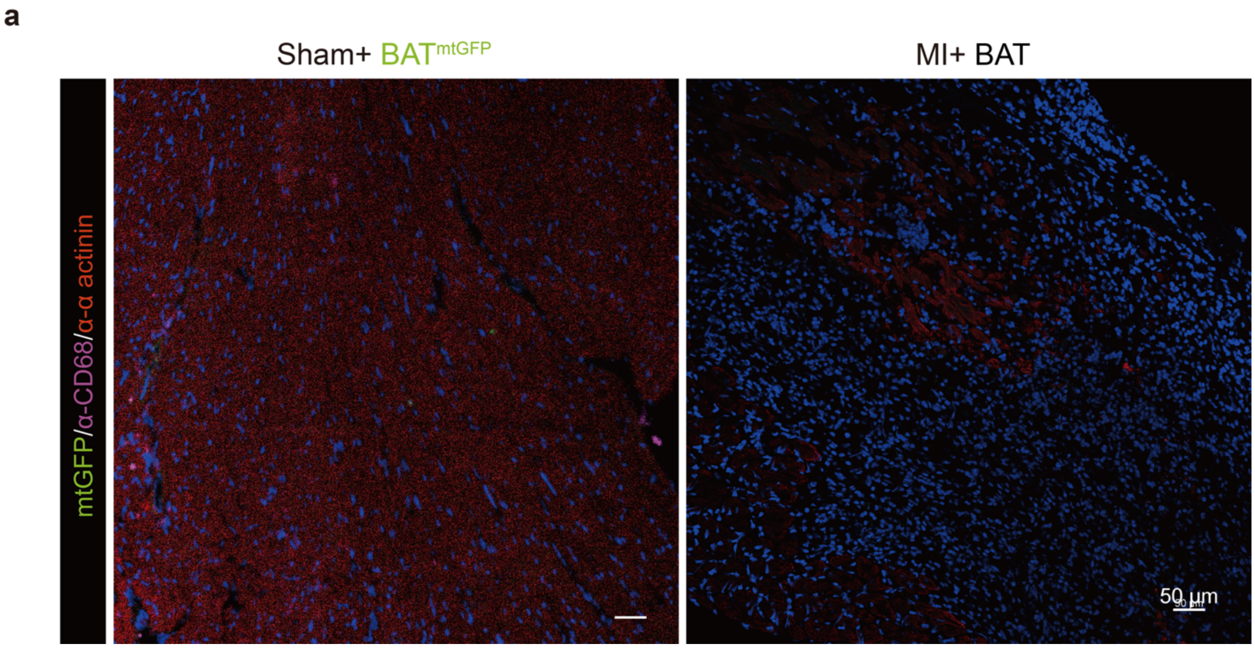

Fig. R34 a, Biological controls (sham+BAT^{mtGFP}; MI+BAT) for Fig. 2g. Scale bar: 50μm.

(4) Immunofluorescence images throughout the manuscript are of relatively low resolution.

Response:

Thank you for pointing out the resolution issue.

In the revised manuscript, we have substantially improved both the acquisition and presentation of the images. We exported all panels at their original native resolution, adjusted brightness and contrast within the linear range to enhance visibility, and added

clearer labels and scale bars. Where appropriate, we also provide larger fields of view together with high-magnification insets. Also, we re-conducted experiments and acquired key images on a laser-scanning confocal microscope using high-resolution settings. We hope these changes improve the readability of the immunofluorescence images throughout the revised manuscript.

(5) In Fig. 4e, ACTB levels are dramatically reduced post-MI, raising concern about loading controls. This should be clarified or alternative loading controls used.

Response:

We are grateful for this careful and important observation.

We agree that Actb may not be an appropriate loading control in the context of MI-induced stress, and that variability in Actb could confound the interpretation of VPS35 changes in our original Fig. 4e. To address this, we re-collected BAT samples at MI day 1, 3, 7 and conducted the BAT Western blots using total protein staining as the loading reference instead of Actb. Total loading protein was used as reference in all western blots in the revised manuscript.

VPS35 and BECN1 were quantified and normalized to the total protein signal in each lane (Fig. R35). This analysis shows a statistically significant, time-associated increase in VPS35 and BECN1 levels at MI d3 and the return towards baseline by MI d7.

a

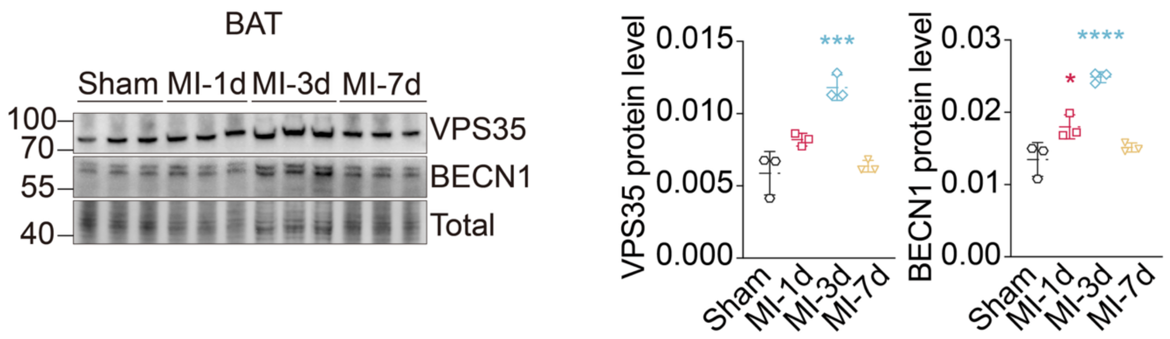

Fig. R35 a, Time course of VPS35 and BECN1protein expression in BAT after MI. Representative immunoblots and quantification of BECN1 and VPS35 in BAT lysates from sham, MI-1 d, MI-3 d, and MI-7 d mice (mean±s.d., n=3, One-way ANOVA test followed

by Bonferroni method). **The above new data are included in Fig. 4 in the revised manuscript.**

Reviewer #4 (Remarks to the Author):

This manuscript by Shi and co-workers describe their study with the aim to demonstrate the impact of BAT on the heart post-MI. The authors show that MI stimulates the release of BAT-derived mitochondria-derived vesicles that influence the inflammatory status of cardiac macrophages. The MDV cargo, dominantly OXPHOS proteins, mediates its cardioprotective effects. The BAT-heart connection, taken into considerations the BATtokines: FGF21, IL6, NRG4, 12,13-diHOME and BMP3b are a timely topic to understand and appreciate interorgan communication. Especially if BAT- derived exosomes delivering iNOS to sites of ischemia and as BAT is rich in mitochondria which they release under stress.

However, there are some issues that need to be addressed. One of them is that it would be appreciated if a native English speaker would be able to read the manuscript and if the material and methods are not organized in a more logical manner.

Response：

We are grateful to your thoughtful summary of our study and for emphasizing the broader significance of the BAT-heart axis and BAT-derived secretory factors (including BATokines such as FGF21, IL-6, NRG4, 12,13-diHOME and BMP3b, as well as BAT-derived exosomes) in interorgan communication after MI. Also, we are grateful for your supportive comments and your acknowledgement of our findings.

In response to your concerns regarding language and manuscript structure, we have undertaken a comprehensive revision of the text with input from native English-speaking colleagues to improve clarity, precision and readability. In addition, we have reorganized the Materials and Methods section to more closely follow the logic of “in vivo experiment methods-in vitro experiment methods-statistical and bioinformatics methods”, with clearer subheadings. We hope that these changes can address your concerns and make the manuscript more accessible.

Other questions are:

Line 102: The authors write that data were excluded only when the mice died during surgery or before the time points of analysis. The first argument is acceptable, but during the course of the experiment is very relevant and excluding may introduce a bias.

Figure 1D&E: The number of animals in the MI group vs BAT transplanted animals differs significantly which may skew the data. What is the rationale behind this discrepancy? Furthermore, an EF of less than 10% is extremely low, which does not translate to the thickness of the wall. Can the authors explain this? Furthermore, the LV wall thickness is much thicker in F compared to E. Finally, it is not clear what Base to Apex means in figure 1F.

Response:

We are deeply grateful for your important comments on the methodological limitation.

In the original submission we used a relative proximal (high) LAD ligation, which resulted in extensive infarcts, profoundly decreased ejection fraction and substantial early mortality within the first week after MI. Recognizing that this model was limited our ability to evaluate longer-term cardiac remodeling, we refined the protocol to a standardized mid-LAD ligation, which yields reproducible infarct sizes with moderate systolic dysfunction and markedly improved early survival. All in vivo data presented in the revised manuscript are based on this refined mid-LAD protocol, and there was no mouse died before the defined endpoints (MI Day 28).

Other improvements in study design include:

i) In the revised manuscript, left ventricular function was assessed by serial echocardiography at baseline (a day before MI induction), day 7, day 14, and day 28 after MI induction. On day 28 after MI induction, hearts were

harvested. Ventricular remodeling and fibrosis were assessed by Masson's trichrome staining and Picrosirius Red staining.

ii) We also increased group sizes across groups to enhance statistical power. Now the sample size is balanced and increased.

iii) We have standardized the surgical procedure, adopting a moderate LAD ligation technique to reduce mortality and minimize survival bias.

LV wall thickness readouts was removed from the revised manuscript, retaining EF, FS and histological fibrosis as the primary functional and structural endpoints. The reasons are, (i) LV wall thickness is highly sensitive to regional heterogeneity after LAD ligation and (ii) without a clear anatomical definition of the measurement site, such data are difficult to interpret. As a result, LV wall thickness could be a highly viriable and regionally biased parameter in the murine LAD-ligation model, and therefore is not a robust primary readout of cardiac remodeling.

In the original submission, LV wall thickness in Fig. 1f was measured at day 4 post-MI, whereas the measurements in Fig. 1e were obtained at day 8 post-MI. Because the LV wall typically thins as post-MI remodeling progresses, the earlier time point shows a thicker wall, which explains why the LV wall in Fig. 1f appears thicker than in Fig. 1e.

In Fig. 1f, “base to apex” refers to the anatomical orientation of serial short-axis sections of the left ventricle, ordered from the basal level (at the valve plane) toward the apical level. To avoid confusion, we have now revised the figure to present the serial sections.

The results of the revised data for four major phenotype experiments are listed below (Fig. R36).

1. Pericardial BAT transplantation attenuated adverse post-MI remodeling, as BAT-grafted mice showed higher EF/FS and smaller fibrotic areas than MI controls, whereas BAT-deficient mice had worse function and more fibrosis. WAT

transplantation provided no benefit, while cold-activated BAT further improved systolic function, indicating a BAT-related, thermogenesis-enhanced cardioprotective effect.

2. Intravenous delivery of adipose-derived MDVs (30 µg protein per mouse) after MI improved LV systolic function, limited adverse remodeling, and reduced fibrosis compared with vehicle controls. BAT- and WAT-derived MDVs exerted comparable benefits, indicating that MDVs from either adipose depot possess intrinsic cardioprotective activity.
3. Transplantation of wild-type BAT improved EF/FS over 4 weeks after MI and reduced infarct fibrosis, whereas BAT from *Becn1*$^{+/-}$ donors, despite similar engraftment, failed to confer functional or structural benefit, indicating that BECN1 in donor BAT is required for BAT-mediated cardioprotection.
4. Intravenous delivery of wild-type BAT-MDVs improved LV function and reduced fibrosis, whereas MDVs from *Becn1*$^{+/-}$ BAT showed markedly attenuated effects, demonstrating that BECN1 is dispensable for bulk MDV yield but essential for the cardioprotective efficacy of BAT-derived MDVs in vivo.

The inconsistency between the effects of WAT-transplantation and WAT-MDV treatment can be explained by the much lower yield of MDV from WAT, and the secretion of cardio deleterious lipids and adipokines from WAT, thereby unmasking the intrinsic cardioprotective capacity of adipose-derived MDVs.

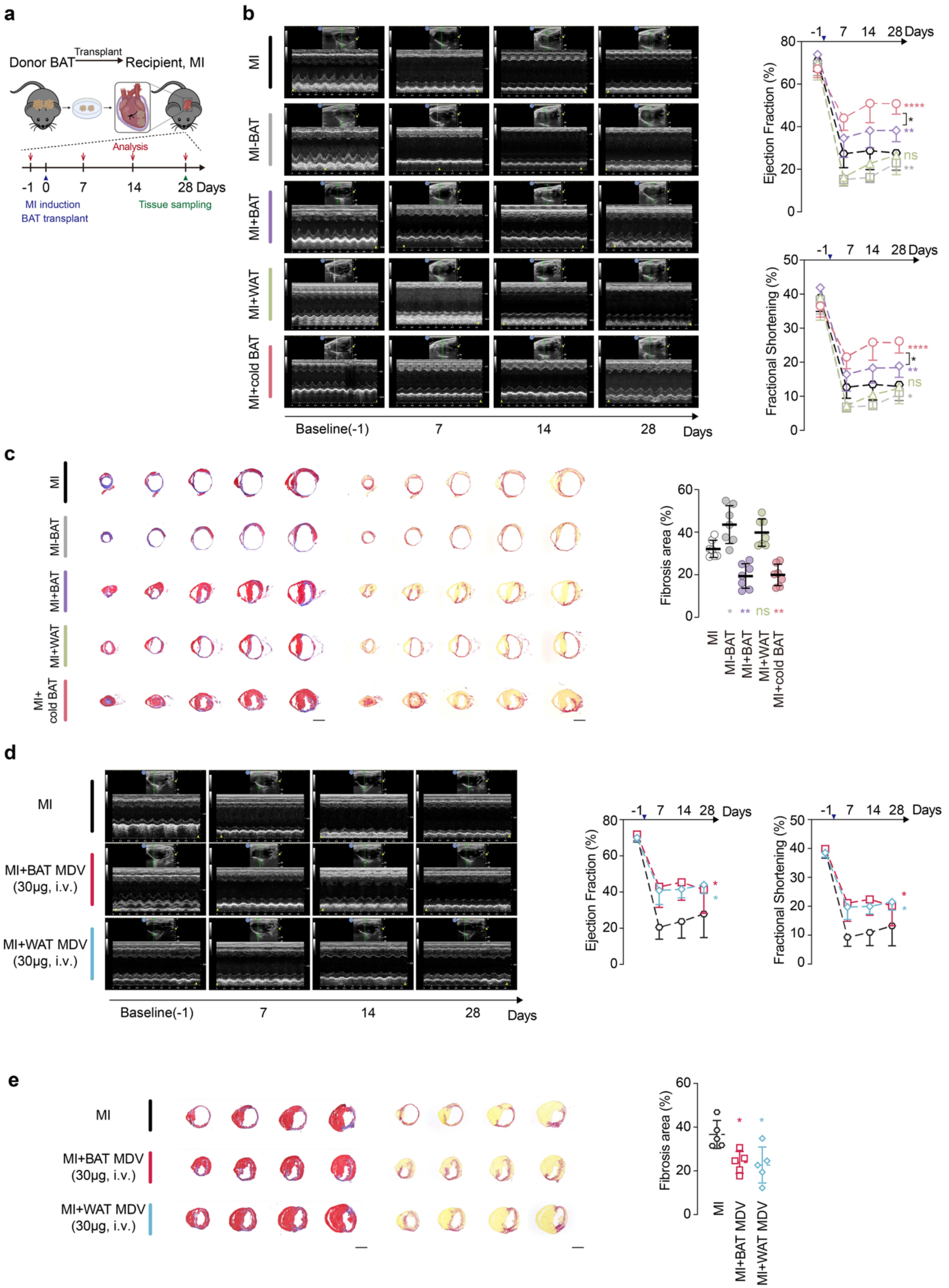
a
Donor BAT
Transplant
Recipient, MI
Analysis
-1 0 7 14 28 Days
MI induction
BAT transplant
Tissue sampling
b
MI
MI-BAT
MI+BAT
MI+WAT
MI+cold BAT
Baseline(-1) 7 14 28 Days
Ejection Fraction (%)
Fractional Shortening (%)
ns
c
MI+
cold BAT
Fibrosis area (%)
MI
MI-BAT
MI+BAT
MI+WAT
MI+cold BAT
d
MI
MI+BAT MDV
(30μg, i.v.)
MI+WAT MDV
(30μg, i.v.)
e
MI+BAT MDV
MI+WAT MDV

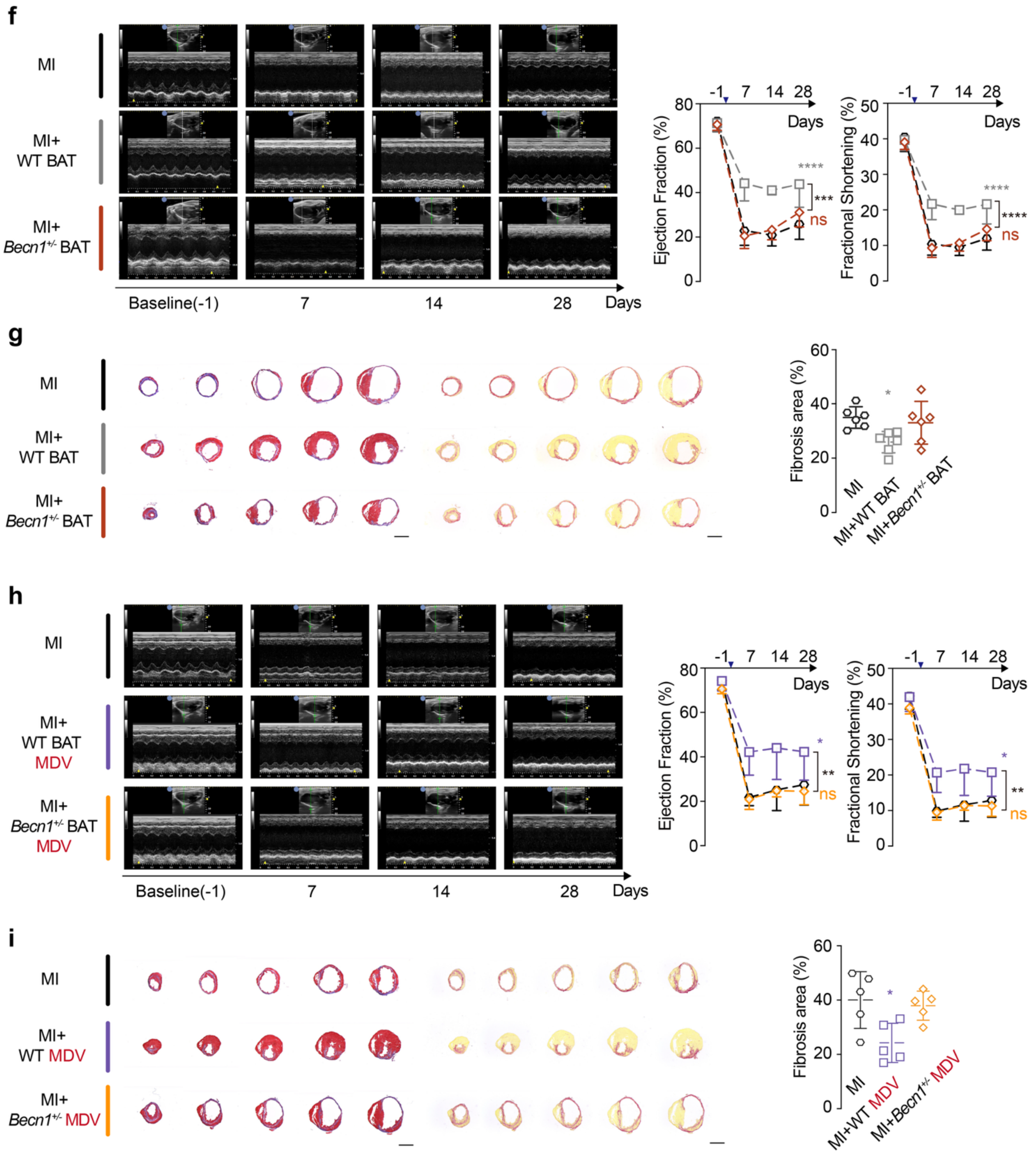

Fig. R36 **a,** Experimental timeline for MI induction, adipose tissue pericardial transplantation, and outcome assessments. Echocardiography was performed at baseline (day −1) and on days 7, 14, and 28. Hearts were harvested on day 28 for Masson's trichrome and Sirius Red staining. **b,** Representative B-mode and M-mode echocardiography of BAT-receiving mice at baseline and days 7, 14, and 28. Ejection

fraction and fractional shortening were quantified (mean±s.d., n=7, repeated two-way ANOVA and Holm-Sidak post hoc, *$p < 0.05$, **$p < 0.01$, ****$p < 0.0001$). **c,** Representative Masson and Sirius Red staining images of mouse hearts at day 28. Fibrosis percentage was quantified (mean±s.d., n=7, One-way ANOVA test followed by Bonferroni method, *$p < 0.05$, **$p < 0.01$, ***$p < 0.001$, ****$p < 0.0001$). Scale bar: 2mm. **d,** Representative B-mode and M-mode echocardiography of adipose tissue-derived MDV-treated mice at baseline and days 7, 14, and 28. Ejection fraction and fractional shortening were quantified (mean±s.d., n=5, repeated two-way ANOVA and Holm-Sidak post hoc, *$p < 0.05$). **e,** Representative Masson and Sirius Red staining images of MDV-treated mouse hearts at day 28. Fibrosis percentage was quantified (mean±s.d., n=5, One-way ANOVA test followed by Bonferroni method, *$p < 0.05$). Scale bar: 2mm. **f,** Representative B-mode and M-mode echocardiography of WT or *Becn1*$^{+/-}$ BAT-receiving mice at baseline and days 7, 14, and 28. Ejection fraction and fractional shortening were quantified (mean±s.d., n=6, repeated two-way ANOVA and Holm-Sidak post hoc, ***$p < 0.001$, ****$p < 0.0001$). **g,** Representative Masson and Sirius Red staining images of WT or *Becn1*$^{+/-}$ BAT-receiving mice hearts at day 28. Fibrosis percentage was quantified (mean±s.d., n=6, One-way ANOVA test followed by Bonferroni method, *$p < 0.05$). Scale bar: 2mm. **h,** Representative B-mode and M-mode echocardiography of WT or *Becn1*$^{+/-}$ MDV-treated mice at baseline and days 7, 14, and 28. Ejection fraction and fractional shortening were quantified (mean±s.d., n=5, repeated two-way ANOVA and Holm-Sidak post hoc, *$p < 0.05$, **$p < 0.01$). **i,** Representative Masson and Sirius Red staining images of WT or *Becn1*$^{+/-}$ MDV-treated mice hearts at day 28. Fibrosis percentage was quantified (mean±s.d., n=5, One-way ANOVA test followed by Bonferroni method, *$p < 0.05$). Scale bar: 2mm. **The above new data are included in Fig. 1, 3, and 5 in the revised manuscript.**

Line 463-464: The authors write: suggested a link between cardiac injury and BAT reduction. This can be just a coincidence.

Response：

We appreciate this important comment. We acknowledge that the initial description (“suggested a link between cardiac injury and BAT reduction”) was not sufficiently precise, as it may overstate what can be concluded from a temporal association alone. In the revised manuscript, we have tempered this statement and now describe the observation in a more strictly descriptive manner “Myocardial infarction induced by left anterior descending (LAD) ligation caused an acute reduction in BAT mass, which gradually recovered to near-baseline levels by two weeks post-MI (Fig. 1a). This transient BAT atrophy occurs within the same time window as early post-injury remodeling.” We present the data as a temporal pattern that motivated our subsequent interventional experiments (BATectomy, BAT transplantation in vivo experiments), which provide the evidence for a BAT-heart axis.

It is not clear from the data presented if the macrophage co-localization of BAT-MDVs is an active process or just because macrophage are programmed to clear damage areas. The IF images presented in figure 2 are difficult to appreciate as the resolution is not very good. In 2B&D, the BATZsGreen is difficult to see, and the same problem is for 2C.

Response:

In response to your concern about image quality, we exported all panels at their original native resolution, adjusted brightness and contrast within the linear range to enhance visibility, and added clearer labels and scale bars. Where appropriate, we also provide larger fields of view together with high-magnification insets. Also, we re-conducted experiments and acquired key images on a laser-scanning confocal microscope using high-resolution settings (Fig 2, Extended Fig. 2 and 3). We hope these changes improve the readability of the immunofluorescence images throughout the revised manuscript.

To strengthen the conclusion that macrophages are the predominant BAT-MDV-positive population in vivo, we have also added new flow cytometry data from $BAT^{ZsGreen}$-recipients (Fig. R37). These analysis demonstrate that approximately 60–70% of ZsGreen+ immune cells in the infarcted myocardium are macrophages, with smaller fractions corresponding to neutrophils and monocytes, supporting a preferential enrichment of BAT-derived vesicles in cardiac macrophages.

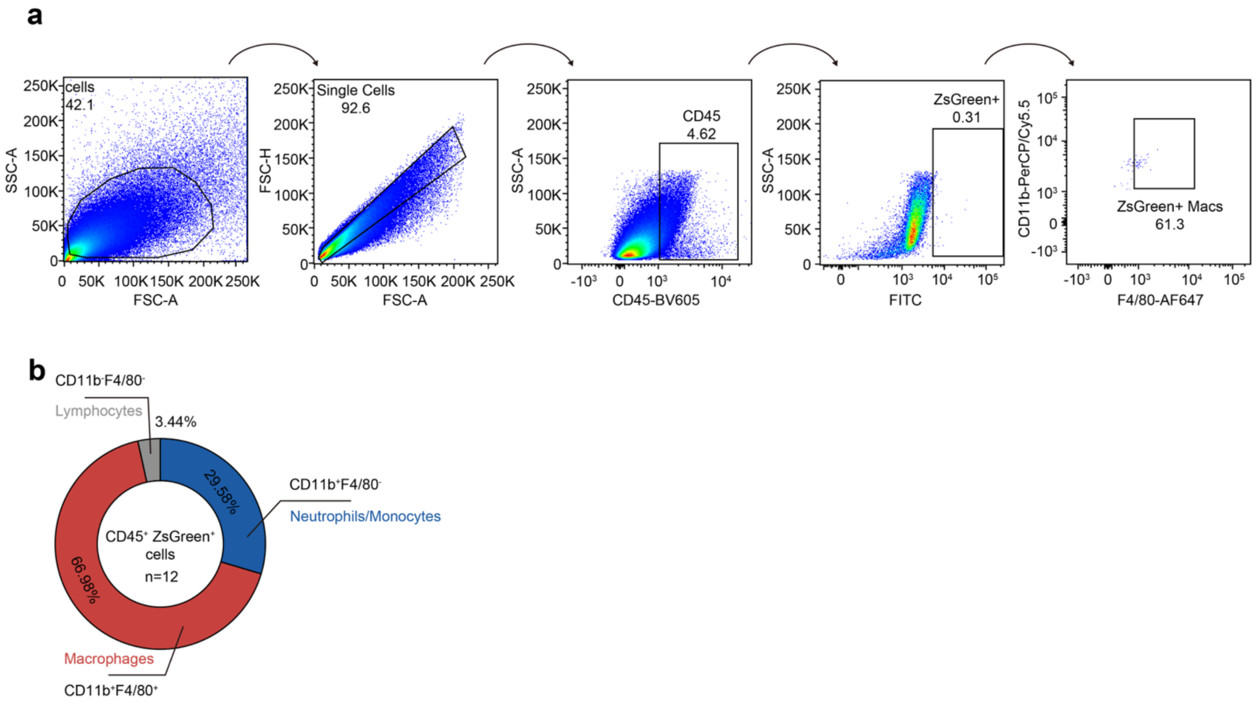

Fig. R37 a, Flow-cytometric gating strategy for identifying BATosome-recipient immune cells in infarcted hearts from $BAT^{ZsGreen}$-receiving mice at MI 4d. After debris exclusion and singlets selection, leukocytes were gated as $CD45^+$ cells, followed by $ZsGreen^+$ events and $CD11b^+F4/80^+$ macrophages within the $CD45^+ZsGreen^+$ population. **b**, Quantification of immune cell subsets among $ZsGreen^+CD45^+$ cells in infarcted hearts from $BAT^{ZsGreen}$-receiving mice at MI 4d. Data are presented as mean percentages of each subset among total $ZsGreen^+CD45^+$ cells (n as indicated). **The above new data are included in Fig. 2 in the revised manuscript.**

To further address whether macrophage uptake of BAT-MDVs is an active, energy-dependent process, we performed new co-culture experiments using mtGFP-labelled BAT-MDVs and iBMDM cells under conditions that selectively impair endocytic or phagocytic activity. Specifically, we compared MDV internalization at 37 °C (physiological temperature), 4 °C (which largely suppresses ATP-dependent vesicle uptake), and 37 °C in the presence of rotenone plus oligomycin, which inhibit

mitochondrial respiration and ATP synthesis. These experiments showed that incubation at 4 °C or at 37 °C with rotenone plus oligomycin reduced MDV uptake by ~50% (Fig. R38), indicating that macrophage internalization of BAT-MDVs comprises both ATP-dependent and ATP-independent pathways.

a

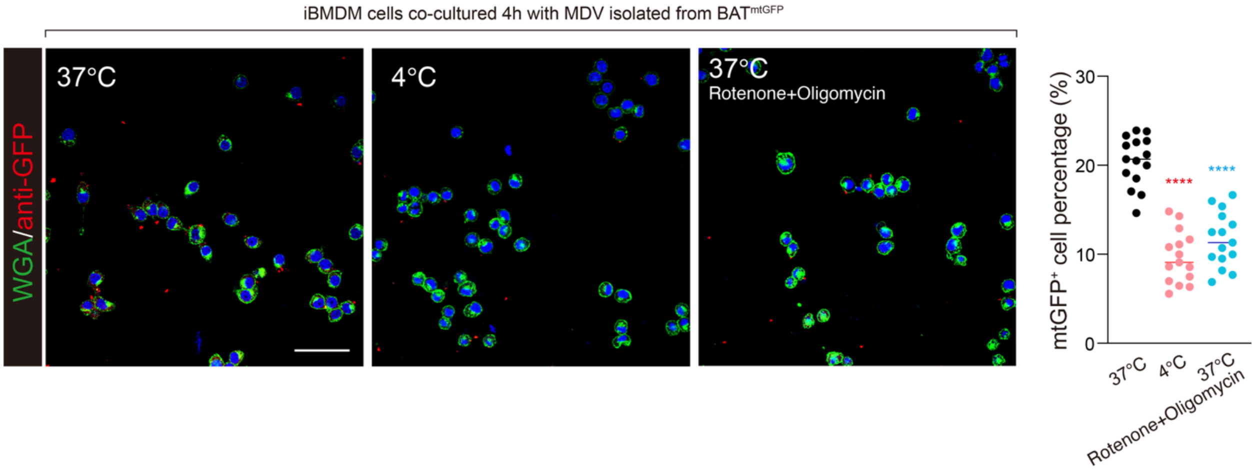

Fig. R38 a, iBMDM cells co-cultured with MDVs (isolated from BATmtGFP, at the concentration of 30 µg/mL) for 4 h at 37℃, 4℃, 37℃ with the presence of rotenone (100nM) and oligomycin (1µM). The cells were stained with anti-GFP antibody (red) and cell membrane (WGA, green). The mtGFP+ cell percentage was calculated(mean. One-way ANOVA test followed by Bonferroni method, ****p < 0.0001, n=15 fields/group).Scale bar: 50µm.

Line 551-553: If the numbers of MDVs were lower in WAT compared to BAT, is it the number that makes the difference of the content? Especially if (line 558) equal amount of tissue is used and not number of MDVs

Response:

Thank you for raising this important point.

In the original manuscript, MDVs were isolated from equal tissue mass (800 mg BAT or WAT). Because WAT produces substantially fewer MDVs than BAT (Fig. 3b), mice in the WAT-MDV group effectively received a much lower MDV dose. In the revised study, we therefore normalized MDV administration by MDV protein (take a small proportion of isolated MDVs and quantify the protein concentration by BCA method), injecting 100μL MDV (at the concentration of 300μg protein/mL) per mouse (i.v.) from either WAT or BAT. Under these dose-matched conditions, WAT- and BAT-derived MDVs produced very similar improvements in ejection fraction, fractional shortening, and fibrosis across 7-28 days post-MI (Fig. R39).

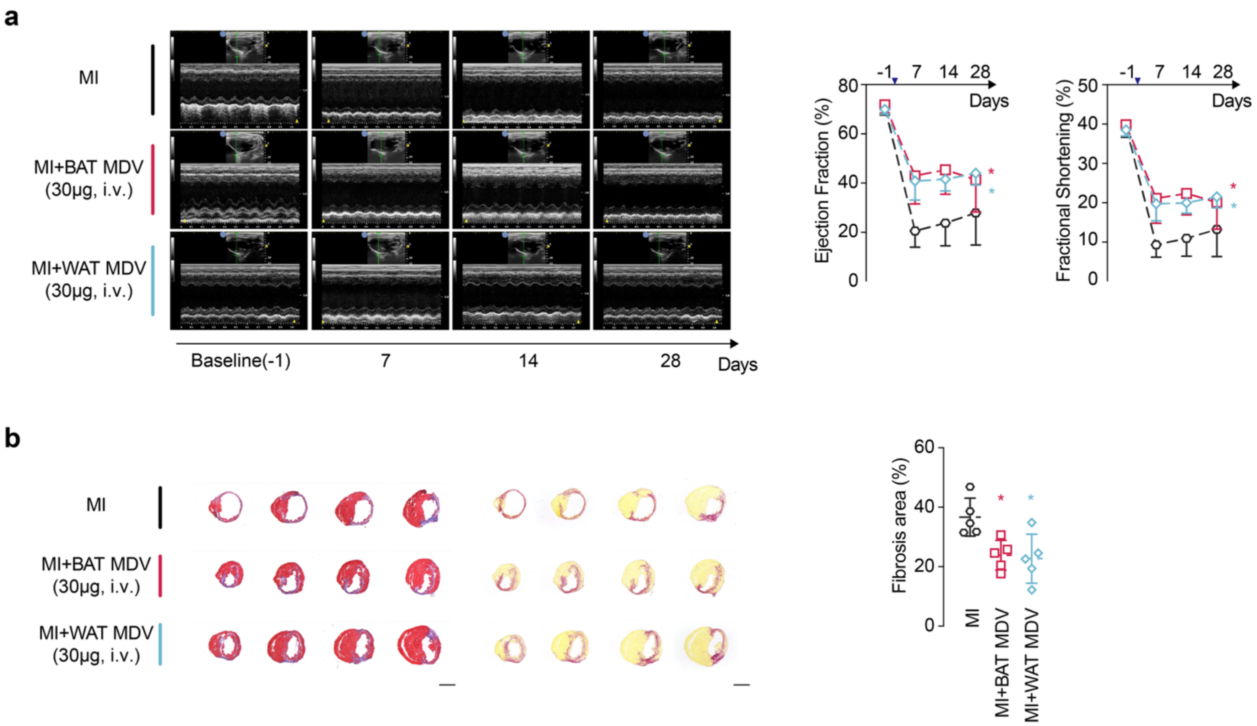

Fig. R39 a, Representative B-mode and M-mode echocardiography of adipose tissue-derived MDV-treated mice at baseline and days 7, 14, and 28. Ejection fraction and

fractional shortening were quantified (mean±s.d., n=5, repeated two-way ANOVA and Holm-Sidak post hoc, *$p < 0.05$). **b,** Representative Masson and Sirius Red staining images of MDV-treated mouse hearts at day 28. Fibrosis percentage was quantified (mean±s.d., n=5, One-way ANOVA test followed by Bonferroni method, *$p < 0.05$). Scale bar: 2mm. **The above new data are included in Fig. 3 in the revised manuscript.**

Figure 3D: as previously mentioned, it is not clear what the Base -> apex refers to. Furthermore, in the comparison the control is missing to appreciate the impact of MDVs on fibrosis. The amount of fibrosis 4 days after MI is quite intensive. Is this true fibrosis or scar tissue? The process normally takes some more time. Again, the control without injection is missing. Figure 3G is not informative. How did the authors distinguish LV-EVs from BAT-EVs post MI?

Response:

We apologize for the unclarity in the initial manuscript.

“Base to apex” refers to the orientation of serial short-axis sections of the heart.

In the revised manuscript, we have moved the primary assessment of fibrosis to a later time point and added the missing control group (Fig. R38). Specifically, Masson/Sirius Red staining and quantitative fibrosis measurements are now performed at 28 days post-MI, a time window that better reflects fibrosis and the comparison now includes MI controls.

In the EV experiments we did not attempt to separate circulating LV-derived EVs from BAT-derived EVs in vivo. Instead, the preparations referred to as “LV-EVs” and “BAT-EVs” were obtained from ex vivo tissue explant cultures of left ventricle and BAT, respectively, after MI. Fresh LV or BAT tissue was dissected, cut into small pieces, with cautions to avoid mechanical disruption of cells, then extensively rinsed to remove blood components and debris, and then incubated in serum-free medium at 37 °C with 5% CO_2 for 24 h to allow EV release. After 24 h, the conditioned medium was collected and EVs were isolated and analyzed. Thus, “LV-EVs” and “BAT-EVs” in this figure denote EVs secreted by LV and BAT explants under identical culture conditions, rather than distinct circulating EV pools in the post-MI mouse.

The rationale to check the genes in figure 4a is not clear. What does Id2 (line 579) tell us?

Response:

In Fig. 4a we selected genes with established roles in brown adipose tissue (BAT) identity, metabolism and mitochondrial quality control.

In experimental settings, activation of brown adipose tissue is usually evaluated by a coordinated transcriptional program. The core readout is Ucp1, PGC-1α, and Dio2. The co-activator Ppargc1a (PGC-1α) is rapidly induced by sympathetic stimulation and cold, drives Ucp1 transcription.

To capture the accompanying lipid-metabolic remodeling, genes such as Elovl3, a BAT-enriched very-long-chain fatty acid elongase that is selectively upregulated by cold and Cidea, a lipid-droplet–associated protein enriched in BAT whose expression correlates with Ucp1 and thermogenic capacity.

The thermogenic/lipolytic gene assessment is complemented by brown-fat identity markers such as Prdm16, Pparg and the beige/brown regulator Id2.

Id2 is a helix–loop–helix transcriptional regulator that functions upstream of Pparg to promote adipogenesis, such that Id2 induction enhances Pparg expression and lipid accumulation, whereas Id2 deficiency reduces adiposity and impairs adipocyte differentiation in vivo and in vitro (Mol Endocrinol. 2008 PMID: 18562627; J Diabetes Res. 2016. PMID: 27144179).

Together with Ucp1, Ppargc1a and Elovl3, Id2 provides information on whether MI primarily engage a thermogenic program (probably non-canonical) in BAT with only modest changes in classical brown-fat identity.

To probe mitochondrial dynamics and MDV biogenesis, we also included genes linked to mitochondrial dynamics. The selected genes were reported to be associated with MDV biogenesis and release. (see Discussion.The molecular pathways underlying MDV production have not been fully elucidated. MDVs display significant heterogeneity in their biogenesis mechanism, cargo composition, and fates. Mitochondria outer

membrane TOMM20$^+$ MDVs start from *Miro1/2* anchoring and *Drp1* scission, then are pulled along cell filaments and degraded by lysosomes[27]. Mitochondria matrix PDH$^+$ mtDNA$^+$ MDVs are produced by *Snx9* under the condition of mitochondria fumarate overload[41]. Mitochondria inner membrane MDVs are generated through the budding of the inner membrane vis VDACs and are incised by ESCRT machinery[42]. VPS35 has been reported to control vesicle transport between the mitochondria and peroxisomes[31].)

Line 602-604: If autophagy is inhibited, is MDV release hampered? Is Becn1 involved in the MDV release or, more likely, in a biological response linked to autophagy? How does this impact cardioprotection, as a direct relation is not clear.

Response:

We really appreciate this insightful question.

To directly test whether autophagy contributes to MDV release, we performed additional experiments in differentiated brown adipocytes treated with cardiac DAMPs in the presence of late-stage autophagy/lysosome inhibitors. Mature brown adipocytes were pre-incubated with chloroquine (10 μM) or bafilomycin A1 (50 nM) for 2 h, followed by co-stimulation with DAMPs for 4 h, then MDVs released wad quantified. Neither chloroquine nor bafilomycin A1 produced a statistically significant reduction in the number of TOMM20⁺PDH⁻ MDVs per cell, indicating blockade of late autophagic flux does not substantially impair DAMP-induced MDV release (Fig. R40).

a

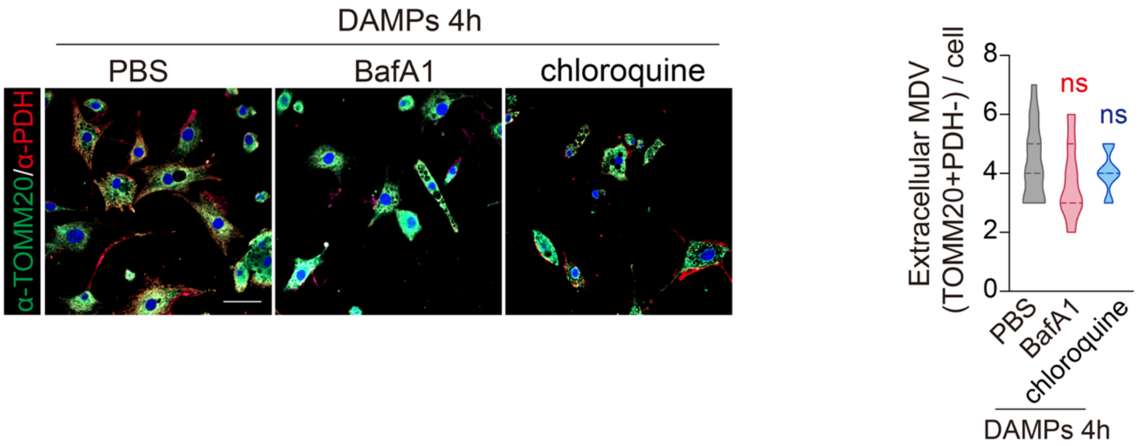

Fig. R40 a, Mature brown adipocytes were pre-incubated with chloroquine (10 μM) or bafilomycin A1 (50 nM) for 2 h, followed by co-stimulation with DAMPs for 4 h. The cells were stained for TOMM20 (green) and PDH (red). Extracellular TOMM20⁺PHD⁻ MDVs were quantified (mean. One-way ANOVA test followed by Bonferroni method, ns, $p > 0.05$, n=15 cells/group). Scale bar: 50μm.

Our data support a model in which Becn1 participates in a specific process of MDV biogenesis and cargo sorting that could overlap with, but not identical to, bulk autophagy. Pharmacological inhibition of autophagy (3-MA) reduces DAMP-induced TOMM20+PDH- MDV release, but increases TOMM20+PHD+ MDV release, so that total MDV remained unaltered. Consistently, the overall MDV yield was not affected in *Becn1*$^{+/-}$ BAT, while proteomic profiling revealed a loss of inner mitochondrial membrane/OXPHOS components, particularly complex V, in *Becn1*$^{+/-}$ MDVs.

These findings suggest that, in the BAT-MDV system, *Becn1* is more critical for controlling the cargo of MDV, namely the enrichment of IMM/OXPHOS proteins, than for simply determining the quantity of vesicles released. These low-complex V MDVs from *Becn1*$^{+/-}$ BAT are less effective at restoring mitochondrial function and promoting a pro-reparative phenotype in cardiac macrophages, indicating that MDV bioenergetic integrity is required for full cardioprotection.

At the same time, we agree that *Becn1* may also influence cardiac outcome through additional autophagy-related pathways independent of MDVs.

Figure 4E, the increase in VPS35 is only due to a difference in beta actin. This is therefore not the correct housekeeping gene to correlate expression to and should be corrected. Figure 4I has the same bias.

Response:

We are grateful for this important comment.

In response, we performed additional Western blot experiments and re-analyzed VPS35 expression using total protein stain (Fig. R41).

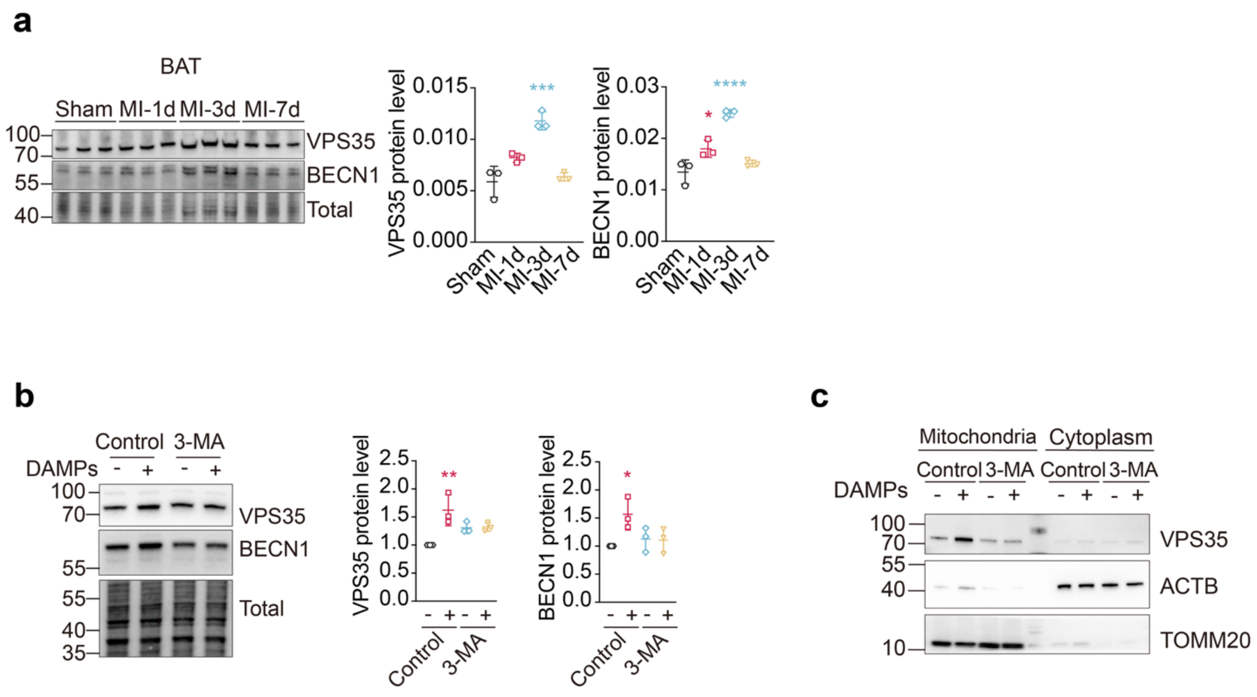

Fig. R41 a, Time course of VPS35 and BECN1protein expression in BAT after MI. Representative immunoblots and quantification of BECN1 and VPS35 in BAT lysates from sham, MI-1 d, MI-3 d, and MI-7 d mice (mean±s.d., n=3, One-way ANOVA test followed by Bonferroni method). **b**, Representative western blots of brown adipocytes incubated with complete media(control) or 3-MA (10mM) and stimulated with heart DAMPs (20μg/mL). Quantification of VPS35 and BECN1 protein relative levels normalized to total loading protein. (mean±s.d., n=3 independent experiments, paired Student's t-test). **i**, Representative mitochondria and cytoplasm western blots of brown

adipocytes incubated with complete media(control) or 3-MA (10mM) and stimulated with heart DAMPs (20μg/mL) (n=3 independent experiments). . **The above new data are included in Fig. 4 in the revised manuscript.**

The date presented in figure 5B is not visible. Again, figure D and E are difficult to appreciate, is there no cardioprotection because the MDVs now are not taken up by the cells they normally would modify? As the mechanism of action is not clear, figure 6 is difficult to appreciate.

Response:

We appreciate your concern that reduced cardioprotection of Becn1$^{+/-}$ BAT-derived MDVs might arise from impaired transfer, or uptake. To address this, we transplanted WT BAT^{mtGFP} or *Becn1*$^{+/-}$ BAT^{mtGFP} into MI mice and quantified BAT-derived MDV transfer to the heart using anti-GFP staining 4 days post-MI. The percentage of GFP^{+} cells were comparable between the two groups (Fig. R42), indicating similar MDV delivery and cellular uptake in vivo.

These data argue that the loss of cardioprotection with *Becn1*$^{+/-}$ BAT-MDVs is unlikely to be explained by reduced transfer or uptake and instead support the hypothesis that MDV cargo quality (particularly complex V level) is the key to the effects on cardiac macrophages and post-MI remodeling.

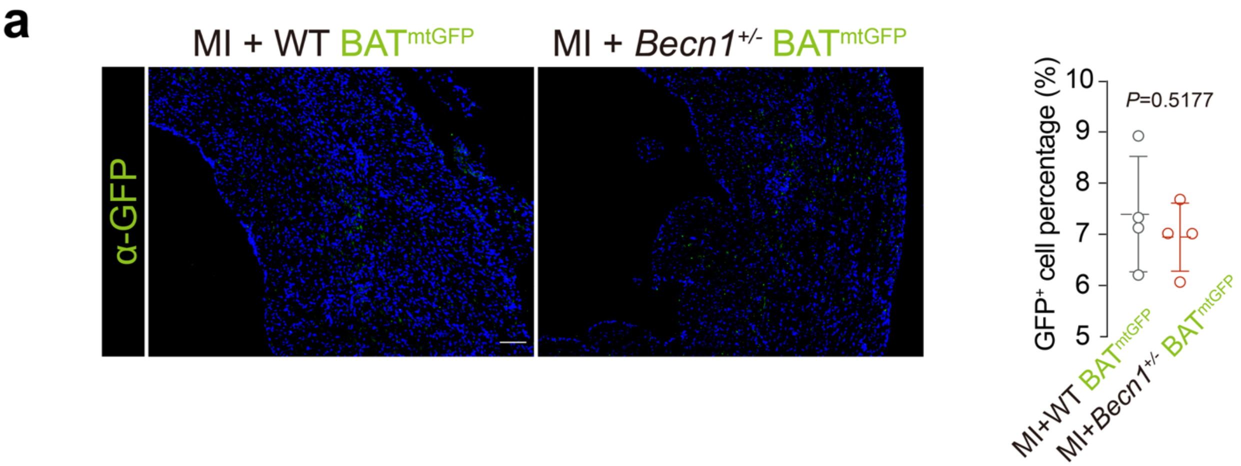

Fig. R42 a, Representative confocal images of BAT^{mtGFP}-receiving mice at MI 4d, stained for GFP (green) and nuclei (DAPI, blue). Scale bar: 100μm. Mt-EGFP^{+} cell percentage was quantified (mean±s.d., n=4, unpaired Student's t-test). **The above new data are included in Fig. 5 in the revised manuscript.**

Figure 7 feels a bit out of scope of the paper. If in the graphical abstract OXPHOS is key, more mechanistic validation is needed to support that finding.

Response：

We appreciate this helpful comment.

Our intention with Figure 7 was to provide a translational correlation of the complex V-macrophage axis defined in the mouse and cell experiments. This analysis emphasizes that, among the major cardiac cell populations, only macrophages display a significant reduction of complex V score in heart failure compared with controls, and that macrophage complex V score exhibits the strongest positive association with LVEF.

Mechanically, we now (i) pharmacologically inhibited complex V activity in MDVs before the co-culture with iBMDM cells, which markedly attenuated the ability of MDVs to induce an anti-inflammatory/pro-reparative program in macrophages, and (ii) overexpressed ATP5A1 to augment complex V subunit level, which partially rescued the macrophage to inflammatory phenotype (Fig. 7). These new data validated that the bioenergetic unites of MDV, particularly complex V/ATP5A1, is required for cardioprotection.

Peer Review File

Response to reviewers for "**Mitochondria-derived-vesicles with bioenergetic units from brown adipose tissue attenuate cardiac remodeling post-myocardial infarction**" by "Tingting Shi, …, Yaozu Xiang." (NCOMMS-25-22893A)

REVIEWER #1 2

Reviewer #1

The authors have made substantial revisions to the manuscript, which have greatly strengthened the paper. However, a few points still require attention.

1. The magnification and clarity of Figure 2c, d and R3 are insufficient to convincingly demonstrate the localization of ZsGreen. The images barely allow for the identification of individual cells, let alone the resolution of subcellular structures. They are certainly not adequate for distinguishing mitochondrial membrane structures. Please do provide images with at least enough resolution to distinguish individual mitochondria and improve the image brightness.

Response:

In the final manuscript, we have replaced the panels with higher-magnification images presented at the original resolution. We have added enlarged insets of representative regions of interest and included single-channel images to allow clear visualization of ZsGreen and mitochondrial markers separately. Image brightness and contrast were improved across channels without altering spatial relationships (Fig. R1).

a

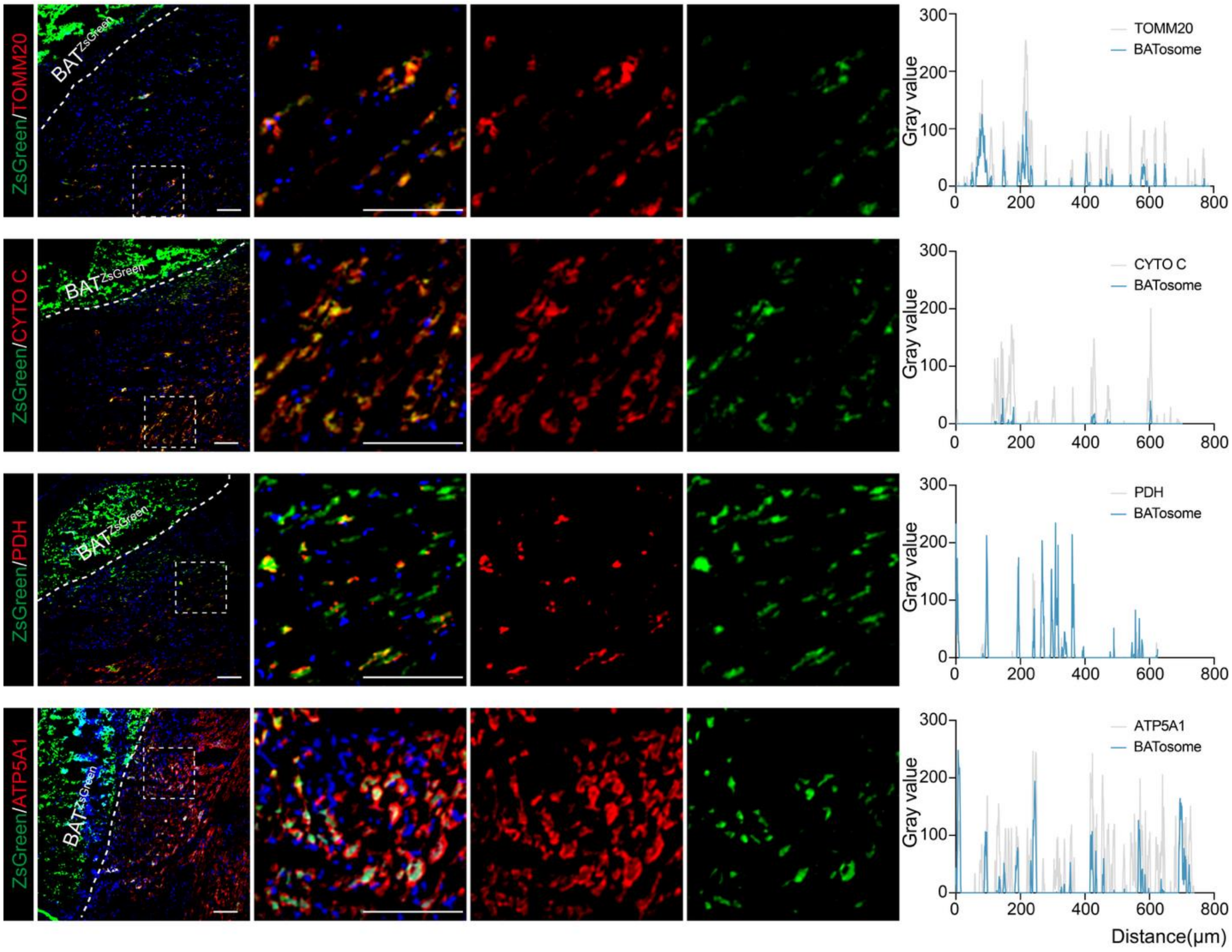

Fig. R1 a, Representative confocal images of left ventricles in BAT^{ZsGreen}-receiving mice at sham or MI 4d, stained for TOMM20 or Cytochrome C or PDH or ATP5A1(red), and nuclei (DAPI, blue). Co-localization analysis of ZsGreen^{+} BATosomes with mitochondrial markers in the infarct myocardium. Scale bar: 100μm. **The above data are included in Fig. 2c in the revised manuscript.**

Although the images with higher magnification and higher-resolution allow visualization at the cellular level and identification of individual cells within the tissue, organelle-level ultrastructural details, particularly mitochondrial membrane architecture, remain technically challenging for tissue section imaging. Super-resolution confocal imaging in cardiac tissue is demanding due to substantial light scattering, autofluorescence, and

limited antibody penetration. In post-infarction myocardium, extensive extracellular matrix remodeling and inflammatory infiltration further limited super-resolution imaging.

We further clarify the hierarchy of evidence in the revised manuscript. Immunofluorescence imaging is used to assess marker-level co-localization associations and cell-type localization, whereas ultrastructural characterization of vesicles is supported by electron microscopy, which provides the necessary resolution of subcellular structures. Therefore, we revised the Results section to moderate the interpretation of the confocal data, limiting our conclusions to cellular localization and marker-level association, and avoiding any implication of mitochondrial membrane ultrastructure.

Manuscript edits:

Results

BAT-MDVs transferred to cardiac macrophages

Four days after MI, $ZsGreen^+$ signals were detected in the border and infarct regions of $BAT^{ZsGreen}$ recipients, whereas no such signal was detected in MI-only controls (Fig. 2a, b, and Extended Data Fig. 2a). These signals appeared as discrete intracellular puncta on three-dimensional visualization.

To further characterize these signals, we examined their spatial association with mitochondrial protein markers. In $BAT^{ZsGreen}$-recipient hearts, $ZsGreen^+$ puncta exhibited marked co-localization with TOMM20 (outer mitochondrial membrane), cytochrome c (intermembrane space), and ATP5A1 (inner mitochondrial membrane), with comparatively limited overlap with the matrix enzyme PDH (Fig. 2c, d, and Extended Data Fig. 2b). These findings support protein-level association of $ZsGreen^+$ puncta with multiple mitochondrial compartments.

2. The colors of α-actinin and CD68 in Figure 2g are too similar to distinguish.

Response:

In the revised figure, we have reassigned pseudocolors to maximize channel separation and visual clarity. Specifically, mtGFP is now displayed in cyan, CD68 in magenta, α-actinin in yellow, and nuclei in blue (Fig. R2). The underlying fluorescence signals and intensity relationships remain unchanged. This revised color scheme provides a clear visual distinction between cardiomyocytes and $CD68^+$ macrophages.

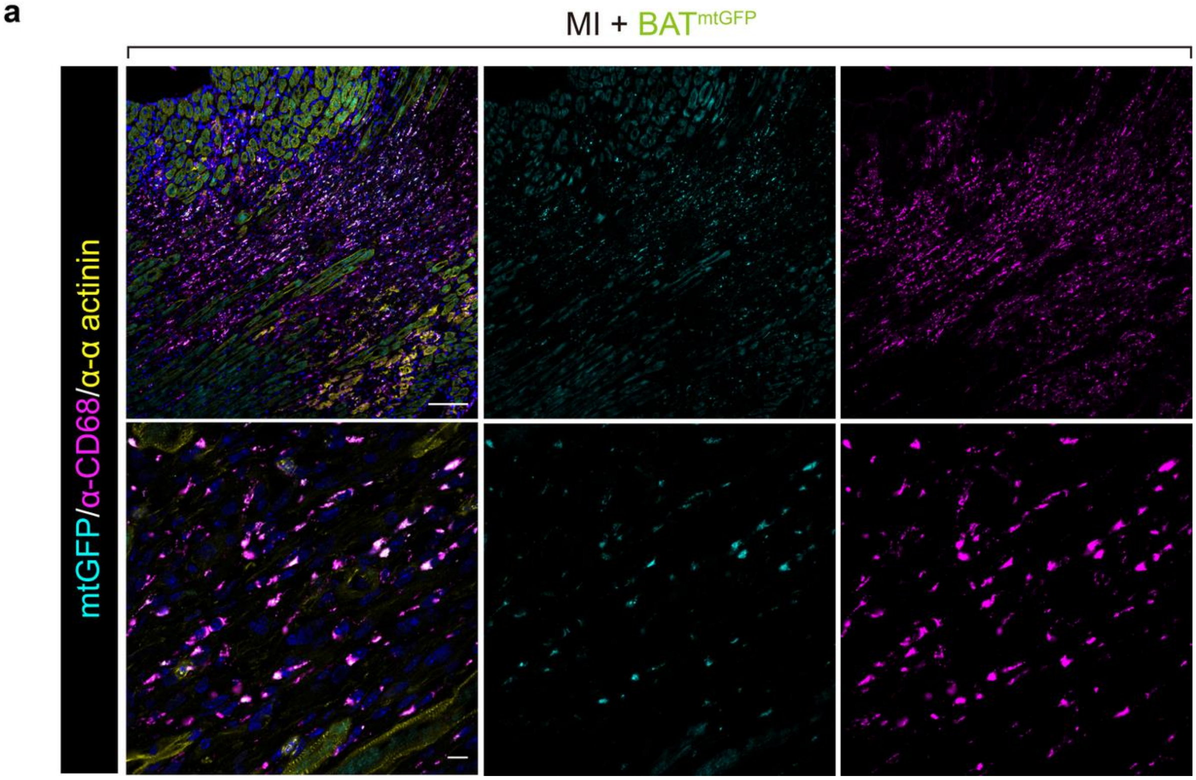

Fig. R2 a, Representative confocal images of infarct myocardium 4 days after MI in BAT^{mtGFP}-receiving mice. Sections were stained for sarcomeric α-actinin (yellow), CD68 (magenta), and nuclei (DAPI, blue). Upper scale bar: 100μm; lower scale bar: 10μm. **The above data are included in Fig. 2g in the revised manuscript.**

3. Figures 3d, e, and f indicate that equal amounts of MDVs derived from WAT and BAT exhibit identical promoting effects on the repair of cardiac structure and function following myocardial infarction. This should be further discussed in the text.

Response:

Under protein-normalized dosing conditions, BAT- and WAT-derived MDVs displayed comparable levels of representative oxidative phosphorylation proteins (Fig. 3c) and exerted similar cardioprotective effects after myocardial infarction (Fig. 3d-g). We therefore interpret these data to indicate that, at the level of individual vesicle, the intrinsic bioenergetic function is similar and comparable. Therefore, when vesicle dose is equalized, WAT-derived MDVs can reveal a cardioprotective potential comparable to that of BAT-derived MDVs, consistent with their similar OXPHOS cargo composition.

However, whole-tissue WAT transplantation did not confer detectable cardioprotection, in contrast to BAT transplantation (Fig. 1c-f), whereas isolated WAT-MDVs were protective when administered in equal amounts (Fig. 3d-g). We believe this divergence is likely explained by several factors.

First, WAT contains substantially fewer MDVs than BAT, with an approximately 5-10-fold lower MDV yield (Fig. 3b), making endogenous WAT-derived MDV flux to the heart relatively inefficient. By contrast, direct MDV injection circumvents this limitation by equalizing vesicle dose.

Second, BAT harbors greater mitochondrial abundance and metabolic activity, which likely further favors MDV production and efflux under pathological stress such as myocardial infarction.

Third, beyond MDVs alone, WAT releases a broader secretome that includes non-coding RNAs, fatty acids, and pro-inflammatory adipokines, which may promote systemic inflammation, adverse cardiac remodeling, and heart failure (PMID: 29500246; 31918577; 29773163). These detrimental signals could outweigh any modest benefit provided by the relatively small amount of endogenous WAT-derived MDVs after WAT transplantation, whereas purified WAT-MDVs administered in isolation can unmask the intrinsic cardioprotective potential of MDVs as a vesicle class. In contrast, BAT is generally

associated with a more beneficial secretory milieu, including endocrine factors and extracellular vesicles that support metabolic homeostasis and cardiac function (PMID: 30017353; 33106031; 37462036; 35652349). These broader depot-specific differences may further contribute to the divergent in vivo effects of BAT and WAT.

We have now clarified this distinction more explicitly in both the Results and Discussion sections.

Manuscript edits：

Results

BAT-MDVs contribute to MI injury repair

Notably, whole-tissue WAT transplantation did not confer detectable cardioprotection in contrast to BAT transplantation (Fig. 1c-f), whereas isolated WAT-MDVs were cardioprotective when administered in equal amounts. Several factors may account for this divergence. First, WAT contains substantially fewer MDVs than BAT (approximately 5-10-fold lower MDV yield, Fig. 3b), making endogenous WAT-derived MDV flux to the heart relatively inefficient, whereas direct MDV injection circumvents this limitation by equalizing vesicle dose. Second, WAT releases a complex secretome that includes non-coding RNAs, fatty acids, and proinflammatory adipokines, which are known to promote systemic inflammation, adverse cardiac remodeling, and heart failure[10,24]. These detrimental signals are likely to outweigh any modest benefit from the small amount of WAT-derived MDVs after WAT transplantation, whereas purified WAT-MDVs administered in isolation can reveal the intrinsic cardioprotective potential of MDVs as a vesicle class.

Discussion

At matched doses, isolated BAT- and WAT-derived MDVs showed comparable oxidative phosphorylation cargo and cardioprotective effects, indicating that the intrinsic bioenergetic activity of individual MDVs is not strictly depot-restricted. This, however, is distinct from the effect of whole-tissue transplantation in vivo, which integrates depot-specific differences in mitochondrial abundance, MDV output, and the broader secretory

profile. BAT likely confers a stronger benefit in this setting because it contains more mitochondria, releases more MDVs under stress, and provides a more favorable secretory profile for metabolic homeostasis and cardiac repair than WAT.

4. The authors used both Masson's trichrome and Sirius Red staining methods to demonstrate cardiac fibrosis levels. However, only one statistical graph is provided (Fig. 1e, f; Fig. 3f, g; Fig. 5e, g). Please provide the statistical results for both staining methods.

Response:

In the final manuscript, we now provide separate statistical analyses for Sirius Red staining alongside Masson's trichrome quantification in Fig. 1, Fig. 3, and Fig. 5. Fibrosis was quantified independently on adjacent sections using identical anatomical levels and thresholding criteria.

Sirius Red staining yielded results that were consistent with those obtained from Masson's trichrome staining across all experimental groups (Fig. R3). We have updated the corresponding figure panels, legends, and Methods section to indicate independent quantification of each staining method.

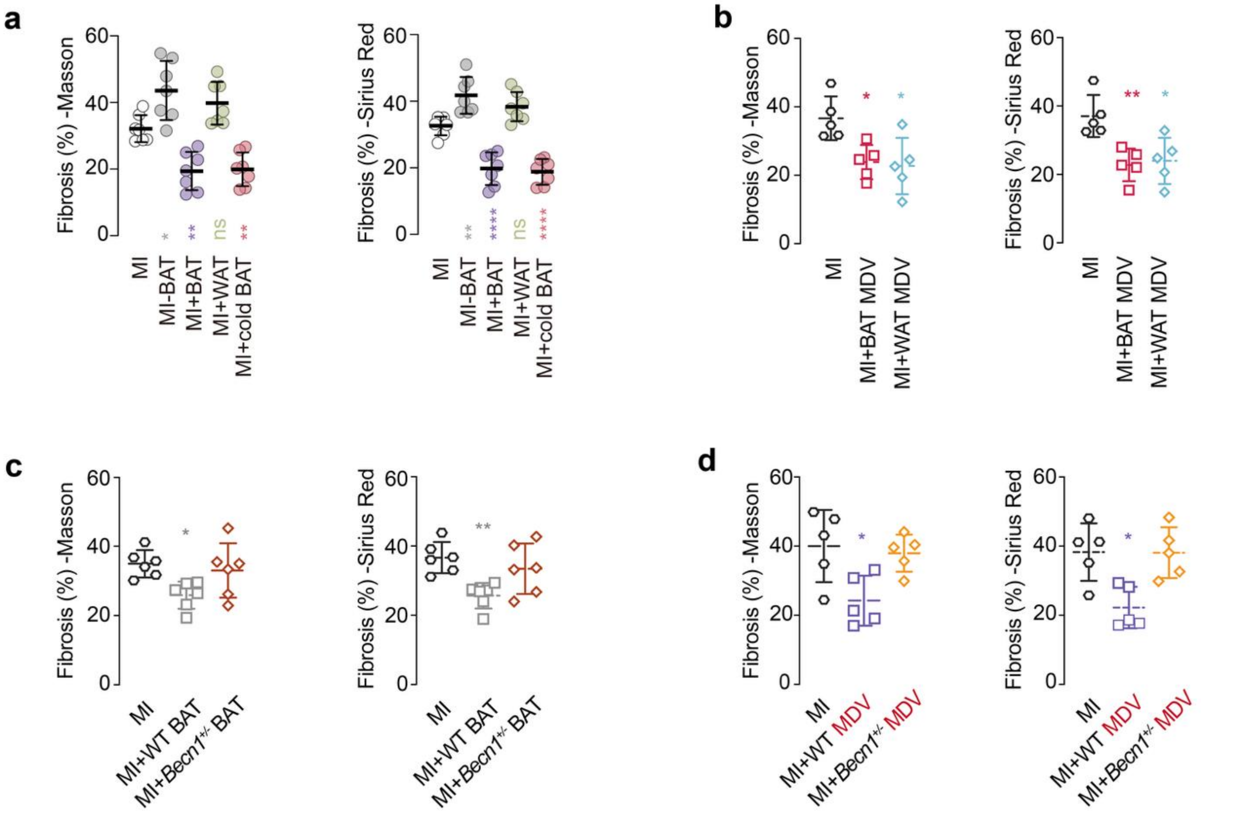

Fig. R3 a, Quantification of Masson and Sirius Red staining of BAT-receiving mouse hearts at day 28. **b,** Quantification of Masson and Sirius Red staining of MDV-treated mouse hearts at day 28. **c,** Quantification of Masson and Sirius Red staining of WT or *Becn1*$^{+/-}$ BAT-receiving mouse hearts at day 28. **d,** Quantification of Masson and Sirius Red staining of WT or *Becn1*$^{+/-}$ MDV-treated mouse hearts at day 28. (mean±s.d., One-way ANOVA test followed by Bonferroni method, *p < 0.05, **p < 0.01, ***p < 0.001, ****p < 0.0001). Scale bar: 2mm. **The above data are included in Fig. 1f, 3g, 5e, and 5g in the revised manuscript.**

Manuscript edits：

Methods

Masson trichrome and Sirius Red staining

Fibrotic regions appeared blue on Masson-stained sections and Sirius Red-positive on adjacent sections. Fibrosis was quantified independently for each staining method using identical anatomical levels and image-analysis parameters. For each section, the fibrotic area was defined as the positively stained region and presented as a percentage of total myocardial cross-sectional area. For each mouse, fibrosis (%) was calculated as the average across 5-6 serial sections spanning from the apex to the ligation level.
